# Whole-genome sequences of 89 Chinese sheep suggest role of *RXFP2* in the development of unique horn phenotype as response to semi-feralization

**DOI:** 10.1093/gigascience/giy019

**Published:** 2018-03-07

**Authors:** Zhangyuan Pan, Shengdi Li, Qiuyue Liu, Zhen Wang, Zhengkui Zhou, Ran Di, Benpeng Miao, Wenping Hu, Xiangyu Wang, Xiaoxiang Hu, Ze Xu, Dongkai Wei, Xiaoyun He, Liyun Yuan, Xiaofei Guo, Benmeng Liang, Ruichao Wang, Xiaoyu Li, Xiaohan Cao, Xinlong Dong, Qing Xia, Hongcai Shi, Geng Hao, Jean Yang, Cuicheng Luosang, Yiqiang Zhao, Mei Jin, Yingjie Zhang, Shenjin Lv, Fukuan Li, Guohui Ding, Mingxing Chu, Yixue Li

**Affiliations:** 1 Institute of Animal Science, Chinese Academy of Agricultural Sciences, Beijing, China; 2 College of Agriculture and Forestry Science, Linyi University, Linyi, China; 3 Key Lab of Computational Biology, CAS-MPG Partner Institute for Computational Biology, Shanghai Institutes for Biological Sciences, Chinese Academy of Sciences, Shanghai, China; 4 University of Chinese Academy of Sciences, Beijing, China; 5 State Key Laboratory for Agrobiotechnology, China Agricultural University, Beijing, China; 6 BasePair BioTechonology Co., Ltd., Suzhou, China; 7 Institute of Biotechnology, Xinjiang Academy of Animal Science, Urumqi, China; 8 Institute of Animal Science, Xinjiang Academy of Animal Science, Urumqi, China; 9 Research Institute of Animal Science, Tibet Academy of Agricultural and Animal Husbandry Sciences, Lhasa, China; 10 College of Life Science, Liaoning Normal University, Dalian, China; 11 College of Animal Science and Technology, Agricultural University of Hebei, Baoding, China; 12 Shanghai Center for Bioinformation Technology, Shanghai Industrial Technology Institute, Shanghai, China

**Keywords:** domestic animal, sheep, adaptive evolution, artificial selection, semi-feralization, horn

## Abstract

**Background:**

Animal domestication has been extensively studied, but the process of feralization remains poorly understood.

**Results:**

Here, we performed whole-genome sequencing of 99 sheep and identified a primary genetic divergence between 2 heterogeneous populations in the Tibetan Plateau, including 1 semi-feral lineage. Selective sweep and candidate gene analysis revealed local adaptations of these sheep associated with sensory perception, muscle strength, eating habit, mating process, and aggressive behavior. In particular, a horn-related gene, *RXFP2*, showed signs of rapid evolution specifically in the semi-feral breeds. A unique haplotype and repressed horn-related tissue expression of *RXFP2* were correlated with higher horn length, as well as spiral and horizontally extended horn shape.

**Conclusions:**

Semi-feralization has an extensive impact on diverse phenotypic traits of sheep. By acquiring features like those of their wild ancestors, semi-feral sheep were able to regain fitness while in frequent contact with wild surroundings and rare human interventions. This study provides a new insight into the evolution of domestic animals when human interventions are no longer dominant.

## Introduction

Animal domestication has been widely investigated to better understand the phenotypic and genetic changes of animals caused by human activities [1–4]. However, the process in which domestic animals become feral is still poorly understood. Domestication is the process where protection offered by domestic habitat suppresses the original environmental adaptation. Feralization is its reverse; the animals restart to fit natural life while human artificial selections are no longer dominant [5].

The history of Chinese sheep domestication can be traced back more than 5000 years according to archeological evidence [6, 7]. The demographic history of Chinese domestic sheep was recently reconstructed based on population genomics, which suggested their origin on the Mongolian Plateau about 5000 to 7000 years ago with later dispersal associated with historical movements of nomadic societies [8]. To date, more than 42 local breeds of sheep have been established in China, comprising lineages from 2 major geographic areas known as northern China, the Tibetan Plateau, and the Yunnan-Kweichow Plateau [8, 9]. Sheep in northern China were also denoted as Mongolian sheep because of their distinctive fat-storage phenotypes (fat tails or rumps) [10, 11]. Tibetan and Yunnan-Kweichow sheep were split from Mongolian sheep about 4000 years ago [8].

Different climate zones have an essential impact over the adaptive evolution of the major ovine lineages in China [9]. However, the role of various husbandry cultures in affecting the phenotypes of modern sheep breeds is not well understood. In fact, the unique domestication history and husbandry system of Tibetan sheep make it an appropriate evolutionary model for studying animal semi-feralization for several reasons. First, because the Tibetan Plateau is rich in grassland, the local breeds, especially those living on prairies, have been roaming with nomads and fed on natural ranches. Second, these sheep were forced to encounter threats from the wild (e.g., Tibetan wolves) because of a sparsely populated and undeveloped environment. Third, unlike in other pastoral areas of China, the breeding of Tibetan sheep was not subject to intense artificial control such as gender-separating management and selective breeding. In this case, the evolution of these semi-feral populations can provide indications about how domestic animals adapt when artificial pressures are loosened.

To enhance the understanding of animal feralization, we sequenced and analyzed the genomes of 30 sheep from 2 semi-feral breeds and 1 domestic breed from the Tibetan Plateau and 69 domestic sheep from other geographic areas. We identified a primary divergence in Tibetan sheep and a set of candidate loci underlying selective sweeps in each Tibetan breed, which is responsible for their distinct phenotypic patterns related with semi-feralization.

## Data Description

**Figure 1:**
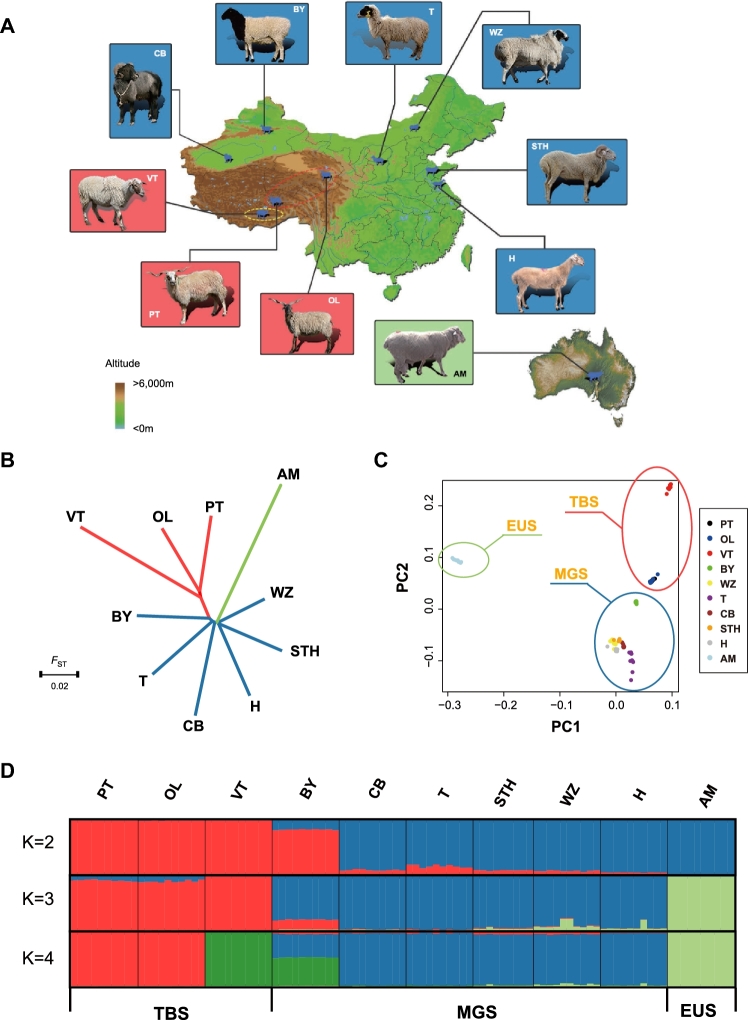
Genetic relationships and population structure in Chinese sheep. a) Geographic distribution of the Chinese indigenous sheep breeds (PT, Prairie Tibetan; OL, Oula; VT, Valley Tibetan; BY, Bayinbuluke; WZ, Wuzhumuqin; T, Tan; CB, Cele Black; STH, Small-tailed Han; H, Hu) and a European-originated breed (AM, Australian Merino) sampled in the present study. The background color of the sheep pictures represents their lineages (red: TBS, Tibetan sheep; blue: MGS, Mongolian sheep; green: EUS, European sheep). b) Neighbor-joining tree of the 10 breeds based on *F*_ST_ distances. c) Principal component plot. The first (PC1) and second (PC2) principal components are shown. d) Population structure analysis of 99 sheep, where number of ancestral clusters were set from K = 2–4.

We selected 30 sheep from 3 typical Tibetan breeds in the Tibetan Plateau (PT, Prairie Tibetan sheep; VT, Valley Tibetan sheep; OL, Oula sheep), 59 sheep from 6 Mongolian breeds across northern China (BY, Bayinbuluke sheep; CB, Cele Black sheep; H, Hu sheep; T, Tan sheep; STH, Small Tail Han sheep; WZ, Wuzhumuqin sheep), as well as 10 Australian Merino sheep (AM) representing a European-originated breed (Fig. 1a, **Supplementary Tables S1 and S2**). Among the 10 breeds, PT and OL were the 2 semi-feral populations that did not receive extensive human interventions, while PT, OL, VT, and BY were 4 populations living at high altitude (>3000 m above sea) (**Supplementary Table S2**). The sex ratio was maintained at approximately 1:1 for each breed. We performed whole-genome sequencing (WGS) of the 99 sheep. The coverage depth after genome alignment was approximately 6-fold for each individual (**Supplementary Tables S3 and S4**), resulting in more than 50× coverage depth for each breed.

## Materials and methods

### Sample collection and sequencing

A total of 89 Chinese sheep from 9 diverse breeds as well as 10 Australian Merino sheep from Australia were sequenced (**Supplementary Tables S1 and S2**). For each sheep, genomic DNA was extracted from 200 μl of peripheral venous blood using the QIAamp DNA blood mini kit (Qiagen, Germany). The quality and integrity of the DNA was assessed using the A260/280 ratio and agarose gel electrophoresis. For sequencing library preparation, the genomic DNA was sheared to fragments of 300–400 bp and subsequently end-repaired, “A”-tailed, and ligated to Illumina sequencing adapters. The ligated products with sizes of 400–500 bp were selected on 2% agarose gels and subsequently amplified by ligation-mediated polymerase chain reaction (PCR). The libraries were sequenced on an Illumina HiSeq 2500 sequencer in 2 × 100 bp paired-end mode and controlled using Illumina HiSeq control software.

### Variant calling

The raw reads were processed using 2 quality control (QC)steps: (1) reads with adapter contamination were removed and (2) reads with more than 10% ambiguous bases were excluded. Read filtering statistics were shown in Supplementary Table S3. Only paired reads were preserved after QC. The filtered reads were subsequently mapped to the sheep reference genome assembly oviAri3 [12] using BWA, version 0.7.12 (BWA, RRID:SCR_010910) [13] for all individuals separately (Supplementary Table S4). PCR duplicates were removed using PICARD, version 1.135 [14]. Indels were realigned using GATK, version 3.2-2 (GATK, RRID:SCR_001876) [15]. SNPs and indels were called using SAMtools, version 1.2 [13] after pooling samples from the same breed (Supplementary Table S5-S7). After SNP calling, the variants were filtered using vcfutil.pl varFilter, with a “-d 20 -D 100” parameter to remove low-quality SNPs and indels. Targeted sequencing (Sanger) of 5 random genomic regions was performed, and the results were used to estimate the false positive rate and false negative rate of SNP calling step (**Supplementary Tables S28 and S29**). After filtering, the variants were annotated using snpEff, version 4.0e [16] according to the National Center for Biotechnology Information (NCBI) annotation [17].

### Population genetics analysis

Pair-wise genetic distances were measured by the number of allele differences for genomic SNP sites. The neighbor-joining tree was calculated based on the distance matrix using PHYLIP, version 3.69 [18]. To place a root for the phylogeny tree, we aligned the goat genome sequence [19] with the sheep reference genome sequence using LASTZ, version 1.02 [20], and used the homologous sites of goat to determine the ancestral alleles for each SNP. Only biallelic autosomal SNPs were used to calculate the distance matrix. PCA was performed using EIGENSOFT, version 6.0.1 [21, 22], and population structures were inferred using FRAPPE software, version 1.1 [23]. Both the PCA and population structures were calculated based on autosomal SNPs after removing highly correlated SNP pairs using PLINK, version 1.07 (PLINK, RRID:SCR_001757) [24], with the “-indep-pairwise 50 5 0.2” parameter. Migration events among sheep breeds were estimated using TreeMix [25] with migration number *m* = 0–5. Statistics including *π* (pair-wise nucleotide differences), *θ* (number of segregating sites), SNP densities, and Tajima's *D* were calculated using VCFtools, v0.1.12b [26]. The linkage disequilibrium *r*^2^ was calculated using Haploview [27] based on 500 000 SNPs randomly selected from the genome. The parameters were set as “–missingCutoff 0.2 –dprime –minMAF 0.1.” The SNP pairs were grouped according to the physical distances between them. The mean *r*^2^ was adopted to represent the average LD for each group (e.g., 0 ∼ 1 kb).

### Selective sweep analysis

Selective sweeps across the sheep genome in 4 populations, PT, OL, VT, and BY, were detected by comparison with MGS, based on fixation index *F*_ST_ and heterozygosity log_2_(*H*_P_ ratio) over a 30-kb sliding window with a step of 15 kb (Supplementary Table S10–S14). *F*_ST_ distances between population were calculated using the Bio::PopGen::PopStats package in BioPerl [28]. The pooled heterozygosity *H*_P_ for population was calculated using the formula *H* = 2∑*p*∑*q*/(∑*p*+∑*q*)^2^, where ∑*p* represents the sum of the major allele frequencies of all SNP sites in the window and ∑*q* represents the sum of the minor allele frequencies [4]. The log_2_(*H*_P_ ratio) between population A and B was calculated as log_2_(*H*_P|B_/*H*_P|A_), which reflected the loss of heterozygosity in A relative to B. A total of 46 windows were excluded because of extremely small variant numbers (<50 variants) (**Supplementary Table S15**). We considered the windows with the top 5% values as the significance threshold for single statistic (e.g., *F*_ST|A vs. B_ > *F*_ST|5%_, where *F*_ST|5%_ denotes the top 5% threshold of *F*_ST|A vs. B_). A 30-kb region was defined as a selective sweep in population A if it had both *F*_ST|A vs. MGS_ and log_2_(*H*_P|MGS_/*H*_P|A_) over the threshold.

All annotated genes overlapped with sweep windows or their flanking windows (15-kb up- and down-stream the sweep region) were defined as candidate genes. Furthermore, the cross-population extended haplotype homozygosity [24] was estimated between PT vs MGS, OL vs MGS, VT vs MGS, and BY vs MGS for candidate sweep region, based on haplotype data phased by fastPHASE [29]. GO functional enrichment analysis of the candidate genes was performed using ClueGO [30], in which the *P* values were corrected using the Benjamini-Hochberg approach (**Supplementary Tables S16–S21**). Then, a list of candidate genes related with semi-feralization were summarized according to their functional categories (Supplementary Table S22). Protein-altering mutations were extracted from selective sweep windows to identify potential functional variants (**Supplementary Tables S23–S26**).

### Validation of SNP genotypes in large population

We collected 1155 additional venous jugular blood samples from sheep of 10 breeds, including 100 AM sheep, 100 PT sheep, 98 VT sheep, 87 OL sheep, 100 BY sheep, 80 CB sheep, 100 H sheep, 100 T sheep, 100 WZ sheep, and 290 STH sheep. Genomic DNA was extracted using the phenol-chloroform method and dissolved in TE buffer (10 mM Tris-HCl [pH 8.0] and 1 mM EDTA [pH 8.0]). To validate the allele frequency of the 2 differentiated protein altering SNPs in *RXFP2* (**Supplementary Tables S23 and S24**), we performed a multiplex screening assay (SNaPshot) [31] on these 1155 individuals. We designed amplification and SNaPshot single-base extension primers **(Supplementary Table S27)**. Genotyping was performed using the SNaPshot™ multiplex kit (ABI) according to the manufacturer's instructions and analyzed using the ABI Genetic Analyzer 3730XL.

### Association study between the *RXFP2* genotype and horn phenotypes

Nine SNPs within or near the *RXFP2* locus (**Supplementary Table S28**) were genotype in 182 PT sheep with 5 horn types: polled (0 cm), scurred (0–12 cm), TCF type (>12 cm, tightly close to the face), SHE-type (>12 cm, spiral and horizontally extended), and uncertain type (>12 cm, uncertain shape) (**Supplementary Table S29**). One of 9 SNPs was ignored because no variations were observed among those sheep. Correlations between SNP genotypes and horn phenotypes (horn size, horn shape) were estimated using linear or logistic regressions (performed with in-house R language scripts), depending on the variable type of outcome. Three genetic models (recessive, additive, and dominant) were applied for each pair of association tests. Confounding effects of individual age and sex were tested by considering them as covariates in the model.

### Gene expression analysis of *RXFP2* and its flanking genes

Tissue expression levels of the 5 genes located within the 1-Mb region encompassing the *RXFP2* locus, including *RXFP2*, *B3GLCT*, *FRY*, LOC101110773 (*EF1A1L*), and LOC106991357 (ncRNA), were examined by Reverse Transcription PCR (RT-PCR). Primer sequences are shown in **Supplementary Table S27**. We studied 13 tissues of TBS and 21 tissues of Sonid sheep. For each tissue type, equal volumes of cDNA from 6 individuals (2 individuals from each horn type) were mixed as pooled cDNA samples. RT-PCR reactions were carried out in 50 μl volume including Taq DNA polymerase (5 U/μl) (TaKaRa, Dalian, China) 0.25 μl, 10 × PCR buffer (+MgCl_2_) 5μl, 10mM dNTPs (2.5 mM each) 4 μl, each primer (10 μM) 1 μl, cDNA 1 μl, ddH_2_O 37.75 μl. Amplification conditions were set as initial denaturation at 95°C for 5 min, followed by 33 cycles of denaturation at 95°C for 30 s, annealing for 20 s at appropriate temperatures, and extension at 72°C for 10 s, with a final extension at 72°C for 2 min on Mastercycler 5333 (Eppendorf AG, Hamburg, Germany). The PCR product was mixed with 5 μl loading buffer (6×) and loaded 5 μl into 1% sepharose gel. After 15 min of 180 mA electrophoresis, a picture under the Biorad GelDoc XR System (Bio-rad, USA) was taken.

Expressions of the 5 genes were measured by real-time PCR in 13 PT sheep soft-horn samples with different horn types. Four to 5 biological replicates were selected from different individuals of the same horn type (4 SHE type, 4 TCF type, and 5 scurred). For all 5 genes and internal control, real-time PCR was performed 3 times in 1 sample as technical replicates, and the average gene expressions of 3 replicates were calculated. Real-time PCR amplification was performed in 20 μl of reaction mixture containing 2 μl of cDNA, 0.4 μl of each forward and reverse primer (10 μM), 0.4 μl of ROX reference dye II (50×), 10 μl of SYBR Green real-time PCR Master Mix (2×), and 6.8 μl of ddH_2_O. The reaction without template was treated as the blank control. PCR amplification was performed in triplicate wells under the following conditions: 95°C for 30 s, followed by 40 cycles of 95°C for 5 s and 60°C for 34 s. The melting curve was analyzed after amplification. The peak Tm on the dissociation curve was used to determine the specificity of PCR amplification. Standard curves of these genes were also constructed. *β*-actin expressions were used as the internal control among samples. Relative expression levels of 5 genes were calculated based on the expression of *RXFP2* in the SHE-type soft horn (its expression was defined as 1.0). The 2^−ΔΔCt^ method was used to process the real-time PCR results [32].

Protein extracts from soft-horn tissues were prepared by complete homogenization of tissues in an immunoprecipitation buffer (Beyotime, CA, USA) according to the manufacturer's instructions. Equal amounts of protein extracts were mixed with sample buffer and then separated on 10% SDS-PAGE gels (60 μg/lane). Details of the Western blot process have been described previously [33]. Rabbit Anti-GPR106 antibody (BIOSS, Beijing, China), polyclonal rabbit anti-mouse *β*-actin antibody (Abcam, USA), and goat anti-rabbit IgG, HRP (Santa Clara, CA, USA) were used.

## Results

### Characterization of the variants

After applying stringent criteria in quality control, we identified 38 090 348 single-nucleotide polymorphisms (SNPs) and 4 348 493 insertions/deletions (indels) in the 99 genomes (**Supplementary Table S5**). The abundance of variants was comparable to those of other domestic animals [4, 34, 35]. Most variants were intergenic or intronic, and only 269 584 SNPs and 5518 indels were exonic (**Supplementary Tables S6 and S7**). Our dataset captured >94.0% (26 598 869 SNPs and indels) of the variants in the dbSNP database build 143, whereas >37.3% (15 839 972 SNPs and indels) of the variants in the 99 sheep genomes are novel (**Supplementary Fig. S1**). The genome-wide average diversity *π* of the sheep breeds was estimated to be 2.44–2.84 × 10^−3^, which was similar as previously reported [9]. In other domestic animals such as pigs and dogs, nucleotide diversity in Tibetan breeds is often higher than in other Chinese breeds [34, 35]. However, our data suggested domesticated that sheep in China have an opposite trend; Tibetan sheep breeds (*π* = 2.44–2.61 × 10^−3^, *θ* = 2.10–2.30 × 10^−3^) have lower nucleotide diversity than Mongolian (*π* = 2.69–2.79 × 10^−3^, *θ* = 2.36–2.52 × 10^−3^) and European breeds (*π* = 2.84 × 10^−3^, *θ* = 2.50 × 10^−3^), which is consistent with the fact that Mongolian sheep diverged earlier than Tibetan sheep from their ancestral lineage [36].

### Population genetics of Chinese sheep

To understand the genetic relationships among these local breeds, we constructed a neighbor-joining tree based on their pairwise genetic distances (measured by fixation index *F*_ST_) (Fig. 1b). We also calculated a phylogenetic tree based on genomic SNPs to visualize the relationship between individual samples, where a goat genome was used to calibrate the root (**Supplementary Fig. S2a**). As expected, the European-originated sheep (AM and Texel) were the first clade separated from the ancestral lineage, followed by the Mongolian breeds and, finally, the Tibetan breeds. This phylogeny structure is again consistent with the migration trajectory of sheep, where Eurasian sheep initially migrated onto the Mongolian Plateau and then spread into local areas of China [36]. The 3 Tibetan sheep breeds formed a monophyletic clade that was robust under bootstrapping tests (**Supplementary Fig. S2b**), indicating a common origin of Tibetan sheep from 1 recent ancestral lineage.

Next, we performed a principal component analysis (PCA) of 99 sheep based on their genomic variants (Fig. 1c). Despite the division among Tibetan sheep (TBS), Mongolian sheep (MGS), and European sheep (EUS), a considerable genetic difference was observed between 2 groups of Tibetan sheep: 1 cluster consisted of 20 individuals from 2 semi-feral breeds, PT and OL, while another consisted of 10 individuals from domestic breed VT (Fig. 1c). We further examined the population structure by assuming the number of ancestry K (Fig. 1d, **Supplementary Fig. S3**). When K = 3, TBS, MGS, and EUS were clearly separated, though BY, 1 breed of MGS, showed a mixture between TBS and MGS. When K = 4, we observed a primary divergence between semi-feral and domestic TBS, in agreement with the PCA result. In addition, analysis with TreeMix [25] confirmed the migration event from VT to BY (**Supplementary Fig. S4**). Due to its genetic admixture, BY was treated separately from other MGS breeds during subsequent analysis.

A previous study of native sheep in China included samples from 4 Tibetan populations (labeled as ZRK, ZLZ, ZNQ, and ZCD) [9]. Here, we provide a supplementary map to summarize their geographical locations and relationship with Tibetan breeds in the present study (**Supplementary Fig. S5**). Briefly, VT, ZRK, and ZLZ were in southern Tibet, while PT, OL, ZNQ, and ZCD were in the north. OL was at a relatively distant area from other local breeds. However, as we observed high similarity between OL and PT, it seems that the geographical distance was not the only determinant of the genetic differences between breeds.

An intriguing phenomenon is that the domestic TBS breed VT seems to show a unique breeding history, represented by its slow linkage disequilibrium (LD) decay and the most positive Tajima's *D* statistics across the genome compared with other breeds (**Supplementary Fig. S6**). These statistics suggest that VT has encountered the most severe contraction of population size. These sheep also showed lower genetic diversity (*π* = 2.44 × 10^−3^) than semi-feral TBS (*π* = 2.60–2.61 × 10^−3^), MGS (*π* = 2.69–2.79 × 10^−3^) and EUS (*π* = 2.84 × 10^−3^). Moreover, population ZLZ in the other study was proximate to VT and also exhibits slow LD decay as a sign of population bottleneck [9].

### Selective sweeps in semi-feral and domestic sheep

We reasoned that different levels of human intervention might have resulted in distinct evolutionary trajectories of PT, OL, and VT. For example, PT and OL raised by nomads were typically free roaming, while VT were captive, intensively managed by local farmers for improving productions and efficiencies (**Supplementary Table S2**). PT and OL live in underdeveloped regions of north Tibet, where human population is sparse (**Supplementary Fig. S7**), suggesting less interaction with human society and more threats from predators (e.g., Tibetan wolves). Moreover, VT was subject to moderate selective breeding, while PT and OL received barely any intervention in their mating process (**Supplementary Table S2**)

**Figure 2:**
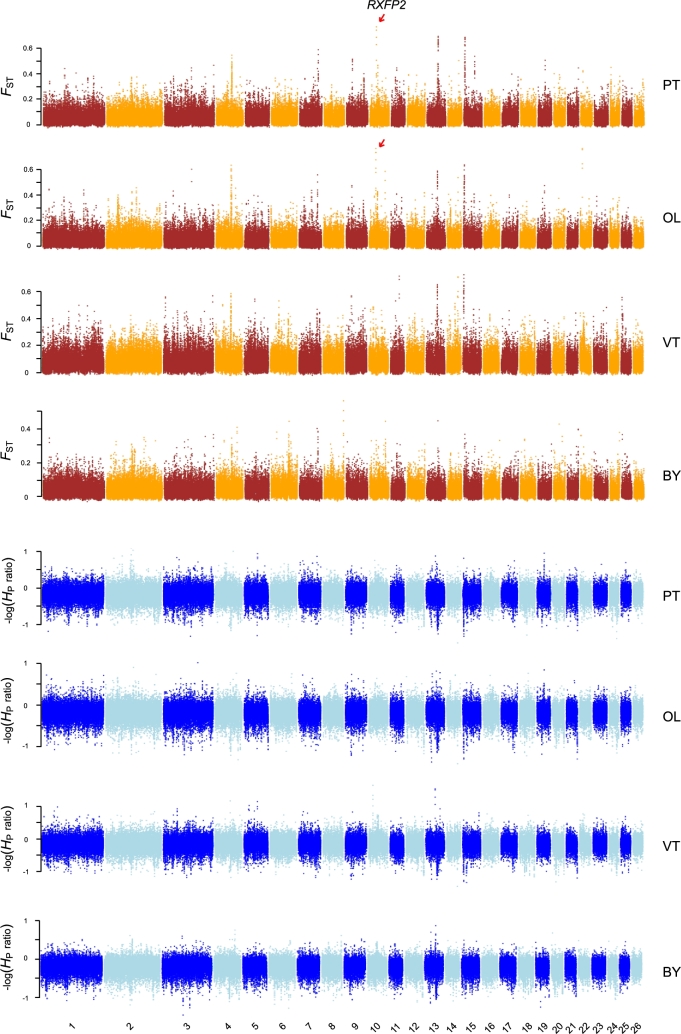
Manhattan plot of genome-wide selective sweep signals (*F*_ST_ and log-scaled *H*_P_ ratio) in 4 sheep breeds. For each metric, a 30-kb sliding window with a step size of 15 kb was applied. *F*_ST_ distances were calculated between each of the 4 breeds (PT, OL, VT, or BY) vs MGS (WZ, T, STH, H, and CB). The log-scaled *H*_P_ ratio was calculated as −log_2_(*H*_P|PT, OL, VT or BY_/*H*_P|MGS_), a positive value of which suggests reduction of variability in the breed.

**Figure 3:**
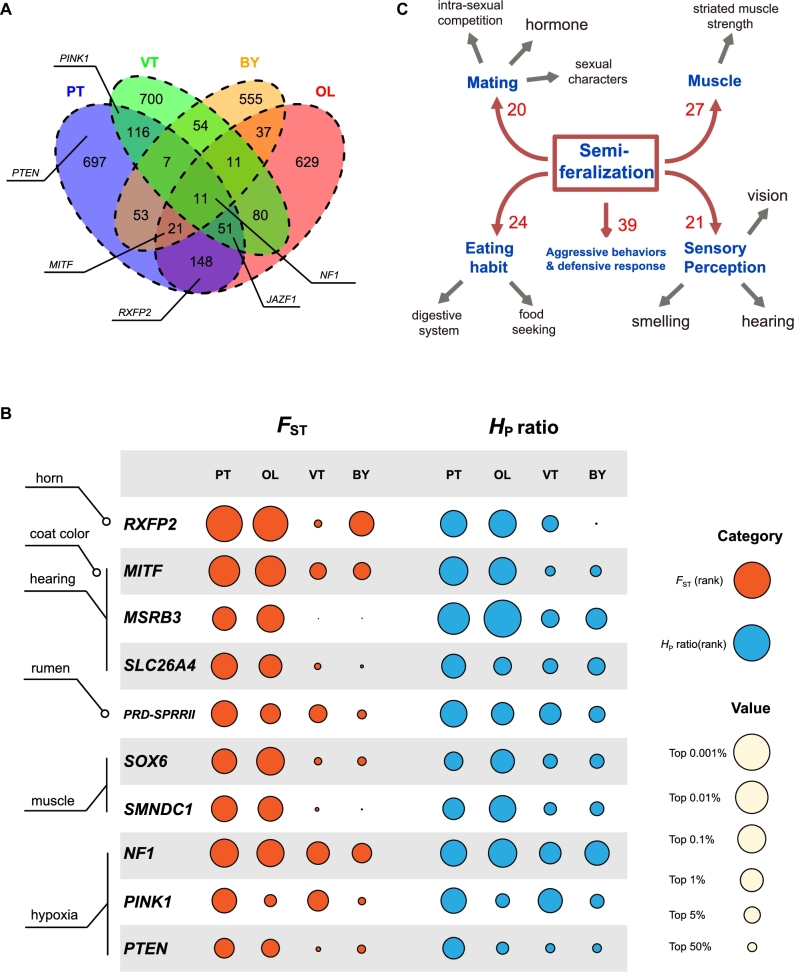
Candidate genes associated with selective sweeps in semi-feral sheep. a) A Venn plot showing numbers of overlapping candidate genes among 4 breeds (PT, OL, VT, and BY). b) Sweep signal metrics for genes selected from feralization-related categories as well as 3 genes associated with hypoxic adaptation. c) A summary of feralization-related adaptation observed in semi-feral sheep. Affected functional terms were manually summarized based on gene ontology enrichment analysis of the candidate genes, as well as literature mining. Numbers denote the count of candidate genes within each major category.

To identify candidate genes under positive selection in different TBS populations, we performed a selective sweep analysis over the whole genome based on population differentiation (fixation index *F*_ST_) and loss of heterozygosity (heterozygosity log_2_[*H*_P_ ratio]) in PT, OL, VT, and BY, respectively by comparing them with MGS (Fig. 2). BY is not a TBS breed but is included here to identify loci potentially under altitude adaptations (PT, OL: semi-feral group; PT, OL, VT: Tibetan group; PT, OL, VT, BY: high-altitude group) (**Supplementary Table S2**). In total, we identified 1104, 988, 1030, and 749 candidate genes in each of the 4 populations (Fig. 3a, **Supplementary Tables S10–S13**).

In 2 semi-feral populations, we observed a consistently strong signal of positive selection on chromosome 10, which harbors a relaxin/insulin-like family peptide receptor 2 (*RXFP2*) gene (Fig. 2, **Supplementary Tables S10 and S11**). *RXFP2* is a well-known gene related to sheep horn phenotypes and is often characterized as a target of natural and sexual selection in wild and feral populations [37, 38]. Since free mating is one of the typical features of wild and feral populations and is often replaced with selective breeding in domestic lines, *RXFP2* potentially serves as the genetic marker of “wildness” in sheep, which confers their essential sexual weaponry during competitions to reproduce.

In addition to *RXFP2*, we also characterized a number of PT and OL candidate genes with a significantly high window *F*_ST_ and *H*_P_ ratio that are functionally plausible for adaptation in the wild (Fig. 3b). For example, these genes include (1) *MITF*, *MSRB3*, and *SLC26A4* associated with hearing [39–48]; (2) *SMNDC1* and *SOX6* involved in muscle development [47, 48]; and (3) *PRD-SPRRII* associated with regulating rumen development [49]. Their signals of selective sweep in 4 populations were in rough agreement with different extents of human intervention, where high *F*_ST_ and low *H*_P_ values were often restricted to PT and OL and were absent in VT and BY (Fig. 3b). Moreover, it is worth noting that some of these candidates are known to mediate diverse phenotypes such as *MITF* variants that contribute to coat color patterns [50–52]. In such cases, additional information such as phenotypic data will be necessary to define the real outcome of positive selection.

Then, we performed a gene ontology (GO) enrichment analysis of the gene sets in sweep regions of the 4 populations: PT, OL, VT, and BY (**Supplementary Tables S16–S19**). We also analyzed enriched GO terms in overlapping gene sets representing the combinations of semi-feral sheep (PT and OL, gene number = 231) and Tibetan sheep (PT, OL, and VT, gene number = 62) (**Supplementary Tables S20 and S21**). The results showed a number of feralization-related functional terms overrepresented in PT, OL, or their overlapping candidate genes (Fig. 3c, **Supplementary Table S22**). For example, a set of key terms was found related with the process of mating and reproduction, such as androgen receptors (GO:00 50681, GO:00 30521), the maternal process in female pregnancy (GO:00 60135), and hormone metabolisms (GO:00 46887, GO:00 32353). Categories associated with muscle function, such as striated muscle development (GO:00 14706), muscle cell apoptosis (GO:00 10656, GO:00 10657), and muscle adaptation (GO:00 43500, GO:00 43502), were also characterized. Moreover, other enriched functions include aggressive behaviors (GO:0 002118), defense response (GO:00 31347), digestive system development (GO:00 55123, GO:00 48565), as well as a number of GO clusters in sensory organ development (GO:0 001754, GO:00 42461, GO:00 42462, GO:00 46530, GO:00 48592, GO:00 48593, GO:00 21772). All functional terms mentioned above had a significant enrichment score (*P* value < 0.05) after considering multiple testing errors (by the Benjamini-Hochberg approach). Taken together, these findings suggested that 2 semi-feral lineages of TBS have undergone diverse processes of semi-feralization in response to the natural environment and reduced human protections. The adaptation potentially brought them with advantages through free mating, improved muscle strength, and food digestion abilities and promoted aggressiveness, defensive responses, and sensory perceptions in order to survive attacks from predators.

On a different note, our data also provide new evidence of the hypoxic adaption of TBS. By comparing our candidate gene list with those from 2 other studies of TBS [9, 53], we found *NF1* as a consistent signal in response to altitude adaptation identified among independent approaches (**Supplementary Table S14**). Our results indicated that *NF1* was under positive selection in all 4 high-altitude populations (Fig. 3b). Nevertheless, it is intriguing that other possible candidate genes of altitude adaptations, such as *PTEN* and *PINK1*, more often exhibit lineage-specific signals (Fig. 3b). Since the response to hypoxia is a well-known cellular process of polygenic basis, it is possible that the trajectories of hypoxia adaptation are highly heterogeneous among independent populations (an existing example is the Tibetan chicken [54]). Thus, it may explain the nonuniform distribution of hypoxia-associated sweeps among the 4 high-altitude populations.

### A horn-related locus *RXFP2* underlies positive selection in semi-feral sheep

**Figure 4:**
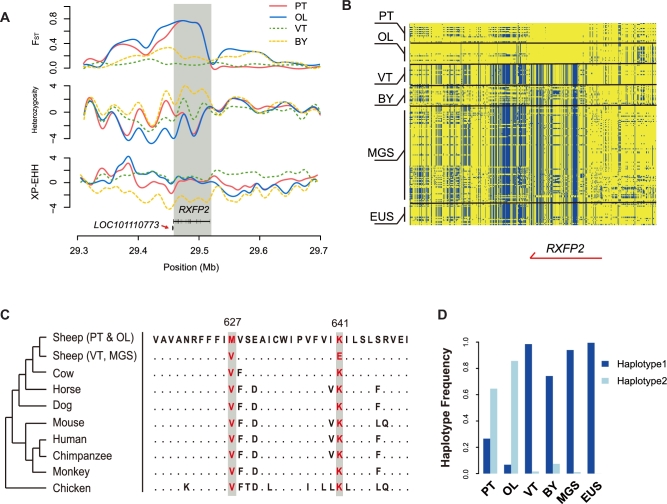
Selective sweep over the horn-related gene *RXFP2*. a) Statistics plotted over a approximately 400-kb region surrounding *RXFP2*, including 1) population differentiation (*F*_ST_) between PT, OL, VT, and BY vs MGS; 2) intrapopulation heterozygosity in PT, OL, VT, and BY, calculated as Z-transformed log_2_(*H*_P|PT, OL, VT or BY_/*H*_P|MGS_); 3) haplotypic length measured by Z-transformed XP-EHH_PT, OL, VT or BY vs. MGS_. b) Haplotypic distributions among 99 sheep of a local region of *RXFP2* (chromosome 10: 29 400 000–29 550 000 bp). Biallelic SNPs are shown in blue and yellow. c) Alignment of the *RXFP2* protein sequences from 9 vertebrate species. Two protein variants (*RXFP2*: 627 and 641) with top *F*_ST_ in PT and OL are indicated in red. For 627, PT and OL have the variant allele, whereas for 641, they have the reference allele. The dots in the alignment denote amino acids that are identical with those in PT and OL. d) Distribution of the haplotype frequency of 2 protein-altering variants (*RXFP2*: 627 and 641) in 1155 sheep. “Haplotype1” corresponds to V627 + E641 (OAR10_29 461 968: C + OAR10_29 462 010: C) and “Haplotype2” corresponds to M627 + K641 (OAR10_29 461 968: T + OAR10_29 462 010: T).

Next, we investigated and validated the strongest adaptive signature of semi-feralization in PT and OL, at chromosome 10 spanning a 60-kb region of *RXFP2* gene (Fig. 2). The sweep region exhibits excess of population differentiation (*F*_ST|PTvsMGS_ = 0.736, *F*_ST|OLvsMGS_ = 0.736) and a dramatic loss of heterozygosity (*H*_P|PT_/*H*_P|MGS_ = 0.768, *H*_P|OL_/*H*_P|MGS_ = 0.757) in 2 semi-feral breeds (Fig. 4a, **Supplementary Tables S10 and S11**). Nevertheless, neither of these signals was observed in VT (*F*_ST|VTvsMGS_ = 0.052, *H*_P|VT_/*H*_P|MGS_ = 0.976), while BY exhibited a moderate increase in genetic differentiation (*F*_ST|BYvsMGS_ = 0.187) but with no evidence of heterozygosity depletion (*H*_P|VT_/*H*_P|MGS_ = 1.519). The pattern of SNPs located in the *RXFP2* gene region revealed a unique haplotype in PT and OL, which was obviously different from those in VT, BY, MGS, and EUS (Fig. 4b).

Two missense mutations in *RXFP2* (OAR10_29 461 968: E641K, OAR10_29 462 010: V627M) were characterized as most significantly differentiated among its protein-altering variants (**Supplementary Tables S23 and S24**). Both sites are highly conserved among vertebrate species and were mutated in PT and OL compared with other sheep (Fig. 4c). To confirm their haplotypic distributions among 10 breeds, we examined the genotypes of 1155 independent individuals at these 2 SNP sites. According to the result, the distributions of both SNPs were consistent with our WGS data, where the haplotype 1 consisting of “OAR10_29 461 968: T + OAR10_29 462 010: T” (*RXFP2*: M627 and K641) was mostly found in PT and OL, while the haplotype 2 consisting of “OAR10_29 461 968: C + OAR10_29 462 010: C” (*RXFP2*: V627 and E641) was predominant in VT, BY, MGS, and EUS (Fig. 4d, **Supplementary Fig. S8**).

We also compared the *RXFP2* haplotypes observed in our 99 sheep with those reported in the wild bighorn sheep population, where *RXFP2* was selected for intrasexual competitions [38]. The result showed no obvious similarity between these 2 haplotypes (**Supplementary Fig. S9 and S10**), suggesting different directions of adaptive evolution at *RXFP2* locus between the semi-feral TBS and the wild bighorn sheep.

Taken together, our results indicate that PT and OL sheep have formed a unique haplotype at *RXFP2* locus under the effect of positive selection.

### *RXFP2* haplotype controls horn size and shape

**Figure 5:**
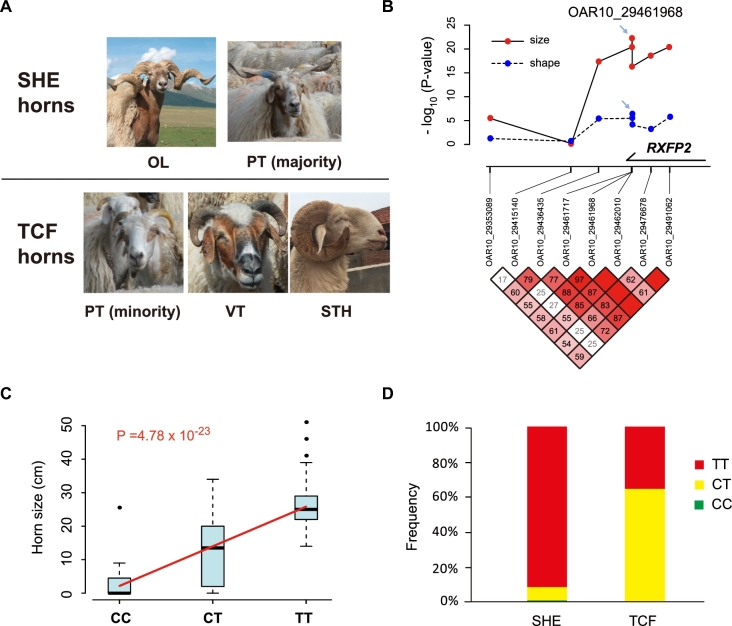
*RXFP2* haplotype is correlated with horn shape and size. a) Features of SHE-type and TCF-type horns. b) Association between 8 SNPs and horn phenotypes (size and shape) analyzed in 182 PT sheep. After testing all combinations of genetic models and confounding effects (**Supplementary Fig. S12**), an additive model (assume *A* as major allele, *a* as minor allele, we have code 2 for *AA*, 1 for *Aa*, and 0 for *aa*) was applied for horn size, and a recessive code (1 for *AA*, 0 for *Aa* and *aa*) was applied for horn shape; pair-wise LD between SNP pairs were plotted at the bottom, where numbers represent *D΄* statistics. c) Box plot of individual horn sizes among different OAR_29 461 968 genotypes; *P* value was calculated by linear regression based on additive genetic model, and the fitting line is shown in red. d) Distribution of OAR10_29 461 968 genotypes among PT sheep with different horn shapes.

A common feature of the semi-feral populations PT and OL is that they often have strong, long horns. These horns typically form a spiral and horizontal extension (SHE type) (Fig. 5a). The SHE horns are clearly different from the horns of European wild sheep (*Ovis orientalis*, *Ovis musimon*), which are regarded as the ancestor of modern domestic sheep in China [36] (**Supplementary Fig. S8**). In contrast, horns of VT, BY, MGS, and EUS are either polled or curled tightly close to the face (TCF type) (Fig. 5a). To determine whether *RXFP2* haplotype directly affected the appearance of horns, we tested their correlation in an independent PT population (n = 182) with heterogeneous horn types. This population consists of 138 SHE-type horned, 16 TCF-type horned, 14 scurred (small and undeveloped horns), 11 polled sheep, and 3 individuals with uncertain horn type (**Supplementary Fig. S11**, **Supplementary Table S29**). Regression models were applied to identify a potential association between horn phenotypes (horn size, horn shape) and 8 *RXFP2*-linked SNPs, which included 2 protein-altering, 2 intronic SNPs with high *F*_ST_ in semi-feral TBS, and 4 previously reported trait-associated SNPs in other sheep populations (**Supplementary Table S28**).

In the 182 PT sheep, we observed strong associations between horn sizes and 3 SNPs we identified based on *F*_ST_ (OAR10_29 461 968, OAR10_29 491 062, OAR10_29 461 717), while the 4 previously reported SNPs showed either minor or no effect (Fig. 5b). The highest correlation among all 8 SNPs was found at 1 of the protein-altering SNPs (OAR10_29 461 968), where each copy of T allele gave rise to about an 11.75-cm increase in horn length (*P* = 4.78 × 10^−23^) (Fig. 5c). By analyzing covariates in the regression model, we confirmed that this correlation was independent from individual age and sex, which potentially affected horn size (regarding covariates, *P* = 1.75 × 10^−27^) (**Supplementary Fig. S12**). Furthermore, we identified the same SNP OAR10_29 461 968, rather than previously reported SNPs, strongly correlated with horn shape (Fig. 5b). OAR10_29 461 968: T homozygotes were found to be overrepresented in SHE-type horned sheep relative to TCF-type individuals (*P* = 2.20 × 10^−7^) (Fig. 5d), which is in agreement with the shape distribution across different populations. These findings support the finding that the unique *RXFP2* haplotype we identified was responsible for the horn-phenotype differences between semi-feral and domestic populations in Chinese sheep.

### *RXFP2* gene expression in sheep horns

**Figure 6:**
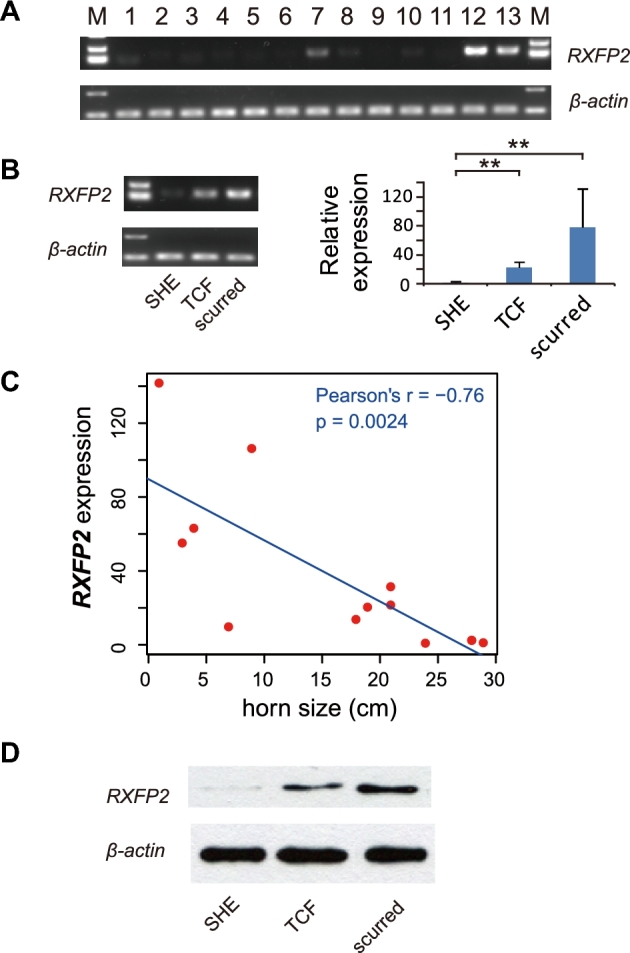
Gene expression patterns of *RXFP2*. a) Expression of *RXFP2* and *β-actin* in 13 tissue samples from PT sheep: 1, heart; 2, liver; 3, spleen; 4, lung; 5, kidney; 6, muscle; 7, brain; 8, ovary; 9, corpus uteri; 10, adipose; 11, thyroid; 12, soft horn; and 13, horn periosteum. b) Expression pattern of *RXFP2* in SHE-type, TCF-type, scurred soft-horn tissues examined by RT-PCR (left) and real-time PCR (right); error bars denote SD of the mean; groups with significant differences (*, *P* < 0.05; **, *P* < 0.001) are indicated. c) Scatter plot on *RXFP2* expression and horn size; the fitting line of linear regression is shown in blue. d) Western blot analysis of soft-horn tissues with different horn types, using antibodies of *RXFP2* and *β*-actin.

Although a solid association has been identified between the haplotype over the *RXFP2* locus and horn phenotypes, it is possible that causal variants might actually affect other flanking genes with respect to regulatory region alterations. To confirm the functional relevant gene for horns of TBS, we studied the expression patterns among different PT sheep tissues of all functional genes (*RXFP2*, *B3GLCT*, *FRY*, LOC101110773, LOC106991357) annotated within a approximately 1-Mb region encompassing *RXFP2*. The region was comprised of multiple LD blocks (**Supplementary Fig. S13**), which covered potential hitchhiking variants associated with the sweep. Interestingly, among all 5 studied genes, only *RXFP2* exhibited a pattern of tissue-specific expression in soft horn and horn periosteum (Fig. 6a, **Supplementary Fig. S14**). This tissue-expression pattern was also confirmed in samples from Sonid sheep (**Supplementary Fig. S15**).

Next, we compared the gene expression in the soft horn tissues of SHE-type, TCF-type, and scurred PT sheep. Despite obvious individual variations, a relatively lower expression of *RXFP2* was found in SHE than in TCF (*P* < 0.001) and scurred samples (*P* < 0.001) (Fig. 6b). Moreover, *RXFP2* expression was negatively correlated with horn size (Pearson *r* = −0.76, *P* = 0.002) (Fig. 6c). No obvious correlations between flanking gene expressions and horn phenotypes were observed (**Supplementary Fig. S16 and S17**). Furthermore, the *RXFP2* protein levels in soft horn tissues were examined using Western blot, which revealed a consistent reduction of translation product in SHE-type horns (Fig. 6d), which is in agreement with the mRNA expressions.

## Discussion

Feralization is the process where domestic animals go back to the wild. Although its reverse, domestication, has been studied extensively [1–4], the genetic basis of feralization remains largely unsolved. In this study, we performed a comprehensive survey of genetic diversity in Tibetan sheep. These animals are separated into various local breeds, within which a contrast of domestic and semi-feral status exists. Analysis of selective sweeps in 2 semi-feral populations compared with domestic lineages revealed a critical role of semi-feralization in development of male reproductive success, muscle strength, eating habits, aggressive behaviors, defensive responses, and sensory perception. Specifically, semi-feral sheep have developed a unique horn phenotype as plausible adaptation to the reduced human intervention and increasing natural and sexual selection.

Horns are crucial for the survival of wild sheep because male individuals with strong horns show advantages in competition to reproduce and aggressive horns are essential weapons against carnivorous enemies. In most domestic lines, horns become vestigial because traits that ensure fitness in natural life are becoming useless under artificial breeding. However, domesticated Tibetan sheep would be an exception, as we found a special SHE-type horn, mediated by the *RXFP2* gene, bringing about individual advantages in 2 semi-feral populations, PT and OL. First, PT and OL represented by their SHE-type horns, contained a special *RXFP2* haplotype with strong signals of selective sweeps compared with VT and MGS. Second, an association study within a PT population suggested that the *RXFP2* haplotype was significantly associated with horn size and shape. Third, gene expression analysis in 13 tissues of PT sheep demonstrated that *RXFP2* was the only gene specifically expressed in horn-related tissues and exhibiting decreased expression in SHE-type–horned individuals. Unlike in other pastoral areas, the breeding of Tibetan sheep is less affected by human intervention, and, most importantly, the mating is relatively random (e.g., opposite sexes are kept separately for most MGS, but not TBS) (**Supplementary Table S2**). PT and OL live in an area with sparse human populations (**Supplementary Fig. S7**), suggesting little artificial impact and more threats from the wild (e.g., more wolves were observed in sparsely populated regions of Tibet). Therefore, large and aggressive horns will bring about advantages for PT and OL, just as in wild and other feral populations [38].

An interesting phenomenon is that SHE-type horns (with a spiral and twisted shape) seem to also exist in other sheep breeds outside of China, such as the Hungarian Racka sheep (**Supplementary Fig. S18**). It is not clear whether there were genetic introgressions related with this phenotype from other ovine lineages into TBS, or vice versa. It is also possible that the *RXFP2* genotype related to SHE horns was newly derived in TBS. These possibilities can be tested in the future when genotypic data from more sheep breeds are generated.

*RXFP2* is a well-known genetic determinant of horn phenotype in sheep. This locus is correlated with quantitative and discrete traits of horns in wild and feral populations [37, 38, 55]. For domestic sheep, SNPs within or around *RXFP2* are predictive for polledness [56–58]. Although the contributions of *RXFP2* to horn phenotypes have been extensively studied [38, 59, 60], little is known about the mechanism that accounts for the various outcomes of sheep horns. Our study confirmed *RXFP2* to be the functional gene responsible for the special horn shape observed in semi-feral breeds PT and OL. The expression patterns of *RXFP2* among horn types, which we identified, provided novel evidence for the genetic basis underlying the growth of horns, as well as polledness. As in cattle, understanding polledness of sheep is crucial because it improves the welfare of animals and protects their handlers [61]. Additional efforts are required to clarify the role of *RXFP2* in development of diverse sheep horns.

## Conclusion

In conclusion, the present study revealed a Tibetan sheep subpopulation that has been subject to alterations in genomic loci related to its semi-feralization. These sheep have undergone rapid evolution across the *RXFP2* gene to acquire strong and weapons-grade horns, as a consequence of sexual selection and reduced human intervention. Our study highlights the importance of human activities in adaptive evolution of domestic animals and provides a novel insight into their processes of semi-feralization.

## Availability of supporting data

Raw sequence data have been submitted to the NCBI Sequence Read Archive under accession number SRP066883. Genotypic data of 99 individuals have been submitted to the European Variation Archive under accession number ERZ480291 (project PRJEB23437). Supporting data, including variant files (VCF), phylogenetic tree files, pictures of individual sheep, gel images, and perl scripts, are available via the *GigaScience* repository GigaDB [62].

## Additional file

**Supplementary Figure S1.** Venn diagram of the variant comparison between the present study and dbSNP. All validated SNPs and indels from dbSNP build 143 were compared with the 42 million variants characterized in the present study.

**Supplementary Figure S2.** Phylogeny tree of 99 sheep and outgroups. (**a**) Neighbor-joining tree of 99 sheep and two outgroup genomes (Texel sheep reference genome and goat genome). (**b**) A bootstrapping phylogeny tree constructed based on pruned genomic variants; number on the branch point indicate bootstrapping values.

**Supplementary Figure S3.** Population structure analysis assuming 2–10 ancestral lineage.

**Supplementary Figure S4.** Migration events estimated by TreeMix. Migration events were estimated by TreeMix using number *m* = 0–5. Table at bottom showed the migration weights for different *m*.

**Supplementary Figure S5.** Geographical locations of Tibetan sheep breeds. The location of Tibetan breeds were marked. Three lineages from the present study were showed in yellow dots (PT, Prairie Tibetan sheep; OL, Oula sheep; VT, Valley Tibetan sheep), and four lineages from another native sheep study (Yang et al. 2016) were showed in green (ZNQ, ZCD, ZLZ and ZRK).

**Supplementary Figure S6.** Distributions of the minor Tajima's *D* and linkage disequilibrium (LD) in 10 sheep breeds. (**a**) Tajima's *D* was calculated using a 50-kb sliding window across the genome. (**b**) The mean correlation coefficient (r^2^) between SNP pairs decayed with increasing pair-wise distance.

**Supplementary Figure S7.** Human population density in China. The human population density map is downloaded as picture from CHINA-MIKE website (http://www.china-mike.com/china-travel-tips/tourist-maps/china-population-maps/). Human population density around YarluZangbu River where presented PT and OL sheep (blue dashed circle) is higher than the region where presented VT sheep (red dashed circle).

**Supplementary Figure S8.** Genotyping using a SNaPshot screening assay of two differentiated SNPs in 1155 domestic sheep. The allele frequencies of two differentiated protein-altering SNPs in *RXFP2* genes are shown: *RXFP2-1*, G>A (E641K); *RXFP2-2*, G>A (V627M). The length of the bar denotes the proportion of individuals carrying the genotype according to color. For each SNP, the frequency was calculated from the 99 WGS dataset (left) and from the 1155 SNaPshot dataset as shown.

**Supplementary Figure S9.** Comparison between *RXFP2* haplotypes in Chinese sheep and the wild bighorn sheep. Genotype data of the bighorn sheep were downloaded from Dryad: doi:10.5061/dryad.3f2t2. Bar plots of allele frequency of 2575 biallelic SNPs at *RXFP2* locus (assembly oviAri3, chromosome 10: 29,400,000–29,600,000 bp) were generated to visualize the haplotypic similarity between breeds, where orange bars denote fraction of the reference allele (identical to the reference genome) and purple bars denote that of the alternative allele. (TET, Tetons; SR, Sun River; WB, Whiskey Basin).

**Supplementary Figure S10.** Genetic relationship between different sheep populations at *RXFP2* locus. Pair-wise genetic distance between breeds were calculated based on average allele frequency differences (average ΔAF) of 2575 SNPs at the *RXFP2* locus. Phylogeny tree was generated based on the distance matrix using Neighbor-joining algorithm.

**Supplementary Figure S11.** Features of different horn types in PT sheep.

**Supplementary Figure S12.** Association map of eight SNPs within or close to *RXFP2* and horn phenotypes. -log_10_(P value) of eight SNPs were showed under different regression models. Different genetic models were considered, including recessive (*AA*: 1, *Aa*: 0, *aa*: 0), additive (*AA*: 2, *Aa*: 1, *aa*: 0) and dominant models (*AA*: 1, *Aa*: 1, *aa*: 0). Missing points under dominant models were resulted from the absence of the homozygotes *aa*.

**Supplementary Figure S13.** LD plot of ∼1Mb region encompassing *RXFP2*. Pair-wise LD (*D*’) was calculated using Haploview for each SNP combination within the genomic region from chr10:28,900,000 bp to chr10:30,100,000 bp. Variants were filtered by minor allele frequency (MAF < 0.2 were excluded) to reduce computational complexity. Positions of five tested genes were showed according to NCBI annotations.

**Supplementary Figure S14.** Tissue expression profile of *RXFP2* and flanking genes in PT sheep. (**a**) Soft horn can be developed from horn periosteum, then after cornification, form hard horn. The soft horn and horn periosteum were collected from the root of horn. (**b**) RT-PCR result of 13 tissues: 1, heart; 2, liver; 3, spleen; 4, lung; 5, kidney; 6, muscle; 7, brain; 8, ovary; 9, corpus uteri; 10, adipose; 11, thyroid; 12, soft horn; 13, horn periosteum. (**c**) Quantitative real-time PCR result of *RXFP2* in the same 13 tissue samples.

**Supplementary Figure S15.** Tissue expression profile of *RXFP2* in Sonid sheep. 1, Heart; 2, liver; 3, spleen; 4, lung; 5, kidney; 6, muscle; 7, brain; 8, ovary; 9, corpus uteri;10, adipose; 11, thyroid; 12, soft horn; 13, horn periosteum; 14, cerebellum; 15, hypothalamus; 16, hypophysis; 17, intestine; 18, duodenum.

**Supplementary Figure. S16.** The expression level of *RXFP2* and flanking genes in PT soft-horn tissues with different horn types.

**Supplementary Figure S17.** Scatter plot of horn size and expressions of *RXFP2* and flanking genes. For each comparison, the Pearson's correlation coefficient *r* was calculated. *P* value and fitting lines were calculated using linear regressions.

**Supplementary Figure S18.** Horn features of other sheep breeds. Images of Racka sheep (https://en.wikipedia.org/wiki/Racka) and 4 different wild sheep were collected from internet: (1) Mouflon (https://en.wikipedia.org/wiki/Mouflon); (2) Urial (https://en.wikipedia.org/wiki/Urial); (3) Argali (https://en.wikipedia.org/wiki/Argali); and (4) Bighorn sheep (https://species.wikimedia.org/wiki/Ovis_canadensis).

**Supplementary Table S1.** Photographs of the 10 sheep breeds examined in the present study.

**Supplementary Table S2.** Breeds, origin, phenotypic characteristics and artificial factors of domestic sheep sequenced in the present study.

**Supplementary Table S3.** Number of reads and bases in quality control.

**Supplementary Table S4.** Read mapping statistics and coverage of depth.

**Supplementary Table S5.** Statistics for the 99 sequenced domestic sheep from 10 indigenous breeds.

**Supplementary Table S6.** Number of annotated SNPs in different gene regions.

**Supplementary Table S7.** Number of annotated indels in different gene regions.

**Supplementary Table S8.** Validation of SNP calling based on targeted sequencing.

**Supplementary Table S9.** Summary of false positive and false negative rate for SNP calling.

**Supplementary Table S10.** Selective sweep regions in PT sheep.

**Supplementary Table S11.** Selective sweep regions in OL sheep.

**Supplementary Table S12.** Selective sweep regions in VT sheep.

**Supplementary Table S13.** Selective sweep regions in BY sheep.

**Supplementary Table S14.** Summary of candidate genes in four populations.

**Supplementary Table S15.** Low diversity genomic regions excluded from LSBL analysis.

**Supplementary Table S16.** GO enrichment analysis of PT candidate genes.

**Supplementary Table S17.** GO enrichment analysis of OL candidate genes.

**Supplementary Table S18.** GO enrichment analysis of VT candidate genes.

**Supplementary Table S19.** GO enrichment analysis of BY candidate genes.

**Supplementary Table S20.** GO enrichment analysis of overlapping candidate genes between PT and OL (semi-feral group).

**Supplementary Table S21.** GO enrichment analysis of overlapping candidate genes among PT, OL and VT (Tibetan group).

**Supplementary Table S22.** Summary of candidate genes related with semi-feralization.

**Supplementary Table S23.** Protein altering mutations in PT candidate genes.

**Supplementary Table S24.** Protein altering mutations in OL candidate genes.

**Supplementary Table S25.** Protein altering mutations in VT candidate genes.

**Supplementary Table S26.** Protein altering mutations in BY candidate genes.

**Supplementary Table S27.** Primer sequences in the present study.

**Supplementary Table S28.** Information of SNPs genotyped in the 182 PT sheep.

**Supplementary Table S29.** Phenotypic information and SNP genotypes of 182 PT sheep.

## Abbreviations

AM, Australian Merino sheep; BY, Bayinbuluke sheep; CB, Cele Black sheep; EUS, European sheep; GO, gene ontology; H, Hu sheep; indel, insertion and deletion; LD, linkage disequilibrium; MGS, Mongolian sheep; NCBI: National Center for Biotechnology Information; OL, Oula sheep; PCA, principal component analysis; PCR: polymerase chain reaction; PT, Prairie Tibetan sheep; QC: quality control; SHE, spirally and horizontally extended; SNP, single nucleotide polymorphism; STH, Small Tail Han sheep; T, Tan sheep; TBS, Tibetan sheep; TCF, tightly close to the face; VT, Valley Tibetan sheep; WGS, whole-genome sequencing; WZ, Wuzhumuqin sheep.

## Ethic approval

All experimental procedures involving animals were approved by the Chinese Ministry of Agriculture and the animal care and use committee at the institution where the experiments were performed.

## Competing interests

The authors declare that they have no competing interests.

## Supplementary Material

GIGA-D-17-00165_Original_Submission.pdf

GIGA-D-17-00165_Revision_1.pdf

GIGA-D-17-00165_Revision_2.pdf

Response_to_Reviewer_Comments_Original_Submission.pdf

Response_to_Reviewer_Comments_Revision_1.pdf

Reviewer_1_Report_(Original_Submission) -- Kerstin Lindblad-Toh 18 Aug 2017 Reviewed

Reviewer_1_Report_(Original_Submission)_ReviewGIGA-17-00165.pdf

Reviewer_2_Report_(Original_Submission) -- Johannes Lenstra 22 Aug 2017 Reviewed

Reviewer_2_Report_(Revision_1) -- Johannes Lenstra 04 Dec 2017 Reviewed

Supplemental material
