# Supplementary material for: Whole-genome sequences of 89 Chinese sheep suggest role of RXFP2 in the development of unique horn phenotype as response to semi-feralization
Source: Gigascience. 2018 Mar 7;7(4):giy019. doi: 10.1093/gigascience/giy019 (PMC5905515; doi:10.1093/gigascience/giy019)
Supplement: GIGA-D-17-00165_Original_Submission.pdf [file giy019_giga-d-17-00165_original_submission.pdf]

GigaScience

Whole-genome sequencing of 99 sheep provides insight into animal semi-feralization

--Manuscript Draft--

**Manuscript Number:**	GIGA-D-17-00165
**Full Title:**	Whole-genome sequencing of 99 sheep provides insight into animal semi-feralization
**Article Type:**	Research
**Funding Information:**	Agricultural Science and Technology Innovation Program of China (ASTIP-IAS13) — Prof. Mingxing Chu<br>Earmarked Fund for China Agriculture Research System (CARS-39) — Prof. Mingxing Chu<br>National Key Technology Support Program (2013BAI101B09) — Prof. Yixue Li<br>National Natural Science Foundation of China (CN) (31472078) — Prof. Mingxing Chu<br>National Natural Science Foundation of China (31402041) — Dr. Qiuyue Liu<br>National Key Scientific Instrument and Equipment Development Project (2012YQ03026108) — Prof. Yixue Li<br>National Basic Research Program of China (2011CB910204) — Prof. Yixue Li<br>National Basic Research Program of China (2011CB510102) — Prof. Yixue Li<br>Youth Innovation Promotion Association of the Chinese Academy of Sciences (2017325) — Dr. Zhen Wang<br>Genetically Modified Organisms Breeding Major Program of China (2016ZX08009-003-006) — Dr. Qiuyue Liu<br>Genetically Modified Organisms Breeding Major Program of China (2016ZX08010-005-003) — Prof. Mingxing Chu<br>Major Science and Technology Program of Inner Mongolia Autonomous Region of China — Prof. Mingxing Chu
**Abstract:**	Background<br>Although animal domestication has been extensively studied, the process of their feralization remains poorly understood.<br>Results<br>Here, we performed whole-genome sequencing of 99 sheep and identified a primary genetic divergence between two heterogeneous population in Tibetan Plateau, including one semi-feral lineage. Selective sweep and candidate gene analysis revealed their local adaptations of these sheep associated with sensory perception, muscle strength, eating habbit, mating process and social behavior. In particular, a horn-related gene RXFP2 underlied rapid evolution in this semi-feral population specifically. A novel haplotype and repressed horn-related-tissue expressions of RXFP2 were correlated with higher horn length, as well as spiral and horizontally extended horn shape.<br>Conclusions<br>Semi-feralization has an extensive impact over diverse phenotypic traits of sheep. By acquiring features simliar with their wild ancestors, semi-feral sheep were able to re-gain fitness in frequent contact with wild and rare human intervention. The present study provides a new insight into the evolution of domestic animals when human interventions are no longer dominant.

Powered by Editorial Manager® and ProduXion Manager® from Aries Systems Corporation

**Corresponding Author:**	Shengdi Li<br><br>CHINA
**Corresponding Author Secondary Information:**	
**Corresponding Author's Institution:**	
**Corresponding Author's Secondary Institution:**	
**First Author:**	Zhangyuan Pan
**First Author Secondary Information:**	
**Order of Authors:**	Zhangyuan Pan
	Shengdi Li
	Qiuyue Liu
	Zhen Wang
	Zhengkui Zhou
	Ran Di
	Benpeng Miao
	Wenping Hu
	Xiangyu Wang
	Xiaoxiang Hu
	Ze Xu
	Dongkai Wei
	Xiaoyun He
	Liyun Yuan
	Xiaofei Guo
	Benmeng Liang
	Ruichao Wang
	Xiaoyu Li
	Xiaohan Cao
	Xinlong Dong
	Qing Xia
	Hongcai Shi
	Geng Hao
	Jean Yang
	Cuicheng Luosang
	Yiqiang Zhao
	Mei Jin
	Yingjie Zhang
	Shenjin Lv
	Fukuan Li
	Guohui Ding

	Mingxing Chu
	Yixue Li
**Order of Authors Secondary Information:**	
**Opposed Reviewers:**	
**Additional Information:**	
**Question**	**Response**
Are you submitting this manuscript to a special series or article collection?	No
**Experimental design and statistics**<br><br>Full details of the experimental design and statistical methods used should be given in the Methods section, as detailed in our Minimum Standards Reporting Checklist. Information essential to interpreting the data presented should be made available in the figure legends.<br><br>Have you included all the information requested in your manuscript?	Yes
**Resources**<br><br>A description of all resources used, including antibodies, cell lines, animals and software tools, with enough information to allow them to be uniquely identified, should be included in the Methods section. Authors are strongly encouraged to cite Research Resource Identifiers (RRIDs) for antibodies, model organisms and tools, where possible.<br><br>Have you included the information requested as detailed in our Minimum Standards Reporting Checklist?	Yes
**Availability of data and materials**<br><br>All datasets and code on which the conclusions of the paper rely must be either included in your submission or deposited in publicly available repositories (where available and ethically appropriate), referencing such data using a unique identifier in the references and in the "Availability of Data and Materials" section of your manuscript.<br><br>Have you have met the above requirement as detailed in our Minimum	Yes

Standards Reporting Checklist?	

Powered by Editorial Manager® and ProduXion Manager® from Aries Systems Corporation

Manuscript
Click here to download Manuscript Manuscript.doc

Whole-genome sequencing of 99 sheep provides insight into animal semi-feralization

Zhangyuan Pan[†,1,3], Shengdi Li[†,2,4], Qiuyue Liu[†,1], Zhen Wang[†,2], Zhengkui Zhou[1], Ran Di[1], Benpeng Miao[2,4], Wenping Hu[1], Xiangyu Wang[1], Xiaoxiang Hu[5], Ze Xu[6], Dongkai Wei[6], Xiaoyun He[1], Liyun Yuan[2], Xiaofei Guo[1], Benmeng Liang[1], Ruichao Wang[2], Xiaoyu Li[1], Xiaohan Cao[1], Xinlong Dong[1], Qing Xia[1], Hongcai Shi[7], Geng Hao[8], Jean Yang[9], Cuicheng Luosang[9], Yiqiang Zhao[5], Mei Jin[10], Yingjie Zhang[11], Shenjin Lv[3], Fukuan Li[3], Guohui Ding[2,12], Mingxing Chu*[,1] & Yixue Li*[,2,12]

[1]Institute of Animal Science, Chinese Academy of Agricultural Sciences, Beijing, China.

[2]Key Lab of Computational Biology, CAS-MPG Partner Institute for Computational Biology, Shanghai Institutes for Biological Sciences, Chinese Academy of Sciences, Shanghai, China.

[3]College of Agriculture and Forestry Science, Linyi University, Linyi, China

[4]University of Chinese Academy of Sciences, Beijing, China;

[5]State Key Laboratory for Agrobiotechnology, China Agricultural University, Beijing, China.

[6]BasePair BioTechonology Co., Ltd., Suzhou, China.

[7]Institute of Biotechnology, Xinjiang Academy of Animal Science, Urumqi, China.

[8]Institute of Animal Science, Xinjiang Academy of Animal Science, Urumqi, China.

[9]Research Institute of Animal Science, Tibet Academy of Agricultural and Animal Husbandry Sciences, Lhasa, China.

[10]College of Life Science, Liaoning Normal University, Dalian, China.

[11]College of Animal Science and Technology, Agricultural University of Hebei, Baoding, China.

[12]Shanghai Center for Bioinformation Techonology, Shanghai Industrial Technology Institute, Shanghai, China.

[†]These authors contributed equally to this work.

*These authors jointly directed this work.

Correspondence should be addressed to Y.L. (yxli@sibs.ac.cn) or M.C. (mxchu@263.net)

Abstract

Background

Although animal domestication has been extensively studied, the process of their feralization remains poorly understood.

Results

Here, we performed whole-genome sequencing of 99 sheep and identified a primary genetic divergence between two heterogeneous population in Tibetan Plateau, including one semi-feral lineage. Selective sweep and candidate gene analysis revealed their local adaptations of these sheep associated with sensory perception, muscle strength, eating habbit, mating process and social behavior. In particular, a horn-related gene *RXFP2* underlied rapid evolution in this semi-feral population specifically. A novel haplotype and repressed horn-related-tissue expressions of *RXFP2* were correlated with higher horn length, as well as spiral and horizontally extended horn shape.

Conclusions

Semi-feralization has an extensive impact over diverse phenotypic traits of sheep. By acquiring features simliar with their wild ancestors, semi-feral sheep were able to re-gain fitness in frequent contact with wild and rare human intervention. The present study provides a new insight into the evolution of domestic animals when human interventions are no longer dominant.

Key words

Domestic animal - Adaptive evolution - Artificial selection - Semi-feralization - Horn

Background

Animal domestication has been widely investigated to better understand the phenotypic and genetic changes of animals caused by human activities [1-4], however the process that domestic animals become feral is still poorly understood. If domestication is the process where human benefits take over the adaptations from natural forces, then feralization could be regarded as its reverse: the animals re-start to fit natural life when artificial selections were no longer dominant [5].

Domestic sheep are usually distinguished from their wild ancestor in terms of many morphological traits like their body and horn size [6]. The history of Chinese sheep domestication can be traced back to 8 ka according to archeological evidences [7, 8]. A recent mito-genomic evolution study proposed that the Chinese domestic sheep originated from Eurasian regions and were subsequently dispersed into China through the Mongolian Plateau [9]. After long-term localization in China, more than 70 indigenous breeds have been formed. Among them, two representative ancestral lineages have been identified: Mongolian sheep and Tibetan sheep [10]. The Mongolian lineage consists of the majority of sheep breeds from low-altitude areas of China, while Tibetan sheep were restrict to the Tibetan Plateau, a high-altitude area (>4,000 m above the sea) with harsh and hypoxic environment.

The unique domestication history and husbandry system of Tibetan sheep make it an appropriate evolutionary model for studying animal semi-feralization. First off, as Tibetan Plateau is rich with grassland, the local breeds, especially ones living in

prairies, were roaming with nomads and fed on natural ranch. Second, these sheep were forced to encounter threats from the wild (e.g. Tibetan wolves), because of sparsely populated and undeveloped environment. Third, unlike other pastoral areas of China, breeding of Tibetan sheep was not subject to intense artificial control, such as gender-separating management and selective breeding. In this case, the evolution of these semi-feral populations can provide indications how domestic animals adapt when artificial pressures are loosen.

To enhance the understanding in animal feralization, we sequenced and analyzed the genomes of 30 sheep from 2 semi-feral breeds and 1 domestic breed in Tibet Plateau and 69 domestic sheep from other geographic areas. We identified a primary divergence in Tibetan sheep and a set of candidate loci underlying selective sweep in each Tibetan sheep population, which is responsible for their distinct phenotypic patterns related with semi-feralization.

Data Description

We selected 30 sheep from 3 typical Tibetan breeds in Tibetan Plateau (PT, Prairie Tibetan sheep; VT, Valley Tibetan sheep; OL, Oula sheep), 59 sheep from 6 Mongolian breeds across other areas of China (BY, Bayinbuluke sheep; CB, Cele Black sheep; H, Hu sheep; T, Tan sheep; STH, Small Tail Han sheep; WZ, Wuzhumuqin sheep), as well as 10 Australian Merino sheep (AM) representing a European-originated breed (**Figure 1a**, **Supplementary Table S1-S2**). Among the 10

breeds, PT and OL were two semi-feral populations which did not receive extensive human interventions (**Supplementary Table S2**). The sex ratio was maintained at approximately 1:1 for each breed. We performed whole-genome sequencing (WGS) of the 99 sheep. The coverage depth after genome alignment was approximately 6-fold for each individual (**Supplementary Table S3-S4**), resulting in more than 50× coverage depth for each breed.

Analyses

Characterization of the variants

After applying stringent criteria of quality control, we identified a total of 38,090,348 SNPs and 4,348,493 insertions/deletions (indels) in the 99 genomes (**Supplementary Table S5**). The abundance of variants was comparable to those of other domestic animals[11-13]. Most variants were intergenic or intronic, and only 269,584 SNPs and 5,518 indels were exonic (**Supplementary Table S6-S7**). More than 94.0% of the variants (26,598,869 SNPs and Indels) in the dbSNP database (build 143) were captured in our dataset (**Supplementary Figure S1**). The genome-wide average diversity π of the sheep breeds was estimated to be $2.44\text{-}2.84 \times 10^{-3}$, which was similar as previous reported [14]. In other Chinese domestic animals, such as pigs and dogs, increased nucleotide diversity is typically detected in Tibetan breeds compared with their counterparts outside of Tibet [11, 12]. However, the Tibetan sheep breeds ($\pi = 2.44\text{-}2.61 \times 10^{-3}$, $\theta = 2.10\text{-}2.30 \times 10^{-3}$) exhibited lower

nucleotide diversity than the Mongolian (π = 2.69-2.79 × 10^{-3}, θ = 2.36-2.52 × 10^{-3}) and European breeds (π = 2.84 × 10^{-3}, θ = 2.50 × 10^{-3}), reflecting the unique migration history of domestic sheep from Mongolian Plateau to Tibet [9].

Population genetics of Chinese sheep

To understand the genetic relationships among these sheep, we constructed a neighbor-joining (NJ) tree based on their genetic distance matrix using whole-genome SNPs (**Figure 1b**). Meanwhile, we calculated a rooted tree regarding the genome of goat as the out-group (**Supplementary Figure S2a**). As expected, the European-originated sheep (AM and Texel) were the first clade separated from the ancestral lineage, which was followed by the Mongolian breeds and finally, the Tibetan breeds. This phylogeny structure is again consistent with the recent hypothesis on domestic sheep migration, which suggested that Eurasian sheep initially migrated into Mongolian Plateau and then spread into local areas of China [9]. The three Tibetan sheep breeds formed a monophyletic clade which was robust under bootstrapping test (**Supplementary Figure S2b**), indicating a common origin of Tibetan sheep from one recent ancestral lineage.

We next performed the principal component analysis (PCA) of 99 sheep based on their genomic variants (**Figure 1c**). In despite of the division among Tibetan sheep (TBS), Mongolian sheep (MGS) and European sheep (EUS), a considerable genetic difference was observed between two groups of Tibetan sheep: Tibetan sheep group

one (TBS1) consisted of 20 individuals from the semi-feral breeds PT and OL, while Tibetan sheep group two (TBS2) contained 10 individuals of the domestic breed VT (**Figure 1c**). We further examined the population structure by assuming the number of ancestry K (**Figure 1d, Supplementary Figure S3**). When K = 3, the TBS, MGS and EUS were fully separated, though BY, one breed of MGS, showed admixture between TBS and MGS. When K = 4, we observed a primary divergence between TBS1 and TBS2, in agreement with the PCA result. In addition, as TreeMix [15] also showed a clear migration event from TBS2 to BY (**Supplementary Figure S4**), we excluded this breed from further analysis of selective sweeps.

The TBS2 breed VT showed a quite unique breeding history, represented by their slow linkage disequilibrium (LD) decay and the most positive Tajima's *D* statistics over genome compared with other breeds (**Supplementary Figure S5**). These statistics suggest that TBS2 has encountered the most severe contraction of population size during localization. These sheep also showed lower genetic diversity ($\pi = 2.44 \times 10^{-3}$) than TBS1 ($\pi = 2.60\text{-}2.61 \times 10^{-3}$), MGS ($\pi = 2.69\text{-}2.79 \times 10^{-3}$) and EUS ($\pi = 2.84 \times 10^{-3}$). Moreover, we checked the LD, PCA and population structure results from the recent study of Chinese native sheep and found a similar Tibetan population ZLZ, which was geographically close to VT and showed distinct genetic background as well as slow LD decay [14]. Based on these observations, we hypothesized that TBS2 might have encountered a recent bottleneck: its current population was derived from relatively small number of founders from the common ancestor of Tibetan sheep.

Given these findings, we concluded that the semi-feral Tibetan sheep breeds PT and OL were genetically proximate, and were distinct from the domestic lineage TBS2. TBS1 were typically free-roaming, raised by nomads, while TBS2 were captive, intensively managed by local farmers for improving productions and efficiencies (**Supplementary Table S2**). Moreover, TBS1 live in under-developed regions of northeastern Tibet, where human population is sparse (**Supplementary Figure S6**), suggesting less influence from human and more threats from the wild (e.g. Tibetan wolves). The small effective population size of TBS2 might be partly resulted from artificial processes, because their breeding processes were potentially more influenced by human.

Selective sweeps in semi-feral and domestic Tibetan sheep

To identify candidate genes that potentially underlie positive selection in different TBS populations, especially in the semi-feral population TBS1, we performed a selective sweep analysis over the whole genome based on population differentiation (Fixation index F_{ST}) and loss of heterozygosity (heterozygosity $log_2[H_P$ ratio]) in TBS1 and TBS2 respectively, treating MGS as an out-group (see **Methods**). In total, we identified 276 TBS1-specific genes, 403 TBS2-specific genes, as well as 203 genes shared by both TBS populations (**Figure 2a-b, Supplementary Table S8-S11**).

Semi-feral animals have more contact with natural environment and intra-sexual competition than their domestic relatives, thus characteristics ensuring better survival

in wild will be favored. For example, they may have higher muscle strength, stronger sensory systems and better food seeking abilities. According to the analysis of selective sweeps in semi-feral population TBS1, we observed a predominant signal of positive selection at chromosome 10, which harbors a Relaxin/insulin-like family peptide receptor 2 (*RXFP2*) gene (**Figure 2a**). *RXFP2* is a well-known gene related with horns, the sexual weaponry of sheep. Previous study has demonstrated a trade-off between horn type and life span, acting on the genetic variation of *RXFP2* in feral Soay sheep [16]. Recent research also proved that the wild bighorn sheep has been subject to selective sweep at the same locus [17]. Hence, *RXFP2* as a genetic marker, combined with morphological alterations in sheep horns, is potentially an indicator of "wildness" for sheep population.

Moreover, in TBS1, a number of lineage-specific candidate genes located in the top fifty most significant sweep regions (**Figure 2a**, **Supplementary Table S8**) are functionally plausible for adaptation in the wild. These genes include: (1) *RXFP2* affecting development of sheep horns [17, 18], which serve as sexual weaponry in intra-male competition; (2) *MITF*, *MSRB3*, *SLC26A4* associated with hearing [19-26]; (3) *GPCPD1* associated with optesthesia [27]; (4) *SMNDC1*, *SOX6* involved in muscle development [28, 29]; and (6) *PRD-SPRRII* regulating rumen development [30]. Besides, positive selection signals of both TBS1 and TBS2 were observed at the olfactory gene cluster (*LOC101102421*, *LOC101104931*) (**Supplementary Table S10**), which potentially affected sense of smell and food seeking.

Then, we performed Gene Ontology (GO) enrichment analysis of the gene sets

underlying positive selection in TBS1 (TBS1-specific + shared) and TBS2 (TBS2-specific + shared) (**Supplementary Table S12-S13**). The results showed a number of overrepresented functional terms related with feralization process. For example, a set of related terms, including "aggressive behavior" (GO:0002118, P = 0.012), "maternal behavior" (GO:0042711, P = 0.016), "parental behavior" (GO:0060746, P = 0.016) and "social behavior" (GO:0035176, P = 0.025) were overrepresented in TBS1 candidate genes, comprising of *AVP*, *OXT*, *MTOR* and *PENK* genes (**Supplementary Table S12**). Candidates related with "eating behavior" (GO:0042755, P = 0.031) were identified, suggesting the adaptation to the change in feeding styles. Genes associated with "male sex differentiation" (GO:0046661, P = 0.014), "male gonad development" (GO:0008584, P = 0.015) and "development of primary male sexual characteristics" (GO:0046546, P = 0.015) were also enriched in TBS1 candidate genes. In particular, most of these terms also consist of genes underlying positive selection in both TBS1 and TBS2, in addition to TBS1-specific genes. This is probably because opposite sexes are usually separated in management of domestic sheep in China, except TBS1 and TBS2. Although TBS2 were intensitively managed by local farmers, the interventions on their mating processes are much smaller than in MGS, thus they have also received selective pressures over genetic alterations on sexual development.

On a different note, the adaptive evolution of domestic population TBS2 showed an correlation with hypoxia adaptation and neuron functions, evidenced by a number of related GO terms with significant p values ($P < 0.05$) (**Supplementary Table S13**).

These terms included “regulation of cellular response to oxidative stress” (GO:1900407), “regulation of neuron projection development” (GO:0010975) and “regulation of neuron apoptotic process” (GO:0043523) (**Supplementary Table S13**). In particular, similar functional categories were also identified in TBS1, such as “regulation of hydrogen peroxide metabolic process” (GO:0010310), “cellular response to oxygen level” (GO:0071453), “regulation of neuron projection development” (GO:0010975), “neuron death” (GO:0070997) and “neuron migration” (GO:0001764) (**Supplementary Table S12**). We reasoned that the rapid evolution of these candidate genes, related with hypoxia and neuron functions, were possibly resulted from environment adaptation, as both TBS1 and TBS2 lived in an high-altitude area of Tibetan Plateau with an average low temperature.

Given these evidences, we reasoned that semi-feralization has an extensive impact over the genetics, and hence the phenotypes of TBS1 population. The phenotypic traits potentially affected in this process include sensory perception, muscle strength, eating habbit, mating process and social behavior (**Figure 2c**). In addition, both semi-feral TBS1 and domestic TBS2 have evolved to counter the strict environment at Tibetan Plateau, including high altitude and low temperature.

A horn-related locus *RXFP2* underlies positive selection in semi-feral sheep

We next investigated and validated the most prodominant adaptive signature of semi-feralization in TBS1, at chromosome 10 spanning a 60-kb region of *RXFP2* gene

(**Figure 2a**). The sweep region exhibits excess of population differentiation (F_{ST} = 0.793) and a dramatic loss of heterozygosity ($H_{P|TBS1}/H_{P|MGS}$ = 0.424) in TBS1 compared with MGS (**Figure 3a, Supplementary Table S8**). However, neither of the signals were observed in TBS2 (F_{ST} = 0.063, $H_{P|TBS2}/H_{P|MGS}$ = 0.966). Single nucleotide polymorphisms (SNPs) located in the *RXFP2* gene region forms a unique haplotype in TBS1, which is largely different from those in TBS2, MGS and EUS (**Figure 3b**).

Two missense variants on *RXFP2* (OAR10_29461968: E641K, OAR10_29462010: V627M) were characterized as the most significantly differentiated variants among protein altering mutations within all TBS1-specific sweep regions (**Supplementary Table S14**). Both of the sites are highly conserved among vertebrate species and were mutated in TBS1 compared with other sheep (**Figure 3c**). To confirm their haplotypic distributions among 10 breeds, we examined the genotypes of 1155 independent individuals at these two SNP sites. According to the result, the distribution of both SNPs were consistent with our whole genome sequencing (WGS) data, where "OAR10_29461968:T + OAR10_29462010:T" (*RXFP2*: M627 and K641) were mostly found in TBS1 and "OAR10_29461968:C + OAR10_29462010:C" (*RXFP2*: V627 and E641) were predominant in TBS2, MGS and EUS (**Figure 3d**, **Supplementary Figure S8S7**).

Taken together, our results indicated that TBS1 sheep have formed a unique haplotype at *RXFP2* locus under the effect of positive selection.

RXFP2 haplotype controls horn size and shape

Individuals from the semi-feral population TBS1, which did not receive intense artificial selections, usually have strong and long horns. Their horns typically formed a spiral and horizontal extension (SHE-type) (**Figure 4a**). The SHE horn shape has a different look from that of the European wild sheep (*Ovis orientalis*, *Ovis musimon*), which are regarded as the ancestor of Chinese domestic sheep [9], although they both are strong and aggressive (**Supplementary Figure S8**). In contrast, horns of TBS2, MGS and EUS are either polled or curled tightly close to face (TCF-type) (**Figure 4a**). To figure out whether *RXFP2* haplotype directly affected the appearance of horns, we further tested their correlation in an independent TBS1 population (PT sheep, n = 182) with heterogeneous horn types. This population consist of 138 SHE-type horned, 16 TCF-type horned, 14 scurred (small and undeveloped horns), 11 polled sheep, as well as 3 individuals with uncertain horn type (**Supplementary Figure S9**, **Supplementary Table S17**). Regression models were applied to identify potential association between horn phenotypes (horn size, horn shape) and 8 SNPs within or close to *RXFP2*, which included the two protein-altering, two intronic SNPs with high F_{ST} in TBS1 and 4 previously reported trait-associated SNPs in other sheep (**Supplementary Table S18**).

In the 182 TBS1 sheep, we observed strong associations between horn sizes and 3 SNPs we identified based on F_{ST} (OAR10_29461968, OAR10_29491062,

OAR10_29461717), while the 4 previously reported SNPs showed either minor or no effect (**Figure 4b**). The highest correlation among all 8 SNPs was found at one of the protein-altering SNPs (OAR10_29461968), where each copy of T allele gave rise to ~11.75 cm increase in horn length (P = 4.78×10^{-23}) (**Figure 4c**). By analyzing covariates in the regression model, we confirmed that this correlation was independent from individual age and sex, which potentially affected horn size (regarding covariates, P = 1.75×10^{-27}) (**Supplementary Figure S10**). Furthermore, we identified the same SNP OAR10_29461968, rather than previously reported SNPs, strongly correlated with horn shape (**Figure 4d**). OAR10_29461968:T homozygotes were found to be overrepresented in SHE-type horned sheep relative to TCF-type individuals (P = 2.20×10^{-7}) (**Figure 4e**), in line with the observations across different populations. These findings supported that the novel *RXFP2* haplotype we identified was responsible for the horn-phenotype differences among Tibetan sheep.

***RXFP2* gene expression in sheep horns**

Although a solid association has been identified between the haplotype over *RXFP2* locus and horn phenotypes, it is possible that causal variants might actually affect other flanking genes in respect of regulatory region alterations. To determine the functional relevant gene for horns of TBS, we studied the expression patterns among different PT sheep tissues of all annotated genes (*RXFP2*, *B3GLCT*, *FRY*, LOC101110773, LOC106991379), after excluding pseudo genes, within a 900-kb

region encompassing *RXFP2*. Interestingly, among all 5 studied genes, only *RXFP2* exhibited a pattern of tissue-specific expression in soft horn and horn periosteum (**Figure 5a, Supplementary Figure S11**). This tissue-expression pattern was also confirmed in samples from Sonid sheep (**Supplementary Figure S12**).

We next compared their gene expressions in the soft-horn tissues of SHE-type, TCF-type and scurred PT sheep. In despite of obvious individual variations, a relatively lower expression of *RXFP2* was found in SHE than in TCF ($P < 0.001$) and scurred samples ($P < 0.001$) (**Figure 5b**). Moreover, *RXFP2* expression was negatively correlated with horn size (Pearson's $r = -0.76$, $P = 0.002$) (**Figure 5c**). No obvious correlations between flanking gene expressions and horn phenotypes were observed (**Supplementary Figure S13-S14**). Furthermore, the *RXFP2* protein levels in soft-horn tissues were studied by western blotting, which revealed a similar reduction of its translation product in SHE-type horns (**Figure 5d**), consistent with the mRNA expressions.

Based on these findings, we preliminarily confirmed *RXFP2* as a functional gene involved in mediating horn phenotypes of TBS. Therefore, the strong selective sweep over *RXFP2* in semi-feral population TBS1 indicated a remarkable fitness advantage for individuals with long, strong and weaponary horns. Although some physical traits, such as aggressive horns, become vestigial during animal domestication because they are useless to human, our study suggested that domestic animals might re-acquired similar features as their wild ancestors under semi-feralization.

Discussion

Feralization is the process where domestic animals go wild. Although its reverse – domestication – has been extensively studied [1-4], the genetic basis of feralization remains largely unsolved. In the present study, we reported a concrete example of animal semi-feralization in sheep. The semi-feral Tibetan sheep are managed by human, but have experienced reduction of artificial intervention after their domestic ancestors have migrated to Tibetan Plateau. Their local adaptations could reflect the response of animal genomes to the shift over the artificial- and natural-selection balance. In particular, analysis of selective sweep in a semi-feral population compared with domestic lineages has revealed diverse mechanisms of semi-feralization, including genetic alterations in sensory perception, muscle strength, eating habbit, mating process and social behavior. Interestingly, the most predominant genomic signature underlying their semi-feralization process, which we identified, was represented by their remarkable physical trait: horns.

Horns are crucial for the survival of wild sheep because (1) male individuals with strong horns show advantages in competitions of reproductive resources; (2) aggressive horns are essential weapons against carnivorous enemies. In most domestic lines, horns become vestigial, because traits ensuring fitness in wild life are getting useless under artificial breeding. However, domesticated Tibetan sheep would be an exception, as we found a special SHE-type horn, mediated by *RXFP2* gene, bringing

about individual advantages in the semi-feral population TBS1. First off, TBS1 represented by their SHE-type horns, contained a special *RXFP2* haplotype with strong signal of selective sweeps compared with TBS2 and other sheep. Second, association study within TBS1 suggested that the *RXFP2* haplotype was significantly associated with horn size and shape. Third, gene expression analysis demonstrated that *RXFP2* was the only gene specifically expressed in horn-related tissues of TBS1 and exhibiting decreased expression in SHE-type horned sheep.

Unlike other pastoral areas, breeding of Tibetan sheep is less affected by human intervention, and most importantly, their mating is relatively random (e.g. opposite sexes are kept separately for most MGS, but not TBS) (**Supplementary Table S2**). TBS1 live in an area with sparse human populations (**Supplementary Figure S6**), suggesting little artificial impact and more threats from the wild (e.g. more wolves were observed in sparsely populated regions of Tibet). Therefore, large and aggressive horns will bring about advantages for TBS1, just as in other wild and feral populations [17]. After domestication, these animals were again constrained by natural and sexual selections, when artificial forces no longer dominated their evolution. However, the morphology of SHE-type horns was quite different from their wild ancestors, though they both looked strong and aggressive (**Supplementary Figure S8**), suggesting that these domestic animals adapted to naturalistic life by regaining similar advantageous traits instead of evolving back to their ancestral state.

RXFP2 was a well-known genetic determinant of horn phenotype in sheep. This locus was shown to be correlated with quantitative and discrete traits of horns in wild

and feral populations [16, 17, 31]. For domestic sheep, SNPs within or around *RXFP2* were predictive for polledness [32-34]. Although the contribtion of *RXFP2* to horn phenotypes have been extensively studied [17, 18, 35], little is known about the mechanism accounting for the various outcomes of sheep horns. Our study confirmed that *RXFP2* was the functional gene responsible for the special horn shape observed in TBS1. The expression patterns of *RXFP2* among horn types, which we identified, provided novel evidences to understand the genetic basis underlying growth of horns, as well as polledness. As in cattle, understanding polledness of sheep are crucial because it improves the welfare of animals and protects their handlers [36]. Further efforts are required to clarify the role of *RXFP2* in the development of diverse sheep horns.

In conclusion, the present study revealed a Tibetan sheep sub-population has been subject to alterations in genomic loci related with their semi-feralization. These sheep have undergone rapid evolution over *RXFP2* gene to acquire strong and weaponary horns, as a consequence of sexual selection and reduced human intervention. Our study highlighted the importance of human activities on adaptive evolutions of domestic animals and provided a novel insight into their semi-feralization.

Methods

Sample Collection and Sequencing

A total of 89 Chinese sheep from 9 diverse breeds, as well as 10 Australian Merino sheep from Australia were sequenced in the present study (**Supplementary Table S1-S2**). For each sheep, genomic DNA was extracted from 200 µl of peripheral venous blood using the QIAamp DNA Blood Mini Kit (Qiagen, Germany). The quality and integrity of the DNA were assessed using the A260/280 ratio and agarose gel electrophoresis. For sequencing library preparation, the genomic DNA was sheared to fragments of 300-400 bp and subsequently end-repaired, 'A'-tailed and ligated to Illumina sequencing adapters. The ligated products with sizes of 400-500 bp were selected on 2% agarose gels and subsequently amplified by ligation mediated PCR (LM-PCR). The libraries were sequenced on an Illumina HiSeq 2500 sequencer in 2 × 100 bp paired-end mode and controlled using Illumina HiSeq Control Software.

Variant Calling

The raw reads were processed using two steps of quality control (QC): (1) reads with adapter contamination were removed; and (2) reads with more than 10% ambiguous bases were excluded. Only paired reads were preserved after QC. The filtered reads were subsequently mapped to the sheep reference genome assembly oviAri3 (ftp://ftp.ncbi.nlm.nih.gov/genomes/all/GCA_000298735.1_Oar_v3.1/GCA_0002987

35.1_Oar_v3.1_genomic.fna.gz) using BWA [37] (version 0.7.12) for all individuals separately. PCR duplicates were removed using PICARD (available at http://broadinstitute.github.io/picard/, version 1.135). Indels were realigned using GATK [38] (version 3.2-2). SNPs and indels were called using SAMtools [37] (version 1.2) after pooling samples from the same breed. After SNP calling, the variants were filtered using vcfutil.pl varFilter, with a "-d 20 -D 100" parameter to remove low-quality SNPs and indels. After filtering, the variants were annotated using snpEff [39] (version 4.0e) according to the NCBI annotation (ftp://ftp.ncbi.nlm.nih.gov/genomes/Ovis_aries/GFF/ref_Oar_v3.1_scaffolds.gff3.gz).

Population Genetics Analysis

Pair-wise genetic distances were measured by the number of allele differences for genomic SNP sites. The neighbor-joining tree was calculated based on the distance matrix using PHYLIP [40] (version 3.69). To place a root for the phylogeny tree, we aligned the goat genome sequences (ftp://ftp.ncbi.nlm.nih.gov/genomes/all/GCA_000317765.1_CHIR_1.0/GCA_000317765.1_CHIR_1.0_genomic.fna.gz) with the sheep reference genome sequences using LASTZ [41] (version 1.02) and used the homologous site in goats to determine the ancestral allele for each SNP. Only biallelic autosomal SNPs were used to calculate the distance matrix. PCA was performed using EIGENSOFT [42, 43] (version 6.0.1), and population structures were inferred using FRAPPE software [44] (version 1.1).

Both the PCA and population structures were calculated based on autosomal SNPs after removing highly correlated SNP pairs using PLINK [45] (version 1.07) with the "-indep-pairwise 50 5 0.2" parameter. Migration events among sheep breeds were estimated using TreeMix [15] with migration number m = 0-5. Statistics including π (pair-wise nucleotide differences), θ (number of segregating sites), SNP densities and Tajima's D, were calculated using VCFtools [46] (v0.1.12b). The linkage disequilibrium r^2 was calculated using Haploview [47] based on 500,000 SNPs randomly selected from the genome. The parameters were set as "--missingCutoff 0.2 --dprime --minMAF 0.1". The SNP pairs were grouped according to the physical distances of these genes. The mean r^2 was adopted to represent the average LD for each group (e.g., 0~1 kb).

Selective Sweep Analysis

We applied a specified approach to detect selective sweeps that were lineage-specific (TBS1-specific, TBS2-specific) or shared by all populations in TBS, regarding population differentiation F_{ST} and heterozygosity $\log_2(H_P$ ratio) over a 30-kb sliding window with a step of 15 kb. F_{ST} distances between population were calculated using the Bio::PopGen::PopStats package in BioPerl [48]. The pooled heterozygosity H_P for population was calculated using the formula $H= 2\sum p\sum q/(\sum p+\sum q)^2$, where $\sum p$ represents the sum of the major allele frequencies of all SNP sites in the window and $\sum q$ represents sum of the minor allele frequencies [13].

The $\log_2(H_P$ ratio) between population A and B was calculated as $\log_2(H_{P|B}/H_{P|A})$, which reflected the loss of heterozygosity in A relative to B. 46 windows were excluded because of extremely small variant numbers (< 50 variants). We considered the windows with top 5% values as the significance threshold for single statistic (e.g. $F_{ST|A\ vs.\ B} > F_{ST|5\%}$, where $F_{ST|5\%}$ denotes the top 5% threshold of $F_{ST|A\ vs.\ B}$).

Briefly, shared selective sweeps were determined if the window had: ($F_{ST|TBS1\ vs.\ MGS} > F_{ST|5\%}$) & ($F_{ST|TBS2\ vs.\ MGS} > F_{ST|5\%}$) & ($\log_2[H_P\ ratio]_{TBS1+TBS2\ vs.\ MGS} > \log_2[H_P\ ratio]_{5\%}$); TBS1 lineage-specific sweeps were determined as: ($F_{ST|TBS1\ vs.\ MGS} > F_{ST|5\%}$) & ($F_{ST|TBS2\ vs.\ MGS} \leq F_{ST|5\%}$) & ($F_{ST|TBS1\ vs.\ TBS2} > F_{ST|5\%}$) & ($\log_2[H_P\ ratio]_{TBS1\ vs.\ MGS} > \log_2[H_P\ ratio]_{5\%}$); TBS2-specific sweeps were identified base on: ($F_{ST|TBS1\ vs.\ MGS} \leq F_{ST|5\%}$) & ($F_{ST|TBS2\ vs.\ MGS} > F_{ST|5\%}$) & ($F_{ST|TBS1\ vs.\ TBS2} > F_{ST|5\%}$) & ($\log_2[H_P\ ratio]_{TBS2\ vs.\ MGS} > \log_2[H_P\ ratio]_{5\%}$). A presentation of F_{ST} distribution over selective sweeps from three categories was showed in **Figure 2b**.

All annotated genes overlapped with sweep windows or their flanking windows (15-kb up- and down-stream the sweep region) were defined as candidate genes. Note that signals of 33 genes were detected as controversial because they were associated with multiple 30-kb windows and these windows were categorized differently (e.g. 6 *MITF* windows were detected as TBS1-specific and 1 was shared). 18 of the 33 controversial genes were re-categorized as lineage-specific or shared signals based on majority of its associated windows (**Supplementary Table S11**). The rest 14 genes were excluded from the candidate gene list. Furthermore, We estimated the cross-population extended haplotype homozygosity (XP-EHH) [49] pairwisely among

TBS1, TBS2 and MGS for candidate gene, based on haplotype data phased by fastPHASE [50]. GO functional enrichment analysis of the candidate genes was performed using ClueGO [51], in which the *P* values were corrected using the Benjamini-Hochberg approach (**Supplementary Table S12-14**). Protein-altering mutations were extracted from selective sweep windows to identify potential functional variants (**Supplementary Table S14-16**).

Validation of SNP Genotypes in Large Population

We collected 1155 additional venous jugular blood samples from sheep of 10 different breeds, including 100 AM sheep, 100 PT sheep, 98 VT sheep, 87 OL sheep, 100 BY sheep, 80 CB sheep, 100 H sheep, 100 T sheep, 100 WZ sheep and 290 STH sheep. Genomic DNA was extracted from whole blood using the phenol-chloroform method and dissolved in TE buffer (10 mM Tris-HCl [pH 8.0] and 1 mM EDTA [pH 8.0]). To validate the allele frequency of the two differentiated protein altering SNPs of *RXFP2* (**Supplementary Table S14**), we performed a multiplex screening assay (SNaPshot) [52] on these 1155 individuals. We designed amplification and SNaPshot Single-base extension primers **(Supplementary Table S19)**. Genotyping was performed using the SNaPshot™ Multiplex Kit (ABI) according to the manufacturer's instructions and analyzed using the ABI Genetic Analyzer 3730XL.

Association Study Between the *RXFP2* Genotype and Horn Phenotypes

9 SNPs within or near *RXFP2* locus (**Supplementary Table S18**) were genotype for 182 PT sheep with 5 horn types: polled (0 cm), scurred (0-12 cm), TCF-type (>12 cm, tightly close to face), SHE-type (>12 cm, spiral and horizontally extended) and uncertain-type (>12 cm, uncertain shape) (**Supplementary Table S17**). 1 out of the 9 SNPs were ignored because no variations were observed among these sheep. Correlations between SNP genotypes and horn phenotypes (horn size, horn shape) were estimated using linear or logistic regressions (performed with in-house R language scripts), depending on the variable type of outcome. Three different genetic models (recessive, additive and dominant) were applied for each pairs of association test. Confounding effect of individual age and sex were also tested by considering them as covariates in the model.

Gene Expression Analysis of *RXFP2* and its Flanking Genes

Tissue expression profiles of 5 genes located within the 900K region encompassing *RXFP2* locus, including *RXFP2*, *B3GLCT*, *FRY*, LOC101110773 (*EF1A1L*) and LOC106991357 (ncRNA) were examined by RT-PCR (**Supplementary Figure S11-S12**). Primer sequences were shown in **Supplementary Table S19**. We studied 13 tissues of TBS and 21 tissues of Sonid sheep. For each tissue type, equal volume of cDNA from 6 individuals (2 individuals from each horn type) were mixed as pooled cDNA sample. RT-PCR reactions were carried out in 50 μl volume including Taq DNA polymerase(5U/μl) (TaKaRa, Dalian, China) 0.25 μl,

10×PCR Buffer(+$MgCl_2$) 5μl, 10mM dNTPs (2.5mM each) 4μl , each primer (10 μM) 1 μl, cDNA 1μl, ddH_2O 37.75 μl. Amplification conditions were set as: initial denaturation at 95°C for 5 min, followed by 33 cycles of denaturation at 95°C for 30 s, annealing for 20 s at appropriate temperatures, extension at 72°C for 10 s; with a final extension at 72°C for 2 min on Mastercycler 5333 (Eppendorf AG, Hamburg, Germany). The PCR product mixture with 5μl loading buffer (6×), and loaded 5μl into 1% sepharose gel. After 15min 180mA electrophoresis, take a picture under Biorad GelDoc XR System (Bio-rad, USA).

Expressions of the 5 genes were measured by real-time PCR in 13 PT sheep soft-horn samples with different horn types. 4-5 biological replicates were selected from different individuals of same horn type (4 SHE-type, 4 TCF-type, and 5 scurred). For all 5 genes and internal control, real-time PCR was performed three times in one sample as technical replicates, and the average gene expressions of three replicates were calculated. Real-time PCR amplification was performed in a 20-μl of reaction mixture containing 2 μl of cDNA, 0.4 μl of each forward and reverse primer (10 μM), 0.4 μl of ROX Reference Dye II (50×), 10 μl of SYBR Green Real-time PCR Master Mix (2×), and 6.8 μl of ddH_2O. The reaction without template was treated as blank control. PCR amplification was performed in triplicate wells using the following conditions: 95ºC for 30 s, followed by 40 cycles of 95ºC for 5 s and 60ºC for 34 s. The melting curve was analyzed after amplification. The peak Tm on the dissociation curve was used to determine the specificity of PCR amplification. Standard curves of these genes were also constructed. *β*-actin expressions were used as internal control

among samples. Relative expression levels of 5 genes were calculated based on the expression of *RXFP2* in the SHE-type soft horn (its expression was defined as 1.0). The $2^{-\Delta\Delta Ct}$ method was used to process the real-time PCR results (Livak and Schmittgen, 2001).

Protein extracts from soft-horn tissues were prepared by complete homogenization of tissues in immunoprecipitation buffer (Beyotime, CA) according to the manufacturer's instructions. Equal amounts of protein extracts were mixed with sample buffer and then separated on 10% SDS-PAGE gels (60 μg/lane). Details of the western blotting process were as described previously [53]. Rabbit Anti-GPR106 antibody (BIOSS, Beijing, China), polyclonal rabbit anti-mouse *β*-actin antibody (Abcam, US) and Goa Anti-Rabbit IgG, HRP (Santa Clara, CA, USA) were used.

Availability of supporting data

All sequencing data from this study have been submitted to the NCBI Sequence Read Archive (SRA; http://www.ncbi.nlm.nih.gov/sra) under accession number SRP0066883.

Declarations

List of abbreviations

TBS, Tibetan sheep; MGS, Mongolian sheep; EUS, European sheep; PT, Prairie Tibetan sheep; VT, Valley Tibetan sheep; OL, Oula sheep; BY, Bayinbuluke sheep; CB, Cele Black sheep; H, Hu sheep; T, Tan sheep; STH, Small Tail Han sheep; WZ, Wuzhumuqin sheep; AM, Australian Merino sheep; WGS, whole-genome sequencing; indel, insertion and deletion; NJ, neighbor-joining; PCA, principal component analysis; LD, linkage disequilibrium; GO, gene ontology; SNP, single nucleotide polymorphism; SHE, spirally and horizontally extended; TCF, tightly close to face.

Consent for publication

Not applicable.

Ethic approval

All experimental procedures involving animals were approved by the Chinese Ministry of Agriculture, the animal care and use committee at the institution where the experiments were performed.

Competing interests

The authors declared no competing interests.

Fundings

This work was supported by the Agricultural Science and Technology Innovation Program of China (ASTIP-IAS13), the Earmarked Fund for China Agriculture Research System (CARS-39), the National Key Technology Support Program (2013BAI101B09), the National Natural Science Foundation of China (31472078 and 31402041), the National Key Scientific Instrument and Equipment Development Project (2012YQ03026108), the National Basic Research Program of China (2011CB910204, 2011CB510102), the Youth Innovation Promotion Association CAS (2017325) and the Genetically Modified Organisms Breeding Major Program of China (2016ZX08009-003-006 and 2016ZX08010-005-003), by Major Science and Technology Program of Inner Mongolia Autonomous Region of China.

Acknowledgements

The authors thank Ori-Gene Technology Co., Ltd. Beijing, China, for their contributions in sample preparations.

References

1. Rubin CJ, Zody MC, Eriksson J, Meadows JRS, Sherwood E, Webster MT, et al. Whole-genome resequencing reveals loci under selection during chicken domestication. Nature. 2010;464 7288:587-U145.

2. Axelsson E, Ratnakumar A, Arendt ML, Maqbool K, Webster MT, Perloski M, et al. The genomic signature of dog domestication reveals adaptation to a starch-rich diet. Nature. 2013;495 7441:360-4.

3. Carneiro M, Rubin CJ, Di Palma F, Albert FW, Alfoldi J, Barrio AM, et al. Rabbit genome analysis reveals a polygenic basis for phenotypic change during domestication. Science. 2014;345 6200:1074-9.

4. Frantz LAF, Schraiber JG, Madsen O, Megens HJ, Cagan A, Bosse M, et al. Evidence of long-term gene flow and selection during domestication from analyses of Eurasian wild and domestic pig genomes. Nat Genet. 2015;47 10:1141-+.

5. Callaway E. When Chickens Go Wild. Nature. 2016;529 7586:270-3.

6. Zohary D, Tchernov E and Horwitz LK. The role of unconscious selection in the domestication of sheep and goats. J Zool. 1998;245:129-35.

7. Chen FH, Dong GH, Zhang DJ, Liu XY, Jia X, An CB, et al. Agriculture facilitated permanent human occupation of the Tibetan Plateau after 3600 B.P. Science. 2015;347 6219:248-50. doi:10.1126/science.1259172.

8. Yang X, Scuderi LA, Wang X, Scuderi LJ, Zhang D, Li H, et al. Groundwater sapping as the cause of irreversible desertification of Hunshandake Sandy Lands, Inner Mongolia, northern China. Proc Natl Acad Sci U S A. 2015;112 3:702-6. doi:10.1073/pnas.1418090112.

9. Lv FH, Peng WF, Yang J, Zhao YX, Li WR, Liu MJ, et al. Mitogenomic meta-analysis identifies two phases of migration in the history of eastern eurasian sheep. Molecular Biology And Evolution. 2015;32 10:2515-33. doi:10.1093/molbev/msv139.

10. Tu YR. The Sheep and Goat Breeds in China. Shanghai Science and Technology Press.

1989:p. 6-19.

11. Ai H, Fang X, Yang B, Huang Z, Chen H, Mao L, et al. Adaptation and possible ancient interspecies introgression in pigs identified by whole-genome sequencing. Nat Genet. 2015;47 3:217-25. doi:10.1038/ng.3199.

12. Gou X, Wang Z, Li N, Qiu F, Xu Z, Yan D, et al. Whole-genome sequencing of six dog breeds from continuous altitudes reveals adaptation to high-altitude hypoxia. Genome Research. 2014;24 8:1308-15. doi:10.1101/gr.171876.113.

13. Rubin CJ, Zody MC, Eriksson J, Meadows JR, Sherwood E, Webster MT, et al. Whole-genome resequencing reveals loci under selection during chicken domestication. Nature. 2010;464 7288:587-91. doi:10.1038/nature08832.

14. Yang J, Li WR, Lv FH, He SG, Tian SL, Peng WF, et al. Whole-genome sequencing of native sheep provides insights into rapid adaptations to extreme environments. Molecular Biology and Evolution. 2016;33:2576-92. doi:10.1093/molbev/msw129.

15. Pickrell JK and Pritchard JK. Inference of population splits and mixtures from genome-wide allele frequency data. PloS Genetics. 2012;8 11:e1002967. doi:10.1371/journal.pgen.1002967.

16. Johnston SE, Gratten J, Berenos C, Pilkington JG, Clutton-Brock TH, Pemberton JM, et al. Life history trade-offs at a single locus maintain sexually selected genetic variation. Nature. 2013;502 7469:93-5. doi:10.1038/nature12489.

17. Kardos M, Luikart G, Bunch R, Dewey S, Edwards W, McWilliam S, et al. Whole-genome resequencing uncovers molecular signatures of natural and sexual selection in wild bighorn sheep. Mol Ecol. 2015;24 22:5616-32. doi:10.1111/mec.13415.

18. Kijas JW, Lenstra JA, Hayes B, Boitard S, Porto Neto LR, San Cristobal M, et al. Genome-wide analysis of the world's sheep breeds reveals high levels of historic mixture and strong recent selection. PLoS Biol. 2012;10 2:e1001258. doi:10.1371/journal.pbio.1001258.

19. Markakis MN, Soedring VE, Dantzer V, Christensen K and Anistoroaei R. Association of MITF gene with hearing and pigmentation phenotype in Hedlund white American mink (Neovison vison). Journal Of Genetics. 2014;93 2:477-81.

20. Chen L, Guo W, Ren L, Yang M, Zhao Y, Guo Z, et al. A de novo silencer causes elimination of MITF-M expression and profound hearing loss in pigs. BMC Biol. 2016;14:52. doi:10.1186/s12915-016-0273-2.

21. Tsukamoto K, Suzuki H, Harada D, Namba A, Abe S and Usami S. Distribution and frequencies of PDS (SLC26A4) mutations in Pendred syndrome and nonsyndromic hearing loss associated with enlarged vestibular aqueduct: a unique spectrum of mutations in Japanese. European Journal Of Human Genetics. 2003;11 12:916-22. doi:10.1038/sj.ejhg.5201073.

22. Shen X, Liu F, Wang Y, Wang H, Ma J, Xia W, et al. Down-regulation of msrb3 and destruction of normal auditory system development through hair cell apoptosis in zebrafish. International Journal Of Developmental Biology. 2015;59 4-6:195-203. doi:10.1387/ijdb.140200md.

23. Ahmed ZM, Yousaf R, Lee BC, Khan SN, Lee S, Lee K, et al. Functional null mutations of MSRB3 encoding methionine sulfoxide reductase are associated with human deafness DFNB74. American Journal Of Human Genetics. 2011;88 1:19-29. doi:10.1016/j.ajhg.2010.11.010.

24. Ni C, Zhang D, Beyer LA, Halsey KE, Fukui H, Raphael Y, et al. Hearing dysfunction in heterozygous Mitf(Mi-wh) /+ mice, a model for Waardenburg syndrome type 2 and Tietz syndrome. Pigment Cell Melanoma Res. 2013;26 1:78-87. doi:10.1111/pcmr.12030.

25. Park HJ, Shaukat S, Liu XZ, Hahn SH, Naz S, Ghosh M, et al. Origins and frequencies of SLC26A4 (PDS) mutations in east and south Asians: global implications for the epidemiology of deafness. Journal Of Medical Genetics. 2003;40 4:242-8.

26. Pryor SP, Madeo AC, Reynolds JC, Sarlis NJ, Arnos KS, Nance WE, et al. SLC26A4/PDS genotype-phenotype correlation in hearing loss with enlargement of the vestibular aqueduct (EVA): evidence that Pendred syndrome and non-syndromic EVA are distinct clinical and genetic entities. Journal Of Medical Genetics. 2005;42 2:159-65. doi:10.1136/jmg.2004.024208.

27. Bakken TE, Roddey JC, Djurovic S, Akshoomoff N, Amaral DG, Bloss CS, et al. Association

of common genetic variants in GPCPD1 with scaling of visual cortical surface area in humans. Proc Natl Acad Sci U S A. 2012;109 10:3985-90. doi:10.1073/pnas.1105829109.

28. Mier P and J P-PA. Fungal Smn and Spf30 homologues are mainly present in filamentous fungi and genomes with many introns: Implications for spinal muscular atrophy. Gene. 2012; 491 2:135-41.

29. Talbot K, Miguel-Aliaga I, Mohaghegh P, Ponting CP and Davies KE. Characterization of a gene encoding survival motor neuron (SMN)-related protein, a constituent of the spliceosome complex. Human Molecular Genetics. 1998;7 13:2149-56. doi:ddb265 [pii].

30. Jiang Y, Xie M, Chen W, Talbot R, Maddox JF, Faraut T, et al. The sheep genome illuminates biology of the rumen and lipid metabolism. Science. 2014;344 6188:1168-73.

31. Johnston SE, McEwan JC, Pickering NK, Kijas JW, Beraldi D, Pilkington JG, et al. Genome-wide association mapping identifies the genetic basis of discrete and quantitative variation in sexual weaponry in a wild sheep population. Mol Ecol. 2011;20 12:2555-66.

32. Dominik S, Henshall JM and Hayes BJ. A single nucleotide polymorphism on chromosome 10 is highly predictive for the polled phenotype in Australian Merino sheep. Anim Genet. 2012;43 4:468-70.

33. Wang XL, Zhou GX, Li Q, Zhao DF and Chen YL. Discovery of SNPs in RXFP2 related to horn types in sheep. Small Ruminant Res. 2014;116 2-3:133-6.

34. Wiedemar N and Drogemuller C. A 1.8-kb insertion in the 3-UTR of RXFP2 is associated with polledness in sheep. Anim Genet. 2015;46 4:457-61.

35. Johnston SE, McEwan JC, Pickering NK, Kijas JW, Beraldi D, Pilkington JG, et al. Genome-wide association mapping identifies the genetic basis of discrete and quantitative variation in sexual weaponry in a wild sheep population. Mol Ecol. 2011;20 12:2555-66. doi:10.1111/j.1365-294X.2011.05076.x.

36. Carlson DF, Lancto CA, Zang B, Kim ES, Walton M, Oldeschulte D, et al. Production of hornless dairy cattle from genome-edited cell lines. Nature Biotechnology. 2016;34 5:479-81. doi:10.1038/nbt.3560.

37. Li H and Durbin R. Fast and accurate short read alignment with Burrows-Wheeler transform. Bioinformatics. 2009;25 14:1754-60.

38. McKenna A, Hanna M, Banks E, Sivachenko A, Cibulskis K, Kernytsky A, et al. The Genome Analysis Toolkit: a MapReduce framework for analyzing next-generation DNA sequencing data. Genome Research. 2010;20 9:1297-303. doi:10.1101/gr.107524.110.

39. Cingolani P, Platts A, Wang LL, Coon M, Nguyen T, Wang L, et al. A program for annotating and predicting the effects of single nucleotide polymorphisms, SnpEff: SNPs in the genome of Drosophila melanogaster strain w(1118); iso-2; iso-3. Fly. 2012;6 2:80-92.

40. Felsenstein J. PHYLIP - Phylogeny Inference Package (Version 3.2). Cladistics. 1989;5: 164-6.

41. Harris RS. *Improved pairwise alignment of genomic DNA.* Improved pairwise alignment of genomic DNA. PhD Thesis, The Pennsylvania State University. PhD Thesis, 2007.

42. Patterson N, Price AL and Reich D. Population structure and eigenanalysis. PloS Genetics. 2006;2 12:2074-93. doi:10.1371/journal.pgen.0020190.

43. Price AL, Patterson NJ, Plenge RM, Weinblatt ME, Shadick NA and Reich D. Principal components analysis corrects for stratification in genome-wide association studies. Nat Genet. 2006;38 8:904-9. doi:10.1038/ng1847.

44. Tang H, Peng J, Wang P and Risch NJ. Estimation of individual admixture: Analytical and study design considerations. Genet Epidemiol. 2005;28 4:289-301. doi:10.1002/gepi.20064.

45. Purcell S, Neale B, Todd-Brown K, Thomas L, Ferreira MAR, Bender D, et al. PLINK: A tool set for whole-genome association and population-based linkage analyses. American Journal Of Human Genetics. 2007;81 3:559-75. doi:10.1086/519795.

46. Danecek P, Auton A, Abecasis G, Albers CA, Banks E, DePristo MA, et al. The variant call format and VCFtools. Bioinformatics. 2011;27 15:2156-8. doi:10.1093/bioinformatics/btr330.

47. Barrett JC, Fry B, Maller J and Daly MJ. Haploview: analysis and visualization of LD and haplotype maps. Bioinformatics. 2005;21 2:263-5. doi:10.1093/bioinformatics/bth457.

48. Stajich JE, Block D, Boulez K, Brenner SE, Chervitz SA, Dagdigian C, et al. The Bioperl toolkit: Perl modules for the life sciences. Genome Research. 2002;12 10:1611-8. doi:10.1101/gr.361602.

49. Sabeti PC, Varilly P, Fry B, Lohmueller J, Hostetter E, Cotsapas C, et al. Genome-wide detection and characterization of positive selection in human populations. Nature. 2007;449 7164:913-8. doi:10.1038/nature06250.

50. Scheet P and Stephens M. A fast and flexible statistical model for large-scale population genotype data: applications to inferring missing genotypes and haplotypic phase. Am J Hum Genet. 2006;78 4:629-44. doi:10.1086/502802.

51. Bindea G, Mlecnik B, Hackl H, Charoentong P, Tosolini M, Kirilovsky A, et al. ClueGO: a Cytoscape plug-in to decipher functionally grouped gene ontology and pathway annotation networks. Bioinformatics. 2009;25 8:1091-3. doi:10.1093/bioinformatics/btp101.

52. Lovly CM, Dahlman KB, Fohn LE, Su Z, Dias-Santagata D, Hicks DJ, et al. Routine multiplex mutational profiling of melanomas enables enrollment in genotype-driven therapeutic trials. PLoS ONE. 2012;7 4:e35309.

53. Zhang R, Rao M, Li C, Cao J, Meng Q, Zheng M, et al. Functional recombinant human anti-HAV antibody expressed in milk of transgenic mice. Transgenic Research. 2009;18 3:445-53. doi:10.1007/s11248-008-9241-0.

Figures and legends

Figure 1. Genetic relationships and population structure in Chinese sheep. (**a**) Geographical distribution of the Chinese indigenous sheep breeds and a European-originated breed (AM) sampled in the present study. The background color of sheep picture represents their belonged lineages (blue: TBS; grey: MGS; green: EUS). (**b**) Neighbor-joining tree of the 99 domestic sheep, and 1 Texel sheep (reference genome) as an out-group point. Each breed is presented using colors consistent with **c**. (**c**) Principal component plot. The first (PC1) and second (PC2) principal components are shown in the figure. (**d**) Population structure analysis of 99 sheep, regarding number of clusters from 2 to 4.

Figure 2. Candidate genes with shared or independent signals of selective sweep in TBS. (**a**) Manhattan plot of the population differentiation F_{ST} for TBS1 vs. MGS and for TBS2 vs. MGS, using a 30-kb sliding window with a step size of 15kb. (**b**) Scatter plot for 30-kb-window F_{ST} statistics over the genome; regions under selective sweep in TBS1 (red), TBS2 (blue) and both populations (green) were highlighted. (**c**) Summary of adaptive signatures of semi-feralization in Tibetan sheep. candidate genes (red: TBS1-specific; green: shared by TBS1 and TBS2) were mapped to key words based on their associated GO terms and literatures.

Figure 3. Selective sweeps on the horn-related gene *RXFP2*. (**a**) Statistics plotted over a ~400 kb region surrounding *RXFP2*, including: 1) population differentiation

(F_{ST}) among TBS1, TBS2 and MGS; 2) heterozygosity in TBS1 and TBS2, calculated as Z-transformed $\log_2(H_{P|TBS1}/H_{P|MGS})$ and $\log_2(H_{P|TBS2}/H_{P|MGS})$; 3) haplotypic length measured by Z-transformed XP-EHH$_{TBS1\ vs.\ MGS}$ and XP-EHH$_{TBS2\ vs.\ MGS}$. (**b**) Haplotypic distributions among 99 sheep of a local region of *RXFP2*. Biallelic SNPs were showed in blue and yellow. (**c**) Alignment of the *RXFP2* protein sequences from 9 vertebrate species. Two protein variants (*RXFP2*: 627 and 641) with top F_{ST} in TBS1 are indicated in red. The dots in the alignment denote amino acids that are identical with those in TBS1. (**d**) Distribution of the haplotype frequency of two protein-altering variants (*RXFP2*: 627 and 641) in 1155 sheep. "C.C" corresponds to V627 + E641 and "T.T" corresponds to M627 + K641.

Figure 4. *RXFP2* haplotype is correlated with horn shape and size. (**a**) Features of SHE-type and TCF-type horns. (**b**) Association between 8 SNPs and horn size; an additive genetic model (assume *A* as major allele, *a* as minor allele, we have code 2 for *AA*, 1 for *Aa* and 0 for *aa*) is applied; pair-wise LD between SNP pairs were plotted at the bottom, where numbers represent *D'* statistics. (**c**) Box-plot of individual horn sizes among different OAR_29461968 genotypes; P value was calculated using linear regression based on additive genetic model, and the fitting line was showed in red. (**d**) Association map between 8 SNPs and horn shape (either TCF or SHE); recessive genetic code (1 for *AA*, 0 for *Aa* and *aa*) was applied (**e**) Distribution of OAR10_29461968 genotypes among PT sheep with different horns.

Figure 5. Gene expression patterns of *RXFP2*. (**a**) Expression of *RXFP2* and

β-actin in 13 tissue samples from PT sheep: 1, heart; 2, liver; 3, spleen; 4, lung; 5, kidney; 6, muscle; 7, brain; 8, ovary; 9, corpus uteri;10, adipose; 11, thyroid; 12, soft horn; 13, horn periosteum. (**b**) Expression pattern of *RXFP2* in SHE-type, TCF-type, scurred soft-horn tissues examined by RT-PCR (left) and real-time PCR (right); error bars denotes S.D. of the mean; groups with significant differences (*: $P < 0.05$; **: $P < 0.001$) were indicated. (**c**) Scatter plot on *RXFP2* expression and horn size; the fitting line of linear regression was showed in red. (**d**) Western blot analysis of soft-horn tissues with different horn types, using antibodies of *RXFP2* and *β*-actin.

Click here to download Figure Fig.1.pdf

Figure 1

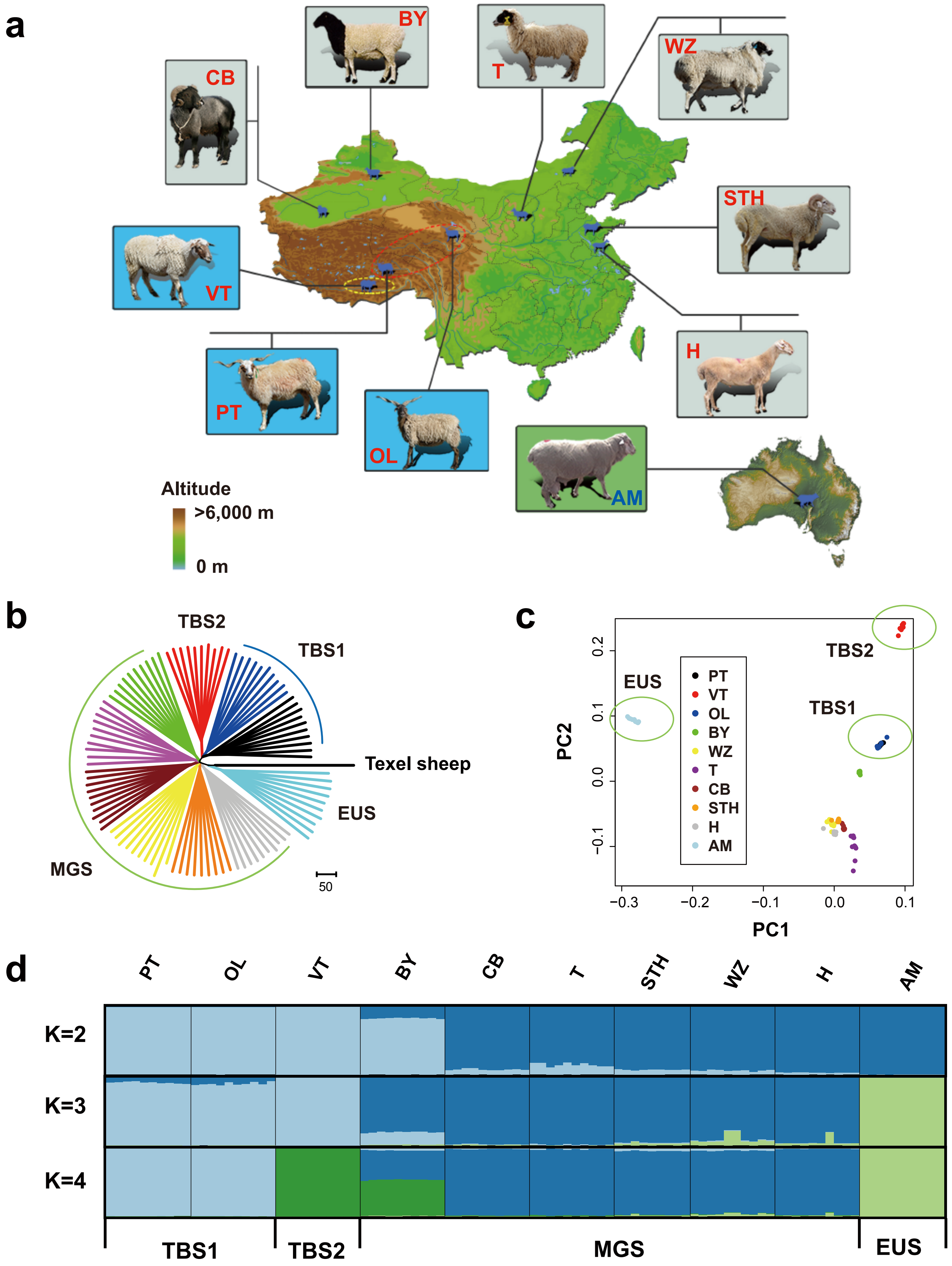

Figure 2

Click here to download Figure Fig.2.pdf

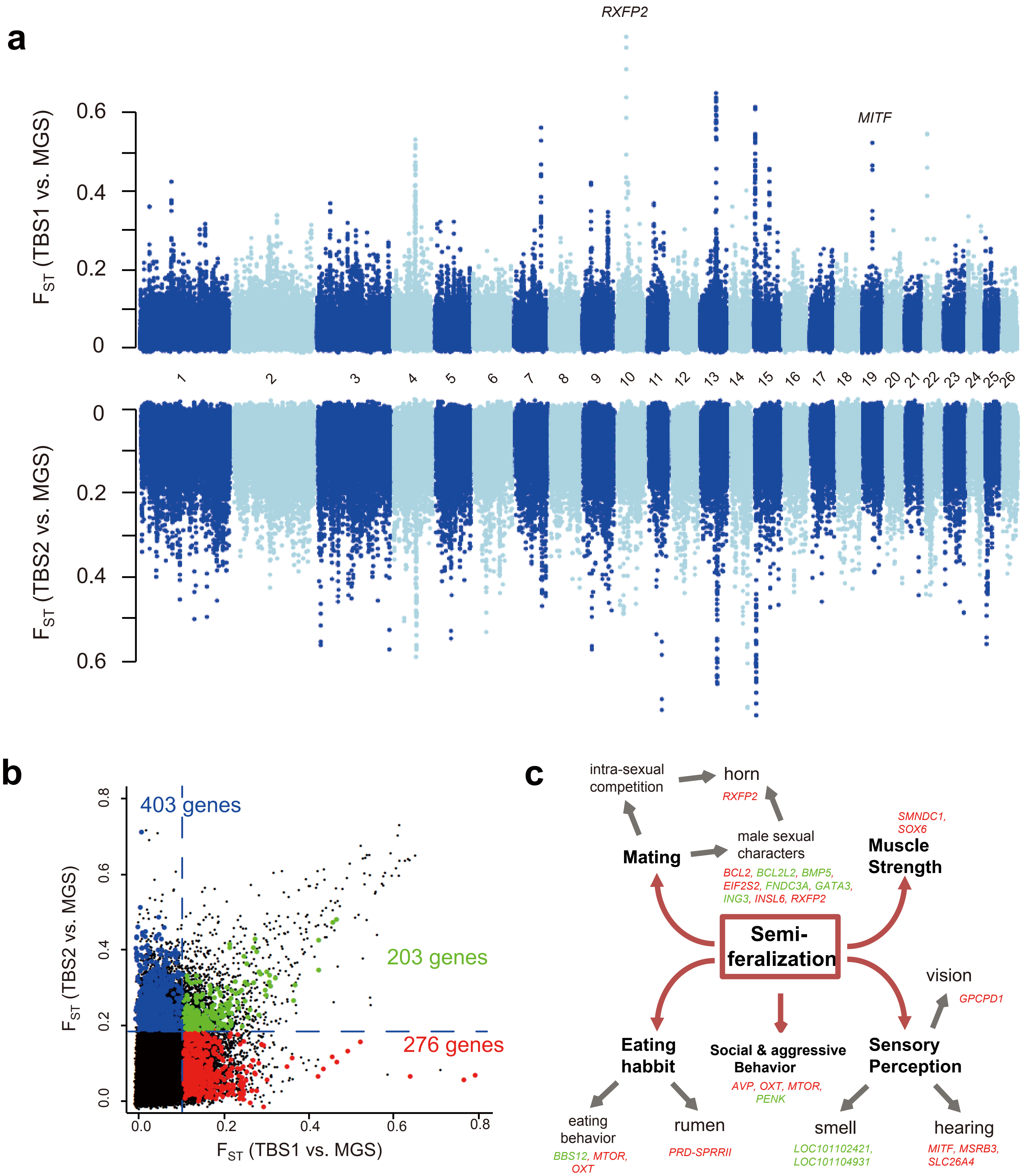

Figure 3

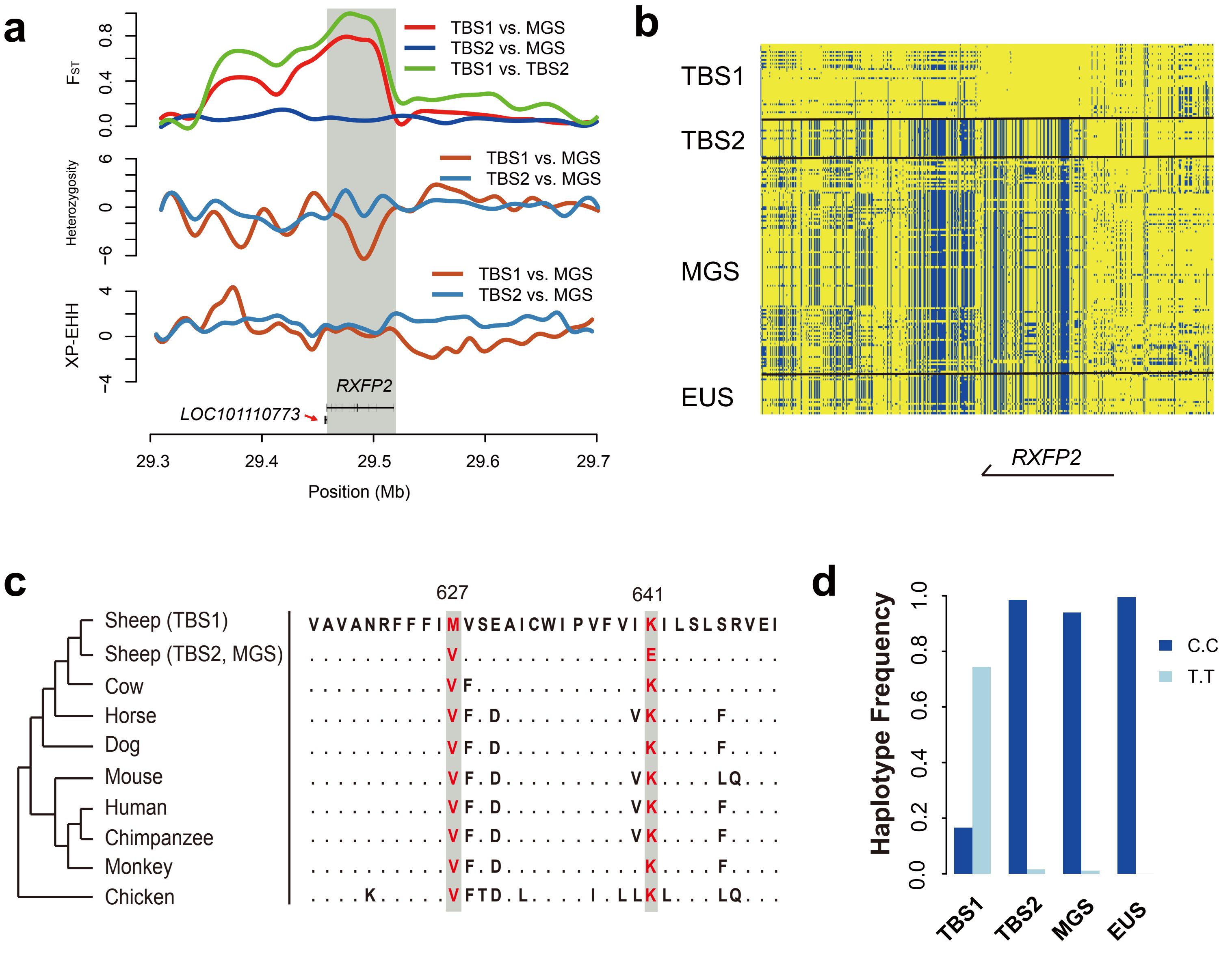
a
FST
Heterozygosity
XP-EHH
TBS1 vs. MGS
TBS2 vs. MGS
TBS1 vs. TBS2
RXFP2
LOC101110773
Position (Mb)
b
TBS1
TBS2
MGS
EUS
RXFP2
c
627
641
Sheep (TBS1)
Sheep (TBS2, MGS)
Cow
Horse
Dog
Mouse
Human
Chimpanzee
Monkey
Chicken
d
Haplotype Frequency
C.C
T.T
TBS1
TBS2
MGS
EUS

Figure 4

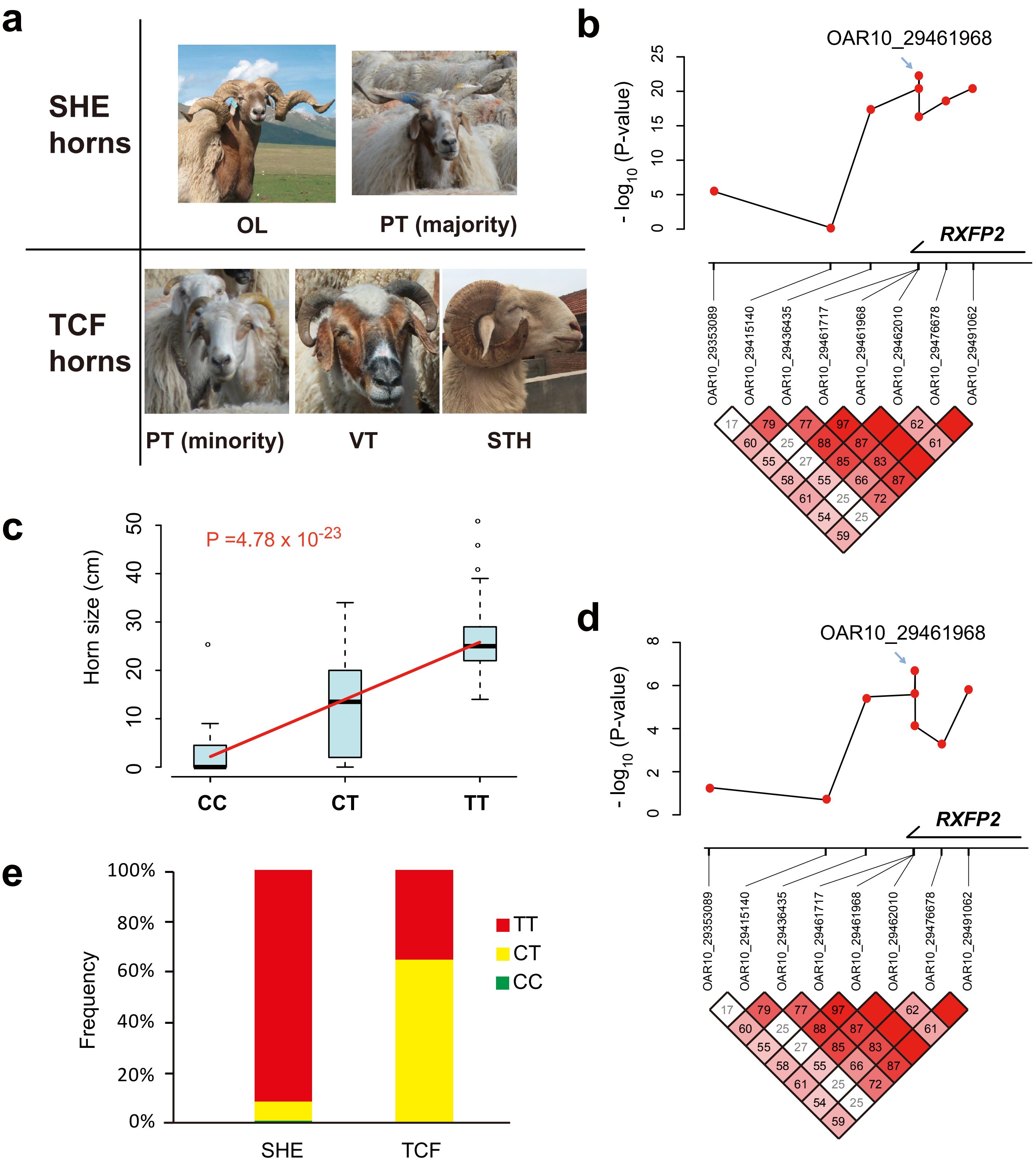
a
SHE horns
OL
PT (majority)
TCF horns
PT (minority)
VT
STH
b
OAR10_29461968
- log10 (P-value)
RXFP2
OAR10_29353089
OAR10_29415140
OAR10_29436435
OAR10_29461717
OAR10_29461968
OAR10_29462010
OAR10_29476678
OAR10_29491062
c
P =4.78 x 10-23
Horn size (cm)
CC
CT
TT
d
OAR10_29461968
- log10 (P-value)
RXFP2
e
Frequency
TT
CT
CC
SHE
TCF

Figure 5 [Click here to download Figure Fig.5.pdf]

Figure 5

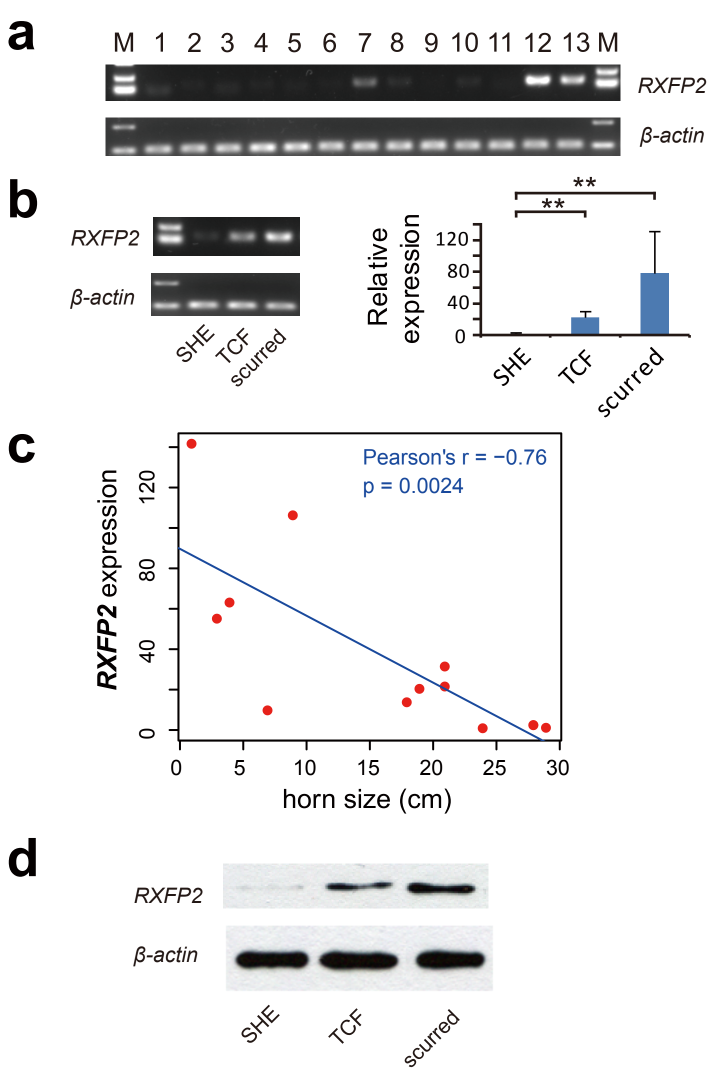

Click here to access/download
Supplementary Material
Supplementary_Materials.doc

Supplementary Tables

Click here to access/download
Supplementary Material
Supplementary Tables.xlsx
