# Supplementary material for: Whole-genome sequences of 89 Chinese sheep suggest role of RXFP2 in the development of unique horn phenotype as response to semi-feralization
Source: Gigascience. 2018 Mar 7;7(4):giy019. doi: 10.1093/gigascience/giy019 (PMC5905515; doi:10.1093/gigascience/giy019)
Supplement: GIGA-D-17-00165_Revision_1.pdf [file giy019_giga-d-17-00165_revision_1.pdf]

GigaScience

Whole-genome sequences of 89 Chinese sheep suggest role of RXFP2 in the development of unique horn phenotype as response to semi-feralization

--Manuscript Draft--

**Manuscript Number:**	GIGA-D-17-00165R1
**Full Title:**	Whole-genome sequences of 89 Chinese sheep suggest role of RXFP2 in the development of unique horn phenotype as response to semi-feralization
**Article Type:**	Research

Funding Information:

Agricultural Science and Technology Innovation Program of China (ASTIP-IAS13)	Prof. Mingxing Chu
Earmarked Fund for China Agriculture Research System (CARS-39)	Prof. Mingxing Chu
National Key Technology Support Program (2013BAI101B09)	Prof. Yixue Li
National Natural Science Foundation of China (CN) (31472078)	Prof. Mingxing Chu
National Natural Science Foundation of China (31402041)	Dr. Qiuyue Liu
National Key Scientific Instrument and Equipment Development Project (2012YQ03026108)	Prof. Yixue Li
National Basic Research Program of China (2011CB910204)	Prof. Yixue Li
National Basic Research Program of China (2011CB510102)	Prof. Yixue Li
Youth Innovation Promotion Association of the Chinese Academy of Sciences (2017325)	Dr. Zhen Wang
Genetically Modified Organisms Breeding Major Program of China (2016ZX08009-003-006)	Dr. Qiuyue Liu
Genetically Modified Organisms Breeding Major Program of China (2016ZX08010-005-003)	Prof. Mingxing Chu
Major Science and Technology Program of Inner Mongolia Autonomous Region of China	Prof. Mingxing Chu

Abstract:

Background
Animal domestication has been extensively studied but the process of feralization remains poorly understood.
Results
Here, we performed whole-genome sequencing of 99 sheep and identified a primary genetic divergence between two heterogeneous populations in the Tibetan Plateau, including one semi-feral lineage. Selective sweep and candidate gene analysis revealed the local adaptations of these sheep associated with sensory perception, muscle strength, eating habbit, mating process and aggressive behavior. In particular, a horn-related gene RXFP2 showed signs of rapid evolution specifically in the semi-feral breeds. A unique haplotype and repressed horn-related-tissue expressions of RXFP2 were correlated with higher horn length, as well as spiral and horizontally extended horn shape.
Conclusions
Semi-feralization has an extensive impact on diverse phenotypic traits of sheep. By acquiring features similar to those of their wild ancestors, semi-feral sheep were able to re-gain fitness in frequent contact with wild surroundings and rare human interventions.

Powered by Editorial Manager® and ProduXion Manager® from Aries Systems Corporation

	The present study provides a new insight into the evolution of domestic animals when human interventions are no longer dominant.
**Corresponding Author:**	Shengdi Li<br><br>CHINA
**Corresponding Author Secondary Information:**	
**Corresponding Author's Institution:**	
**Corresponding Author's Secondary Institution:**	
**First Author:**	Zhangyuan Pan
**First Author Secondary Information:**	
**Order of Authors:**	Zhangyuan Pan
	Shengdi Li
	Qiuyue Liu
	Zhen Wang
	Zhengkui Zhou
	Ran Di
	Benpeng Miao
	Wenping Hu
	Xiangyu Wang
	Xiaoxiang Hu
	Ze Xu
	Dongkai Wei
	Xiaoyun He
	Liyun Yuan
	Xiaofei Guo
	Benmeng Liang
	Ruichao Wang
	Xiaoyu Li
	Xiaohan Cao
	Xinlong Dong
	Qing Xia
	Hongcai Shi
	Geng Hao
	Jean Yang
	Cuicheng Luosang
	Yiqiang Zhao
	Mei Jin
	Yingjie Zhang
	Shenjin Lv

Powered by Editorial Manager® and ProduXion Manager® from Aries Systems Corporation

	Fukuan Li
	Guohui Ding
	Mingxing Chu
	Yixue Li
**Order of Authors Secondary Information:**	
**Response to Reviewers:**	A general statement from the authors:<br>We thank all the reviewers for their great work in reviewing our manuscript entitled "Whole-genome sequences of 89 Chinese sheep suggest role of RXFP2 in the development of unique horn phenotype as response to semi-feralization". Indeed, the advices and comments are provoke-thinking and helpful to improve the quality of our work. We have considered the questions and suggestions raised by the reviewers, and revised the manuscript accordingly. We list some major revisions here:<br>Additional analyses:<br>1.We performed selective sweep analysis, gene ontology analysis, RXFP2 haplotype distribution calculation after breaking down four populations PT, OL, VT and BY.<br>2.We did target sequencing of five random genomic regions in original samples (76 out of 99 sheep) to evaluate the accuracy of our WGS variant calling approach.<br>3.We compared the candidate gene list of present study with those from two previous approaches of Chinese native sheep.<br>4.We compared the RXFP2 haplotype in our dataset with that reported in wild bighorn sheep (based on their vcf genotype file, downloaded from internet).<br>5.We calculated breed-to-breed FST distance and generated a NJ tree.<br>Figures:<br>1.Figure 1b is replaced with a FST tree.<br>2.Original Figure 2 is broken down into two figures. Currently, Figure 2 is comprised of FST and HP manhattan plots from four divergent populations; Figure 3 shows the result of candidate gene analysis.<br>3.Figure details and legend texts are revised according to the suggestions by reviewers.<br>Main text:<br>1.The Introduction and Discussions were revised after we have consulted literatures recommended by the reviewers.<br>2.The Analyses section was revised based on new results (separately in four populations).<br>3.We marked up some of the revisions in the main text, those we think are essential for addressing the question asked by the reviewers.<br>4.Typos have been carefully checked and corrected. The manuscript was edited by a native-English speaker before uploading.<br><br>Point-to-point responses<br>Authors: Here are our point-to-point responses to the reviewers' questions. Please note that for each reviewer we grouped the comments on typos and language editing as one point.<br>Reviewer 1<br>General comments by reviewer:<br>Reviewer: The authors have performed sequencing of 99 sheep from multiple populations in China (and Australia). They identify many regions with high Fst as well as reduced heterozygosity. Their top region contains the RXFP2 gene, which they use for functional analysis linking it to horn size (as has been reported earlier).<br><br>Major comments and responses:<br>1. Reviewer: Figure 1 nicely summarizes the breeds and their geographic location and their genomic relationship. However, the authors quickly put the PT and OL population together labeling it TBS1. The VT population is relabeled TBP2. Since the TBS1 population separates according to the two breeds these populations should be treated separately. The paper needs to be redone throughout based on keeping the two (TBS1) populations separate.<br>Authors: It is a great suggestion to provide more information by treating each breed separately, even if we think PT and OL may share a similar trajectory of adaptation (as they are genetically proximate according to the PCA and admixture plot). In the revised manuscript, we reported selective sweep and candidate gene analysis in PT, OL, VT

	and BY, by comparing them separately with MGS (line 182-190, also see Figure 2, 3a-c). Overlaps between gene sets in different breeds were showed as venn plots (Figure 3a).<br><br>2. Reviewer: Figure 2 shows the Fst for TBS1 vs MGS and TBP2 vs MGS. It would be great to be able to compare the Fst plots of PT, OL, VT and BY separately compared to the MGS. This would allow the comparison of the signals that come up in different combinations of populations. I.e. PT & OL = big SHE horn; PT, OL, VT and BY = high altitude.<br>Authors: We agree that showing more Manhattan plots potentially gives more combinations of parameters, like horn size and altitudes. To address this, we analyzed the selective sweep and associated candidate genes in PT, OL, VT and BY compared with MGS (line 182-190). The result shows that BY has a smaller gene set (n=758) than PT, OL and VT (n=1125; 999; 1046), which makes sense because BY has relatively small genetic differences from other MGS lineages. Moreover, inclusion of the BY gene set revealed NF1 as a consistent signal across four populations (Figure 3a, c). However, more hypoxia-related genes (e.g. PTEN, PINK1) have undergone lineage-specific evolution (Figure 3c).<br>It is also worth noting that BY may not necessarily share their altitude adaptation genes with TBS. The polygenic basis of hypoxic response pathway means that different high-altitude lineages may have multiple ways to achieve the similar adaptations (a well-known example is the Tibetan chicken). In this case, although it would be interesting to know whether some “altitude genes” are shared by TBS and BY, considering consistency among high-altitude populations (PT, VT, OL, BY) may not be a golden standard to detect all “altitude genes”, since BY is a distant population and the adaptations are more likely to be lineage-specific. This concern is discussed in line 244-249.<br><br>3. Reviewer: While the data would be clearer if each of the four populations were shown, the authors may want to comment on the regions now found on chrom 13 and 15 - maybe they are related to altitude?<br>Authors: A consistently high FST in PT vs. MGS, OL vs. MGS and VT vs. MGS could be explained by a selective sweep either in TBS (PT + OL + VT), or MGS. A simple way to distinguish these two possibilities is to check whether TBS or MGS shows reduction of heterozygosity, or to calculate which haplotype is more different from the ancestral state (the lineage-specific branch length, LSBL). According to Figure 2, the window FST and HP signals on chromosome 13 and 15 suggest the latter possibility, which means they might contribute to the adaptive evolution of MGS.<br><br>4. Reviewer: The authors also need to compare the identified Fst regions with those found in previous sheet selection studies. It would also be interesting to compare the exact RXFP2 haplotypes 'associated' with horn here and in previous studies.<br>Authors: We followed this helpful suggestion and compared the candidate genes at all sweep regions we found (PT, OL, VT and BY vs. MGS) with two previous studies of Chinese sheep (Supplementary Table S12). Genes underlying altitude adaptations such as NF1 were confirmed by multiple studies. However, our data suggested a large number of genes underwent lineage-specific evolution, which is probably the reason why they are not identified when treating all Tibetan lineages as one group in other approaches.<br>We also compare the RXFP2 genotypic data in our 99 sheep with that from the wild bighorn sheep (Supplementary Figure S9, S10), whose WGS data are easily accessible from the Dryad (doi:10.5061/dryad.3f2t2). The result suggested that PT and OL were basically carrying a different haplotype from the bighorn sheep, although they were all subject to selective pressures at the same locus RXFP2 for developing strong horns.<br>We also think it would be informative if we can directly compare our genotypic data with more populations based on SNP chips. We consulted the most extensive collection of world-wide ovine genotypic data [1]. Nevertheless, we found that the SNP markers near RXFP2 are not polymorphic in our dataset, which means that these markers cannot be used to explain the haplotypic differences between SHE and TCF populations in our study.<br><br>5. Reviewer: How were SNPs/genotypes called given the low coverage sequencing data? Were all data from a breed analyzed together?

Powered by Editorial Manager® and ProduXion Manager® from Aries Systems Corporation

Authors: The variant calling step was performed by an early version of samtools (v1.2), which by default imputed missing genotypes based on other samples that were simultaneously processed. All data from one breed (n=10) were analyzed together, and then data from different breeds were merged into single variant file.
The advantage of the low-coverage design is to have more samples when given a total coverage depth for each breed (average depth per breed = average depth per sample × samples per breed), while the disadvantage is, apparently, the higher missing rate at each genetic variant. However, genotypic imputation (based on individuals from same breed) and window-based metrics compromised the problem, as the missing variants are supposed to be randomly distributed on the genome and among individuals.

6. Reviewer: Using multiple breeds for the analysis it might be possible to break down the selection signal near/over the RXFP2 signal (p 10 first paragraph, p13 1st paragraph - are haplotypes the same in the two semi-feral populations?)
Authors: It is a very helpful suggestion to break down the signal of selective sweep by each population. In the current version of Figure 4 and its related sections in the main text (line 254-275), we separately showed signals of each population (PT, OL, VT and BY), and the result was consistent with the previous one when PT and OL were combined. Basically, PT and OL have one dominant haplotype, and rest of the sheep breeds has another. A slight difference in haplotype frequency was observed between PT and OL as showed in Figure 4d. This is in rough agreement with the fact that SHE-type horns comprise >70% of PT population (in our 182 PT dataset), and is nearly fixed in OL population.

Minor comments and responses:
7. Reviewer: L36 "RXFP2 underlied rapid evolution" replace with "RXFP2 showed signs of rapid evolution"
-L43: "frequent contact with wild surroundings and rare human interventions".
-Keywords: add "sheep"
-L51: "the process where domestic animals"
-L54 "fit natural life while human artificial selection is no longer"
-L57 "trace back to 8 ka" replace with "trace back to 8,000 years ago "
-L58 "mito-genomic evolutionary study"
-L67-69: "as the Tibetan Plateau is rich in grasslands, the local breeds, especially ones living on prairies, have been roaming with nomads and fed on natural ranches"
-L71 "Third, unlike in other"
-L75 "loosened"
-L77-78 "breed from the Tibetan Plateau"
-L79 "sweeps"
-L84: "three"
- L118: "a rooted tree using the genome of the goat"
-L128 "performed principal component"
-L129 "Despite the division"
-L142-3 "statistics across the genome"
-L152 "from a relatively"
-L177 "observed the strongest signal"
-L178 "on chromosome 10"
-L181 "A previous study"
-L197 "in regions of positive selection"
-L199 "processes"
-L213 "intensely"
-L217 "a correlation"
-L231 "the TBS1 populations"
-L234-5 "the harsh environment on the Tibetan"
-L247 "variants in RXFP2"
-L297-8 "over the RXFP2 locus"
-L307 "compared the gene expression in"
-L308 "Despite obvious"
-L322 "study suggest that domestic animals might have re-acquired"
-L331 "to the Tibetan"
-L333 "selective sweeps"
-L340 "in competition to reproduce"
-L373 "evidence"
-L380 "across the RXFP2 gene"

Powered by Editorial Manager® and ProduXion Manager® from Aries Systems Corporation

	-L483 "in RXFP2"<br>-L490 "genotyped in 182"<br>-L533 "levels of the five genes"<br>-p37: Figure 1 legend: spell out the names/shortenings of all sheep populations<br>-L746 "of the sheep pictures represent their lineage"<br>-L751 "clusters from K=2-4"<br>-L755-6 "regions under selection in TBS1"<br>-L768 "in red. For 627 TBS1 has the variant allele, whereas for 641 it has the reference allele"<br>-L779 Fig 4d: write how many individuals were included in analysis<br>-L789 linear regression is shown in red - it is not red - please correct<br>Authors: We appreciate these helpful suggestions to improve the quality of text. All these details were revised accordingly, and the manuscript was edited by a native speaker before uploading.<br><br>8. Reviewer: - L90 when you describe the two different semi-feral populations, you may want to indicate also which sheep are at high altitude as this might be an important parameter.<br>Authors: As suggested by the reviewer, we add a sentence at line 95-98 to indicate PT, VT, OL and BY as four high-altitude populations.<br><br>9. Reviewer: - L105 You describe that your dataset encompasses 94% of variants found in dbSNP. Can you say also how many novel SNPs you found?<br>Authors: >37.3% of variants in our dataset were novel compared with dbSNP build 143. This information is supplemented in the current version of manuscript at line 112-113.<br><br>10. Reviewer: - L190 MITF is being linked to hearing - it is worth noting that MITF is also frequently mutated in different coat color types so I don't think you know which is the case in this study.<br>Authors: We agree with the reviewer on this point. In the revised manuscript, we have now emphasized that some of the candidate genes like MITF might have multiple phenotypic outcomes (at line 208-211), and we also highlighted the double function of MITF in Figure 3b.<br><br>11. Reviewer: - A larger number of genes are described on p10 and it is not clear how they were selected and how they were assigned potential function.<br>Authors: We now use Figure 3b to summarize the selection signals of these typical genes, as well as the key words of their functional categories related with semi-feralization (also see Supplementary Table S19).<br><br>12. Reviewer: - p16 top paragraph: how were the five genes tested for expression chosen - what was rationale for 900 kb?<br>Authors: The number 900 was a typo in our original submission. The five genes we tested actually covered a ~1Mb region from chr10:28,984,259 bp to chr10: 30,002,883 bp.<br>The idea of this step is to include functional genes that are possibly affected by the selective sweep observed near RXFP2. The major concern is that the observed sweep signal might be hitchhiking with a causal variant located in the regulatory DNA elements of the flanking genes. From the LD plot showed in Supplementary Figure S13, it is obvious that the 1Mb region is comprised of multiple LD blocks, which means that the selected window size should be big enough to include all potential hitchhiking variants. Since we did not observed concordant expression level change in flanking genes, it is strong supporting evidence that RXFP2, instead of hitchhiking genes, is causal to the horn phenotypic changes. See revision at line 326-327.<br><br>13. Reviewer: -p18 first paragraph discussing expression pattern - would be useful to mention how many tissues you tried for expression before saying the RXFP2 gene is specifically expressed.<br>Authors: The sentence is now revised as “Thirdly, gene expression analysis in 13 tissues of PT sheep demonstrated …”. (line 364-365)<br><br>14. Reviewer: -L363+ How do you know whether sheep have been selected for a new mutation versus picked up the ancestral state again? Can you find the key candidate

Powered by Editorial Manager® and ProduXion Manager® from Aries Systems Corporation

mutations and see if they if they overlap between ancestral wild sheep and the re-feralized populations?
Authors: Our observations suggest PT and OL sheep were selected to evolve big and aggressive horns in a similar way like their wild relatives (because of sexual competition), but indeed the data is not sufficient to clarify whether they have derived a new genotype or picked up an old one. We showed two candidate protein mutations 627 and 641 with high FST and strong correlation with horn phenotypes (Figure 4c). Nevertheless, their phylogenetic origins are contradictory (where at 641 PT and OL have the ancestral allele, at 627 they have the mutated allele). We also compared the RXFP2 haplotype of our populations with the wild bighorn sheep (Supplementary Figure S9-S10), but failed to see a correlation between the wild population and SHE horned PT and OL. To further clarify the origin of this phenotype, it would be interesting to test whether the RXFP2 haplotype conferring SHE horns might have come from other sheep population with similar horn shapes (Racka), as well as from other wild populations regarded as the ancestors of Chinese sheep, but this is not currently achievable because there is no available data.

15. Reviewer: -p21 first paragraph - how much false positive and false negative variants do you expect?
Authors: To address this question, we performed targeted sequencing of five random genomic regions (including 33 SNPs) over 72 out of the 99 original samples. The results showed in Supplementary Table S26 were utilized to calculate false positive and false negative rates of the SNP calling step. The false positive rate (FPR) defined as the proportion of wrongly defined mutated alleles is 3.34% (FPR = 34/1017) in tested samples, while the false negative rate (FNR) defined as the proportion of wrongly defined reference alleles is 1.47% (FNR = 53/3611).
Sanger (Validation)
RefAltTotal
WGS variant callingRef3558343630
Alt53945998
Total36111017
Ref, Reference allele; Alt, Alternative allele;
According to the ensuing validation, heterozygotes has a much higher false calling rate than homozygotes: 63 out of 411 heterozygotes and 19 out of 1,903 homozygotes were not correctly genotyped in variant calling step (error rate: heterozygotes = 15.3%; homozygotes = 1.0%). This error type distribution does not exceed our expectation, because calling genotypes of heterozygotes in diploid organisms often requires a high coverage depth. The average read depth for each individual is about 6X in our study (one allele is overlapped with ~3 reads), which is quite satisfactory for detecting population-wide allele frequencies, but with restricted power to annotate allelic heterogeneity.

16. Reviewer: -L449 - Is it possible that the 46 windows with few variants may be selected similarly in both populations?
Authors: We list in the current Supplementary Table S28 the sweep statistics for the 46 removed windows. The rationale for removing these windows is because the sweep signals are less convincing when there are few numbers of variants. Indeed, there are significant FST and HP also in these few variant regions, but it is difficult to test whether that is caused by a few random fixations without sufficient observations of genetic hitchhiking. On a different note, with a relatively small window size and step size (30kb, 15kb), a selective sweep signal is often broken down into multiple windows (e.g. the signal on chromosome 20), which enables us to detect most of the sweeps even if we exclude 46 few variant regions.

17. Reviewer: -23 last paragraph: it is quite possible some genes, such as MITF might have multiple mutations/multiple sweep signals across the gene for different coat color patterns. I would therefore not use the majority rules but actually report all signals.
Authors: We agree that the majority rule is not applicable if one gene has multiple sweep events. We now have defined candidate genes from each population separately (based on FST and HP), and then calculated their overlaps like showed in Figure 3a.

18. Reviewer: -L765 add the window size used around the gene
Authors: The visualized window is from chromosome 10: 29,400,000-29,550,000 bp. This information is added to the legend text (line 804-805).

Powered by Editorial Manager® and ProduXion Manager® from Aries Systems Corporation

19. Reviewer: Figure 1: a. Make lines from map to breed not have extra lines
b/c. replace tBS labels with individual breed labels
Authors: The figure is revised according to the suggestion.

Figure 2: adjust to show all four divergent populations in parallel
Authors: The current version of Figure 2 contains parallel comparison of FST and HP from four divergent populations.
Reviewer 2
General comments by reviewer:
Reviewer: This is a useful study on whole-genome sequences from Chinese sheep, which yields evidence that a mutation in the RXPF2 gene is causative for a horn phenotype as adaptation to semi-feralization. The analysis is according to the state-of-the-art. It is likely that the dataset harbors many more mutations in several of the hundreds of genes implicated in environmental adaptation, so there are clear opportunities for follow-up studies. The present results are interesting and deserve publication after a major revision.

Major comments and responses:
1. Reviewer: We understand that a grandiloquent title attracts attention. However, this title does not mention that the study is focused on Chinese sheep and does not refer to RXPF2, the major target of this study, the subject in three of the five figures and also dominating the Discussion. A possible alternative:
Whole-genome sequences of 89 Chinese sheep breeds suggest a role of RXFP2 in the development of a unique horn phenotype as response to semi-feralization.
Note that such a more informative title transmits the same message as the present one (and does it even better).
Authors: As suggested by the reviewer, we have now revised the title as "Whole-genome sequences of 89 Chinese sheep suggest role of RXFP2 in the development of unique horn phenotype as response to semi-feralization".

2. Reviewer: Giga amounts of data require time-consuming analyses, which can only deliver a small part of the potential output. However, this should not be at the expense of an essential part of any scientific report: a comparison with previous literature, mentioning result that are not entirely novel but confirm previous findings. Ref. 14 reports WGS of 80 sheep from 3 climate zones in China. Because in this study Tibetan sheep were treated as one group, RXFP2 as gene subject to selection has been missed. However, Yang et al. [14] also target the high-altitude adaptation of Tibetan sheep, highlighting the role of SOCS2. A complete meta-analysis would be most fruitful, but is outside the scope of the current submission. Nevertheless, the Introduction and Discussion should pay more attention to the previous study [14] and at least touch the following points:
(1) The introduction should refer to the demographic history of the main groups of Chinese breeds [14; Zhao et al. (2017), Genomic reconstruction of the history of Chinese native sheep: insights into peopling role of nomadic nationalities societies and expansions of early pastoralism. Mol. Biol. Evol., in press and accessible via Internet].
Authors: We appreciate this helpful suggestion and agree that the Introduction and Discussion would be more informative after revision on these points. We have consulted the papers suggested by the reviewer, and revised the second paragraph of the Introduction. In its current form, we briefly describe the demographic history and geographic distributions of Chinese sheep based on literatures. This is in order to give a general picture of domestic sheep origin and their spread in China, as well as the sequential order of the split of major ovine groups from their ancestral lineage.

Reviewer: (2) How are the PT, OL and VT breeds related to the Nagqu (ZNQ), Qamdo (ZCD), Shigatse (ZRK), Nyingchi (ZLZ), also from Tibet [14]? Lines 147-150 mention only briefly the proximity of VT and ZLZ. I recommend a Supplementary map giving the locations of the populations studied in [14] and in the present report.
Authors: As suggested by the reviewer, we have provided a map (Supplementary Figure S5) to show the geographic distribution of different Tibetan lineages in our study and in the previous study of native sheep. The introduction of this geographic distribution patterns is described in a separate paragraph at line 152-159.

Reviewer: (3) If phenotypic data are available for the other Tibetan breeds [14]: do they

also have twisted SHE horns?
(4) If so, and assuming the WGS data from [14] are accessible: do they also have the same RXPF2 mutation? This would lend strong support to the message of this study!
Authors: We agree that a direct comparison between our genotypic data and that from the suggested reference paper will be valuable. However, the raw reads or genotypic data from the suggested reference paper has not been released to any public resource. We also tried to contact the authors, but failed to get the access.

(5) Do both studies share other genes as being implicated in adaptation to the high-altitude and hypoxia? I saw that at least part of the genes listed in the Supplementary Table 10 are also mentioned in [14] as being selected in Tibetan sheep. It is relevant to indicate these shared genes in Table S10, if only to indicate that these results are not novel.
Authors: In the current Supplementary Table S12, we have listed the candidate genes in our four populations PT, OL, VT and BY, and their overlap with two previous gene list from references [2] and [3].

3. Reviewer: At the beginning of the Discussion, a clear survey of the most essential features of diversity pattern would support the take-home message: a separate position of Tibetan sheep; within these sheep a contrast of domestic and semi-feral breeds, the former even less diverse than the latter; development of a unique semi-feral horn morphology as plausible adaptation to semi-feralization.
Authors: We followed the suggestion and revised the first paragraph of the Discussion accordingly (line 346-353).

4. Reviewer: Fig. 1b: a tree of NeighborNet graph of FST genetic distance between the breeds will be more informative and better support the message of this paper.
Authors: Indeed, the FST tree can better represent the structure across breeds. From the current Figure 1b, a clear relationship between 10 breeds is showed. Nevertheless, we also preserved the previous phylogeny tree in Supplementary Figure S2 because it provided additional information of the relationship between individual samples, and the position of root (goat).

5. Reviewer: The manuscript needs to be read by a native-English speaker, preferable a scientist, in order to weed out the several awkward phrasings. A few are mentioned below.
Line 49: "in order to understand better (etc.)".
Line 52: you probably mean that protection offered by the domestic habitat suppresses the original environmental adaptation.
Line 108: rephrase in order to indicate more clearly that the nucleotide diversity in Tibetan breeds is higher than in other Chinese breeds.
Lines 265-266, rephrase: "The SHE horns are clearly different from the horns of (etc.)".
Line 331: "the Tibetan Plateau". Lines 331-333: awkward and superfluous sentence.
Line 333: selective sweeps [plural].
Line 361: "naturalistic" refers to an artistic style; probably you mean a natural wildlife habitat.
Line 543: Goa -> goat
Authors: We have carefully checked and revised these sentences mentioned by the reviewer. The revised manuscript was edited by a native speaker before uploading.

Minor comments and responses:
6. Reviewer: Fig. 1: please define in the legends the abbreviations for the breed categories (EUS, MGS, TBS1, TBS2).
Authors: We revised the legend text of Figure 1. Its current form contains definition of all abbreviations for breeds and lineages.

7. Reviewer: It is a good idea to use colors consistently across figures. However, in Fig. 1a the MGS sheep should be shown at a dark blue background and the TBS sheep at a light blue background instead of vice versa in order to harmonize with Figs. 1d and S3.
Authors: We realize that some color inconsistency in our figures is misleading to the readers. To address the problem, we have adjusted the color in Figure 1 and Figure S3. Also, we paid more attention to color consistency in other figures, such like that between Figure 2 and 3c.

8. Reviewer: Data have been submitted to the SRA. In addition, it would be most useful to submit the novel SNPs to the Ensemble Variation Archive.
Authors: We assume you mean the European Variation Archive (EVA), which collects variation data from non-human organisms.
The vcf file was submitted to EVA before uploading this revised manuscript. All data has been released to the public. See data access information at line 557-560

9. Reviewer: Line 62: also refer to [14] and Zhao et al. (2017), who on the basis of genome-wide SNPs differentiate three breed clusters.
Authors: We revised the introduction of Chinese sheep lineages based on the suggested literature (line 57-67).

10. Reviewer: Lines 147-149: just mention the close proximity of ZLZ and VT and the comparably low LD. See point 2 about a more complete comparison of these breeds and other Tibetan breeds [14], which should precede this paragraph.
Authors: The sentence is revised as “Moreover, population ZLZ in the other study was proximate to VT and also exhibited a sign of population bottleneck (evidenced by slow LD decay).” (line 166-168)
Also, the geographic distribution of our 3 populations and the 4 populations in [2] is now discussed in the preceding paragraph at line 152-159.

11. Reviewer: Lines 150-153: just mention that the LD indicates a population bottleneck.
Authors: The sentence is revised as “and also exhibited a sign of population bottleneck (evidenced by slow LD decay)” (line 166-168).

12. Reviewer: Lines 154-156: this was already convincingly clear on the basis of Fig. 1.
Authors: We deleted this paragraph.

13. Reviewer: Lines 188-190 repeat the preceding paragraph; this should be integrated.
Authors: We revised this sentence, so it now describes signals in addition to RXFP2 (line 200).

14. Reviewer: In this context, it is should be mentioned that the well-known Hungarian Racka sheep also has SHE horns (haven't they?).
Authors: It is an intriguing similarity between SHE-horned Tibetan sheep and Hungarian Racka sheep, which we hadn’t noticed before. An important question behind this is whether the SHE-horn genotype is newly derived in semi-feral TBS, or is an introgression from other sheep populations. We are not sure which is the case, since we don’t have the genotypic data from other possible “donors” of RXFP2 haplotypes, including Racka. From our data, what is certain, however, is that this haplotype of RXFP2 confers SHE horns, and is nearly driven to fixation in semi-feral TBS under positive selection. See our discussion at line 375-381.

15. Reviewer: Lines 268, 362: of course, the horns are used during fighting with competitors and predators, but it is a bit curious to state that SHE sheep and the wild ancestors look strong and aggressive; better omit these statements.
Authors: As suggested, we deleted these sentences.

16. Reviewer: Figs. 4b and 4d can easily be combined, while the legends should mention more clearly that (as I understand) they show correlations with horn length and horn shape, respectively.
Authors: These two figures are now combined into Figure 5b, where different line types were used to indicate the measurement outcome (either horn size or shape).

17. Reviewer: Lines 317-323: this paragraph can be omitted since the same points will be made in the Discussion (where it belongs anyway).
Authors: As suggested, we removed this paragraph.

References

1.Kijas JW, Lenstra JA, Hayes B, Boitard S, Neto LRP, San Cristobal M, et al.

	Genome-Wide Analysis of the World's Sheep Breeds Reveals High Levels of Historic Mixture and Strong Recent Selection. Plos Biology. 2012;10 2.<br>2.Yang J, Li WR, Lv FH, He SG, Tian SL, Peng WF, et al. Whole-genome sequencing of native sheep provides insights into rapid adaptations to extreme environments. Molecular Biology and Evolution. 2016;33:2576-92. doi:10.1093/molbev/msw129.<br>3.Wei C, Wang H, Liu G, Zhao F, Kijas JW, Ma Y, et al. Genome-wide analysis reveals adaptation to high altitudes in Tibetan sheep. Scientific reports. 2016;6:26770. doi:10.1038/srep26770.
**Additional Information:**	
**Question**	**Response**
Are you submitting this manuscript to a special series or article collection?	No
**Experimental design and statistics**<br><br>Full details of the experimental design and statistical methods used should be given in the Methods section, as detailed in our Minimum Standards Reporting Checklist. Information essential to interpreting the data presented should be made available in the figure legends.<br><br>Have you included all the information requested in your manuscript?	Yes
**Resources**<br><br>A description of all resources used, including antibodies, cell lines, animals and software tools, with enough information to allow them to be uniquely identified, should be included in the Methods section. Authors are strongly encouraged to cite Research Resource Identifiers (RRIDs) for antibodies, model organisms and tools, where possible.<br><br>Have you included the information requested as detailed in our Minimum Standards Reporting Checklist?	Yes
**Availability of data and materials**<br><br>All datasets and code on which the conclusions of the paper rely must be either included in your submission or deposited in publicly available repositories (where available and ethically appropriate), referencing such data using a unique identifier in the references and in the "Availability of Data and Materials" section of your manuscript.	Yes

Powered by Editorial Manager® and ProduXion Manager® from Aries Systems Corporation

Have you have met the above requirement as detailed in our Minimum Standards Reporting Checklist?	

Powered by Editorial Manager® and ProduXion Manager® from Aries Systems Corporation

Revised Manuscript Click here to download Manuscript Manuscript.marked_up.doc

Whole-genome sequences of 89 Chinese sheep suggest role of *RXFP2* in the development of unique horn phenotype as response to semi-feralization

Zhangyuan Pan[†,1,3], Shengdi Li[†,2,4], Qiuyue Liu[†,1], Zhen Wang[2], Zhengkui Zhou[1], Ran Di[1], Benpeng Miao[2,4], Wenping Hu[1], Xiangyu Wang[1], Xiaoxiang Hu[5], Ze Xu[6], Dongkai Wei[6], Xiaoyun He[1], Liyun Yuan[2], Xiaofei Guo[1], Benmeng Liang[1], Ruichao Wang[2], Xiaoyu Li[1], Xiaohan Cao[1], Xinlong Dong[1], Qing Xia[1], Hongcai Shi[7], Geng Hao[8], Jean Yang[9], Cuicheng Luosang[9], Yiqiang Zhao[5], Mei Jin[10], Yingjie Zhang[11], Shenjin Lv[3], Fukuan Li[3], Guohui Ding[2,12], Mingxing Chu*[,1] & Yixue Li*[,2,12]

[1]Institute of Animal Science, Chinese Academy of Agricultural Sciences, Beijing, China.

[2]Key Lab of Computational Biology, CAS-MPG Partner Institute for Computational Biology, Shanghai Institutes for Biological Sciences, Chinese Academy of Sciences, Shanghai, China.

[3]College of Agriculture and Forestry Science, Linyi University, Linyi, China

[4]University of Chinese Academy of Sciences, Beijing, China;

[5]State Key Laboratory for Agrobiotechnology, China Agricultural University, Beijing, China.

[6]BasePair BioTechonology Co., Ltd., Suzhou, China.

[7]Institute of Biotechnology, Xinjiang Academy of Animal Science, Urumqi, China.

[8]Institute of Animal Science, Xinjiang Academy of Animal Science, Urumqi, China.

[9]Research Institute of Animal Science, Tibet Academy of Agricultural and Animal Husbandry Sciences, Lhasa, China.

[10]College of Life Science, Liaoning Normal University, Dalian, China.

[11]College of Animal Science and Technology, Agricultural University of Hebei, Baoding, China.

[12]Shanghai Center for Bioinformation Techonology, Shanghai Industrial Technology Institute, Shanghai, China.

[†]These authors contributed equally to this work.

*These authors jointly directed this work.

Correspondence should be addressed to Y.L. (yxli@sibs.ac.cn) or M.C. (mxchu@263.net)

Abstract

Background

Animal domestication has been extensively studied but the process of feralization remains poorly understood.

Results

Here, we performed whole-genome sequencing of 99 sheep and identified a primary genetic divergence between two heterogeneous populations in the Tibetan Plateau, including one semi-feral lineage. Selective sweep and candidate gene analysis revealed the local adaptations of these sheep associated with sensory perception, muscle strength, eating habbit, mating process and aggressive behavior. In particular, a horn-related gene *RXFP2* showed signs of rapid evolution specifically in the semi-feral breeds. A unique haplotype and repressed horn-related-tissue expressions of *RXFP2* were correlated with higher horn length, as well as spiral and horizontally extended horn shape.

Conclusions

Semi-feralization has an extensive impact on diverse phenotypic traits of sheep. By acquiring features similar to those of their wild ancestors, semi-feral sheep were able to re-gain fitness in frequent contact with wild surroundings and rare human interventions. The present study provides a new insight into the evolution of domestic animals when human interventions are no longer dominant.

Key words

Domestic animal - Sheep - Adaptive evolution - Artificial selection - Semi-feralization - Horn

Background

Animal domestication has been widely investigated in order to better understand the phenotypic and genetic changes of animals caused by human activities [1-4]. However, the process in which domestic animals become feral is still poorly understood. Domestication is the process where protection offered by domestic habitat suppresses the original environmental adaptation. Feralization is its reverse: the animals re-start to fit natural life while human artificial selections were no longer dominant [5].

The history of Chinese sheep domestication can be traced back more than 5,000 years according to archeological evidence [6, 7]. The demographic history of Chinese domestic sheep was recently reconstructed based on population genomics, which suggested their origin on the Mongolian Plateau about 5,000 to 7,000 years ago with later dispersal associated with historical movements of nomadic societies [8]. To date, more than 42 local breeds of sheep have been established in China, comprising lineages from three major geographic areas known as northern China, the Tibetan Plateau and the Yunnan-Kweichow Plateau [8, 9]. Sheep in northern China were also documented as Mongolian sheep represented by their distinctive phenotypes related with fat storage (fat-tails or fat-rumps) [10, 11]. Tibetan and Yunnan-Kweichow sheep were split from Mongolian sheep about 4,000 years ago [8].

It has been proven that different climate zones have had an essential impact over the adaptive evolution of the major ovine lineages in China [9]. However, the role of

various husbandry cultures in affecting the phenotypes of modern sheep breeds is not well understood. In fact, the unique domestication history and husbandry system of Tibetan sheep makes it an appropriate evolutionary model for studying animal semi-feralization for several reasons. Firstly, because the Tibetan Plateau is rich in grassland, the local breeds, especially those living on prairies, have been roaming with nomads and fed on natural ranches. Secondly, these sheep were forced to encounter threats from the wild (e.g. Tibetan wolves), because of a sparsely populated and undeveloped environment. Thirdly, unlike in other pastoral areas of China, the breeding of Tibetan sheep was not subject to intense artificial control, such as gender-separating management and selective breeding. In this case, the evolution of these semi-feral populations can provide indications about how domestic animals adapt when artificial pressures are loosened.

To enhance the understanding of animal feralization, we sequenced and analyzed the genomes of 30 sheep from two semi-feral breeds and one domestic breed from the Tibetan Plateau and 69 domestic sheep from other geographic areas. We identified a primary divergence in Tibetan sheep and a set of candidate loci underlying selective sweeps in each Tibetan breed, which is responsible for their distinct phenotypic patterns related with semi-feralization.

Data Description

We selected 30 sheep from three typical Tibetan breeds in the Tibetan Plateau

(PT, Prairie Tibetan sheep; VT, Valley Tibetan sheep; OL, Oula sheep), 59 sheep from six Mongolian breeds across northern China (BY, Bayinbuluke sheep; CB, Cele Black sheep; H, Hu sheep; T, Tan sheep; STH, Small Tail Han sheep; WZ, Wuzhumuqin sheep), as well as 10 Australian Merino sheep (AM) representing a European-originated breed (**Figure 1a**, **Supplementary Table S1-S2**). Among the 10 breeds, PT and OL were two semi-feral populations that did not receive extensive human interventions, while PT, OL, VT and BY were four populations living at high altitude (>3,000m above sea) (**Supplementary Table S2**). The sex ratio was maintained at approximately 1:1 for each breed. We performed whole-genome sequencing (WGS) of the 99 sheep. The coverage depth after genome alignment was approximately six-fold for each individual (**Supplementary Table S3-S4**), resulting in more than 50× coverage depth for each breed.

Analyses

Characterization of the variants

After applying stringent criteria in quality control, we identified a total of 38,090,348 SNPs and 4,348,493 insertions/deletions (indels) in the 99 genomes (**Supplementary Table S5**). The abundance of variants was comparable to those of other domestic animals [4, 12, 13]. Most variants were intergenic or intronic, and only 269,584 SNPs and 5,518 indels were exonic (**Supplementary Table S6-S7**). Our dataset captured >94.0% (26,598,869 SNPs and indels) of the variants in the dbSNP

database build 143, whereas >37.3% (15,839,972 SNPs and indels) of the variants in the 99 sheep genomes were absent from the public collection (**Supplementary Figure S1**). The genome-wide average diversity π of the sheep breeds was estimated to be 2.44-2.84 × 10^{-3}, which was similar as previously reported [9]. In other domestic animals such as pigs and dogs, nucleotide diversity in Tibetan breeds is often higher than in other Chinese breeds [12, 13]. However, our data suggested domesticated sheep in China has an opposite trend: the Tibetan sheep breeds (π = 2.44-2.61 × 10^{-3}, θ = 2.10-2.30 × 10^{-3}) have lower nucleotide diversity than Mongolian (π = 2.69-2.79 × 10^{-3}, θ = 2.36-2.52 × 10^{-3}) and European breeds (π = 2.84 × 10^{-3}, θ = 2.50 × 10^{-3}), which is consistent with the fact that Mongolian sheep diverged earlier than Tibetan sheep from their ancestral lineage [14].

Population genetics of Chinese sheep

To understand the genetic relationships among these local breeds, we constructed a neighbor-joining (NJ) tree based on their pair-wise genetic distances (measured by fixation index F_{ST}) (**Figure 1b**). We also calculated a phylogeny tree based on genomic SNPs to visualize the relationship between individual samples, where a goat genome was used to calibrate the root (**Supplementary Figure S2a**). As expected, the European-originated sheep (AM and Texel) were the first clade separated from the ancestral lineage. That was followed by the Mongolian breeds and finally, the Tibetan breeds. This phylogeny structure is again consistent with the migration trajectory of

sheep, where Eurasian sheep initially migrated onto the Mongolian Plateau and then spread into local areas of China [14]. The three Tibetan sheep breeds formed a monophyletic clade which was robust under bootstrapping tests (**Supplementary Figure S2b**), indicating a common origin of Tibetan sheep from one recent ancestral lineage.

We next performed a principal component analysis (PCA) of 99 sheep based on their genomic variants (**Figure 1c**). Despite the division among Tibetan sheep (TBS), Mongolian sheep (MGS) and European sheep (EUS), a considerable genetic difference was observed between two groups of Tibetan sheep: one cluster consisted of 20 individuals from two semi-feral breeds PT and OL, while another consisted of 10 individuals from domestic breed VT (**Figure 1c**). We further examined the population structure by assuming the number of ancestry K (**Figure 1d, Supplementary Figure S3**). When K = 3, TBS, MGS and EUS were clearly separated, though BY, one breed of MGS, showed a mixture between TBS and MGS. When K = 4, we observed a primary divergence between semi-feral and domestic TBS, in agreement with the PCA result. In addition, analysis by TreeMix [15] confirmed the migration event from VT to BY (**Supplementary Figure S4**). Due to its genetic admixture, BY was treated separately from other MGS breeds during subsequent analysis.

The previous study of native sheep in China has included samples from four Tibetan populations (labeled as ZRK, ZLZ, ZNQ and ZCD) [9]. Here, we provided a supplementary map to summarize their geographical locations and relationship with

Tibetan breeds in the present study (**Supplementary Figure S5**). Briefly, VT, ZRK and ZLZ were located in southern Tibet, while PT, OL, ZNQ and ZCD were in the north. OL was at a relatively distant area from other local breeds. However, as we observed high similarity between OL and PT, it seems that the geographical distance was not the only or even minor determinant of the genetic differences between breeds.

An intriguing phenomenon is that the domestic TBS breed VT seems to show a unique breeding history, represented by its slow linkage disequilibrium (LD) decay and the most positive Tajima's *D* statistics across the genome compared with other breeds (**Supplementary Figure S6**). These statistics suggest that VT has encountered the most severe contraction of population size during localization. These sheep also showed lower genetic diversity ($\pi = 2.44 \times 10^{-3}$) than semi-feral TBS ($\pi = 2.60\text{-}2.61 \times 10^{-3}$), MGS ($\pi = 2.69\text{-}2.79 \times 10^{-3}$) and EUS ($\pi = 2.84 \times 10^{-3}$). Moreover, population ZLZ in the other study was proximate to VT and also exhibited a sign of population bottleneck (evidenced by slow LD decay) [9]. This data suggested the current VT population was derived from a relatively small number of founders from the common ancestor of TBS.

Selective sweeps in semi-feral and domestic sheep

We reasoned that different levels of human intervention might have resulted in distinct evolutionary trajectories of PT, OL and VT. For example, PT and OL raised by nomads were typically free-roaming, while VT were captive, intensively managed

by local farmers for improving productions and efficiencies (**Supplementary Table S2**). PT and OL live in under-developed regions of north Tibet, where human population is sparse (**Supplementary Figure S7**), suggesting less interaction with human society and more threats from predators (e.g. Tibetan wolves). Moreover, VT was subject to moderate selective breeding, while PT and OL received barely any intervention in their mating process (**Supplementary Table S2**)

To identify candidate genes under positive selection in different TBS populations, we performed a selective sweep analysis over the whole genome based on population differentiation (Fixation index F_{ST}) and loss of heterozygosity (heterozygosity $\log_2[H_P$ ratio]) in PT, OL, VT and BY respectively, by comparing them with MGS (**Figure 2**). BY is not a TBS breed, but is included here to identify loci potentially under altitude adaptations (PT, OL: semi-feral group; PT, OL, VT: Tibetan group; PT, OL, VT, BY: high-altitude group) (**Supplementary Table S2**). In total, we identified 1,104, 988, 1,030 and 749 candidate genes in each of the four populations (**Figure 3a, Supplementary Table S8-S11**).

In two semi-feral populations, we observed a consistently strong signal of positive selection on chromosome 10, which harbors a Relaxin/insulin-like family peptide receptor 2 (*RXFP2*) gene (**Figure 2, Supplementary Table S8-S9**). *RXFP2* is a well-known gene related with sheep horn phenotypes, and is often characterized as a target of natural and sexual selection in wild and feral populations [16, 17]. Since free mating is one of the typical features of wild and feral populations, and is often replaced with selective breeding in domestic lines, *RXFP2* potentially serves as the

genetic marker of "wildness" in sheep, which confers their essential sexual weaponry during competitions to reproduce.

In addition to *RXFP2*, we also characterized a number of PT and OL candidate genes with a significantly high window F_{ST} and H_P ratio, that are functionally plausible for adaptation in the wild (**Figure 3b**). For example, these genes include: (1) *MITF*, *MSRB3*, *SLC26A4* associated with hearing [18-25]; (2) *SMNDC1*, *SOX6* involved in muscle development [26, 27]; and (3) *PRD-SPRRII* regulating rumen development [28]. Their signals of selective sweep in four populations were in rough agreement with different extents of human intervention, where high F_{ST} and low H_P values were often restricted to PT and OL and were absent in VT and BY (**Figure 3b**). Moreover, it is worth noting that some of these candidates are known to mediate diverse phenotypes, like *MITF* variants also contribute to coat color patterns [29-31]. In such cases, additional information like phenotypic data will be necessary to define the real outcome of positive selection.

Then, we performed a Gene Ontology (GO) enrichment analysis of the gene sets in sweep regions of the four populations PT, OL, VT and BY (**Supplementary Table S13-S16**). We also analyzed enriched GO terms in overlapping gene sets representing the combinations of semi-feral sheep (PT and OL, gene number = 231), and Tibetan sheep (PT, OL and VT, gene number = 62) (**Supplementary Table S17-S18**). The results showed a number of feralization-related functional terms over-represented in PT, OL or their overlapping candidate genes (**Figure 3c**, **Supplementary Table S19**). For example, a set of key terms was found related with the process of mating and

reproduction, such as androgen receptors (GO:0050681, GO:0030521), the maternal process in female pregnancy (GO:0060135), and hormone metabolisms (GO:0046887, GO:0032353). Categories associated with muscle function, such as striated muscle development (GO:0014706), muscle cell apoptosis (GO:0010656, GO:0010657) and muscle adaptation (GO:0043500, GO:0043502), were also characterized. Moreover, other enriched functions include aggressive behaviors (GO:0002118), defense response (GO:0031347), digestive system development (GO:0055123, GO:0048565), as well as a number of GO clusters in sensory organ development (GO:0001754, GO:0042461, GO:0042462, GO:0046530, GO:0048592, GO:0048593, GO:0021772). All functional terms mentioned above had a significant enrichment score (P value $<$ 0.05) after considering multiple testing errors (by the Benjamini-Hochberg approach). Taken together, these findings suggested that two semi-feral lineages of TBS have undergone diverse processes of semi-feralization in response to the natural environment and reduced human protections. The adaptation potentially brought them with advantages through free mating, improved muscle strength and food digestion abilities, and promoted aggressiveness, defensive responses and sensory perceptions in order to survive attacks from predators.

On a different note, our data also corroborated the previous findings of TBS on their adaptation to the hypoxic environment. By comparing our candidate gene list with those from two other studies of TBS [9, 32], we found *NF1* as a consistent signal in response to altitude adaptation identified among independent approaches (**Supplementary Table S12**). Our results indicated that *NF1* was under positive

selection in all four high-altitude populations (**Figure 3b**). Nevertheless, it is intriguing that other possible candidate genes of altitude adaptations, such as *PTEN* and *PINK1*, are more often exhibiting lineage-specific signals (**Figure 3b**). Since the response to hypoxia is a well-known cellular process of polygenic basis, it is possible that the trajectories of hypoxia adaptation are highly heterogeneous among independent populations (an existing example is the Tibetan chicken [33]). Thus, it may explain the non-uniform distribution of hypoxia-associated sweeps among the four high-altitude populations.

A horn-related locus *RXFP2* underlies positive selection in semi-feral sheep

We next investigated and validated the strongest adaptive signature of semi-feralization in PT and OL, at chromosome 10 spanning a 60-kb region of *RXFP2* gene (**Figure 2**). The sweep region exhibits excess of population differentiation ($F_{ST|PTvsMGS}$ = 0.736, $F_{ST|OLvsMGS}$ = 0.736) and a dramatic loss of heterozygosity ($H_{P|PT}/H_{P|MGS}$ = 0.768, $H_{P|OL}/H_{P|MGS}$ = 0.757) in two semi-feral breeds (**Figure 4a, Supplementary Table S8-S9**). Nevertheless, neither of these signals was observed in VT ($F_{ST|VTvsMGS}$ = 0.052, $H_{P|VT}/H_{P|MGS}$ = 0.976), while BY exhibited a moderate increase in genetic differentiation ($F_{ST|BYvsMGS}$ = 0.187) but with no evidence of heterozygosity depletion ($H_{P|VT}/H_{P|MGS}$ = 1.519). The pattern of single nucleotide polymorphisms (SNPs) located in the *RXFP2* gene region revealed a unique haplotype in PT and OL, which was obviously different from those in VT, BY, MGS and EUS

(**Figure 4b**).

Two missense mutations in *RXFP2* (OAR10_29461968: E641K, OAR10_29462010: V627M) were characterized as most significantly differentiated among its protein-altering variants (**Supplementary Table S20-S21**). Both of the two sites are highly conserved among vertebrate species and were mutated in PT and OL compared with other sheep (**Figure 4c**). To confirm their haplotypic distributions among 10 breeds, we examined the genotypes of 1,155 independent individuals at these two SNP sites. According to the result, the distribution of both SNPs were consistent with our whole genome sequencing (WGS) data, where the haplotype 1 consisting of "OAR10_29461968:T + OAR10_29462010:T" (*RXFP2*: M627 and K641) were mostly found in PT and OL, while the haplotype 2 consisting of "OAR10_29461968:C + OAR10_29462010:C" (*RXFP2*: V627 and E641) were predominant in VT, BY, MGS and EUS (**Figure 4d**, **Supplementary Figure S8**).

We also compared the *RXFP2* haplotypes observed in our 99 sheep with those reported in the wild bighorn sheep population, where *RXFP2* was selected for intra-sexual competitions [17]. The result showed no obvious similarity between these two haplotypes (**Supplementary Figure S9-S10**), suggesting different directions of adaptive evolution at *RXFP2* locus between the semi-feral TBS and the wild bighorn sheep.

Taken together, our results indicated that PT and OL sheep have formed a unique haplotype at *RXFP2* locus under the effect of positive selection.

RXFP2 haplotype controls horn size and shape

A common feature of the semi-feral populations PT and OL is that they often have strong, long horns. These horns typically formed a spiral and horizontal extension (SHE-type) (**Figure 5a**). The SHE horns are clearly different from the horns of European wild sheep (*Ovis orientalis*, *Ovis musimon*), which are regarded as the ancestor of modern domestic sheep in China [14] (**Supplementary Figure S8**). In contrast, horns of VT, BY, MGS and EUS are either polled or curled tightly close to the face (TCF-type) (**Figure 5a**). To figure out whether *RXFP2* haplotype directly affected the appearance of horns, we tested their correlation in an independent PT population (n = 182) with heterogeneous horn types. This population consists of 138 SHE-type horned, 16 TCF-type horned, 14 scurred (small and undeveloped horns), 11 polled sheep, as well as three individuals with uncertain horn type (**Supplementary Figure S11**, **Supplementary Table S24**). Regression models were applied to identify potential association between horn phenotypes (horn size, horn shape) and eight *RXFP2*-linked SNPs, which included two protein-altering, two intronic SNPs with high F_{ST} in semi-feral TBS and four previously reported trait-associated SNPs in other sheep populations (**Supplementary Table S25**).

In the 182 PT sheep, we observed strong associations between horn sizes and three SNPs we identified based on F_{ST} (OAR10_29461968, OAR10_29491062, OAR10_29461717), while the four previously reported SNPs showed either minor or

no effect (**Figure 5b**). The highest correlation among all eight SNPs was found at one of the protein-altering SNPs (OAR10_29461968), where each copy of T allele gave rise to about 11.75 cm increase in horn length (P = 4.78×10^{-23}) (**Figure 5c**). By analyzing covariates in the regression model, we confirmed that this correlation was independent from individual age and sex, which potentially affected horn size (regarding covariates, P = 1.75×10^{-27}) (**Supplementary Figure S12**). Furthermore, we identified the same SNP OAR10_29461968, rather than previously reported SNPs, strongly correlated with horn shape (**Figure 5b**). OAR10_29461968:T homozygotes were found to be overrepresented in SHE-type horned sheep relative to TCF-type individuals (P = 2.20×10^{-7}) (**Figure 5d**), in agreement with the shape distribution across different populations. These findings supported that the unique *RXFP2* haplotype we identified was responsible for the horn-phenotype differences between semi-feral and domestic populations in Chinese sheep.

RXFP2 gene expression in sheep horns

Although a solid association has been identified between the haplotype over the *RXFP2* locus and horn phenotypes, it is possible that causal variants might actually affect other flanking genes in respect of regulatory region alterations. To corroborate the functional relevant gene for horns of TBS, we studied the expression patterns among different PT sheep tissues of all functional genes (*RXFP2*, *B3GLCT*, *FRY*, LOC101110773, LOC106991357) annotated within a ~1Mb region encompassing

RXFP2. The region was comprised of multiple LD blocks (**Supplementary S13**), which covered potential hitchhiking variants associated with the sweep. Interestingly, among all five studied genes, only *RXFP2* exhibited a pattern of tissue-specific expression in soft horn and horn periosteum (**Figure 6a, Supplementary Figure S14**). This tissue-expression pattern was also confirmed in samples from Sonid sheep (**Supplementary Figure S15**).

We next compared the gene expression in the soft-horn tissues of SHE-type, TCF-type and scurred PT sheep. Despite obvious individual variations, a relatively lower expression of *RXFP2* was found in SHE than in TCF ($P < 0.001$) and scurred samples ($P < 0.001$) (**Figure 6b**). Moreover, *RXFP2* expression was negatively correlated with horn size (Pearson's $r = -0.76$, $P = 0.002$) (**Figure 6c**). No obvious correlations between flanking gene expressions and horn phenotypes were observed (**Supplementary Figure S16-S17**). Furthermore, the *RXFP2* protein levels in soft-horn tissues were examined by western blotting, which revealed a consistent reduction of translation product in SHE-type horns (**Figure 6d**), in agreement with the mRNA expressions.

Discussion

Feralization is the process where domestic animals go back to the wild. Although its reverse – domestication – has been extensively studied [1-4], the genetic basis of feralization remains largely unsolved. In the present study, we performed a comprehensive survey of genetic diversity in Tibetan sheep. These animals are separated into various local breeds, within which a contrast of domestic and semi-feral status exists. Analysis of selective sweeps in two semi-feral populations compared with domestic lineages has revealed a critical role of semi-feralization in diversity patterns, mechanically related with mating, muscle strength, eating habit, aggressive behaviors, defensive responses and sensory perception. In particular, the semi-feral sheep have developed a unique horn phenotype as plausible adaptation to the reduced human intervention and increasing natural and sexual selection.

Horns are crucial for the survival of wild sheep because: (1) male individuals with strong horns show advantages in competition to reproduce; (2) aggressive horns are essential weapons against carnivorous enemies. In most domestic lines, horns become vestigial, because traits ensuring fitness in natural life are becoming useless under artificial breeding. However, domesticated Tibetan sheep would be an exception, as we found a special SHE-type horn, mediated by *RXFP2* gene, bringing about individual advantages in two semi-feral populations PT and OL. Firstly, PT and OL represented by their SHE-type horns, contained a special *RXFP2* haplotype with strong signals of selective sweeps compared with VT and MGS. Secondly, an association study within a PT population suggested that the *RXFP2* haplotype was

significantly associated with horn size and shape. Thirdly, gene expression analysis in 13 tissues of PT sheep demonstrated that *RXFP2* was the only gene specifically expressed in horn-related tissues and exhibiting decreased expression in SHE-type horned individuals. Unlike in other pastoral areas, the breeding of Tibetan sheep is less affected by human intervention, and most importantly, the mating is relatively random (e.g. opposite sexes are kept separately for most MGS, but not TBS) (**Supplementary Table S2**). PT and OL live in an area with sparse human populations (**Supplementary Figure S7**), suggesting little artificial impact and more threats from the wild (e.g. more wolves were observed in sparsely populated regions of Tibet). Therefore, large and aggressive horns will bring about advantages for PT and OL, just as in wild and other feral populations [17].

An interesting phenomenon is that SHE-type horns (with a spiral and twisted shape) seem to also exist in other sheep breeds out of China, such as the Hungarian Racka sheep (**Supplementary Figure S18**). It is not clear whether there was genetic introgressions related with this phenotype from other ovine lineages into TBS, or vice versa. It is also possible that the *RXFP2* genotype related with SHE horns was newly derived in TBS. These possibilities can be tested in the future when genotypic data from more sheep breeds is generated.

RXFP2 was a well-known genetic determinant of horn phenotype in sheep. This locus was shown to be correlated with quantitative and discrete traits of horns in wild and feral populations [16, 17, 34]. For domestic sheep, SNPs within or around *RXFP2* were predictive for polledness [35-37]. Although the contribtion of *RXFP2* to horn

phenotypes have been extensively studied [17, 38, 39], little is known about the mechanism accounting for the various outcomes of sheep horns. Our study confirmed that *RXFP2* was the functional gene responsible for the special horn shape observed in semi-feral breeds PT and OL. The expression patterns of *RXFP2* among horn types, which we identified, provided novel evidence to understand the genetic basis underlying growth of horns, as well as polledness. As in cattle, understanding polledness of sheep is crucial because it improves the welfare of animals and protects their handlers [40]. Additional efforts are required to clarify the role of *RXFP2* in the development of diverse sheep horns.

In conclusion, the present study revealed a Tibetan sheep sub-population that has been subject to alterations in genomic loci related with its semi-feralization. These sheep have undergone rapid evolution across the *RXFP2* gene to acquire strong and weapons-grade horns, as a consequence of sexual selection and reduced human intervention. Our study highlighted the importance of human activities in adaptive evolution of domestic animals and provided a novel insight into their processes of semi-feralization.

Methods

Sample collection and sequencing

A total of 89 Chinese sheep from nine diverse breeds, as well as 10 Australian Merino sheep from Australia were sequenced in the present study (**Supplementary Table S1-S2**). For each sheep, genomic DNA was extracted from 200 µl of peripheral venous blood using the QIAamp DNA Blood Mini Kit (Qiagen, Germany). The quality and integrity of the DNA was assessed using the A260/280 ratio and agarose gel electrophoresis. For sequencing library preparation, the genomic DNA was sheared to fragments of 300-400 bp and subsequently end-repaired, 'A'-tailed and ligated to Illumina sequencing adapters. The ligated products with sizes of 400-500 bp were selected on 2% agarose gels and subsequently amplified by ligation mediated PCR (LM-PCR). The libraries were sequenced on an Illumina HiSeq 2500 sequencer in 2 × 100 bp paired-end mode and controlled using Illumina HiSeq Control Software.

Variant calling

The raw reads were processed using two steps of quality control (QC): (1) reads with adapter contamination were removed; and (2) reads with more than 10% ambiguous bases were excluded. Only paired reads were preserved after QC. The filtered reads were subsequently mapped to the sheep reference genome assembly oviAri3 (ftp://ftp.ncbi.nlm.nih.gov/genomes/all/GCA_000298735.1_Oar_v3.1/GCA_0002987

35.1_Oar_v3.1_genomic.fna.gz) using BWA [41] (version 0.7.12) for all individuals separately. PCR duplicates were removed using PICARD (available at http://broadinstitute.github.io/picard/, version 1.135). Indels were realigned using GATK [42] (version 3.2-2). SNPs and indels were called using SAMtools [41] (version 1.2) after pooling samples from the same breed. After SNP calling, the variants were filtered using vcfutil.pl varFilter, with a "-d 20 -D 100" parameter to remove low-quality SNPs and indels. Targeted sequencing (Sanger) of five random genomic regions were performed, and the results were utilized to estimate the false positive (FPR) and false negative rate (FNR) of SNP calling step (**Supplementary Table S26-S27**). After filtering, the variants were annotated using snpEff [43] (version 4.0e) according to the NCBI annotation (ftp://ftp.ncbi.nlm.nih.gov/genomes/Ovis_aries/GFF/ref_Oar_v3.1_scaffolds.gff3.gz).

Population genetics analysis

Pair-wise genetic distances were measured by the number of allele differences for genomic SNP sites. The neighbor-joining tree was calculated based on the distance matrix using PHYLIP [44] (version 3.69). To place a root for the phylogeny tree, we aligned the goat genome sequence (ftp://ftp.ncbi.nlm.nih.gov/genomes/all/GCA_000317765.1_CHIR_1.0/GCA_000317765.1_CHIR_1.0_genomic.fna.gz) with the sheep reference genome sequence using LASTZ [45] (version 1.02) and used the homologous sites of goat to determine the

ancestral alleles for each SNP. Only biallelic autosomal SNPs were used to calculate the distance matrix. PCA was performed using EIGENSOFT [46, 47] (version 6.0.1), and population structures were inferred using FRAPPE software [48] (version 1.1). Both the PCA and population structures were calculated based on autosomal SNPs after removing highly correlated SNP pairs using PLINK [49] (version 1.07) with the "-indep-pairwise 50 5 0.2" parameter. Migration events among sheep breeds were estimated using TreeMix [15] with migration number m = 0-5. Statistics including π (pair-wise nucleotide differences), θ (number of segregating sites), SNP densities and Tajima's D, were calculated using VCFtools [50] (v0.1.12b). The linkage disequilibrium r^2 was calculated using Haploview [51] based on 500,000 SNPs randomly selected from the genome. The parameters were set as "--missingCutoff 0.2 --dprime --minMAF 0.1". The SNP pairs were grouped according to the physical distances between them. The mean r^2 was adopted to represent the average LD for each group (e.g., 0~1 kb).

Selective sweep analysis

Selective sweeps across the sheep genome in four populations PT, OL, VT and BY were detected by comparison with MGS, based on fixation index F_{ST} and heterozygosity $\log_2(H_P$ ratio) over a 30-kb sliding window with a step of 15 kb. F_{ST} distances between population were calculated using the Bio::PopGen::PopStats package in BioPerl [52]. The pooled heterozygosity H_P for population was calculated

using the formula $H= 2\sum p\sum q/(\sum p+\sum q)^2$, where $\sum p$ represents the sum of the major allele frequencies of all SNP sites in the window and $\sum q$ represents sum of the minor allele frequencies [4]. The $\log_2(H_P$ ratio) between population A and B was calculated as $\log_2(H_{P|B}/H_{P|A})$, which reflected the loss of heterozygosity in A relative to B. 46 windows were excluded because of extremely small variant numbers (< 50 variants) (**Supplementary Table S28**). We considered the windows with top 5% values as the significance threshold for single statistic (e.g. $F_{ST|A\text{ vs. }B} > F_{ST|5\%}$, where $F_{ST|5\%}$ denotes the top 5% threshold of $F_{ST|A\text{ vs. }B}$). A 30-kb region was defined as a selective sweep in population A if it had both $F_{ST|A\text{ vs. }MGS}$ and $\log_2(H_{P|MGS}/H_{P|A})$ over the threshold.

All annotated genes overlapped with sweep windows or their flanking windows (15-kb up- and down-stream the sweep region) were defined as candidate genes. Furthermore, the cross-population extended haplotype homozygosity (XP-EHH) [49] was estimated between PT vs. MGS, OL vs. MGS, VT vs. MGS and BY vs. MGS for candidate sweep region, based on haplotype data phased by fastPHASE [53]. GO functional enrichment analysis of the candidate genes was performed using ClueGO [54], in which the P values were corrected using the Benjamini-Hochberg approach (**Supplementary Table S13-18**). Protein-altering mutations were extracted from selective sweep windows to identify potential functional variants (**Supplementary Table S20-23**).

Validation of SNP genotypes in large population

We collected 1,155 additional venous jugular blood samples from sheep of 10 different breeds, including 100 AM sheep, 100 PT sheep, 98 VT sheep, 87 OL sheep, 100 BY sheep, 80 CB sheep, 100 H sheep, 100 T sheep, 100 WZ sheep and 290 STH sheep. Genomic DNA was extracted using the phenol-chloroform method and dissolved in TE buffer (10 mM Tris-HCl [pH 8.0] and 1 mM EDTA [pH 8.0]). To validate the allele frequency of the two differentiated protein altering SNPs in *RXFP2* (**Supplementary Table S20-21**), we performed a multiplex screening assay (SNaPshot) [55] on these 1,155 individuals. We designed amplification and SNaPshot Single-base extension primers **(Supplementary Table S29)**. Genotyping was performed using the SNaPshot™ Multiplex Kit (ABI) according to the manufacturer's instructions and analyzed using the ABI Genetic Analyzer 3730XL.

Association study between the *RXFP2* genotype and horn phenotypes

Nine SNPs within or near *RXFP2* locus (**Supplementary Table S25**) were genotype in 182 PT sheep with five horn types: polled (0 cm), scurred (0-12 cm), TCF-type (>12 cm, tightly close to the face), SHE-type (>12 cm, spiral and horizontally extended) and uncertain-type (>12 cm, uncertain shape) (**Supplementary Table S24**). One out of the nine SNPs was ignored because no variations were observed among those sheep. Correlations between SNP genotypes and horn phenotypes (horn size, horn shape) were estimated using linear or logistic regressions (performed with in-house R language scripts), depending on the variable type of

outcome. Three different genetic models (recessive, additive and dominant) were applied for each pair of association test. Confounding effect of individual age and sex were tested by considering them as covariates in the model.

Gene expression analysis of *RXFP2* and its flanking genes

Tissue expression levels of the five genes located within the 1Mb region encompassing *RXFP2* locus, including *RXFP2*, *B3GLCT*, *FRY*, LOC101110773 (*EF1A1L*) and LOC106991357 (ncRNA) were examined by RT-PCR (**Supplementary Figure S16-S17**). Primer sequences were shown in **Supplementary Table S29**. We studied 13 tissues of TBS and 21 tissues of Sonid sheep. For each tissue type, equal volume of cDNA from six individuals (two individuals from each horn type) were mixed as pooled cDNA samples. RT-PCR reactions were carried out in 50 μl volume including Taq DNA polymerase(5U/μl) (TaKaRa, Dalian, China) 0.25 μl, 10×PCR Buffer(+$MgCl_2$) 5μl, 10mM dNTPs (2.5mM each) 4μl , each primer (10 μM) 1 μl, cDNA 1μl, ddH_2O 37.75 μl. Amplification conditions were set as: initial denaturation at 95°C for 5 min, followed by 33 cycles of denaturation at 95°C for 30 s, annealing for 20 s at appropriate temperatures, extension at 72°C for 10 s; with a final extension at 72°C for 2 min on Mastercycler 5333 (Eppendorf AG, Hamburg, Germany). The PCR product mixture with 5μl loading buffer (6×), and loaded 5μl into 1% sepharose gel. After 15min 180mA electrophoresis, take a picture under Biorad GelDoc XR System (Bio-rad, USA).

Expressions of the five genes were measured by real-time PCR in 13 PT sheep soft-horn samples with different horn types. Four to five biological replicates were selected from different individuals of same horn type (four SHE-type, four TCF-type, and five scurred). For all five genes and internal control, real-time PCR was performed three times in one sample as technical replicates, and the average gene expressions of three replicates were calculated. Real-time PCR amplification was performed in a 20-μl of reaction mixture containing 2 μl of cDNA, 0.4 μl of each forward and reverse primer (10 μM), 0.4 μl of ROX Reference Dye II (50×), 10 μl of SYBR Green Real-time PCR Master Mix (2×), and 6.8 μl of ddH_2O. The reaction without template was treated as blank control. PCR amplification was performed in triplicate wells using the following conditions: 95ºC for 30 s, followed by 40 cycles of 95ºC for 5 s and 60ºC for 34 s. The melting curve was analyzed after amplification. The peak Tm on the dissociation curve was used to determine the specificity of PCR amplification. Standard curves of these genes were also constructed. *β*-actin expressions were used as internal control among samples. Relative expression levels of 5 genes were calculated based on the expression of *RXFP2* in the SHE-type soft horn (its expression was defined as 1.0). The $2^{-\Delta\Delta Ct}$ method was used to process the real-time PCR results [56].

Protein extracts from soft-horn tissues were prepared by complete homogenization of tissues in an immunoprecipitation buffer (Beyotime, CA) according to the manufacturer's instructions. Equal amounts of protein extracts were mixed with sample buffer and then separated on 10% SDS-PAGE gels (60 μg/lane).

Details of the western blotting process were described previously [57]. Rabbit Anti-GPR106 antibody (BIOSS, Beijing, China), polyclonal rabbit anti-mouse *β*-actin antibody (Abcam, US) and goat anti-rabbit IgG, HRP (Santa Clara, CA, USA) were used.

Availability of supporting data

Raw sequence data has been submitted to the NCBI Sequence Read Archive (SRA; http://www.ncbi.nlm.nih.gov/sra) under accession number SRP066883. Genotypic data of 99 individuals has been submitted to the European Variation Archive (EVA; https://www.ebi.ac.uk/eva/ https://www.ebi.ac.uk/eva/) under accession number ERZ480291 (Project ID: PRJEB23437).

Declarations

List of abbreviations

TBS, Tibetan sheep; MGS, Mongolian sheep; EUS, European sheep; PT, Prairie Tibetan sheep; VT, Valley Tibetan sheep; OL, Oula sheep; BY, Bayinbuluke sheep; CB, Cele Black sheep; H, Hu sheep; T, Tan sheep; STH, Small Tail Han sheep; WZ, Wuzhumuqin sheep; AM, Australian Merino sheep; WGS, whole-genome sequencing; indel, insertion and deletion; NJ, neighbor-joining; PCA, principal component analysis; LD, linkage disequilibrium; GO, gene ontology; SNP, single nucleotide polymorphism; SHE, spirally and horizontally extended; TCF, tightly close to the face.

Consent for publication

Not applicable.

Ethic approval

All experimental procedures involving animals were approved by the Chinese Ministry of Agriculture, the animal care and use committee at the institution where the experiments were performed.

Competing interests

The authors declared no competing interests.

Fundings

This work was supported by the Agricultural Science and Technology Innovation Program of China (ASTIP-IAS13), the Earmarked Fund for China Agriculture Research System (CARS-39), the National Key Technology Support Program (2013BAI101B09), the National Natural Science Foundation of China (31472078 and 31402041), the National Key Scientific Instrument and Equipment Development Project (2012YQ03026108), the National Basic Research Program of China (2011CB910204, 2011CB510102), the Youth Innovation Promotion Association CAS (2017325) and the Genetically Modified Organisms Breeding Major Program of China (2016ZX08009-003-006 and 2016ZX08010-005-003), by Major Science and Technology Program of Inner Mongolia Autonomous Region of China.

Authors contribution

YX.L., MX.C. designed and supervised the project. ZY.P., QY.L. collected and generated the data. Z.X., DK.W. performed formal analysis. SD.L., Z.W., BP.M. performed bioinformatics analysis. LY.Y., RC.W., YQ.Z. supported data analysis. WP.H., XY.W., XX. H., G.H., J.Y., C.L., M.J., YJ.Z. provided samples. ZY.P., XY.H., XF.G., BM.L., XY.L., XH.C., XL.D., Q.X., HC.S., FK.L. performed validations. ZK.Z., GH.D., SJ.L. supportively supervised the project. SD.L., ZY.P. drafted the original manuscript. YX.L., MX.C., Z.W., QY.L., ZK.Z., R.D. edited the manuscript. All authors reviewed the final version of manuscript.

Acknowledgements

The authors thank Ori-Gene Technology Co., Ltd. Beijing, China, for their contributions in sample preparations.

References

1. Frantz LAF, Schraiber JG, Madsen O, Megens HJ, Cagan A, Bosse M, et al. Evidence of long-term gene flow and selection during domestication from analyses of Eurasian wild and domestic pig genomes. Nat Genet. 2015;47 10:1141-+.

2. Axelsson E, Ratnakumar A, Arendt ML, Maqbool K, Webster MT, Perloski M, et al. The genomic signature of dog domestication reveals adaptation to a starch-rich diet. Nature. 2013;495 7441:360-4.

3. Carneiro M, Rubin CJ, Di Palma F, Albert FW, Alfoldi J, Barrio AM, et al. Rabbit genome analysis reveals a polygenic basis for phenotypic change during domestication. Science. 2014;345 6200:1074-9.

4. Rubin CJ, Zody MC, Eriksson J, Meadows JR, Sherwood E, Webster MT, et al. Whole-genome resequencing reveals loci under selection during chicken domestication. Nature. 2010;464 7288:587-91. doi:10.1038/nature08832.

5. Callaway E. When Chickens Go Wild. Nature. 2016;529 7586:270-3.

6. Chen FH, Dong GH, Zhang DJ, Liu XY, Jia X, An CB, et al. Agriculture facilitated permanent human occupation of the Tibetan Plateau after 3600 B.P. Science. 2015;347 6219:248-50. doi:10.1126/science.1259172.

7. Yang X, Scuderi LA, Wang X, Scuderi LJ, Zhang D, Li H, et al. Groundwater sapping as the cause of irreversible desertification of Hunshandake Sandy Lands, Inner Mongolia, northern China. Proceedings of the National Academy of Sciences of the United States of America. 2015;112 3:702-6. doi:10.1073/pnas.1418090112.

8. Zhao YX, Yang J, Lv FH, Hu XJ, Xie XL, Zhang M, et al. Genomic Reconstruction of the History of Native Sheep Reveals the Peopling Patterns of Nomads and the Expansion of Early Pastoralism in East Asia. Mol Biol Evol. 2017;34 9:2380-95. doi:10.1093/molbev/msx181.

9. Yang J, Li WR, Lv FH, He SG, Tian SL, Peng WF, et al. Whole-genome sequencing of native sheep provides insights into rapid adaptations to extreme environments. Molecular Biology and Evolution. 2016;33:2576-92. doi:10.1093/molbev/msw129.

10. Zhong T, Han JL, Guo J, Zhao QJ, Fu BL, He XH, et al. Genetic diversity of Chinese indigenous sheep breeds inferred from microsatellite markers. Small Ruminant Res. 2010;90 1-3:88-94.

11. Tu YR. The Sheep and Goat Breeds in China. Shanghai Science and Technology Press; 1989. p. 6-19.

12. Ai H, Fang X, Yang B, Huang Z, Chen H, Mao L, et al. Adaptation and possible ancient interspecies introgression in pigs identified by whole-genome sequencing. Nat Genet. 2015;47 3:217-25. doi:10.1038/ng.3199.

13. Gou X, Wang Z, Li N, Qiu F, Xu Z, Yan D, et al. Whole-genome sequencing of six dog breeds from continuous altitudes reveals adaptation to high-altitude hypoxia. Genome

Research. 2014;24 8:1308-15. doi:10.1101/gr.171876.113.

14. Lv FH, Peng WF, Yang J, Zhao YX, Li WR, Liu MJ, et al. Mitogenomic meta-analysis identifies two phases of migration in the history of eastern eurasian sheep. Molecular Biology And Evolution. 2015;32 10:2515-33. doi:10.1093/molbev/msv139.

15. Pickrell JK and Pritchard JK. Inference of population splits and mixtures from genome-wide allele frequency data. PloS Genetics. 2012;8 11:e1002967. doi:10.1371/journal.pgen.1002967.

16. Johnston SE, Gratten J, Berenos C, Pilkington JG, Clutton-Brock TH, Pemberton JM, et al. Life history trade-offs at a single locus maintain sexually selected genetic variation. Nature. 2013;502 7469:93-5. doi:10.1038/nature12489.

17. Kardos M, Luikart G, Bunch R, Dewey S, Edwards W, McWilliam S, et al. Whole-genome resequencing uncovers molecular signatures of natural and sexual selection in wild bighorn sheep. Mol Ecol. 2015;24 22:5616-32. doi:10.1111/mec.13415.

18. Markakis MN, Soedring VE, Dantzer V, Christensen K and Anistoroaei R. Association of MITF gene with hearing and pigmentation phenotype in Hedlund white American mink (Neovison vison). Journal Of Genetics. 2014;93 2:477-81.

19. Chen L, Guo W, Ren L, Yang M, Zhao Y, Guo Z, et al. A de novo silencer causes elimination of MITF-M expression and profound hearing loss in pigs. BMC Biol. 2016;14:52. doi:10.1186/s12915-016-0273-2.

20. Tsukamoto K, Suzuki H, Harada D, Namba A, Abe S and Usami S. Distribution and frequencies of PDS (SLC26A4) mutations in Pendred syndrome and nonsyndromic hearing loss associated with enlarged vestibular aqueduct: a unique spectrum of mutations in Japanese. European Journal Of Human Genetics. 2003;11 12:916-22. doi:10.1038/sj.ejhg.5201073.

21. Shen X, Liu F, Wang Y, Wang H, Ma J, Xia W, et al. Down-regulation of msrb3 and destruction of normal auditory system development through hair cell apoptosis in zebrafish. International Journal Of Developmental Biology. 2015;59 4-6:195-203. doi:10.1387/ijdb.140200md.

22. Ahmed ZM, Yousaf R, Lee BC, Khan SN, Lee S, Lee K, et al. Functional null mutations of MSRB3 encoding methionine sulfoxide reductase are associated with human deafness DFNB74. American Journal Of Human Genetics. 2011;88 1:19-29. doi:10.1016/j.ajhg.2010.11.010.

23. Ni C, Zhang D, Beyer LA, Halsey KE, Fukui H, Raphael Y, et al. Hearing dysfunction in heterozygous Mitf(Mi-wh) /+ mice, a model for Waardenburg syndrome type 2 and Tietz syndrome. Pigment Cell Melanoma Res. 2013;26 1:78-87. doi:10.1111/pcmr.12030.

24. Park HJ, Shaukat S, Liu XZ, Hahn SH, Naz S, Ghosh M, et al. Origins and frequencies of SLC26A4 (PDS) mutations in east and south Asians: global implications for the epidemiology of deafness. Journal Of Medical Genetics. 2003;40 4:242-8.

25. Pryor SP, Madeo AC, Reynolds JC, Sarlis NJ, Arnos KS, Nance WE, et al. SLC26A4/PDS genotype-phenotype correlation in hearing loss with enlargement of the vestibular aqueduct

(EVA): evidence that Pendred syndrome and non-syndromic EVA are distinct clinical and genetic entities. Journal Of Medical Genetics. 2005;42 2:159-65. doi:10.1136/jmg.2004.024208.

26. Mier P and J P-PA. Fungal Smn and Spf30 homologues are mainly present in filamentous fungi and genomes with many introns: Implications for spinal muscular atrophy. Gene. 2012; 491 2:135-41.

27. Talbot K, Miguel-Aliaga I, Mohaghegh P, Ponting CP and Davies KE. Characterization of a gene encoding survival motor neuron (SMN)-related protein, a constituent of the spliceosome complex. Human Molecular Genetics. 1998;7 13:2149-56. doi:ddb265 [pii].

28. Jiang Y, Xie M, Chen W, Talbot R, Maddox JF, Faraut T, et al. The sheep genome illuminates biology of the rumen and lipid metabolism. Science. 2014;344 6188:1168-73.

29. Hayes BJ, Pryce J, Chamberlain AJ, Bowman PJ and Goddard ME. Genetic Architecture of Complex Traits and Accuracy of Genomic Prediction: Coat Colour, Milk-Fat Percentage, and Type in Holstein Cattle as Contrasting Model Traits. Plos Genetics. 2010;6 9.

30. Schmutz SM and Berryere TG. Genes affecting coat colour and pattern in domestic dogs: a review. Anim Genet. 2007;38 6:539-49.

31. Moore KJ. Insight into the Microphthalmia Gene. Trends Genet. 1995;11 11:442-8.

32. Wei C, Wang H, Liu G, Zhao F, Kijas JW, Ma Y, et al. Genome-wide analysis reveals adaptation to high altitudes in Tibetan sheep. Scientific reports. 2016;6:26770. doi:10.1038/srep26770.

33. Wang MS, Li Y, Peng MS, Zhong L, Wang ZJ, Li QY, et al. Genomic Analyses Reveal Potential Independent Adaptation to High Altitude in Tibetan Chickens. Molecular Biology And Evolution. 2015;32 7:1880-9.

34. Johnston SE, McEwan JC, Pickering NK, Kijas JW, Beraldi D, Pilkington JG, et al. Genome-wide association mapping identifies the genetic basis of discrete and quantitative variation in sexual weaponry in a wild sheep population. Mol Ecol. 2011;20 12:2555-66.

35. Dominik S, Henshall JM and Hayes BJ. A single nucleotide polymorphism on chromosome 10 is highly predictive for the polled phenotype in Australian Merino sheep. Anim Genet. 2012;43 4:468-70.

36. Wang XL, Zhou GX, Li Q, Zhao DF and Chen YL. Discovery of SNPs in RXFP2 related to horn types in sheep. Small Ruminant Res. 2014;116 2-3:133-6.

37. Wiedemar N and Drogemuller C. A 1.8-kb insertion in the 3-UTR of RXFP2 is associated with polledness in sheep. Anim Genet. 2015;46 4:457-61.

38. Johnston SE, McEwan JC, Pickering NK, Kijas JW, Beraldi D, Pilkington JG, et al. Genome-wide association mapping identifies the genetic basis of discrete and quantitative variation in sexual weaponry in a wild sheep population. Mol Ecol. 2011;20 12:2555-66. doi:10.1111/j.1365-294X.2011.05076.x.

39. Kijas JW, Lenstra JA, Hayes B, Boitard S, Porto Neto LR, San Cristobal M, et al. Genome-wide analysis of the world's sheep breeds reveals high levels of historic mixture and strong recent selection. PLoS Biol. 2012;10 2:e1001258. doi:10.1371/journal.pbio.1001258.

40. Carlson DF, Lancto CA, Zang B, Kim ES, Walton M, Oldeschulte D, et al. Production of hornless dairy cattle from genome-edited cell lines. Nature Biotechnology. 2016;34 5:479-81. doi:10.1038/nbt.3560.

41. Li H and Durbin R. Fast and accurate short read alignment with Burrows-Wheeler transform. Bioinformatics. 2009;25 14:1754-60.

42. McKenna A, Hanna M, Banks E, Sivachenko A, Cibulskis K, Kernytsky A, et al. The Genome Analysis Toolkit: a MapReduce framework for analyzing next-generation DNA sequencing data. Genome Research. 2010;20 9:1297-303. doi:10.1101/gr.107524.110.

43. Cingolani P, Platts A, Wang LL, Coon M, Nguyen T, Wang L, et al. A program for annotating and predicting the effects of single nucleotide polymorphisms, SnpEff: SNPs in the genome of Drosophila melanogaster strain w(1118); iso-2; iso-3. Fly. 2012;6 2:80-92.

44. Felsenstein J. PHYLIP - Phylogeny Inference Package (Version 3.2). Cladistics. 1989;5: 164-6.

45. Harris RS. *Improved pairwise alignment of genomic DNA.* Improved pairwise alignment of genomic DNA. PhD Thesis, The Pennsylvania State University. PhD Thesis, 2007.

46. Patterson N, Price AL and Reich D. Population structure and eigenanalysis. PloS Genetics. 2006;2 12:2074-93. doi:10.1371/journal.pgen.0020190.

47. Price AL, Patterson NJ, Plenge RM, Weinblatt ME, Shadick NA and Reich D. Principal components analysis corrects for stratification in genome-wide association studies. Nat Genet. 2006;38 8:904-9. doi:10.1038/ng1847.

48. Tang H, Peng J, Wang P and Risch NJ. Estimation of individual admixture: Analytical and study design considerations. Genet Epidemiol. 2005;28 4:289-301. doi:10.1002/gepi.20064.

49. Sabeti PC, Varilly P, Fry B, Lohmueller J, Hostetter E, Cotsapas C, et al. Genome-wide detection and characterization of positive selection in human populations. Nature. 2007;449 7164:913-8. doi:10.1038/nature06250.

50. Danecek P, Auton A, Abecasis G, Albers CA, Banks E, DePristo MA, et al. The variant call format and VCFtools. Bioinformatics. 2011;27 15:2156-8. doi:10.1093/bioinformatics/btr330.

51. Barrett JC, Fry B, Maller J and Daly MJ. Haploview: analysis and visualization of LD and haplotype maps. Bioinformatics. 2005;21 2:263-5. doi:10.1093/bioinformatics/bth457.

52. Stajich JE, Block D, Boulez K, Brenner SE, Chervitz SA, Dagdigian C, et al. The Bioperl toolkit: Perl modules for the life sciences. Genome Research. 2002;12 10:1611-8. doi:10.1101/gr.361602.

53. Scheet P and Stephens M. A fast and flexible statistical model for large-scale population genotype data: applications to inferring missing genotypes and haplotypic phase. Am J Hum

Genet. 2006;78 4:629-44. doi:10.1086/502802.

54. Bindea G, Mlecnik B, Hackl H, Charoentong P, Tosolini M, Kirilovsky A, et al. ClueGO: a Cytoscape plug-in to decipher functionally grouped gene ontology and pathway annotation networks. Bioinformatics. 2009;25 8:1091-3. doi:10.1093/bioinformatics/btp101.

55. Lovly CM, Dahlman KB, Fohn LE, Su Z, Dias-Santagata D, Hicks DJ, et al. Routine multiplex mutational profiling of melanomas enables enrollment in genotype-driven therapeutic trials. PLoS ONE. 2012;7 4:e35309.

56. Livak KJ and Schmittgen TD. Analysis of relative gene expression data using real-time quantitative PCR and the 2(T)(-Delta Delta C) method. Methods. 2001;25 4:402-8.

57. Zhang R, Rao M, Li C, Cao J, Meng Q, Zheng M, et al. Functional recombinant human anti-HAV antibody expressed in milk of transgenic mice. Transgenic Research. 2009;18 3:445-53. doi:10.1007/s11248-008-9241-0.

Figures and legends

Figure 1. Genetic relationships and population structure in Chinese sheep. (**a**) Geographical distribution of the Chinese indigenous sheep breeds (PT, Prairie Tibetan; OL, Oula; VT, Valley Tibetan; BY, Bayinbuluke; WZ, Wuzhumuqin; T, Tan; CB, Cele Black; STH, Small-tailed Han; H, Hu) and a European-originated breed (AM, Australian Merino) sampled in the present study. The background color of the sheep pictures represent their lineages (red: TBS, Tibetan sheep; blue: MGS, Mongolian sheep; green: EUS, European sheep). (**b**) Neighbor-joining tree of the ten breeds based on F_{ST} distances. (**c**) Principal component plot. The first (PC1) and second (PC2) principal components are shown. (**d**) Population structure analysis of 99 sheep, where number of ancestral clusters were set from K = 2-4.

Figure 2. Manhattan plot of genome-wide selective sweep signals (F_{ST} and log-scaled H_P ratio) in four sheep breeds. For each metric, a 30-kb sliding window with a step size of 15kb was applied. F_{ST} distances were calculated between each of the four breed (PT, OL, VT or BY) vs. MGS (WZ, T, STH, H and CB). The log-scaled H_P ratio was calculated as $-\log_2(H_{P|PT, OL, VT \text{ or } BY}/H_{P|MGS})$, a positive value of which suggests reduction of variability in the breed.

Figure 3. Candidate genes associated with selective sweeps in semi-feral sheep. (**a**) A venn plot showing numbers of overlapping candidate genes among four breeds (PT, OL, VT and BY). (**b**) A brief summary of feralization-related adaptation observed in

semi-feral sheep. Affected functional terms were manually summarized based on Gene Ontology (GO) enrichment analysis of the candidate genes, as well as literature mining. Numbers denote the count of candidate genes within each major category. (**c**) Sweep signal metrics for genes selected from feralization-related categories described in **Figure 2b**, as well as three genes associated with hypoxic adaptation.

Figure 4. Selective sweep over the horn-related gene *RXFP2*. (**a**) Statistics plotted over a ~400 kb region surrounding *RXFP2*, including: 1) population differentiation (F_{ST}) between PT, OL, VT and BY vs. MGS; 2) intra-population heterozygosity in PT, OL, VT and BY, calculated as Z-transformed $\log_2(H_{P|PT,\ OL,\ VT\ or\ BY}/H_{P|MGS})$; 3) haplotypic length measured by Z-transformed XP-EHH$_{PT,\ OL,\ VT\ or\ BY\ vs.\ MGS}$. (**b**) Haplotypic distributions among 99 sheep of a local region of *RXFP2* (chromosome 10: 29,400,000-29,550,000 bp). Biallelic SNPs were showed in blue and yellow. (**c**) Alignment of the *RXFP2* protein sequences from 9 vertebrate species. Two protein variants (*RXFP2*: 627 and 641) with top F_{ST} in PT and OL are indicated in red. For 627 PT and OL have the variant allele, whereas for 641 they have the reference allele. The dots in the alignment denote amino acids that are identical with those in PT and OL. (**d**) Distribution of the haplotype frequency of two protein-altering variants (*RXFP2*: 627 and 641) in 1155 sheep. “Haplotype1” corresponds to V627 + E641 (OAR10_29461968:C + OAR10_29462010:C) and “Haplotype2” corresponds to M627 + K641 (OAR10_29461968:T + OAR10_29462010:T).

Figure 5. *RXFP2* haplotype is correlated with horn shape and size. (**a**) Features of

SHE-type and TCF-type horns. (**b**) Association between eight SNPs and horn phenotypes (size and shape) analyzed in 182 PT sheep; after testing all combinations of genetic models and confounding effects (**Supplementary Figure S12**), an additive model (assume *A* as major allele, *a* as minor allele, we have code 2 for *AA*, 1 for *Aa* and 0 for *aa*) was applied for horn size, and a recessive code (1 for *AA*, 0 for *Aa* and *aa*) was applied for horn shape; pair-wise LD between SNP pairs were plotted at the bottom, where numbers represent *D'* statistics. (**c**) Box-plot of individual horn sizes among different OAR_29461968 genotypes; P value was calculated by linear regression based on additive genetic model, and the fitting line was showed in red. (**d**) Distribution of OAR10_29461968 genotypes among PT sheep with different horn shapes.

Figure 6. Gene expression patterns of *RXFP2*. (**a**) Expression of *RXFP2* and *β-actin* in 13 tissue samples from PT sheep: 1, heart; 2, liver; 3, spleen; 4, lung; 5, kidney; 6, muscle; 7, brain; 8, ovary; 9, corpus uteri; 10, adipose; 11, thyroid; 12, soft horn; 13, horn periosteum. (**b**) Expression pattern of *RXFP2* in SHE-type, TCF-type, scurred soft-horn tissues examined by RT-PCR (left) and real-time PCR (right); error bars denotes S.D. of the mean; groups with significant differences (*: $P < 0.05$; **: $P < 0.001$) were indicated. (**c**) Scatter plot on *RXFP2* expression and horn size; the fitting line of linear regression was showed in blue. (**d**) Western blot analysis of soft-horn tissues with different horn types, using antibodies of *RXFP2* and *β*-actin.

Figure 1

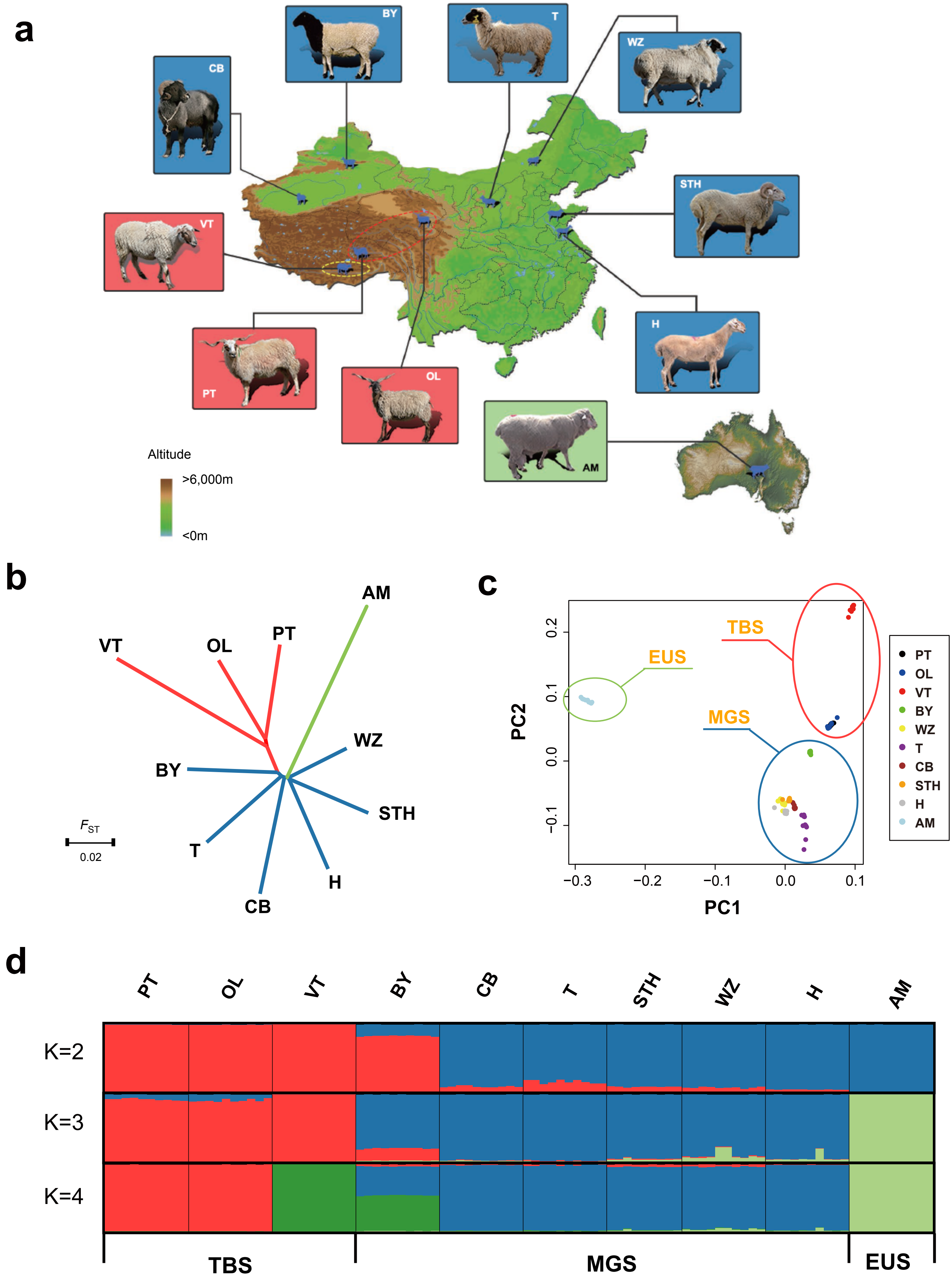
a
BY
T
CB
WZ
STH
VT
PT
OL
H
AM
Altitude
>6,000m
<0m
b
VT
OL
PT
AM
WZ
BY
STH
T
CB
H
F_{ST}
0.02
c
TBS
EUS
MGS
PC2
PC1
0.2
0.1
0.0
−0.1
−0.3
−0.2
−0.1
0.0
0.1
PT
OL
VT
BY
WZ
T
CB
STH
H
AM
d
PT
OL
VT
BY
CB
T
STH
WZ
H
AM
K=2
K=3
K=4
TBS
MGS
EUS

Click here to download Figure Fig.2.revised.pdf

Figure 2

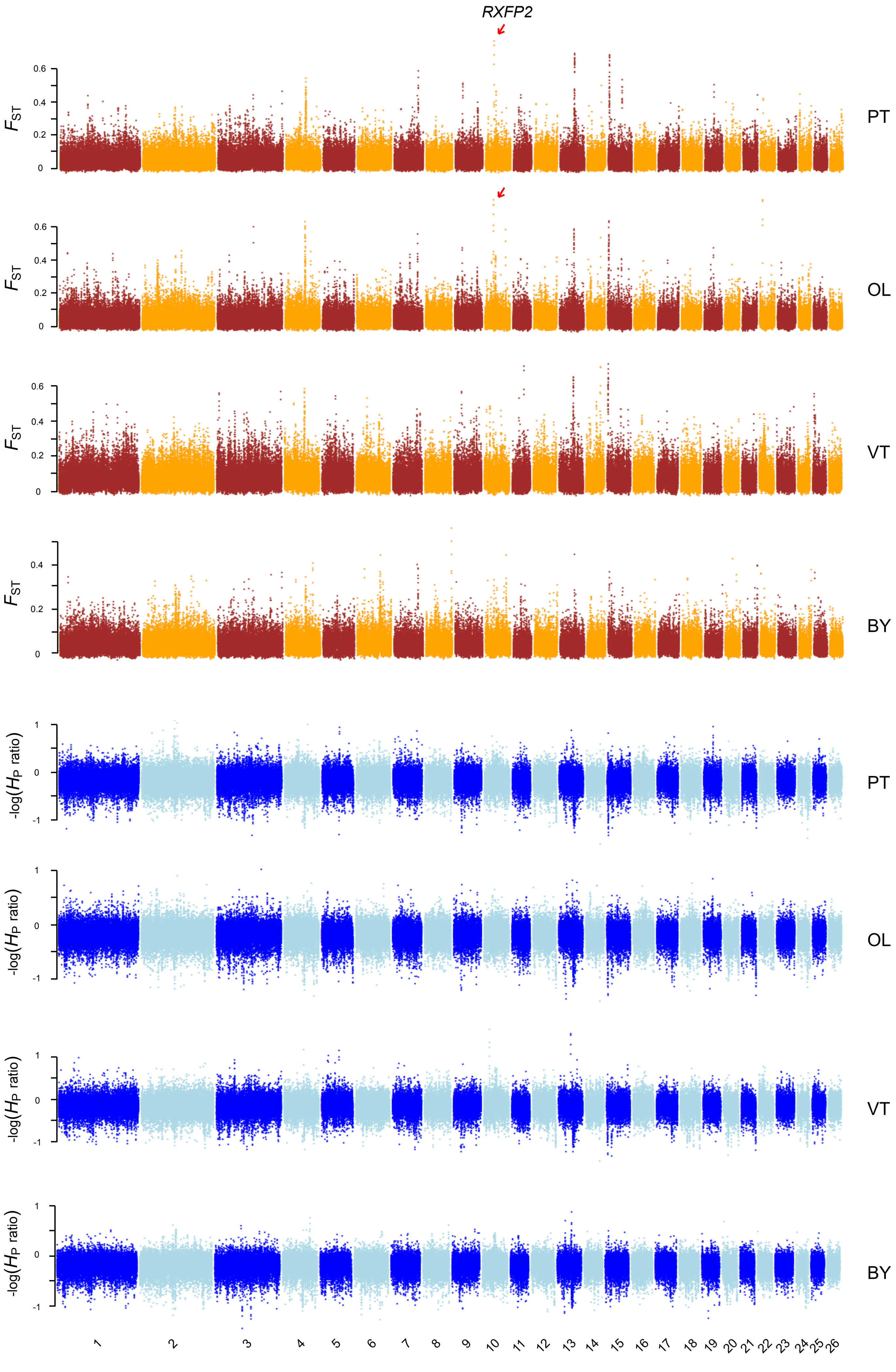

Click here to download Figure Fig.3.revised.pdf

Figure 3

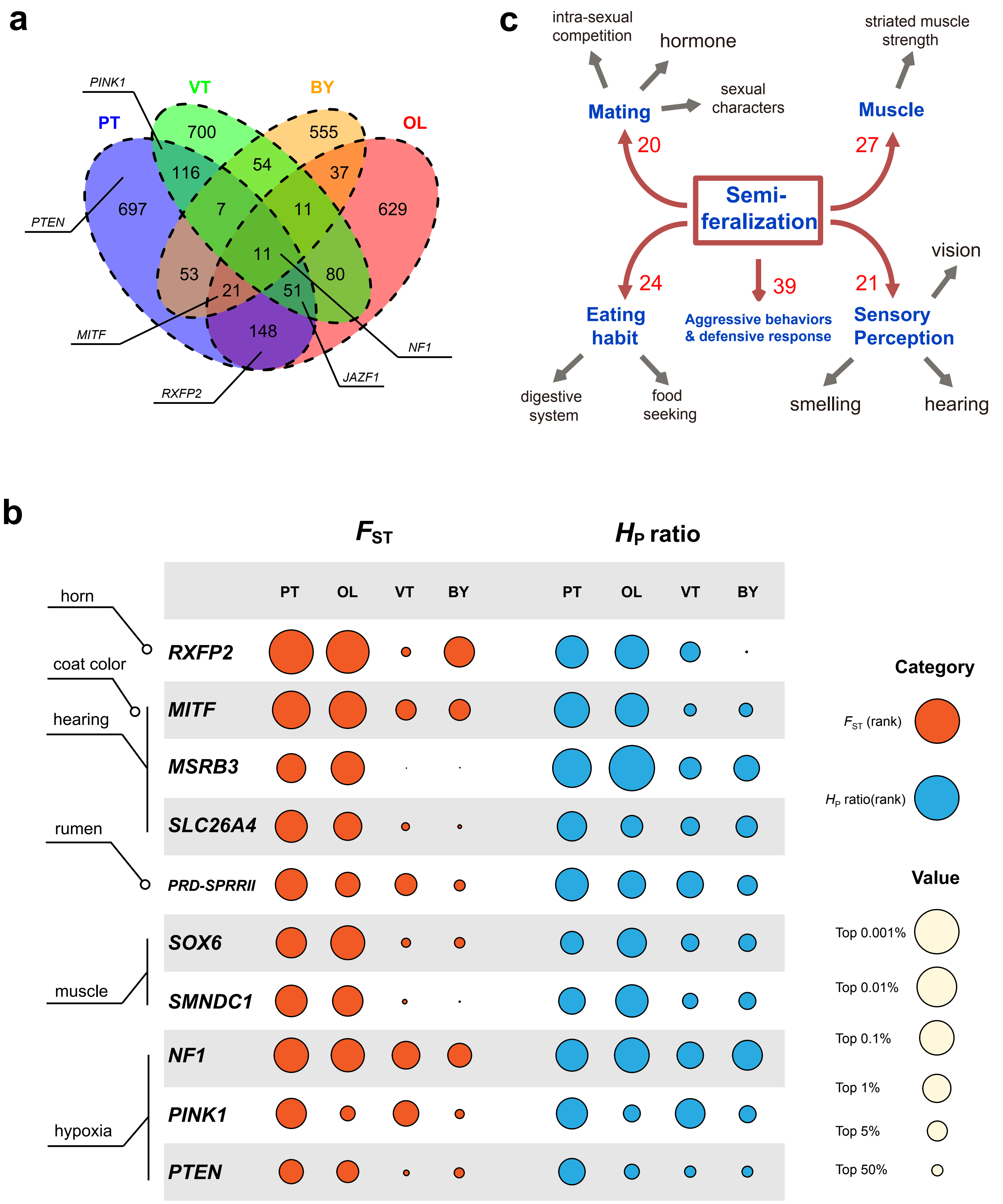

Revised Figure 4

Click here to download Figure Fig.4.revised.pdf

Figure 4

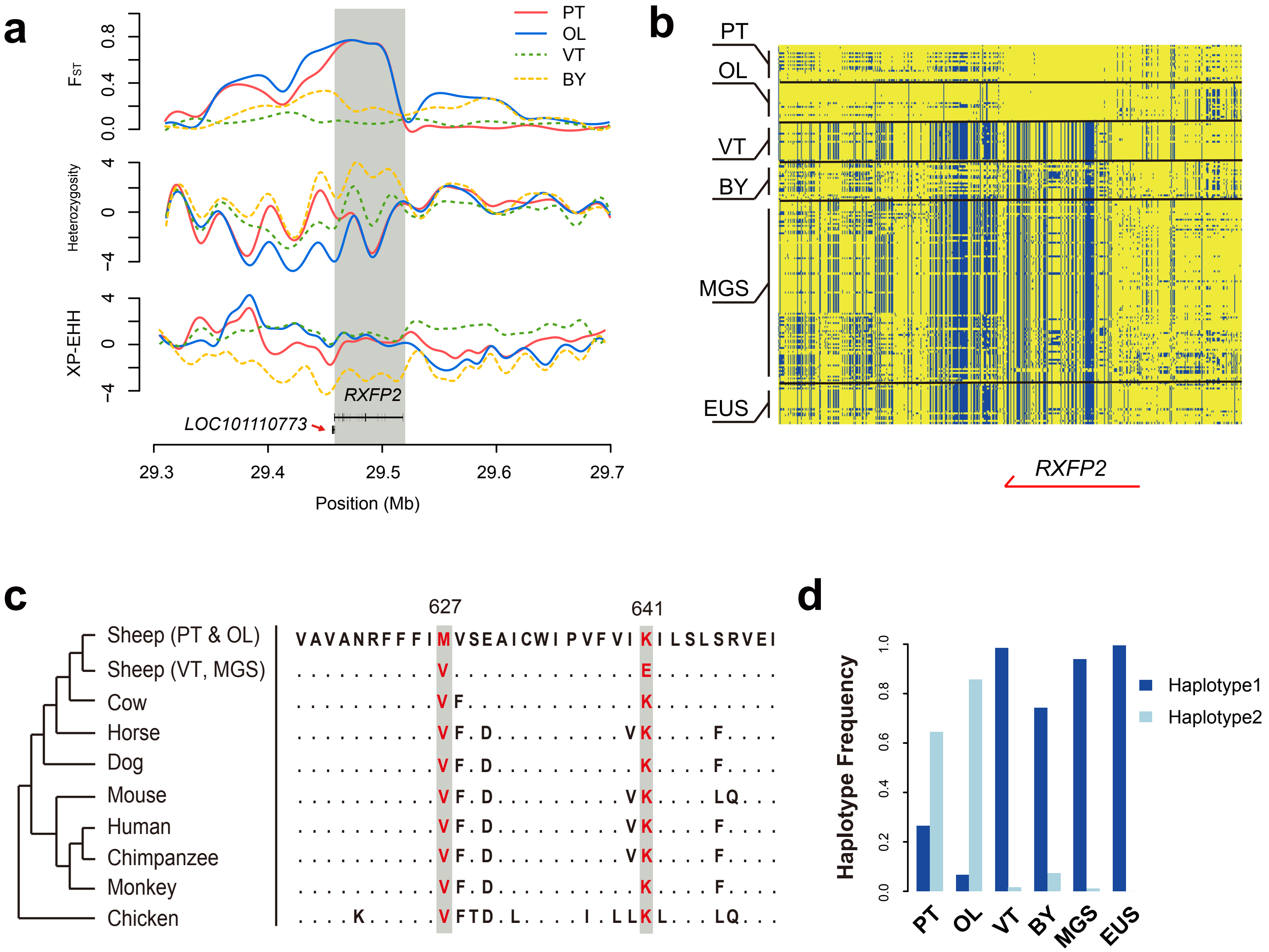

 Click here to download Figure Fig.5.revised.pdf

Figure 5

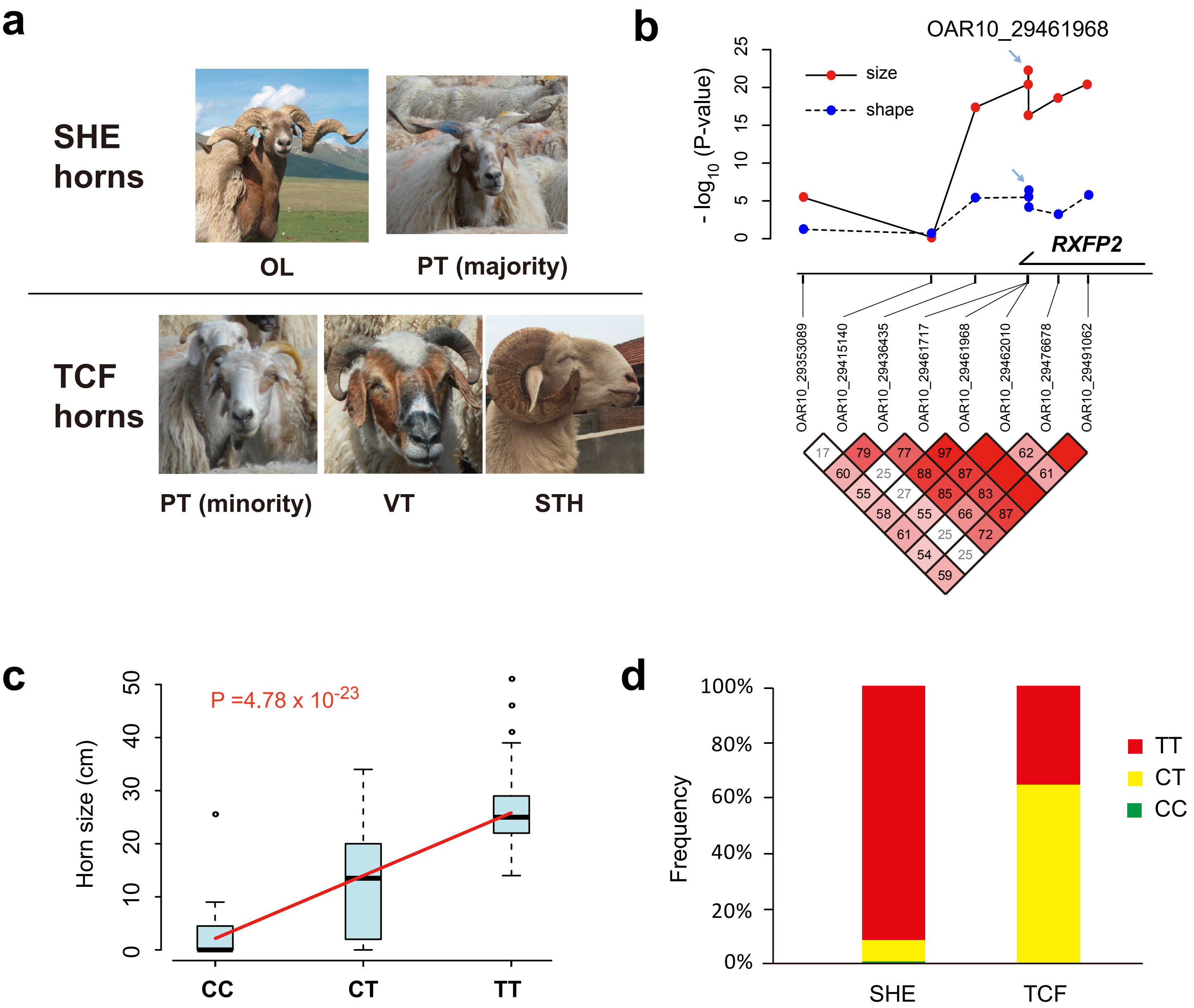

 Click here to download Figure Fig.6.revised.pdf

Figure 6

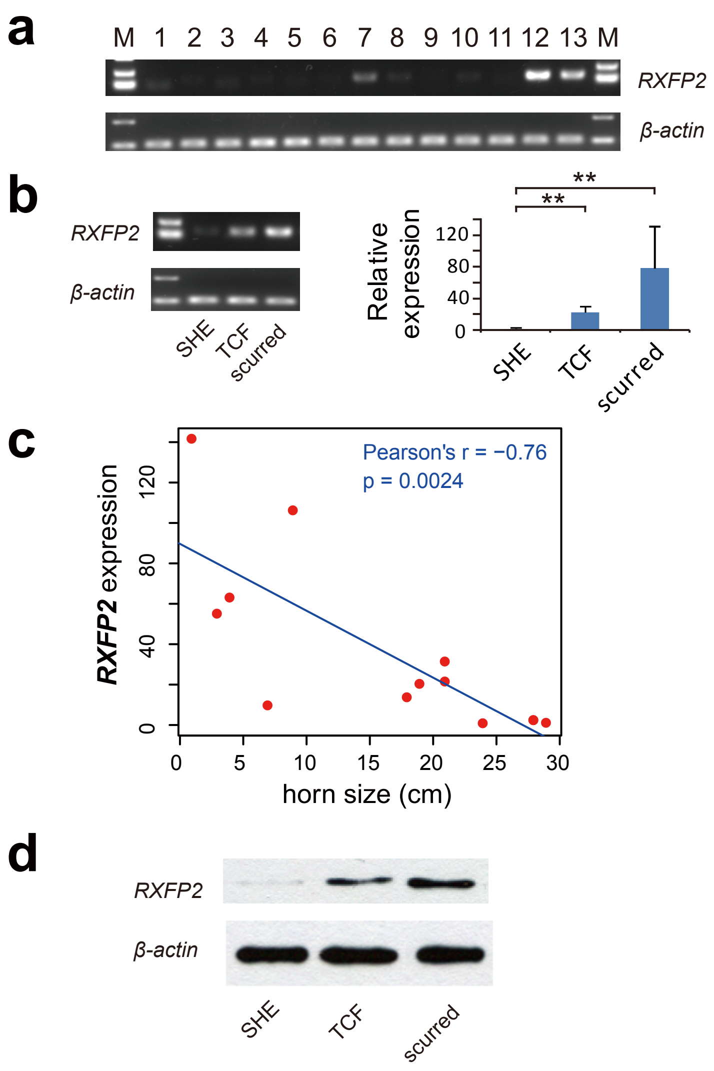

Click here to access/download
Supplementary Material
Supplementary_Materials.revised.doc

Click here to access/download
Supplementary Material
Supplementary Tables.revised.xlsx

Cover letter

Click here to download Personal Cover Cover_letter_to_editor.docx

Yixue Li, Ph.D

Key Laboratory of Computational Biology, CAS-MPG Partner Institute for Computational Biology,

Shanghai Institutes for Biological Sciences, Chinese Academy of Sciences,

320 Yue Yang Road, 200031 Shanghai, China

Email: yxli@sibs.ac.cn

Tel: +89-021-54920089

Dear Dr. Hans Zauner,

Thank you very much for your great editorial work regarding our manuscript entitled "**Whole genome sequences of 89 Chinese sheep suggest role of *RXFP2* in the development of unique horn phenotype as response to semi-feralization**". We also thank all the reviewers for their valuable advices and constructive comments. After considering all these comments, we revised our manuscript and now re-submit it for your consideration of publication in *GigaScience*.

In the revised manuscript, we have carried out additional analysis of the data to address the concerns raised by reviewers. A brief summary of our major revisions is in the head page of "point-to-point responses to reviewer comments".

Also, we have invited a native-English speaker to help improve quality of the text. The number of co-first authors has been adjusted to three. Before uploading the manuscript, our raw data and genotypic data has been released through SRA and EVA databases. Please feel free to contact us with any questions or concerns.

Best Regards.

Yours Sincerely,

Yixue Li & Mingxing Chu

Point-to-point responses to reviewers
Click here to download Personal Cover point-to-point response to reviewer comments.docx

A general statement from the authors:

We thank all the reviewers for their great work in reviewing our manuscript entitled "**Whole-genome sequences of 89 Chinese sheep suggest role of *RXFP2* in the development of unique horn phenotype as response to semi-feralization**". Indeed, the advices and comments are provoke-thinking and helpful to improve the quality of our work. We have considered the questions and suggestions raised by the reviewers, and revised the manuscript accordingly. We list some major revisions here:

Additional analyses:

1. We performed selective sweep analysis, gene ontology analysis, *RXFP2* haplotype distribution calculation after breaking down four populations PT, OL, VT and BY.
2. We did target sequencing of five random genomic regions in original samples (76 out of 99 sheep) to evaluate the accuracy of our WGS variant calling approach.
3. We compared the candidate gene list of present study with those from two previous approaches of Chinese native sheep.
4. We compared the *RXFP2* haplotype in our dataset with that reported in wild bighorn sheep (based on their vcf genotype file, downloaded from internet).
5. We calculated breed-to-breed F_{ST} distance and generated a NJ tree.

Figures:

1. **Figure 1b** is replaced with a F_{ST} tree.
2. Original **Figure 2** is broken down into two figures. Currently, **Figure 2** is comprised of F_{ST} and H_P manhattan plots from four divergent populations; **Figure 3** shows the result of candidate gene analysis.
3. Figure details and legend texts are revised according to the suggestions by reviewers.

Main text:

1. The Introduction and Discussions were revised after we have consulted literatures recommended by the reviewers.
2. The Analyses section was revised based on new results (separately in four populations).
3. We marked up some of the revisions in the main text, those we think are essential for addressing the question asked by the reviewers.
4. Typos have been carefully checked and corrected. The manuscript was edited by a native-English speaker before uploading.

Point-to-point responses

Authors: Here are our point-to-point responses to the reviewers' questions. Please note that for each reviewer we grouped the comments on typos and language editing as one point.

Reviewer 1

General comments by reviewer:

Reviewer: *The authors have performed sequencing of 99 sheep from multiple populations in China (and Australia). They identify many regions with high Fst as well as reduced heterozygosity. Their top region contains the RXFP2 gene, which they use for functional analysis linking it to horn size (as has been reported earlier).*

Major comments and responses:

1. Reviewer: *Figure 1 nicely summarizes the breeds and their geographic location and their genomic relationship. However, the authors quickly put the PT and OL population together labeling it TBS1. The VT population is relabeled TBP2. Since the TBS1 population separates according to the two breeds these populations should be treated separately. The paper needs to be redone throughout based on keeping the two (TBS1) populations separate.*

Authors: It is a great suggestion to provide more information by treating each breed separately, even if we think PT and OL may share a similar trajectory of adaptation (as they are genetically proximate according to the PCA and admixture plot). In the revised manuscript, we reported selective sweep and candidate gene analysis in PT, OL, VT and BY, by comparing them separately with MGS (line 182-190, also see **Figure 2, 3a-c**). Overlaps between gene sets in different breeds were showed as venn plots (**Figure 3a**).

2. Reviewer: *Figure 2 shows the Fst for TBS1 vs MGS and TBP2 vs MGS. It would be great to be able to compare the Fst plots of PT, OL, VT and BY separately compared to the MGS. This would allow the comparison of the signals that come up in different combinations of populations. I.e. PT & OL = big SHE horn; PT, OL, VT and BY = high altitude.*

Authors: We agree that showing more Manhattan plots potentially gives more combinations of parameters, like horn size and altitudes. To address this, we analyzed the selective sweep and associated candidate genes in PT, OL, VT and BY compared with MGS (line 182-190). The result shows that BY has a smaller gene set (n=758) than PT, OL and VT (n=1125; 999; 1046), which makes sense because BY has relatively small genetic differences from other MGS lineages. Moreover, inclusion of the BY gene set revealed *NF1* as a consistent signal across four populations (**Figure 3a, c**). However, more hypoxia-related genes (e.g. *PTEN*, *PINK1*) have undergone lineage-specific evolution (**Figure 3c**).

It is also worth noting that BY may not necessarily share their altitude adaptation genes with TBS.

The polygenic basis of hypoxic response pathway means that different high-altitude lineages may have multiple ways to achieve the similar adaptations (a well-known example is the Tibetan chicken). In this case, although it would be interesting to know whether some "altitude genes" are shared by TBS and BY, considering consistency among high-altitude populations (PT, VT, OL, BY) may not be a golden standard to detect all "altitude genes", since BY is a distant population and the adaptations are more likely to be lineage-specific. This concern is discussed in line 244-249.

3. Reviewer: *While the data would be clearer if each of the four populations were shown, the authors may want to comment on the regions now found on chrom 13 and 15 - maybe they are related to altitude?*

Authors: A consistently high F_{ST} in PT vs. MGS, OL vs. MGS and VT vs. MGS could be explained by a selective sweep either in TBS (PT + OL + VT), or MGS. A simple way to distinguish these two possibilities is to check whether TBS or MGS shows reduction of heterozygosity, or to calculate which haplotype is more different from the ancestral state (the lineage-specific branch length, LSBL). According to **Figure 2**, the window F_{ST} and H_P signals on chromosome 13 and 15 suggest the latter possibility, which means they might contribute to the adaptive evolution of MGS.

4. Reviewer: *The authors also need to compare the identified Fst regions with those found in previous sheet selection studies. It would also be interesting to compare the exact RXFP2 haplotypes 'associated' with horn here and in previous studies.*

Authors: We followed this helpful suggestion and compared the candidate genes at all sweep regions we found (PT, OL, VT and BY vs. MGS) with two previous studies of Chinese sheep (**Supplementary Table S12**). Genes underlying altitude adaptations such as *NF1* were confirmed by multiple studies. However, our data suggested a large number of genes underwent lineage-specific evolution, which is probably the reason why they are not identified when treating all Tibetan lineages as one group in other approaches.

We also compare the *RXFP2* genotypic data in our 99 sheep with that from the wild bighorn sheep (**Supplementary Figure S9, S10**), whose WGS data are easily accessible from the Dryad (doi:10.5061/dryad.3f2t2). The result suggested that PT and OL were basically carrying a different haplotype from the bighorn sheep, although they were all subject to selective pressures at the same locus *RXFP2* for developing strong horns.

We also think it would be informative if we can directly compare our genotypic data with more populations based on SNP chips. We consulted the most extensive collection of world-wide ovine genotypic data [1]. Nevertheless, we found that the SNP markers near *RXFP2* are not polymorphic in our dataset, which means that these markers cannot be used to explain the haplotypic differences between SHE and TCF populations in our study.

5. Reviewer: *How were SNPs/genotypes called given the low coverage sequencing data? Were all data from a breed analyzed together?*

Authors: The variant calling step was performed by an early version of samtools (v1.2), which by default imputed missing genotypes based on other samples that were simultaneously processed. All data from one breed (n=10) were analyzed together, and then data from different breeds were merged into single variant file.

The advantage of the low-coverage design is to have more samples when given a total coverage depth for each breed (average depth per breed = average depth per sample × samples per breed), while the disadvantage is, apparently, the higher missing rate at each genetic variant. However, genotypic imputation (based on individuals from same breed) and window-based metrics compromised the problem, as the missing variants are supposed to be randomly distributed on the genome and among individuals.

6. Reviewer: *Using multiple breeds for the analysis it might be possible to break down the selection signal near/over the RXFP2 signal (p 10 first paragraph, p13 1st paragraph - are haplotypes the same in the two semi-feral populations?)*

Authors: It is a very helpful suggestion to break down the signal of selective sweep by each population. In the current version of **Figure 4** and its related sections in the main text (line 254-275), we separately showed signals of each population (PT, OL, VT and BY), and the result was consistent with the previous one when PT and OL were combined. Basically, PT and OL have one dominant haplotype, and rest of the sheep breeds has another. A slight difference in haplotype frequency was observed between PT and OL as showed in **Figure 4d**. This is in rough agreement with the fact that SHE-type horns comprise >70% of PT population (in our 182 PT dataset), and is nearly fixed in OL population.

Minor comments and responses:

7. Reviewer: *L36 "RXFP2 underlied rapid evolution" replace with "RXFP2 showed signs of rapid evolution"*

-L43: "frequent contact with wild surroundings and rare human interventions".

-Keywords: add "sheep"

-L51: "the process where domestic animals"

-L54 "fit natural life while human artificial selection is no longer"

-L57 "trace back to 8 ka" replace with "trace back to 8,000 years ago "

-L58 "mito-genomic evolutionary study"

-L67-69: "as the Tibetan Plateau is rich in grasslands, the local breeds, especially ones living on prairies, have been roaming with nomads and fed on natural ranches"

-L71 "Third, unlike in other"

-L75 "loosened"

-L77-78 "breed from the Tibetan Plateau"

-L79 "sweeps"

-L84: "three"

- L118: "a rooted tree using the genome of the goat"

-L128 "performed principal component"

-L129 "Despite the division"

-L142-3 "statistics across the genome"

-L152 "from a relatively"

-L177 "observed the strongest signal"

-L178 "on chromosome 10"

-L181 "A previous study"

-L197 "in regions of positive selection"

-L199 "processes"

-L213 "intensely"

-L217 "a correlation"

-L231 "the TBS1 populations"

-L234-5 "the harsh environment on the Tibetan"

-L247 "variants in RXFP2"

-L297-8 "over the RXFP2 locus"

-L307 "compared the gene expression in"

-L308 "Despite obvious"

-L322 "study suggest that domestic animals might have re-acquired"

-L331 "to the Tibetan"

-L333 "selective sweeps"

-L340 "in competition to reproduce"

-L373 "evidence"

-L380 "across the RXFP2 gene"

-L483 "in RXFP2"

-L490 "genotyped in 182"

-L533 "levels of the five genes"

-p37: Figure 1 legend: spell out the names/shortenings of all sheep populations

-L746 "of the sheep pictures represent their lineage"

-L751 "clusters from K=2-4"

-L755-6 "regions under selection in TBS1"

-L768 "in red. For 627 TBS1 has the variant allele, whereas for 641 it has the reference allele"

-L779 Fig 4d: write how many individuals were included in analysis

-L789 linear regression is shown in red - it is not red - please correct

Authors: We appreciate these helpful suggestions to improve the quality of text. All these details were revised accordingly, and the manuscript was edited by a native speaker before uploading.

8. Reviewer: - *L90 when you describe the two different semi-feral populations, you may want to indicate also which sheep are at high altitude as this might be an important parameter.*

Authors: As suggested by the reviewer, we add a sentence at line 95-98 to indicate PT, VT, OL and BY as four high-altitude populations.

9. Reviewer: - *L105 You describe that your dataset encompasses 94% of variants found in dbSNP. Can you say also how many novel SNPs you found?*

Authors: >37.3% of variants in our dataset were novel compared with dbSNP build 143. This information is supplemented in the current version of manuscript at line 112-113.

10. Reviewer: - *L190 MITF is being linked to hearing - it is worth noting that MITF is also frequently mutated in different coat color types so I don't think you know which is the case in this study.*

Authors: We agree with the reviewer on this point. In the revised manuscript, we have now emphasized that some of the candidate genes like *MITF* might have multiple phenotypic outcomes (at line 208-211), and we also highlighted the double function of *MITF* in **Figure 3b**.

11. Reviewer: - *A larger number of genes are described on p10 and it is not clear how they were selected and how they were assigned potential function.*

Authors: We now use **Figure 3b** to summarize the selection signals of these typical genes, as well as the key words of their functional categories related with semi-feralization (also see **Supplementary Table S19**).

12. Reviewer: - *p16 top paragraph: how were the five genes tested for expression chosen - what was rationale for 900 kb?*

Authors: The number 900 was a typo in our original submission. The five genes we tested actually covered a ~1Mb region from chr10:28,984,259 bp to chr10: 30,002,883 bp.

The idea of this step is to include functional genes that are possibly affected by the selective sweep observed near *RXFP2*. The major concern is that the observed sweep signal might be hitchhiking with a causal variant located in the regulatory DNA elements of the flanking genes. From the LD plot showed in **Supplementary Figure S13**, it is obvious that the 1Mb region is comprised of multiple LD blocks, which means that the selected window size should be big enough to include all potential hitchhiking variants. Since we did not observed concordant expression level change in flanking genes, it is strong supporting evidence that *RXFP2*, instead of hitchhiking genes, is causal to the horn phenotypic changes. See revision at line 326-327.

13. Reviewer: *-p18 first paragraph discussing expression pattern - would be useful to mention how many tissues you tried for expression before saying the RXFP2 gene is specifically expressed.*

Authors: The sentence is now revised as "Thirdly, gene expression analysis in 13 tissues of PT sheep demonstrated …". (line 364-365)

14. Reviewer: *-L363+ How do you know whether sheep have been selected for a new mutation versus picked up the ancestral state again? Can you find the key candidate mutations and see if they if they overlap between ancestral wild sheep and the re-feralized populations?*

Authors: Our observations suggest PT and OL sheep were selected to evolve big and aggressive horns in a similar way like their wild relatives (because of sexual competition), but indeed the data is not sufficient to clarify whether they have derived a new genotype or picked up an old one. We showed two candidate protein mutations 627 and 641 with high F_{ST} and strong correlation with horn phenotypes (**Figure 4c**). Nevertheless, their phylogenetic origins are contradictory (where at 641 PT and OL have the ancestral allele, at 627 they have the mutated allele). We also compared the *RXFP2* haplotype of our populations with the wild bighorn sheep (**Supplementary Figure S9-S10**), but failed to see a correlation between the wild population and SHE horned PT and OL. To further clarify the origin of this phenotype, it would be interesting to test whether the *RXFP2* haplotype conferring SHE horns might have come from other sheep population with similar horn shapes (Racka), as well as from other wild populations regarded as the ancestors of Chinese sheep, but this is not currently achievable because there is no available data.

15. Reviewer: *-p21 first paragraph - how much false positive and false negative variants do you expect?*

Authors: To address this question, we performed targeted sequencing of five random genomic regions (including 33 SNPs) over 72 out of the 99 original samples. The results showed in **Supplementary Table S26** were utilized to calculate false positive and false negative rates of the SNP calling step. The false positive rate (FPR) defined as the proportion of wrongly defined mutated alleles is 3.34% (FPR = 34/1017) in tested samples, while the false negative rate (FNR) defined as the proportion of wrongly defined reference alleles is 1.47% (FNR = 53/3611).

		**Sanger (Validation)**		
		**Ref**	**Alt**	**Total**
**WGS variant calling**	**Ref**	3558	34	3630
	**Alt**	53	945	998
	**Total**	3611	1017	

Ref, Reference allele; Alt, Alternative allele;

According to the ensuing validation, heterozygotes has a much higher false calling rate than homozygotes: 63 out of 411 heterozygotes and 19 out of 1,903 homozygotes were not correctly genotyped in variant calling step (error rate: heterozygotes = 15.3%; homozygotes = 1.0%). This error type distribution does not exceed our expectation, because calling genotypes of heterozygotes in diploid organisms often requires a high coverage depth. The average read depth for each individual is about 6X in our study (one allele is overlapped with ~3 reads), which is quite satisfactory for detecting population-wide allele frequencies, but with restricted power to annotate allelic heterogeneity.

16. Reviewer: *-L449 - Is it possible that the 46 windows with few variants may be selected similarly in both populations?*

Authors: We list in the current **Supplementary Table S28** the sweep statistics for the 46 removed windows. The rationale for removing these windows is because the sweep signals are less convincing when there are few numbers of variants. Indeed, there are significant F_{ST} and H_P also in these few variant regions, but it is difficult to test whether that is caused by a few random fixations without sufficient observations of genetic hitchhiking. On a different note, with a relatively small window size and step size (30kb, 15kb), a selective sweep signal is often broken down into multiple windows (e.g. the signal on chromosome 20), which enables us to detect most of the sweeps even if we exclude 46 few variant regions.

17. Reviewer: *-23 last paragraph: it is quite possible some genes, such as MITF might have multiple mutations/multiple sweep signals across the gene for different coat color patterns. I would therefore not use the majority rules but actually report all signals.*

Authors: We agree that the majority rule is not applicable if one gene has multiple sweep events. We now have defined candidate genes from each population separately (based on F_{ST} and H_P), and then calculated their overlaps like showed in **Figure 3a**.

18. Reviewer: *-L765 add the window size used around the gene*

Authors: The visualized window is from chromosome 10: 29,400,000-29,550,000 bp. This information is added to the legend text (line 804-805).

19. Reviewer: *Figure 1: a. Make lines from map to breed not have extra lines*

b/c. replace tBS labels with individual breed labels

Authors: The figure is revised according to the suggestion.

Figure 2: adjust to show all four divergent populations in parallel

Authors: The current version of **Figure 2** contains parallel comparison of F_{ST} and H_P from four

divergent populations.

Reviewer 2

General comments by reviewer:

Reviewer: *This is a useful study on whole-genome sequences from Chinese sheep, which yields evidence that a mutation in the RXPF2 gene is causative for a horn phenotype as adaptation to semi-feralization. The analysis is according to the state-of-the-art. It is likely that the dataset harbors many more mutations in several of the hundreds of genes implicated in environmental adaptation, so there are clear opportunities for follow-up studies. The present results are interesting and deserve publication after a major revision.*

Major comments and responses:

1. Reviewer: *We understand that a grandiloquent title attracts attention. However, this title does not mention that the study is focused on Chinese sheep and does not refer to RXPF2, the major target of this study, the subject in three of the five figures and also dominating the Discussion. A possible alternative:*

Whole-genome sequences of 89 Chinese sheep breeds suggest a role of RXFP2 in the development of a unique horn phenotype as response to semi-feralization.

Note that such a more informative title transmits the same message as the present one (and does it even better).

Authors: As suggested by the reviewer, we have now revised the title as "Whole-genome sequences of 89 Chinese sheep suggest role of *RXFP2* in the development of unique horn phenotype as response to semi-feralization".

2. Reviewer: *Giga amounts of data require time-consuming analyses, which can only deliver a small part of the potential output. However, this should not be at the expense of an essential part of any scientific report: a comparison with previous literature, mentioning result that are not entirely novel but confirm previous findings. Ref. 14 reports WGS of 80 sheep from 3 climate zones in China. Because in this study Tibetan sheep were treated as one group, RXFP2 as gene subject to selection has been missed. However, Yang et al. [14] also target the high-altitude adaptation of Tibetan sheep, highlighting the role of SOCS2. A complete meta-analysis would be most fruitful, but is outside the scope of the current submission. Nevertheless, the Introduction and Discussion should pay more attention to the previous study [14] and at least touch the following points:*

(1) The introduction should refer to the demographic history of the main groups of Chinese breeds [14; Zhao et al. (2017), Genomic reconstruction of the history of Chinese native sheep: insights into peopling role of nomadic nationalities societies and expansions of early pastoralism. Mol. Biol. Evol., in press and accessible via Internet].

Authors: We appreciate this helpful suggestion and agree that the Introduction and Discussion would be more informative after revision on these points. We have consulted the papers suggested by the reviewer, and revised the second paragraph of the Introduction. In its current form, we

briefly describe the demographic history and geographic distributions of Chinese sheep based on literatures. This is in order to give a general picture of domestic sheep origin and their spread in China, as well as the sequential order of the split of major ovine groups from their ancestral lineage.

Reviewer: *(2) How are the PT, OL and VT breeds related to the Nagqu (ZNQ), Qamdo (ZCD), Shigatse (ZRK), Nyingchi (ZLZ), also from Tibet [14]? Lines 147-150 mention only briefly the proximity of VT and ZLZ. I recommend a Supplementary map giving the locations of the populations studied in [14] and in the present report.*

Authors: As suggested by the reviewer, we have provided a map (**Supplementary Figure S5**) to show the geographic distribution of different Tibetan lineages in our study and in the previous study of native sheep. The introduction of this geographic distribution patterns is described in a separate paragraph at line 152-159.

Reviewer: *(3) If phenotypic data are available for the other Tibetan breeds [14]: do they also have twisted SHE horns?*

(4) If so, and assuming the WGS data from [14] are accessible: do they also have the same RXPF2 mutation? This would lend strong support to the message of this study!

Authors: We agree that a direct comparison between our genotypic data and that from the suggested reference paper will be valuable. However, the raw reads or genotypic data from the suggested reference paper has not been released to any public resource. We also tried to contact the authors, but failed to get the access.

(5) Do both studies share other genes as being implicated in adaptation to the high-altitude and hypoxia? I saw that at least part of the genes listed in the Supplementary Table 10 are also mentioned in [14] as being selected in Tibetan sheep. It is relevant to indicate these shared genes in Table S10, if only to indicate that these results are not novel.

Authors: In the current **Supplementary Table S12**, we have listed the candidate genes in our four populations PT, OL, VT and BY, and their overlap with two previous gene list from references [2] and [3].

3. Reviewer: *At the beginning of the Discussion, a clear survey of the most essential features of diversity pattern would support the take-home message: a separate position of Tibetan sheep; within these sheep a contrast of domestic and semi-feral breeds, the former even less diverse than the latter; development of a unique semi-feral horn morphology as plausible adaptation to semi-feralization.*

Authors: We followed the suggestion and revised the first paragraph of the Discussion accordingly (line 346-353).

4. Reviewer: *Fig. 1b: a tree of NeighborNet graph of FST genetic distance between the breeds will be more informative and better support the message of this paper.*

Authors: Indeed, the F_{ST} tree can better represent the structure across breeds. From the current **Figure 1b**, a clear relationship between 10 breeds is showed. Nevertheless, we also preserved the previous phylogeny tree in **Supplementary Figure S2** because it provided additional information of the relationship between individual samples, and the position of root (goat).

5. Reviewer: *The manuscript needs to be read by a native-English speaker, preferable a scientist, in order to weed out the several awkward phrasings. A few are mentioned below.*

Line 49: "in order to understand better (etc.)".

Line 52: you probably mean that protection offered by the domestic habitat suppresses the original environmental adaptation.

Line 108: rephrase in order to indicate more clearly that the nucleotide diversity in Tibetan breeds is higher than in other Chinese breeds.

Lines 265-266, rephrase: "The SHE horns are clearly different from the horns of (etc.)".

Line 331: "the Tibetan Plateau". Lines 331-333: awkward and superfluous sentence. Line 333: selective sweeps [plural].

Line 361: "naturalistic" refers to an artistic style; probably you mean a natural wildlife habitat.

Line 543: Goa -> goat

Authors: We have carefully checked and revised these sentences mentioned by the reviewer. The revised manuscript was edited by a native speaker before uploading.

Minor comments and responses:

6. Reviewer: *Fig. 1: please define in the legends the abbreviations for the breed categories (EUS, MGS, TBS1, TBS2).*

Authors: We revised the legend text of **Figure 1**. Its current form contains definition of all abbreviations for breeds and lineages.

7. Reviewer: *It is a good idea to use colors consistently across figures. However, in Fig. 1a the MGS sheep should be shown at a dark blue background and the TBS sheep at a light blue background instead of vice versa in order to harmonize with Figs. 1d and S3.*

Authors: We realize that some color inconsistency in our figures is misleading to the readers. To address the problem, we have adjusted the color in **Figure 1** and **Figure S3**. Also, we paid more attention to color consistency in other figures, such like that between **Figure 2** and **3c**.

8. Reviewer: *Data have been submitted to the SRA. In addition, it would be most useful to submit*

the novel SNPs to the Ensemble Variation Archive.

Authors: We assume you mean the European Variation Archive (EVA), which collects variation data from non-human organisms.

The vcf file was submitted to EVA before uploading this revised manuscript. All data has been released to the public. See data access information at line 557-560

9. Reviewer: *Line 62: also refer to [14] and Zhao et al. (2017), who on the basis of genome-wide SNPs differentiate three breed clusters.*

Authors: We revised the introduction of Chinese sheep lineages based on the suggested literature (line 57-67).

10. Reviewer: *Lines 147-149: just mention the close proximity of ZLZ and VT and the comparably low LD. See point 2 about a more complete comparison of these breeds and other Tibetan breeds [14], which should precede this paragraph.*

Authors: The sentence is revised as "Moreover, population ZLZ in the other study was proximate to VT and also exhibited a sign of population bottleneck (evidenced by slow LD decay)." (line 166-168)

Also, the geographic distribution of our 3 populations and the 4 populations in [2] is now discussed in the preceding paragraph at line 152-159.

11. Reviewer: *Lines 150-153: just mention that the LD indicates a population bottleneck.*

Authors: The sentence is revised as "and also exhibited a sign of population bottleneck (evidenced by slow LD decay)" (line 166-168).

12. Reviewer: *Lines 154-156: this was already convincingly clear on the basis of Fig. 1.*

Authors: We deleted this paragraph.

13. Reviewer: *Lines 188-190 repeat the preceding paragraph; this should be integrated.*

Authors: We revised this sentence, so it now describes signals in addition to *RXFP2* (line 200).

14. Reviewer: *In this context, it is should be mentioned that the well-known Hungarian Racka sheep also has SHE horns (haven't they?).*

Authors: It is an intriguing similarity between SHE-horned Tibetan sheep and Hungarian Racka sheep, which we hadn't noticed before. An important question behind this is whether the

SHE-horn genotype is newly derived in semi-feral TBS, or is an introgression from other sheep populations. We are not sure which is the case, since we don't have the genotypic data from other possible "donors" of *RXFP2* haplotypes, including Racka. From our data, what is certain, however, is that this haplotype of *RXFP2* confers SHE horns, and is nearly driven to fixation in semi-feral TBS under positive selection. See our discussion at line 375-381.

15. Reviewer: *Lines 268, 362: of course, the horns are used during fighting with competitors and predators, but it is a bit curious to state that SHE sheep and the wild ancestors look strong and aggressive; better omit these statements.*

Authors: As suggested, we deleted these sentences.

16. Reviewer: *Figs. 4b and 4d can easily be combined, while the legends should mention more clearly that (as I understand) they show correlations with horn length and horn shape, respectively.*

Authors: These two figures are now combined into **Figure 5b**, where different line types were used to indicate the measurement outcome (either horn size or shape).

17. Reviewer: *Lines 317-323: this paragraph can be omitted since the same points will be made in the Discussion (where it belongs anyway).*

Authors: As suggested, we removed this paragraph.

References

1. Kijas JW, Lenstra JA, Hayes B, Boitard S, Neto LRP, San Cristobal M, et al. Genome-Wide Analysis of the World's Sheep Breeds Reveals High Levels of Historic Mixture and Strong Recent Selection. Plos Biology. 2012;10 2.
2. Yang J, Li WR, Lv FH, He SG, Tian SL, Peng WF, et al. Whole-genome sequencing of native sheep provides insights into rapid adaptations to extreme environments. Molecular Biology and Evolution. 2016;33:2576-92. doi:10.1093/molbev/msw129.
3. Wei C, Wang H, Liu G, Zhao F, Kijas JW, Ma Y, et al. Genome-wide analysis reveals adaptation to high altitudes in Tibetan sheep. Scientific reports. 2016;6:26770. doi:10.1038/srep26770.
