# Supplementary material for: Whole-genome sequences of 89 Chinese sheep suggest role of RXFP2 in the development of unique horn phenotype as response to semi-feralization
Source: Gigascience. 2018 Mar 7;7(4):giy019. doi: 10.1093/gigascience/giy019 (PMC5905515; doi:10.1093/gigascience/giy019)
Supplement: GIGA-D-17-00165_Revision_2.pdf [file giy019_giga-d-17-00165_revision_2.pdf]

# GigaScience


## Whole-genome sequences of 89 Chinese sheep suggest role of RXFP2 in the development of unique horn phenotype as response to semi-feralization

--Manuscript Draft--

| | |
|---|---|
| **Manuscript Number:** | GIGA-D-17-00165R2 |
| **Full Title:** | Whole-genome sequences of 89 Chinese sheep suggest role of RXFP2 in the development of unique horn phenotype as response to semi-feralization |
| **Article Type:** | Research |
| **Funding Information:** | |


| Funding | Recipient |
|---|---|
| Agricultural Science and Technology Innovation Program of China (ASTIP-IAS13) | Prof. Mingxing Chu |
| Earmarked Fund for China Agriculture Research System (CARS-39) | Prof. Mingxing Chu |
| National Key Technology Support Program (2013BAI101B09) | Prof. Yixue Li |
| National Natural Science Foundation of China (CN) (31472078) | Prof. Mingxing Chu |
| National Natural Science Foundation of China (31402041) | Dr. Qiuyue Liu |
| National Key Scientific Instrument and Equipment Development Project (2012YQ03026108) | Prof. Yixue Li |
| National Basic Research Program of China (2011CB910204) | Prof. Yixue Li |
| National Basic Research Program of China (2011CB510102) | Prof. Yixue Li |
| Youth Innovation Promotion Association of the Chinese Academy of Sciences (2017325) | Dr. Zhen Wang |
| Genetically Modified Organisms Breeding Major Program of China (2016ZX08009-003-006) | Dr. Qiuyue Liu |
| Genetically Modified Organisms Breeding Major Program of China (2016ZX08010-005-003) | Prof. Mingxing Chu |
| Major Science and Technology Program of Inner Mongolia Autonomous Region of China | Prof. Mingxing Chu |


**Abstract:**


Background
Animal domestication has been extensively studied but the process of feralization remains poorly understood.
Results
Here, we performed whole-genome sequencing of 99 sheep and identified a primary genetic divergence between two heterogeneous populations in the Tibetan Plateau, including one semi-feral lineage. Selective sweep and candidate gene analysis revealed the local adaptations of these sheep associated with sensory perception, muscle strength, eating habit, mating process and aggressive behavior. In particular, a horn-related gene RXFP2 showed signs of rapid evolution specifically in the semi-feral breeds. A unique haplotype and repressed horn-related-tissue expressions of RXFP2 were correlated with higher horn length, as well as spiral and horizontally extended horn shape.
Conclusions
Semi-feralization has an extensive impact on diverse phenotypic traits of sheep. By acquiring features like those of their wild ancestors, semi-feral sheep were able to re-gain fitness in frequent contact with wild surroundings and rare human interventions.



*Powered by Editorial Manager® and ProduXion Manager® from Aries Systems Corporation*

| | |
|---|---|
| | The present study provides a new insight into the evolution of domestic animals when human interventions are no longer dominant. |
| **Corresponding Author:** | Shengdi Li<br><br>CHINA |
| **Corresponding Author Secondary Information:** | |
| **Corresponding Author's Institution:** | |
| **Corresponding Author's Secondary Institution:** | |
| **First Author:** | Zhangyuan Pan |
| **First Author Secondary Information:** | |
| **Order of Authors:** | Zhangyuan Pan |
| | Shengdi Li |
| | Qiuyue Liu |
| | Zhen Wang |
| | Zhengkui Zhou |
| | Ran Di |
| | Benpeng Miao |
| | Wenping Hu |
| | Xiangyu Wang |
| | Xiaoxiang Hu |
| | Ze Xu |
| | Dongkai Wei |
| | Xiaoyun He |
| | Liyun Yuan |
| | Xiaofei Guo |
| | Benmeng Liang |
| | Ruichao Wang |
| | Xiaoyu Li |
| | Xiaohan Cao |
| | Xinlong Dong |
| | Qing Xia |
| | Hongcai Shi |
| | Geng Hao |
| | Jean Yang |
| | Cuicheng Luosang |
| | Yiqiang Zhao |
| | Mei Jin |
| | Yingjie Zhang |
| | Shenjin Lv |
| | |

Powered by Editorial Manager® and ProduXion Manager® from Aries Systems Corporation

| | |
|---|---|
| | Fukuan Li |
| | Guohui Ding |
| | Mingxing Chu |
| | Yixue Li |
| **Order of Authors Secondary Information:** | |
| **Response to Reviewers:** | We would like to thank again the editor and reviewers for their critical suggestions on our manuscript during the entire submission process. In this 2nd revision, we have made language corrections including all those suggested by the reviewer. Moreover, we fixed some incorrect annotations in the Supplementary Table S20-23, which were caused by errors during extracting protein-altering information. Details of these corrections are listed below:<br><br>Language corrections:<br>1.Reviewer: L65: denoted as Mongolian sheep because of their distinctive fat-storage phenotypes (fat tails or rumps) (etc).<br>L68: Omit first 4 words.<br>L113: were absent from the public collection-> are novel<br>L159: is not the only determinant of (etc.)<br>L164: omit "during localization"<br>L166: Moreover, population ZLA is proximate to VT and also exhihits slow LD decay as sign of a population bottleneck.<br>L168-170: Omit this sentence. (has already been said)<br>L209: MITF variants that contribute<br>L349: critical role of semi-feralization in development of male reproductive success, eating habits (etc.). (present formulation a bit awkward)<br><br>Authors: Corrections done.<br><br>2.Reviewer: L237: corroborate (present tense for findings that still are true)<br><br>Authors: We now use “provide new evidences to the hypoxic adaptation of TBS”, which we agree is more precise.<br><br><br>Update to Supplementary Table S20-23:<br>Authors: We updated the columns related with protein-altering annotations because we found the previous one is incorrect at some specific rows (for example, in Table S20, row 12 column E, “p.Glu641Gln” should be “p.Glu641Lys”). This is caused by an error in our previous pipeline for extracting these annotation information, which has been fixed now. |
| **Additional Information:** | |
| **Question** | **Response** |
| Are you submitting this manuscript to a special series or article collection? | No |
| **Experimental design and statistics**<br><br>Full details of the experimental design and statistical methods used should be given in the Methods section, as detailed in our Minimum Standards Reporting Checklist. Information essential to interpreting the data presented should be made available in the figure legends.<br><br>Have you included all the information requested in your manuscript? | Yes |

Powered by Editorial Manager® and ProduXion Manager® from Aries Systems Corporation

| | |
|---|---|
| **Resources**<br><br>A description of all resources used, including antibodies, cell lines, animals and software tools, with enough information to allow them to be uniquely identified, should be included in the Methods section. Authors are strongly encouraged to cite Research Resource Identifiers (RRIDs) for antibodies, model organisms and tools, where possible.<br><br>Have you included the information requested as detailed in our Minimum Standards Reporting Checklist? | Yes |
| **Availability of data and materials**<br><br>All datasets and code on which the conclusions of the paper rely must be either included in your submission or deposited in publicly available repositories (where available and ethically appropriate), referencing such data using a unique identifier in the references and in the “Availability of Data and Materials” section of your manuscript.<br><br>Have you have met the above requirement as detailed in our Minimum Standards Reporting Checklist? | Yes |

*Powered by Editorial Manager® and ProduXion Manager® from Aries Systems Corporation*

Manuscript
Click here to download Manuscript 99sheep22-2.doc

# Whole-genome sequences of 89 Chinese sheep suggest role of *RXFP2* in the development of unique horn phenotype as response to semi-feralization

Zhangyuan Pan[†,1,3], Shengdi Li[†,2,4], Qiuyue Liu[†,1], Zhen Wang[2], Zhengkui Zhou[1], Ran Di[1], Benpeng Miao[2,4], Wenping Hu[1], Xiangyu Wang[1], Xiaoxiang Hu[5], Ze Xu[6], Dongkai Wei[6], Xiaoyun He[1], Liyun Yuan[2], Xiaofei Guo[1], Benmeng Liang[1], Ruichao Wang[2], Xiaoyu Li[1], Xiaohan Cao[1], Xinlong Dong[1], Qing Xia[1], Hongcai Shi[7], Geng Hao[8], Jean Yang[9], Cuicheng Luosang[9], Yiqiang Zhao[5], Mei Jin[10], Yingjie Zhang[11], Shenjin Lv[3], Fukuan Li[3], Guohui Ding[2,12], Mingxing Chu*[,1] & Yixue Li*[,2,12]

[1]Institute of Animal Science, Chinese Academy of Agricultural Sciences, Beijing, China.

[2]Key Lab of Computational Biology, CAS-MPG Partner Institute for Computational Biology, Shanghai Institutes for Biological Sciences, Chinese Academy of Sciences, Shanghai, China.

[3]College of Agriculture and Forestry Science, Linyi University, Linyi, China

[4]University of Chinese Academy of Sciences, Beijing, China;

[5]State Key Laboratory for Agrobiotechnology, China Agricultural University, Beijing, China.

[6]BasePair BioTechonology Co., Ltd., Suzhou, China.

[7]Institute of Biotechnology, Xinjiang Academy of Animal Science, Urumqi, China.

[8]Institute of Animal Science, Xinjiang Academy of Animal Science, Urumqi, China.

[9]Research Institute of Animal Science, Tibet Academy of Agricultural and Animal Husbandry Sciences, Lhasa, China.

[10]College of Life Science, Liaoning Normal University, Dalian, China.

[11]College of Animal Science and Technology, Agricultural University of Hebei, Baoding, China.

[12]Shanghai Center for Bioinformation Techonology, Shanghai Industrial Technology Institute, Shanghai, China.

[†]These authors contributed equally to this work.

*These authors jointly directed this work.

Correspondence should be addressed to Y.L. (yxli@sibs.ac.cn) or M.C. (mxchu@263.net)

# Abstract

## Background

Animal domestication has been extensively studied but the process of feralization remains poorly understood.

## Results

Here, we performed whole-genome sequencing of 99 sheep and identified a primary genetic divergence between two heterogeneous populations in the Tibetan Plateau, including one semi-feral lineage. Selective sweep and candidate gene analysis revealed local adaptations of these sheep associated with sensory perception, muscle strength, eating habit, mating process and aggressive behavior. In particular, a horn-related gene, *RXFP2*, showed signs of rapid evolution specifically in the semi-feral breeds. A unique haplotype and repressed horn-related tissue expression of *RXFP2* were correlated with higher horn length, as well as spiral and horizontally extended horn shape.

## Conclusions

Semi-feralization has an extensive impact on diverse phenotypic traits of sheep. By acquiring features like those of their wild ancestors, semi-feral sheep were able to re-gain fitness in frequent contact with wild surroundings and rare human interventions. The present study provides a new insight into the evolution of domestic animals when human interventions are no longer dominant.

**Key words**

Domestic animal - Sheep - Adaptive evolution - Artificial selection - Semi-feralization - Horn

## Background

Animal domestication has been widely investigated to better understand the phenotypic and genetic changes of animals caused by human activities [1-4]. However, the process in which domestic animals become feral is still poorly understood. Domestication is the process where protection offered by domestic habitat suppresses the original environmental adaptation. Feralization is its reverse: the animals re-start to fit natural life while human artificial selections are no longer dominant [5].

The history of Chinese sheep domestication can be traced back more than 5,000 years according to archeological evidence [6, 7]. The demographic history of Chinese domestic sheep was recently reconstructed based on population genomics, which suggested their origin on the Mongolian Plateau about 5,000 to 7,000 years ago with later dispersal associated with historical movements of nomadic societies [8]. To date, more than 42 local breeds of sheep have been established in China, comprising lineages from three major geographic areas known as northern China, the Tibetan Plateau and the Yunnan-Kweichow Plateau [8, 9]. Sheep in northern China were also denoted as Mongolian sheep because of their distinctive fat-storage phenotypes (fat tails or rumps) [10, 11]. Tibetan and Yunnan-Kweichow sheep were split from Mongolian sheep about 4,000 years ago [8].

Different climate zones have an essential impact over the adaptive evolution of the major ovine lineages in China [9]. However, the role of various husbandry

cultures in affecting the phenotypes of modern sheep breeds is not well understood. In fact, the unique domestication history and husbandry system of Tibetan sheep makes it an appropriate evolutionary model for studying animal semi-feralization for several reasons. Firstly, because the Tibetan Plateau is rich in grassland, the local breeds, especially those living on prairies, have been roaming with nomads and fed on natural ranches. Secondly, these sheep were forced to encounter threats from the wild (e.g. Tibetan wolves), because of a sparsely populated and undeveloped environment. Thirdly, unlike in other pastoral areas of China, the breeding of Tibetan sheep was not subject to intense artificial control, such as gender-separating management and selective breeding. In this case, the evolution of these semi-feral populations can provide indications about how domestic animals adapt when artificial pressures are loosened.

To enhance the understanding of animal feralization, we sequenced and analyzed the genomes of 30 sheep from two semi-feral breeds and one domestic breed from the Tibetan Plateau and 69 domestic sheep from other geographic areas. We identified a primary divergence in Tibetan sheep and a set of candidate loci underlying selective sweeps in each Tibetan breed, which is responsible for their distinct phenotypic patterns related with semi-feralization.

## Data Description

We selected 30 sheep from three typical Tibetan breeds in the Tibetan Plateau

(PT, Prairie Tibetan sheep; VT, Valley Tibetan sheep; OL, Oula sheep), 59 sheep from six Mongolian breeds across northern China (BY, Bayinbuluke sheep; CB, Cele Black sheep; H, Hu sheep; T, Tan sheep; STH, Small Tail Han sheep; WZ, Wuzhumuqin sheep), as well as 10 Australian Merino sheep (AM) representing a European-originated breed (**Figure 1a**, **Supplementary Table S1-S2**). Among the 10 breeds, PT and OL were two semi-feral populations that did not receive extensive human interventions, while PT, OL, VT and BY were four populations living at high altitude (>3,000m above sea) (**Supplementary Table S2**). The sex ratio was maintained at approximately 1:1 for each breed. We performed whole-genome sequencing (WGS) of the 99 sheep. The coverage depth after genome alignment was approximately six-fold for each individual (**Supplementary Table S3-S4**), resulting in more than 50× coverage depth for each breed.

## Analyses

### Characterization of the variants

After applying stringent criteria in quality control, we identified a total of 38,090,348 single-nucleotide polymorphisms (SNPs) and 4,348,493 insertions/deletions (indels) in the 99 genomes (**Supplementary Table S5**). The abundance of variants was comparable to those of other domestic animals [4, 12, 13]. Most variants were intergenic or intronic, and only 269,584 SNPs and 5,518 indels were exonic (**Supplementary Table S6-S7**). Our dataset captured >94.0%

(26,598,869 SNPs and indels) of the variants in the dbSNP database build 143, whereas >37.3% (15,839,972 SNPs and indels) of the variants in the 99 sheep genomes are novel (**Supplementary Figure S1**). The genome-wide average diversity $\pi$ of the sheep breeds was estimated to be 2.44-2.84 × $10^{-3}$, which was similar as previously reported [9]. In other domestic animals such as pigs and dogs, nucleotide diversity in Tibetan breeds is often higher than in other Chinese breeds [12, 13]. However, our data suggested domesticated sheep in China has an opposite trend: Tibetan sheep breeds ($\pi$ = 2.44-2.61 × $10^{-3}$, $\theta$ = 2.10-2.30 × $10^{-3}$) have lower nucleotide diversity than Mongolian ($\pi$ = 2.69-2.79 × $10^{-3}$, $\theta$ = 2.36-2.52 × $10^{-3}$) and European breeds ($\pi$ = 2.84 × $10^{-3}$, $\theta$ = 2.50 × $10^{-3}$), which is consistent with the fact that Mongolian sheep diverged earlier than Tibetan sheep from their ancestral lineage [14].

**Population genetics of Chinese sheep**

To understand the genetic relationships among these local breeds, we constructed a neighbor-joining (NJ) tree based on their pair-wise genetic distances (measured by fixation index $F_{ST}$) (**Figure 1b**). We also calculated a phylogenetic tree based on genomic SNPs to visualize the relationship between individual samples, where a goat genome was used to calibrate the root (**Supplementary Figure S2a**). As expected, the European-originated sheep (AM and Texel) were the first clade separated from the ancestral lineage. That was followed by the Mongolian breeds and finally, the Tibetan

breeds. This phylogeny structure is again consistent with the migration trajectory of sheep, where Eurasian sheep initially migrated onto the Mongolian Plateau and then spread into local areas of China [14]. The three Tibetan sheep breeds formed a monophyletic clade which was robust under bootstrapping tests (**Supplementary Figure S2b**), indicating a common origin of Tibetan sheep from one recent ancestral lineage.

We next performed a principal component analysis (PCA) of 99 sheep based on their genomic variants (**Figure 1c**). Despite the division among Tibetan sheep (TBS), Mongolian sheep (MGS) and European sheep (EUS), a considerable genetic difference was observed between two groups of Tibetan sheep: one cluster consisted of 20 individuals from two semi-feral breeds PT and OL, while another consisted of 10 individuals from domestic breed VT (**Figure 1c**). We further examined the population structure by assuming the number of ancestry K (**Figure 1d, Supplementary Figure S3**). When K = 3, TBS, MGS and EUS were clearly separated, though BY, one breed of MGS, showed a mixture between TBS and MGS. When K = 4, we observed a primary divergence between semi-feral and domestic TBS, in agreement with the PCA result. In addition, analysis by TreeMix [15] confirmed the migration event from VT to BY (**Supplementary Figure S4**). Due to its genetic admixture, BY was treated separately from other MGS breeds during subsequent analysis.

The previous study of native sheep in China has included samples from four Tibetan populations (labeled as ZRK, ZLZ, ZNQ and ZCD) [9]. Here, we provided a

supplementary map to summarize their geographical locations and relationship with Tibetan breeds in the present study (**Supplementary Figure S5**). Briefly, VT, ZRK and ZLZ were in southern Tibet, while PT, OL, ZNQ and ZCD were in the north. OL was at a relatively distant area from other local breeds. However, as we observed high similarity between OL and PT, it seems that the geographical distance was not the only determinant of the genetic differences between breeds.

An intriguing phenomenon is that the domestic TBS breed VT seems to show a unique breeding history, represented by its slow linkage disequilibrium (LD) decay and the most positive Tajima's *D* statistics across the genome compared with other breeds (**Supplementary Figure S6**). These statistics suggest that VT has encountered the most severe contraction of population size. These sheep also showed lower genetic diversity ($\pi$ = 2.44 × $10^{-3}$) than semi-feral TBS ($\pi$ = 2.60-2.61 × $10^{-3}$), MGS ($\pi$ = 2.69-2.79 × $10^{-3}$) and EUS ($\pi$ = 2.84 × $10^{-3}$). Moreover, population ZLZ in the other study was proximate to VT and also exhibits slow LD decay as a sign of population bottleneck [9].

**Selective sweeps in semi-feral and domestic sheep**

We reasoned that different levels of human intervention might have resulted in distinct evolutionary trajectories of PT, OL and VT. For example, PT and OL raised by nomads were typically free-roaming, while VT were captive, intensively managed by local farmers for improving productions and efficiencies (**Supplementary Table**

**S2**). PT and OL live in under-developed regions of north Tibet, where human population is sparse (**Supplementary Figure S7**), suggesting less interaction with human society and more threats from predators (e.g. Tibetan wolves). Moreover, VT was subject to moderate selective breeding, while PT and OL received barely any intervention in their mating process (**Supplementary Table S2**)

To identify candidate genes under positive selection in different TBS populations, we performed a selective sweep analysis over the whole genome based on population differentiation (fixation index $F_{ST}$) and loss of heterozygosity (heterozygosity $\log_2[H_P$ ratio$]$) in PT, OL, VT and BY respectively, by comparing them with MGS (**Figure 2**). BY is not a TBS breed, but is included here to identify loci potentially under altitude adaptations (PT, OL: semi-feral group; PT, OL, VT: Tibetan group; PT, OL, VT, BY: high-altitude group) (**Supplementary Table S2**). In total, we identified 1,104, 988, 1,030 and 749 candidate genes in each of the four populations (**Figure 3a, Supplementary Table S8-S11**).

In two semi-feral populations, we observed a consistently strong signal of positive selection on chromosome 10, which harbors a relaxin/insulin-like family peptide receptor 2 (*RXFP2*) gene (**Figure 2, Supplementary Table S8-S9**). *RXFP2* is a well-known gene related with sheep horn phenotypes, and is often characterized as a target of natural and sexual selection in wild and feral populations [16, 17]. Since free mating is one of the typical features of wild and feral populations, and is often replaced with selective breeding in domestic lines, *RXFP2* potentially serves as the genetic marker of “wildness” in sheep, which confers their essential sexual weaponry

during competitions to reproduce.

In addition to *RXFP2*, we also characterized a number of PT and OL candidate genes with a significantly high window $F_{ST}$ and $H_P$ ratio, that are functionally plausible for adaptation in the wild (**Figure 3b**). For example, these genes include: (1) *MITF*, *MSRB3*, *SLC26A4* associated with hearing [18-25]; (2) *SMNDC1*, *SOX6* involved in muscle development [26, 27]; and (3) *PRD-SPRRII* regulating rumen development [28]. Their signals of selective sweep in four populations were in rough agreement with different extents of human intervention, where high $F_{ST}$ and low $H_P$ values were often restricted to PT and OL and were absent in VT and BY (**Figure 3b**). Moreover, it is worth noting that some of these candidates are known to mediate diverse phenotypes, like *MITF* variants that contribute to coat color patterns [29-31]. In such cases, additional information like phenotypic data will be necessary to define the real outcome of positive selection.

Then, we performed a Gene Ontology (GO) enrichment analysis of the gene sets in sweep regions of the four populations PT, OL, VT and BY (**Supplementary Table S13-S16**). We also analyzed enriched GO terms in overlapping gene sets representing the combinations of semi-feral sheep (PT and OL, gene number = 231), and Tibetan sheep (PT, OL and VT, gene number = 62) (**Supplementary Table S17-S18**). The results showed a number of feralization-related functional terms over-represented in PT, OL or their overlapping candidate genes (**Figure 3c**, **Supplementary Table S19**). For example, a set of key terms was found related with the process of mating and reproduction, such as androgen receptors (GO:0050681, GO:0030521), the maternal

process in female pregnancy (GO:0060135), and hormone metabolisms (GO:0046887, GO:0032353). Categories associated with muscle function, such as striated muscle development (GO:0014706), muscle cell apoptosis (GO:0010656, GO:0010657) and muscle adaptation (GO:0043500, GO:0043502), were also characterized. Moreover, other enriched functions include aggressive behaviors (GO:0002118), defense response (GO:0031347), digestive system development (GO:0055123, GO:0048565), as well as a number of GO clusters in sensory organ development (GO:0001754, GO:0042461, GO:0042462, GO:0046530, GO:0048592, GO:0048593, GO:0021772). All functional terms mentioned above had a significant enrichment score (P value < 0.05) after considering multiple testing errors (by the Benjamini-Hochberg approach). Taken together, these findings suggested that two semi-feral lineages of TBS have undergone diverse processes of semi-feralization in response to the natural environment and reduced human protections. The adaptation potentially brought them with advantages through free mating, improved muscle strength and food digestion abilities, and promoted aggressiveness, defensive responses and sensory perceptions in order to survive attacks from predators.

On a different note, our data also provided new evidences to the hypoxic adaption of TBS. By comparing our candidate gene list with those from two other studies of TBS [9, 32], we found *NF1* as a consistent signal in response to altitude adaptation identified among independent approaches (**Supplementary Table S12**). Our results indicated that *NF1* was under positive selection in all four high-altitude populations (**Figure 3b**). Nevertheless, it is intriguing that other possible candidate genes of

altitude adaptations, such as *PTEN* and *PINK1*, are more often exhibiting lineage-specific signals (**Figure 3b**). Since the response to hypoxia is a well-known cellular process of polygenic basis, it is possible that the trajectories of hypoxia adaptation are highly heterogeneous among independent populations (an existing example is the Tibetan chicken [33]). Thus, it may explain the non-uniform distribution of hypoxia-associated sweeps among the four high-altitude populations.

### A horn-related locus *RXFP2* underlies positive selection in semi-feral sheep

We next investigated and validated the strongest adaptive signature of semi-feralization in PT and OL, at chromosome 10 spanning a 60-kb region of *RXFP2* gene (**Figure 2**). The sweep region exhibits excess of population differentiation ($F_{ST|PTvsMGS}$ = 0.736, $F_{ST|OLvsMGS}$ = 0.736) and a dramatic loss of heterozygosity ($H_{P|PT}/H_{P|MGS}$ = 0.768, $H_{P|OL}/H_{P|MGS}$ = 0.757) in two semi-feral breeds (**Figure 4a, Supplementary Table S8-S9**). Nevertheless, neither of these signals was observed in VT ($F_{ST|VTvsMGS}$ = 0.052, $H_{P|VT}/H_{P|MGS}$ = 0.976), while BY exhibited a moderate increase in genetic differentiation ($F_{ST|BYvsMGS}$ = 0.187) but with no evidence of heterozygosity depletion ($H_{P|VT}/H_{P|MGS}$ = 1.519). The pattern of SNPs located in the *RXFP2* gene region revealed a unique haplotype in PT and OL, which was obviously different from those in VT, BY, MGS and EUS (**Figure 4b**).

Two missense mutations in *RXFP2* (OAR10_29461968: E641K, OAR10_29462010: V627M) were characterized as most significantly differentiated

among its protein-altering variants (**Supplementary Table S20-S21**). Both sites are highly conserved among vertebrate species and were mutated in PT and OL compared with other sheep (**Figure 4c**). To confirm their haplotypic distributions among ten breeds, we examined the genotypes of 1,155 independent individuals at these two SNP sites. According to the result, the distributions of both SNPs were consistent with our whole genome sequencing (WGS) data, where the haplotype 1 consisting of "OAR10_29461968:T + OAR10_29462010:T" (*RXFP2*: M627 and K641) were mostly found in PT and OL, while the haplotype 2 consisting of "OAR10_29461968:C + OAR10_29462010:C" (*RXFP2*: V627 and E641) were predominant in VT, BY, MGS and EUS (**Figure 4d**, **Supplementary Figure S8**).

We also compared the *RXFP2* haplotypes observed in our 99 sheep with those reported in the wild bighorn sheep population, where *RXFP2* was selected for intra-sexual competitions [17]. The result showed no obvious similarity between these two haplotypes (**Supplementary Figure S9-S10**), suggesting different directions of adaptive evolution at *RXFP2* locus between the semi-feral TBS and the wild bighorn sheep.

Taken together, our results indicated that PT and OL sheep have formed a unique haplotype at *RXFP2* locus under the effect of positive selection.

### *RXFP2* haplotype controls horn size and shape

A common feature of the semi-feral populations PT and OL is that they often

have strong, long horns. These horns typically form a spiral and horizontal extension (SHE-type) (**Figure 5a**). The SHE horns are clearly different from the horns of European wild sheep (*Ovis orientalis*, *Ovis musimon*), which are regarded as the ancestor of modern domestic sheep in China [14] (**Supplementary Figure S8**). In contrast, horns of VT, BY, MGS and EUS are either polled or curled tightly close to the face (TCF-type) (**Figure 5a**). To figure out whether *RXFP2* haplotype directly affected the appearance of horns, we tested their correlation in an independent PT population (n = 182) with heterogeneous horn types. This population consists of 138 SHE-type horned, 16 TCF-type horned, 14 scurred (small and undeveloped horns), 11 polled sheep, as well as three individuals with uncertain horn type (**Supplementary Figure S11**, **Supplementary Table S24**). Regression models were applied to identify potential association between horn phenotypes (horn size, horn shape) and eight *RXFP2*-linked SNPs, which included two protein-altering, two intronic SNPs with high $F_{ST}$ in semi-feral TBS and four previously reported trait-associated SNPs in other sheep populations (**Supplementary Table S25**).

In the 182 PT sheep, we observed strong associations between horn sizes and three SNPs we identified based on $F_{ST}$ (OAR10_29461968, OAR10_29491062, OAR10_29461717), while the four previously reported SNPs showed either minor or no effect (**Figure 5b**). The highest correlation among all eight SNPs was found at one of the protein-altering SNPs (OAR10_29461968), where each copy of T allele gave rise to about 11.75 cm increase in horn length ($P$ = $4.78\times10^{-23}$) (**Figure 5c**). By analyzing covariates in the regression model, we confirmed that this correlation was

independent from individual age and sex, which potentially affected horn size (regarding covariates, $P$ = $1.75\times10^{-27}$) (**Supplementary Figure S12**). Furthermore, we identified the same SNP OAR10_29461968, rather than previously reported SNPs, strongly correlated with horn shape (**Figure 5b**). OAR10_29461968:T homozygotes were found to be overrepresented in SHE-type horned sheep relative to TCF-type individuals ($P$ = $2.20\times10^{-7}$) (**Figure 5d**), in agreement with the shape distribution across different populations. These findings supported that the unique *RXFP2* haplotype we identified was responsible for the horn-phenotype differences between semi-feral and domestic populations in Chinese sheep.

### *RXFP2* gene expression in sheep horns

Although a solid association has been identified between the haplotype over the *RXFP2* locus and horn phenotypes, it is possible that causal variants might actually affect other flanking genes in respect of regulatory region alterations. To confirm the functional relevant gene for horns of TBS, we studied the expression patterns among different PT sheep tissues of all functional genes (*RXFP2*, *B3GLCT*, *FRY*, LOC101110773, LOC106991357) annotated within a ~1Mb region encompassing *RXFP2*. The region was comprised of multiple LD blocks (**Supplementary Figure S13**), which covered potential hitchhiking variants associated with the sweep. Interestingly, among all five studied genes, only *RXFP2* exhibited a pattern of tissue-specific expression in soft horn and horn periosteum (**Figure 6a,**

**Supplementary Figure S14**). This tissue-expression pattern was also confirmed in samples from Sonid sheep (**Supplementary Figure S15**).

We next compared the gene expression in the soft-horn tissues of SHE-type, TCF-type and scurred PT sheep. Despite obvious individual variations, a relatively lower expression of *RXFP2* was found in SHE than in TCF ($P$ < 0.001) and scurred samples ($P$ < 0.001) (**Figure 6b**). Moreover, *RXFP2* expression was negatively correlated with horn size (Pearson's $r$ = -0.76, $P$ = 0.002) (**Figure 6c**). No obvious correlations between flanking gene expressions and horn phenotypes were observed (**Supplementary Figure S16-S17**). Furthermore, the *RXFP2* protein levels in soft-horn tissues were examined by western blotting, which revealed a consistent reduction of translation product in SHE-type horns (**Figure 6d**), in agreement with the mRNA expressions.

## Discussion

Feralization is the process where domestic animals go back to the wild. Although its reverse – domestication – has been extensively studied [1-4], the genetic basis of feralization remains largely unsolved. In the present study, we performed a comprehensive survey of genetic diversity in Tibetan sheep. These animals are separated into various local breeds, within which a contrast of domestic and semi-feral status exists. Analysis of selective sweeps in two semi-feral populations compared with domestic lineages has revealed a critical role of semi-feralization in development of male reproductive success, muscle strength, eating habits, aggressive behaviors, defensive responses and sensory perception. Specifically, the semi-feral sheep have developed a unique horn phenotype as plausible adaptation to the reduced human intervention and increasing natural and sexual selection.

Horns are crucial for the survival of wild sheep because: (1) male individuals with strong horns show advantages in competition to reproduce; (2) aggressive horns are essential weapons against carnivorous enemies. In most domestic lines, horns become vestigial, because traits ensuring fitness in natural life are becoming useless under artificial breeding. However, domesticated Tibetan sheep would be an exception, as we found a special SHE-type horn, mediated by *RXFP2* gene, bringing about individual advantages in two semi-feral populations PT and OL. Firstly, PT and OL represented by their SHE-type horns, contained a special *RXFP2* haplotype with strong signals of selective sweeps compared with VT and MGS. Secondly, an association study within a PT population suggested that the *RXFP2* haplotype was

significantly associated with horn size and shape. Thirdly, gene expression analysis in 13 tissues of PT sheep demonstrated that *RXFP2* was the only gene specifically expressed in horn-related tissues and exhibiting decreased expression in SHE-type-horned individuals. Unlike in other pastoral areas, the breeding of Tibetan sheep is less affected by human intervention, and most importantly, the mating is relatively random (e.g. opposite sexes are kept separately for most MGS, but not TBS) (**Supplementary Table S2**). PT and OL live in an area with sparse human populations (**Supplementary Figure S7**), suggesting little artificial impact and more threats from the wild (e.g. more wolves were observed in sparsely populated regions of Tibet). Therefore, large and aggressive horns will bring about advantages for PT and OL, just as in wild and other feral populations [17].

An interesting phenomenon is that SHE-type horns (with a spiral and twisted shape) seem to also exist in other sheep breeds out of China, such as the Hungarian Racka sheep (**Supplementary Figure S18**). It is not clear whether there were genetic introgressions related with this phenotype from other ovine lineages into TBS, or vice versa. It is also possible that the *RXFP2* genotype related with SHE horns was newly derived in TBS. These possibilities can be tested in the future when genotypic data from more sheep breeds is generated.

*RXFP2* is a well-known genetic determinant of horn phenotype in sheep. This locus is correlated with quantitative and discrete traits of horns in wild and feral populations [16, 17, 34]. For domestic sheep, SNPs within or around *RXFP2* are predictive for polledness [35-37]. Although the contribution of *RXFP2* to horn

phenotypes have been extensively studied [17, 38, 39], little is known about the mechanism accounting for the various outcomes of sheep horns. Our study confirmed *RXFP2* to be the functional gene responsible for the special horn shape observed in semi-feral breeds PT and OL. The expression patterns of *RXFP2* among horn types, which we identified, provided novel evidence to understand the genetic basis underlying the growth of horns, as well as polledness. As in cattle, understanding polledness of sheep is crucial because it improves the welfare of animals and protects their handlers [40]. Additional efforts are required to clarify the role of *RXFP2* in development of diverse sheep horns.

In conclusion, the present study revealed a Tibetan sheep sub-population that has been subject to alterations in genomic loci related with its semi-feralization. These sheep have undergone rapid evolution across the *RXFP2* gene to acquire strong and weapons-grade horns, as a consequence of sexual selection and reduced human intervention. Our study highlighted the importance of human activities in adaptive evolution of domestic animals and provided a novel insight into their processes of semi-feralization.

# Methods

## Sample collection and sequencing

A total of 89 Chinese sheep from nine diverse breeds, as well as 10 Australian Merino sheep from Australia were sequenced in the present study (**Supplementary Table S1-S2**). For each sheep, genomic DNA was extracted from 200 µl of peripheral venous blood using the QIAamp DNA Blood Mini Kit (Qiagen, Germany). The quality and integrity of the DNA was assessed using the A260/280 ratio and agarose gel electrophoresis. For sequencing library preparation, the genomic DNA was sheared to fragments of 300-400 bp and subsequently end-repaired, 'A'-tailed and ligated to Illumina sequencing adapters. The ligated products with sizes of 400-500 bp were selected on 2% agarose gels and subsequently amplified by ligation mediated PCR (LM-PCR). The libraries were sequenced on an Illumina HiSeq 2500 sequencer in 2 × 100 bp paired-end mode and controlled using Illumina HiSeq Control Software.

## Variant calling

The raw reads were processed using two steps of quality control (QC): (1) reads with adapter contamination were removed; and (2) reads with more than 10% ambiguous bases were excluded. Only paired reads were preserved after QC. The filtered reads were subsequently mapped to the sheep reference genome assembly oviAri3 [41] using BWA version 0.7.12 (BWA, RRID:SCR_010910) [42] for all individuals separately. PCR duplicates were removed using PICARD [43] (version

1.135). Indels were realigned using GATK version 3.2-2 (GATK, RRID:SCR_001876) [44]. SNPs and indels were called using SAMtools [42] (version 1.2) after pooling samples from the same breed. After SNP calling, the variants were filtered using vcfutil.pl varFilter, with a “-d 20 -D 100” parameter to remove low-quality SNPs and indels. Targeted sequencing (Sanger) of five random genomic regions were performed, and the results were utilized to estimate the false positive (FPR) and false negative rate (FNR) of SNP calling step (**Supplementary Table S26-S27**). After filtering, the variants were annotated using snpEff [45] (version 4.0e) according to the NCBI annotation [46].

**Population genetics analysis**

Pair-wise genetic distances were measured by the number of allele differences for genomic SNP sites. The neighbor-joining tree was calculated based on the distance matrix using PHYLIP [47] (version 3.69). To place a root for the phylogeny tree, we aligned the goat genome sequence [48] with the sheep reference genome sequence using LASTZ [49] (version 1.02) and used the homologous sites of goat to determine the ancestral alleles for each SNP. Only biallelic autosomal SNPs were used to calculate the distance matrix. PCA was performed using EIGENSOFT [50, 51] (version 6.0.1), and population structures were inferred using FRAPPE software [52] (version 1.1). Both the PCA and population structures were calculated based on autosomal SNPs after removing highly correlated SNP pairs using PLINK version

1.07 (PLINK, RRID:SCR_001757) [53] with the "-indep-pairwise 50 5 0.2" parameter. Migration events among sheep breeds were estimated using TreeMix [15] with migration number $m$ = 0-5. Statistics including $\pi$ (pair-wise nucleotide differences), $\theta$ (number of segregating sites), SNP densities and Tajima's $D$, were calculated using VCFtools [54] (v0.1.12b). The linkage disequilibrium $r^2$ was calculated using Haploview [55] based on 500,000 SNPs randomly selected from the genome. The parameters were set as "--missingCutoff 0.2 --dprime --minMAF 0.1". The SNP pairs were grouped according to the physical distances between them. The mean $r^2$ was adopted to represent the average LD for each group (e.g., 0~1 kb).

## Selective sweep analysis

Selective sweeps across the sheep genome in four populations PT, OL, VT and BY were detected by comparison with MGS, based on fixation index $F_{ST}$ and heterozygosity $\log_2(H_P$ ratio) over a 30-kb sliding window with a step of 15 kb. $F_{ST}$ distances between population were calculated using the Bio::PopGen::PopStats package in BioPerl [56]. The pooled heterozygosity $H_P$ for population was calculated using the formula $H = 2\sum p\sum q/(\sum p+\sum q)^2$, where $\sum p$ represents the sum of the major allele frequencies of all SNP sites in the window and $\sum q$ represents sum of the minor allele frequencies [4]. The $\log_2(H_P$ ratio) between population A and B was calculated as $\log_2(H_{P|B}/H_{P|A})$, which reflected the loss of heterozygosity in A relative to B. 46 windows were excluded because of extremely small variant numbers (< 50 variants)

(**Supplementary Table S28**). We considered the windows with top 5% values as the significance threshold for single statistic (e.g. $F_{ST|A\ vs.\ B} > F_{ST|5\%}$, where $F_{ST|5\%}$ denotes the top 5% threshold of $F_{ST|A\ vs.\ B}$). A 30-kb region was defined as a selective sweep in population A if it had both $F_{ST|A\ vs.\ MGS}$ and $\log_2(H_{P|MGS}/H_{P|A})$ over the threshold.

All annotated genes overlapped with sweep windows or their flanking windows (15-kb up- and down-stream the sweep region) were defined as candidate genes. Furthermore, the cross-population extended haplotype homozygosity (XP-EHH) [53] was estimated between PT vs. MGS, OL vs. MGS, VT vs. MGS and BY vs. MGS for candidate sweep region, based on haplotype data phased by fastPHASE [57]. GO functional enrichment analysis of the candidate genes was performed using ClueGO [58], in which the *P* values were corrected using the Benjamini-Hochberg approach (**Supplementary Table S13-18**). Protein-altering mutations were extracted from selective sweep windows to identify potential functional variants (**Supplementary Table S20-23**).

**Validation of SNP genotypes in large population**

We collected 1,155 additional venous jugular blood samples from sheep of 10 different breeds, including 100 AM sheep, 100 PT sheep, 98 VT sheep, 87 OL sheep, 100 BY sheep, 80 CB sheep, 100 H sheep, 100 T sheep, 100 WZ sheep and 290 STH sheep. Genomic DNA was extracted using the phenol-chloroform method and dissolved in TE buffer (10 mM Tris-HCl [pH 8.0] and 1 mM EDTA [pH 8.0]). To

validate the allele frequency of the two differentiated protein altering SNPs in *RXFP2* (**Supplementary Table S20-21**), we performed a multiplex screening assay (SNaPshot) [59] on these 1,155 individuals. We designed amplification and SNaPshot Single-base extension primers **(Supplementary Table S29)**. Genotyping was performed using the SNaPshot™ Multiplex Kit (ABI) according to the manufacturer's instructions and analyzed using the ABI Genetic Analyzer 3730XL.

**Association study between the *RXFP2* genotype and horn phenotypes**

Nine SNPs within or near *RXFP2* locus (**Supplementary Table S25**) were genotype in 182 PT sheep with five horn types: polled (0 cm), scurred (0-12 cm), TCF-type (>12 cm, tightly close to the face), SHE-type (>12 cm, spiral and horizontally extended) and uncertain-type (>12 cm, uncertain shape) (**Supplementary Table S24**). One out of the nine SNPs was ignored because no variations was observed among those sheep. Correlations between SNP genotypes and horn phenotypes (horn size, horn shape) were estimated using linear or logistic regressions (performed with in-house R language scripts), depending on the variable type of outcome. Three different genetic models (recessive, additive and dominant) were applied for each pair of association test. Confounding effects of individual age and sex were tested by considering them as covariates in the model.

**Gene expression analysis of *RXFP2* and its flanking genes**

Tissue expression levels of the five genes located within the 1Mb region encompassing *RXFP2* locus, including *RXFP2*, *B3GLCT*, *FRY*, LOC101110773 (*EF1A1L*) and LOC106991357 (ncRNA) were examined by RT-PCR (**Supplementary Figure S16-S17**). Primer sequences were shown in **Supplementary Table S29**. We studied 13 tissues of TBS and 21 tissues of Sonid sheep. For each tissue type, equal volume of cDNA from six individuals (two individuals from each horn type) were mixed as pooled cDNA samples. RT-PCR reactions were carried out in 50 µl volume including Taq DNA polymerase(5U/µl) (TaKaRa, Dalian, China) 0.25 µl, 10×PCR Buffer(+$MgCl_2$) 5µl, 10mM dNTPs (2.5mM each) 4µl , each primer (10 µM) 1 µl, cDNA 1µl, dd$H_2O$ 37.75 µl. Amplification conditions were set as: initial denaturation at 95°C for 5 min, followed by 33 cycles of denaturation at 95°C for 30 s, annealing for 20 s at appropriate temperatures, extension at 72°C for 10 s; with a final extension at 72°C for 2 min on Mastercycler 5333 (Eppendorf AG, Hamburg, Germany). The PCR product mixture with 5µl loading buffer (6×), and loaded 5µl into 1% sepharose gel. After 15min 180mA electrophoresis, take a picture under Biorad GelDoc XR System (Bio-rad, USA).

Expressions of the five genes were measured by real-time PCR in 13 PT sheep soft-horn samples with different horn types. Four to five biological replicates were selected from different individuals of same horn type (four SHE-type, four TCF-type, and five scurred). For all five genes and internal control, real-time PCR was performed three times in one sample as technical replicates, and the average gene expressions of three replicates were calculated. Real-time PCR amplification was

performed in a 20-μl of reaction mixture containing 2 μl of cDNA, 0.4 μl of each forward and reverse primer (10 μM), 0.4 μl of ROX Reference Dye II (50×), 10 μl of SYBR Green Real-time PCR Master Mix (2×), and 6.8 μl of $ddH_2O$. The reaction without template was treated as blank control. PCR amplification was performed in triplicate wells using the following conditions: 95ºC for 30 s, followed by 40 cycles of 95ºC for 5 s and 60ºC for 34 s. The melting curve was analyzed after amplification. The peak Tm on the dissociation curve was used to determine the specificity of PCR amplification. Standard curves of these genes were also constructed. *β*-actin expressions were used as internal control among samples. Relative expression levels of 5 genes were calculated based on the expression of *RXFP2* in the SHE-type soft horn (its expression was defined as 1.0). The $2^{-\Delta\Delta Ct}$ method was used to process the real-time PCR results [60].

Protein extracts from soft-horn tissues were prepared by complete homogenization of tissues in an immunoprecipitation buffer (Beyotime, CA) according to the manufacturer's instructions. Equal amounts of protein extracts were mixed with sample buffer and then separated on 10% SDS-PAGE gels (60 μg/lane). Details of the western blotting process were described previously [61]. Rabbit Anti-GPR106 antibody (BIOSS, Beijing, China), polyclonal rabbit anti-mouse *β*-actin antibody (Abcam, US) and goat anti-rabbit IgG, HRP (Santa Clara, CA, USA) were used.

## Availability of supporting data

Raw sequence data has been submitted to the NCBI Sequence Read Archive (SRA) under accession number SRP066883. Genotypic data of 99 individuals has been submitted to the European Variation Archive (EVA) under accession number ERZ480291 (Project ID: PRJEB23437). Supporting data, including variant files (VCF), phylogenetic tree files, pictures of individual sheep, gel images and perl scripts are available via the *GigaScience* repository GigaDB[62].

## Declarations

### List of abbreviations

TBS, Tibetan sheep; MGS, Mongolian sheep; EUS, European sheep; PT, Prairie Tibetan sheep; VT, Valley Tibetan sheep; OL, Oula sheep; BY, Bayinbuluke sheep; CB, Cele Black sheep; H, Hu sheep; T, Tan sheep; STH, Small Tail Han sheep; WZ, Wuzhumuqin sheep; AM, Australian Merino sheep; WGS, whole-genome sequencing; indel, insertion and deletion; NJ, neighbor-joining; PCA, principal component analysis; LD, linkage disequilibrium; GO, gene ontology; SNP, single nucleotide polymorphism; SHE, spirally and horizontally extended; TCF, tightly close to the face.

### Consent for publication

Not applicable.

## Ethic approval

All experimental procedures involving animals were approved by the Chinese Ministry of Agriculture, the animal care and use committee at the institution where the experiments were performed.

## Competing interests

The authors declared no competing interests.

## Fundings

This work was supported by the Agricultural Science and Technology Innovation Program of China (ASTIP-IAS13), the Earmarked Fund for China Agriculture Research System (CARS-39), the National Key Technology Support Program (2013BAI101B09), the National Natural Science Foundation of China (31472078 and 31402041), the National Key Scientific Instrument and Equipment Development Project (2012YQ03026108), the National Basic Research Program of China (2011CB910204, 2011CB510102), the Youth Innovation Promotion Association CAS (2017325) and the Genetically Modified Organisms Breeding Major Program of China (2016ZX08009-003-006 and 2016ZX08010-005-003), by Major Science and

Technology Program of Inner Mongolia Autonomous Region of China.

## Authors' contributions

YX.L., MX.C. designed and supervised the project. ZY.P., SD.L., QY.L. provided further conceptions based on original design. ZY.P., QY.L. collected and generated the data. SD.L., Z.W., BP.M. performed bioinformatics analysis. Z.X., DK.W., LY.Y., RC.W., YQ.Z. supported data analysis. WP.H., XY.W., XX. H., G.H., J.Y., C.L., M.J., YJ.Z. provided samples. ZY.P., XY.H., XF.G., BM.L., XY.L., XH.C., XL.D., Q.X., HC.S., FK.L. performed experimental validations. ZK.Z., GH.D., SJ.L. supportively supervised the project. SD.L., ZY.P. drafted the original manuscript. YX.L., MX.C., Z.W., QY.L., ZK.Z., R.D. edited the manuscript. All authors reviewed the final version of manuscript.

## Acknowledgements

The authors thank Ori-Gene Technology Co., Ltd. Beijing, China, for their contributions in sample preparations.

# References

1. Frantz LAF, Schraiber JG, Madsen O, Megens HJ, Cagan A, Bosse M, et al. Evidence of long-term gene flow and selection during domestication from analyses of Eurasian wild and domestic pig genomes. Nat Genet. 2015;47 10:1141-+.

2. Axelsson E, Ratnakumar A, Arendt ML, Maqbool K, Webster MT, Perloski M, et al. The genomic signature of dog domestication reveals adaptation to a starch-rich diet. Nature. 2013;495 7441:360-4.

3. Carneiro M, Rubin CJ, Di Palma F, Albert FW, Alfoldi J, Barrio AM, et al. Rabbit genome analysis reveals a polygenic basis for phenotypic change during domestication. Science. 2014;345 6200:1074-9.

4. Rubin CJ, Zody MC, Eriksson J, Meadows JR, Sherwood E, Webster MT, et al. Whole-genome resequencing reveals loci under selection during chicken domestication. Nature. 2010;464 7288:587-91. doi:10.1038/nature08832.

5. Callaway E. When Chickens Go Wild. Nature. 2016;529 7586:270-3.

6. Chen FH, Dong GH, Zhang DJ, Liu XY, Jia X, An CB, et al. Agriculture facilitated permanent human occupation of the Tibetan Plateau after 3600 B.P. Science. 2015;347 6219:248-50. doi:10.1126/science.1259172.

7. Yang X, Scuderi LA, Wang X, Scuderi LJ, Zhang D, Li H, et al. Groundwater sapping as the cause of irreversible desertification of Hunshandake Sandy Lands, Inner Mongolia, northern China. Proceedings of the National Academy of Sciences of the United States of America. 2015;112 3:702-6. doi:10.1073/pnas.1418090112.

8. Zhao YX, Yang J, Lv FH, Hu XJ, Xie XL, Zhang M, et al. Genomic Reconstruction of the History of Native Sheep Reveals the Peopling Patterns of Nomads and the Expansion of Early Pastoralism in East Asia. Molecular biology and evolution. 2017;34 9:2380-95. doi:10.1093/molbev/msx181.

9. Yang J, Li WR, Lv FH, He SG, Tian SL, Peng WF, et al. Whole-genome sequencing of native sheep provides insights into rapid adaptations to extreme environments. Molecular biology and evolution. 2016;33:2576-92. doi:10.1093/molbev/msw129.

10. Zhong T, Han JL, Guo J, Zhao QJ, Fu BL, He XH, et al. Genetic diversity of Chinese indigenous sheep breeds inferred from microsatellite markers. Small Ruminant Res. 2010;90 1-3:88-94.

11. Tu YR. The Sheep and Goat Breeds in China. Shanghai Science and Technology Press; 1989. p. 6-19.

12. Ai H, Fang X, Yang B, Huang Z, Chen H, Mao L, et al. Adaptation and possible ancient interspecies introgression in pigs identified by whole-genome sequencing. Nat Genet. 2015;47 3:217-25. doi:10.1038/ng.3199.

13. Gou X, Wang Z, Li N, Qiu F, Xu Z, Yan D, et al. Whole-genome sequencing of six dog

breeds from continuous altitudes reveals adaptation to high-altitude hypoxia. Genome Research. 2014;24 8:1308-15. doi:10.1101/gr.171876.113.

14. Lv FH, Peng WF, Yang J, Zhao YX, Li WR, Liu MJ, et al. Mitogenomic meta-analysis identifies two phases of migration in the history of eastern eurasian sheep. Molecular biology and evolution. 2015;32 10:2515-33. doi:10.1093/molbev/msv139.

15. Pickrell JK and Pritchard JK. Inference of population splits and mixtures from genome-wide allele frequency data. PloS Genetics. 2012;8 11:e1002967. doi:10.1371/journal.pgen.1002967.

16. Johnston SE, Gratten J, Berenos C, Pilkington JG, Clutton-Brock TH, Pemberton JM, et al. Life history trade-offs at a single locus maintain sexually selected genetic variation. Nature. 2013;502 7469:93-5. doi:10.1038/nature12489.

17. Kardos M, Luikart G, Bunch R, Dewey S, Edwards W, McWilliam S, et al. Whole-genome resequencing uncovers molecular signatures of natural and sexual selection in wild bighorn sheep. Mol Ecol. 2015;24 22:5616-32. doi:10.1111/mec.13415.

18. Markakis MN, Soedring VE, Dantzer V, Christensen K and Anistoroaei R. Association of MITF gene with hearing and pigmentation phenotype in Hedlund white American mink (Neovison vison). Journal Of Genetics. 2014;93 2:477-81.

19. Chen L, Guo W, Ren L, Yang M, Zhao Y, Guo Z, et al. A de novo silencer causes elimination of MITF-M expression and profound hearing loss in pigs. BMC Biol. 2016;14:52. doi:10.1186/s12915-016-0273-2.

20. Tsukamoto K, Suzuki H, Harada D, Namba A, Abe S and Usami S. Distribution and frequencies of PDS (SLC26A4) mutations in Pendred syndrome and nonsyndromic hearing loss associated with enlarged vestibular aqueduct: a unique spectrum of mutations in Japanese. European Journal Of Human Genetics. 2003;11 12:916-22. doi:10.1038/sj.ejhg.5201073.

21. Shen X, Liu F, Wang Y, Wang H, Ma J, Xia W, et al. Down-regulation of msrb3 and destruction of normal auditory system development through hair cell apoptosis in zebrafish. International Journal Of Developmental Biology. 2015;59 4-6:195-203. doi:10.1387/ijdb.140200md.

22. Ahmed ZM, Yousaf R, Lee BC, Khan SN, Lee S, Lee K, et al. Functional null mutations of MSRB3 encoding methionine sulfoxide reductase are associated with human deafness DFNB74. American Journal Of Human Genetics. 2011;88 1:19-29. doi:10.1016/j.ajhg.2010.11.010.

23. Ni C, Zhang D, Beyer LA, Halsey KE, Fukui H, Raphael Y, et al. Hearing dysfunction in heterozygous Mitf(Mi-wh) /+ mice, a model for Waardenburg syndrome type 2 and Tietz syndrome. Pigment Cell Melanoma Res. 2013;26 1:78-87. doi:10.1111/pcmr.12030.

24. Park HJ, Shaukat S, Liu XZ, Hahn SH, Naz S, Ghosh M, et al. Origins and frequencies of SLC26A4 (PDS) mutations in east and south Asians: global implications for the epidemiology of deafness. Journal Of Medical Genetics. 2003;40 4:242-8.

25. Pryor SP, Madeo AC, Reynolds JC, Sarlis NJ, Arnos KS, Nance WE, et al. SLC26A4/PDS

genotype-phenotype correlation in hearing loss with enlargement of the vestibular aqueduct (EVA): evidence that Pendred syndrome and non-syndromic EVA are distinct clinical and genetic entities. Journal Of Medical Genetics. 2005;42 2:159-65. doi:10.1136/jmg.2004.024208.

26. Mier P and J P-PA. Fungal Smn and Spf30 homologues are mainly present in filamentous fungi and genomes with many introns: Implications for spinal muscular atrophy. Gene. 2012; 491 2:135-41.

27. Talbot K, Miguel-Aliaga I, Mohaghegh P, Ponting CP and Davies KE. Characterization of a gene encoding survival motor neuron (SMN)-related protein, a constituent of the spliceosome complex. Human Molecular Genetics. 1998;7 13:2149-56. doi:ddb265 [pii].

28. Jiang Y, Xie M, Chen W, Talbot R, Maddox JF, Faraut T, et al. The sheep genome illuminates biology of the rumen and lipid metabolism. Science. 2014;344 6188:1168-73.

29. Hayes BJ, Pryce J, Chamberlain AJ, Bowman PJ and Goddard ME. Genetic Architecture of Complex Traits and Accuracy of Genomic Prediction: Coat Colour, Milk-Fat Percentage, and Type in Holstein Cattle as Contrasting Model Traits. Plos Genetics. 2010;6 9.

30. Schmutz SM and Berryere TG. Genes affecting coat colour and pattern in domestic dogs: a review. Anim Genet. 2007;38 6:539-49.

31. Moore KJ. Insight into the Microphthalmia Gene. Trends Genet. 1995;11 11:442-8.

32. Wei C, Wang H, Liu G, Zhao F, Kijas JW, Ma Y, et al. Genome-wide analysis reveals adaptation to high altitudes in Tibetan sheep. Scientific reports. 2016;6:26770. doi:10.1038/srep26770.

33. Wang MS, Li Y, Peng MS, Zhong L, Wang ZJ, Li QY, et al. Genomic Analyses Reveal Potential Independent Adaptation to High Altitude in Tibetan Chickens. Molecular biology and evolution. 2015;32 7:1880-9.

34. Johnston SE, McEwan JC, Pickering NK, Kijas JW, Beraldi D, Pilkington JG, et al. Genome-wide association mapping identifies the genetic basis of discrete and quantitative variation in sexual weaponry in a wild sheep population. Mol Ecol. 2011;20 12:2555-66.

35. Dominik S, Henshall JM and Hayes BJ. A single nucleotide polymorphism on chromosome 10 is highly predictive for the polled phenotype in Australian Merino sheep. Anim Genet. 2012;43 4:468-70.

36. Wang XL, Zhou GX, Li Q, Zhao DF and Chen YL. Discovery of SNPs in RXFP2 related to horn types in sheep. Small Ruminant Res. 2014;116 2-3:133-6.

37. Wiedemar N and Drogemuller C. A 1.8-kb insertion in the 3-UTR of RXFP2 is associated with polledness in sheep. Anim Genet. 2015;46 4:457-61.

38. Johnston SE, McEwan JC, Pickering NK, Kijas JW, Beraldi D, Pilkington JG, et al. Genome-wide association mapping identifies the genetic basis of discrete and quantitative variation in sexual weaponry in a wild sheep population. Mol Ecol. 2011;20 12:2555-66. doi:10.1111/j.1365-294X.2011.05076.x.

39. Kijas JW, Lenstra JA, Hayes B, Boitard S, Porto Neto LR, San Cristobal M, et al. Genome-wide analysis of the world's sheep breeds reveals high levels of historic mixture and strong recent selection. PLoS Biol. 2012;10 2:e1001258. doi:10.1371/journal.pbio.1001258.

40. Carlson DF, Lancto CA, Zang B, Kim ES, Walton M, Oldeschulte D, et al. Production of hornless dairy cattle from genome-edited cell lines. Nature Biotechnology. 2016;34 5:479-81. doi:10.1038/nbt.3560.

41. Sheep reference genome assembly oviAri3. [ftp://ftp.ncbi.nlm.nih.gov/genomes/all/GCA_000298735.1_Oar_v3.1/GCA_000298735.1_Oar_v3.1_genomic.fna.gz](ftp://ftp.ncbi.nlm.nih.gov/genomes/all/GCA_000298735.1_Oar_v3.1/GCA_000298735.1_Oar_v3.1_genomic.fna.gz). Accessed 10 Oct 2015.

42. Li H and Durbin R. Fast and accurate short read alignment with Burrows-Wheeler transform. Bioinformatics. 2009;25 14:1754-60.

43. PICARD. [http://broadinstitute.github.io/picard/](http://broadinstitute.github.io/picard/).

44. McKenna A, Hanna M, Banks E, Sivachenko A, Cibulskis K, Kernytsky A, et al. The Genome Analysis Toolkit: a MapReduce framework for analyzing next-generation DNA sequencing data. Genome Research. 2010;20 9:1297-303. doi:10.1101/gr.107524.110.

45. Cingolani P, Platts A, Wang LL, Coon M, Nguyen T, Wang L, et al. A program for annotating and predicting the effects of single nucleotide polymorphisms, SnpEff: SNPs in the genome of Drosophila melanogaster strain w(1118); iso-2; iso-3. Fly. 2012;6 2:80-92.

46. NCBI annotation for sheep genome assembly oviAri3. [ftp://ftp.ncbi.nlm.nih.gov/genomes/Ovis_aries/GFF/ref_Oar_v3.1_scaffolds.gff3.gz](ftp://ftp.ncbi.nlm.nih.gov/genomes/Ovis_aries/GFF/ref_Oar_v3.1_scaffolds.gff3.gz). Accessed 10 Oct 2015.

47. Felsenstein J. PHYLIP - Phylogeny Inference Package (Version 3.2). Cladistics. 1989;5: 164-6.

48. Goat genome assembly CHIR 1.0. [ftp://ftp.ncbi.nlm.nih.gov/genomes/all/GCA_000317765.1_CHIR_1.0/GCA_000317765.1_CHIR_1.0_genomic.fna.gz](ftp://ftp.ncbi.nlm.nih.gov/genomes/all/GCA_000317765.1_CHIR_1.0/GCA_000317765.1_CHIR_1.0_genomic.fna.gz). Accessed 10 Oct 2015.

49. Harris RS. *Improved pairwise alignment of genomic DNA.* Improved pairwise alignment of genomic DNA. PhD Thesis, The Pennsylvania State University. PhD Thesis, 2007.

50. Patterson N, Price AL and Reich D. Population structure and eigenanalysis. PloS Genetics. 2006;2 12:2074-93. doi:10.1371/journal.pgen.0020190.

51. Price AL, Patterson NJ, Plenge RM, Weinblatt ME, Shadick NA and Reich D. Principal components analysis corrects for stratification in genome-wide association studies. Nat Genet. 2006;38 8:904-9. doi:10.1038/ng1847.

52. Tang H, Peng J, Wang P and Risch NJ. Estimation of individual admixture: Analytical and study design considerations. Genet Epidemiol. 2005;28 4:289-301. doi:10.1002/gepi.20064.

53. Sabeti PC, Varilly P, Fry B, Lohmueller J, Hostetter E, Cotsapas C, et al. Genome-wide detection and characterization of positive selection in human populations. Nature. 2007;449

7164:913-8. doi:10.1038/nature06250.

54. Danecek P, Auton A, Abecasis G, Albers CA, Banks E, DePristo MA, et al. The variant call format and VCFtools. Bioinformatics. 2011;27 15:2156-8. doi:10.1093/bioinformatics/btr330.

55. Barrett JC, Fry B, Maller J and Daly MJ. Haploview: analysis and visualization of LD and haplotype maps. Bioinformatics. 2005;21 2:263-5. doi:10.1093/bioinformatics/bth457.

56. Stajich JE, Block D, Boulez K, Brenner SE, Chervitz SA, Dagdigian C, et al. The Bioperl toolkit: Perl modules for the life sciences. Genome Research. 2002;12 10:1611-8. doi:10.1101/gr.361602.

57. Scheet P and Stephens M. A fast and flexible statistical model for large-scale population genotype data: applications to inferring missing genotypes and haplotypic phase. Am J Hum Genet. 2006;78 4:629-44. doi:10.1086/502802.

58. Bindea G, Mlecnik B, Hackl H, Charoentong P, Tosolini M, Kirilovsky A, et al. ClueGO: a Cytoscape plug-in to decipher functionally grouped gene ontology and pathway annotation networks. Bioinformatics. 2009;25 8:1091-3. doi:10.1093/bioinformatics/btp101.

59. Lovly CM, Dahlman KB, Fohn LE, Su Z, Dias-Santagata D, Hicks DJ, et al. Routine multiplex mutational profiling of melanomas enables enrollment in genotype-driven therapeutic trials. PLoS ONE. 2012;7 4:e35309.

60. Livak KJ and Schmittgen TD. Analysis of relative gene expression data using real-time quantitative PCR and the 2(T)(-Delta Delta C) method. Methods. 2001;25 4:402-8.

61. Zhang R, Rao M, Li C, Cao J, Meng Q, Zheng M, et al. Functional recombinant human anti-HAV antibody expressed in milk of transgenic mice. Transgenic Research. 2009;18 3:445-53. doi:10.1007/s11248-008-9241-0.

62. Pan Z, Li S, Liu Q, Wang Z, Zhou Z, Di R et al. Supporting data for ‘Whole-genome sequences of 89 Chinese sheep suggest role of RXFP2 in the development of unique horn phenotype as response to semi-feralization’. *GigaScience Database 2018.* http://dx.doi.org/10.5524/100408

## Figures and legends

**Figure 1. Genetic relationships and population structure in Chinese sheep.** (**a**) Geographical distribution of the Chinese indigenous sheep breeds (PT, Prairie Tibetan; OL, Oula; VT, Valley Tibetan; BY, Bayinbuluke; WZ, Wuzhumuqin; T, Tan; CB, Cele Black; STH, Small-tailed Han; H, Hu) and a European-originated breed (AM, Australian Merino) sampled in the present study. The background color of the sheep pictures represents their lineages (red: TBS, Tibetan sheep; blue: MGS, Mongolian sheep; green: EUS, European sheep). (**b**) Neighbor-joining tree of the ten breeds based on $F_{ST}$ distances. (**c**) Principal component plot. The first (PC1) and second (PC2) principal components are shown. (**d**) Population structure analysis of 99 sheep, where number of ancestral clusters were set from K = 2-4.

**Figure 2. Manhattan plot of genome-wide selective sweep signals ($F_{ST}$ and log-scaled $H_P$ ratio) in four sheep breeds.** For each metric, a 30-kb sliding window with a step size of 15kb was applied. $F_{ST}$ distances were calculated between each of the four breeds (PT, OL, VT or BY) vs. MGS (WZ, T, STH, H and CB). The log-scaled $H_P$ ratio was calculated as $-\log_2(H_{P|PT,\ OL,\ VT\ or\ BY}/H_{P|MGS})$, a positive value of which suggests reduction of variability in the breed.

**Figure 3. Candidate genes associated with selective sweeps in semi-feral sheep.** (**a**) A Venn plot showing numbers of overlapping candidate genes among four breeds (PT, OL, VT and BY). (**b**) A summary of feralization-related adaptation observed in

semi-feral sheep. Affected functional terms were manually summarized based on Gene Ontology (GO) enrichment analysis of the candidate genes, as well as literature mining. Numbers denote the count of candidate genes within each major category. (**c**) Sweep signal metrics for genes selected from feralization-related categories described in **Figure 2b**, as well as three genes associated with hypoxic adaptation.

**Figure 4. Selective sweep over the horn-related gene *RXFP2*.** (**a**) Statistics plotted over a ~400 kb region surrounding *RXFP2*, including: 1) population differentiation ($F_{ST}$) between PT, OL, VT and BY vs. MGS; 2) intra-population heterozygosity in PT, OL, VT and BY, calculated as Z-transformed $\log_2(H_{P|PT,\ OL,\ VT\ or\ BY}/H_{P|MGS})$; 3) haplotypic length measured by Z-transformed $\text{XP-EHH}_{PT,\ OL,\ VT\ or\ BY\ vs.\ MGS}$. (**b**) Haplotypic distributions among 99 sheep of a local region of *RXFP2* (chromosome 10: 29,400,000-29,550,000 bp). Biallelic SNPs were showed in blue and yellow. (**c**) Alignment of the *RXFP2* protein sequences from 9 vertebrate species. Two protein variants (*RXFP2*: 627 and 641) with top $F_{ST}$ in PT and OL are indicated in red. For 627 PT and OL have the variant allele, whereas for 641 they have the reference allele. The dots in the alignment denote amino acids that are identical with those in PT and OL. (**d**) Distribution of the haplotype frequency of two protein-altering variants (*RXFP2*: 627 and 641) in 1155 sheep. “Haplotype1” corresponds to V627 + E641 (OAR10_29461968:C + OAR10_29462010:C) and “Haplotype2” corresponds to M627 + K641 (OAR10_29461968:T + OAR10_29462010:T).

**Figure 5. *RXFP2* haplotype is correlated with horn shape and size.** (**a**) Features of

SHE-type and TCF-type horns. (**b**) Association between eight SNPs and horn phenotypes (size and shape) analyzed in 182 PT sheep; after testing all combinations of genetic models and confounding effects (**Supplementary Figure S12**), an additive model (assume *A* as major allele, *a* as minor allele, we have code 2 for *AA*, 1 for *Aa* and 0 for *aa*) was applied for horn size, and a recessive code (1 for *AA*, 0 for *Aa* and *aa*) was applied for horn shape; pair-wise LD between SNP pairs were plotted at the bottom, where numbers represent *D'* statistics. (**c**) Box-plot of individual horn sizes among different OAR_29461968 genotypes; P value was calculated by linear regression based on additive genetic model, and the fitting line was showed in red. (**d**) Distribution of OAR10_29461968 genotypes among PT sheep with different horn shapes.

**Figure 6. Gene expression patterns of *RXFP2*.** (**a**) Expression of *RXFP2* and *β-actin* in 13 tissue samples from PT sheep: 1, heart; 2, liver; 3, spleen; 4, lung; 5, kidney; 6, muscle; 7, brain; 8, ovary; 9, corpus uteri; 10, adipose; 11, thyroid; 12, soft horn; 13, horn periosteum. (**b**) Expression pattern of *RXFP2* in SHE-type, TCF-type, scurred soft-horn tissues examined by RT-PCR (left) and real-time PCR (right); error bars denote S.D. of the mean; groups with significant differences (*: $P < 0.05$; **: $P < 0.001$) were indicated. (**c**) Scatter plot on *RXFP2* expression and horn size; the fitting line of linear regression was showed in blue. (**d**) Western blot analysis of soft-horn tissues with different horn types, using antibodies of *RXFP2* and *β*-actin.

 Click here to download Figure Fig.1.revised2.pdf

# Figure 1

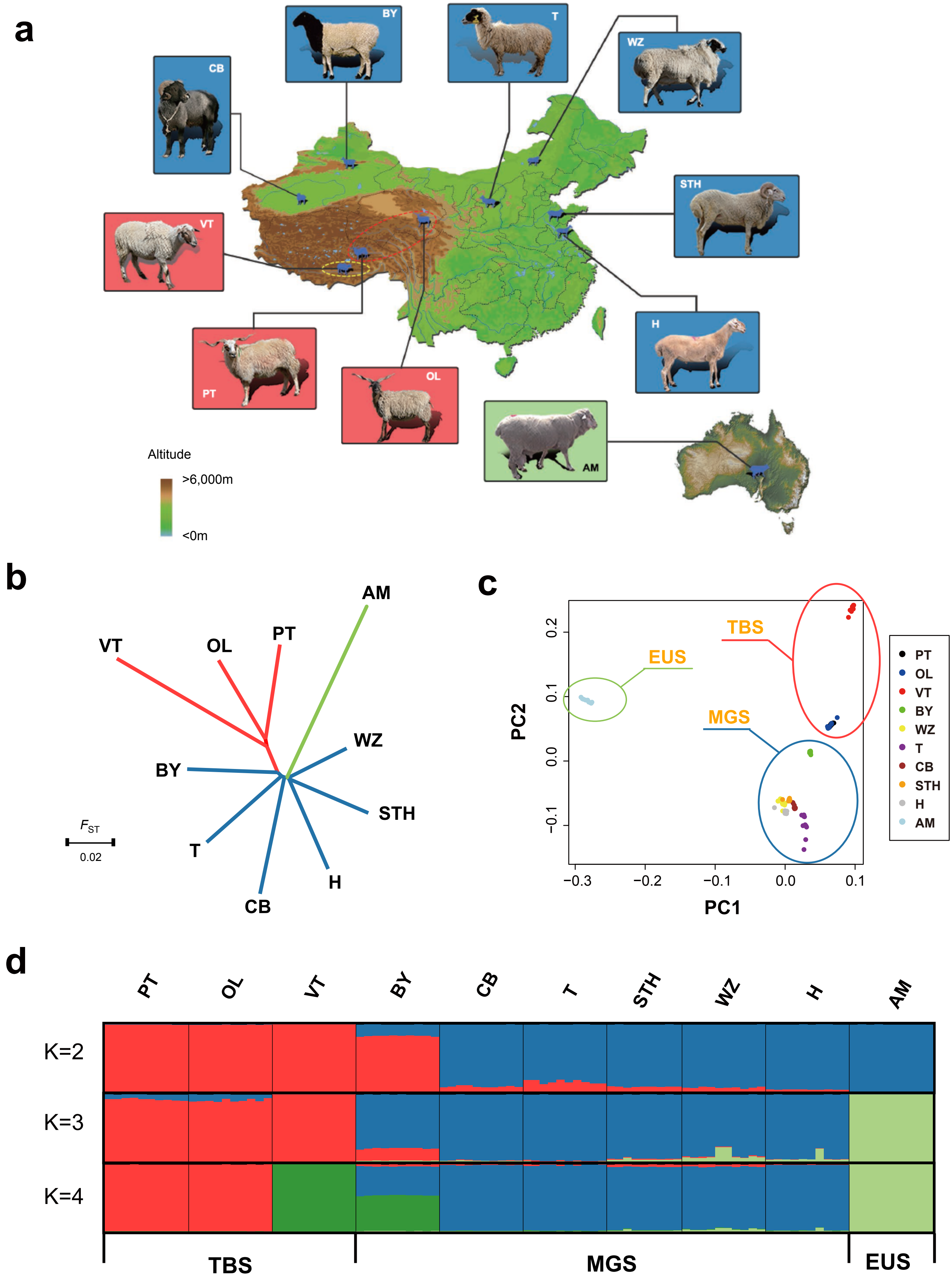

Click here to download Figure Fig.2.revised2.pdf

# Figure 2

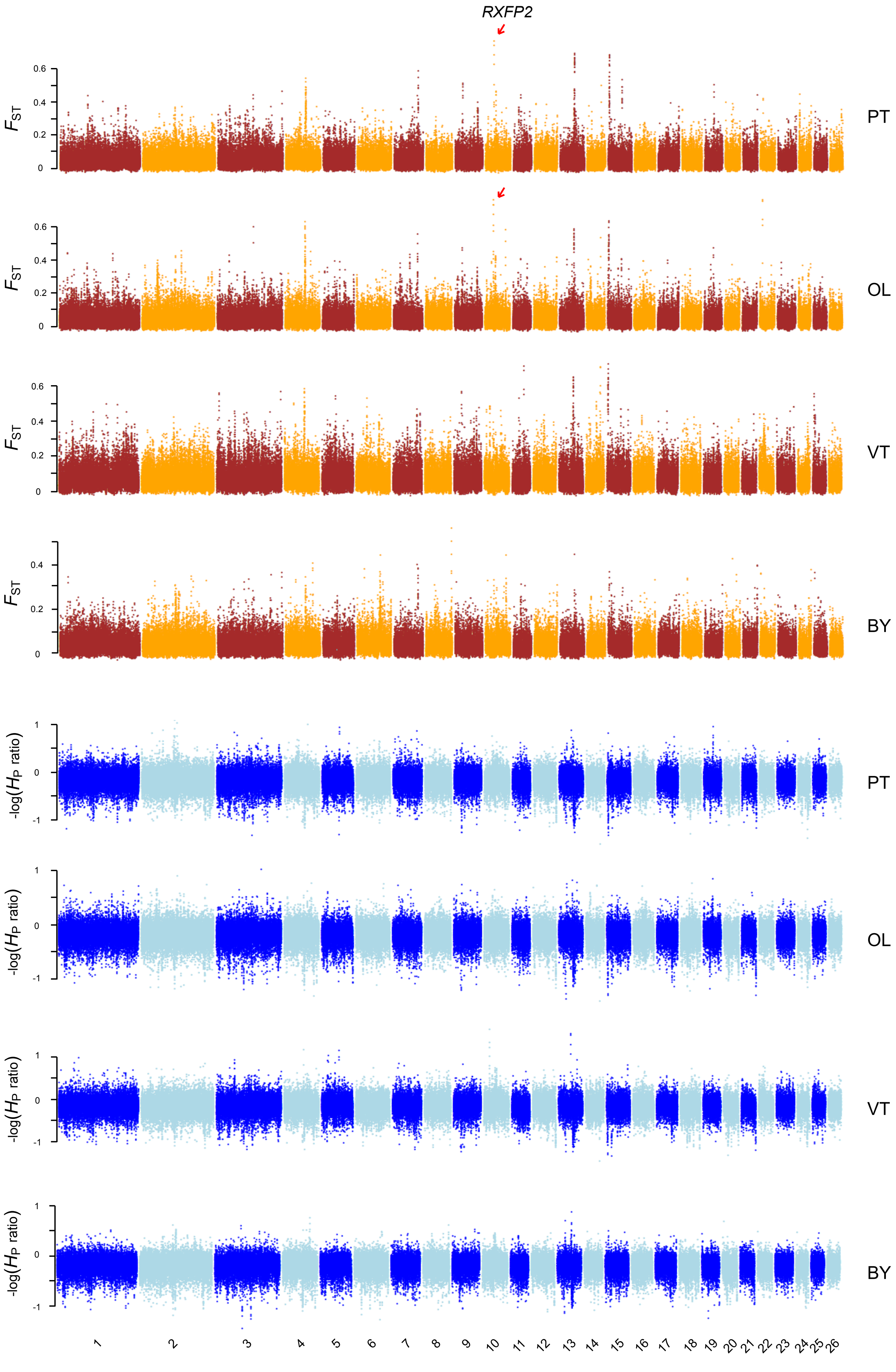

Figure 3

Click here to download Figure Fig.3.revised2.pdf

# Figure 3

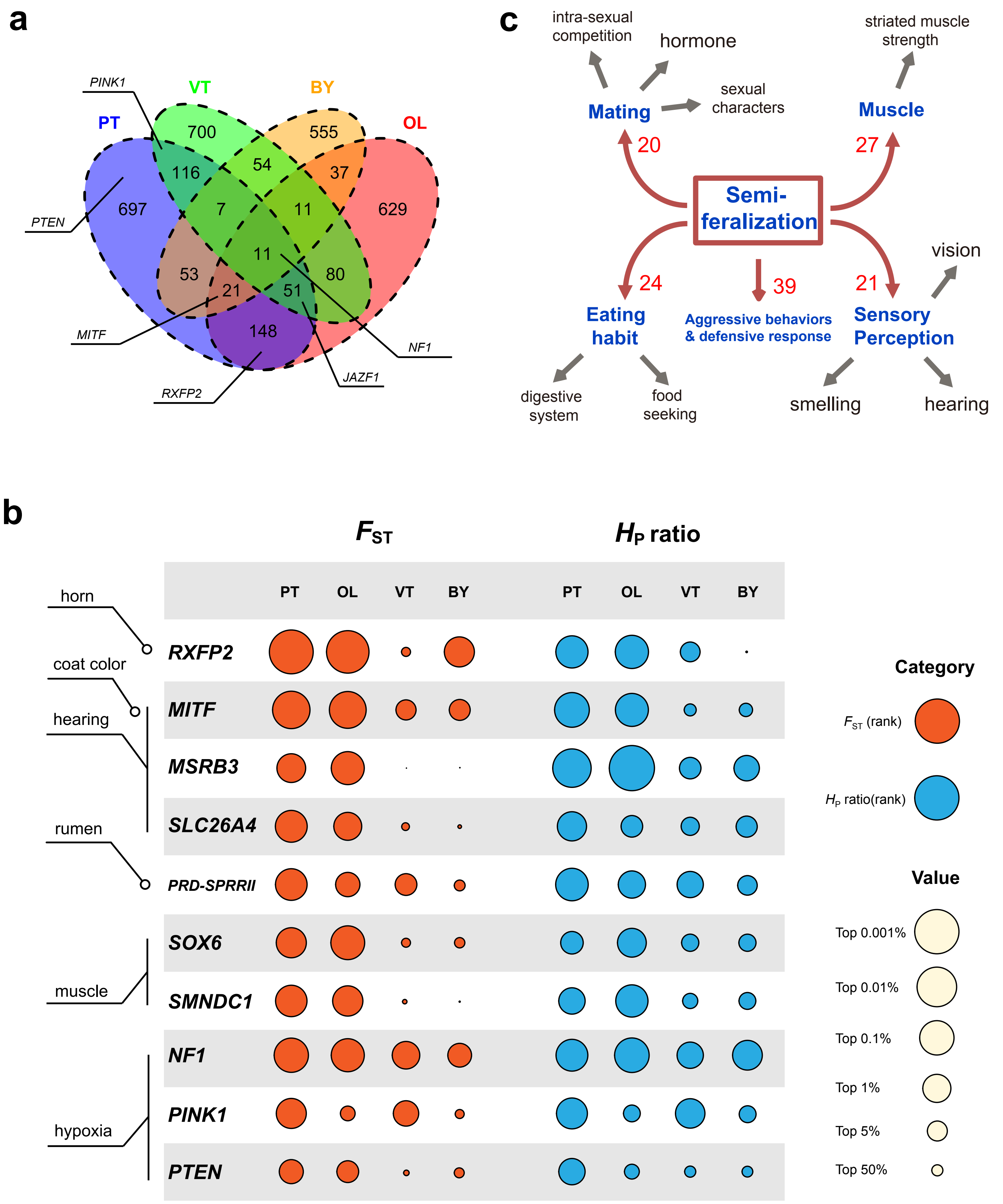

Figure 4
Click here to download Figure Fig.4.revised2.pdf

# Figure 4

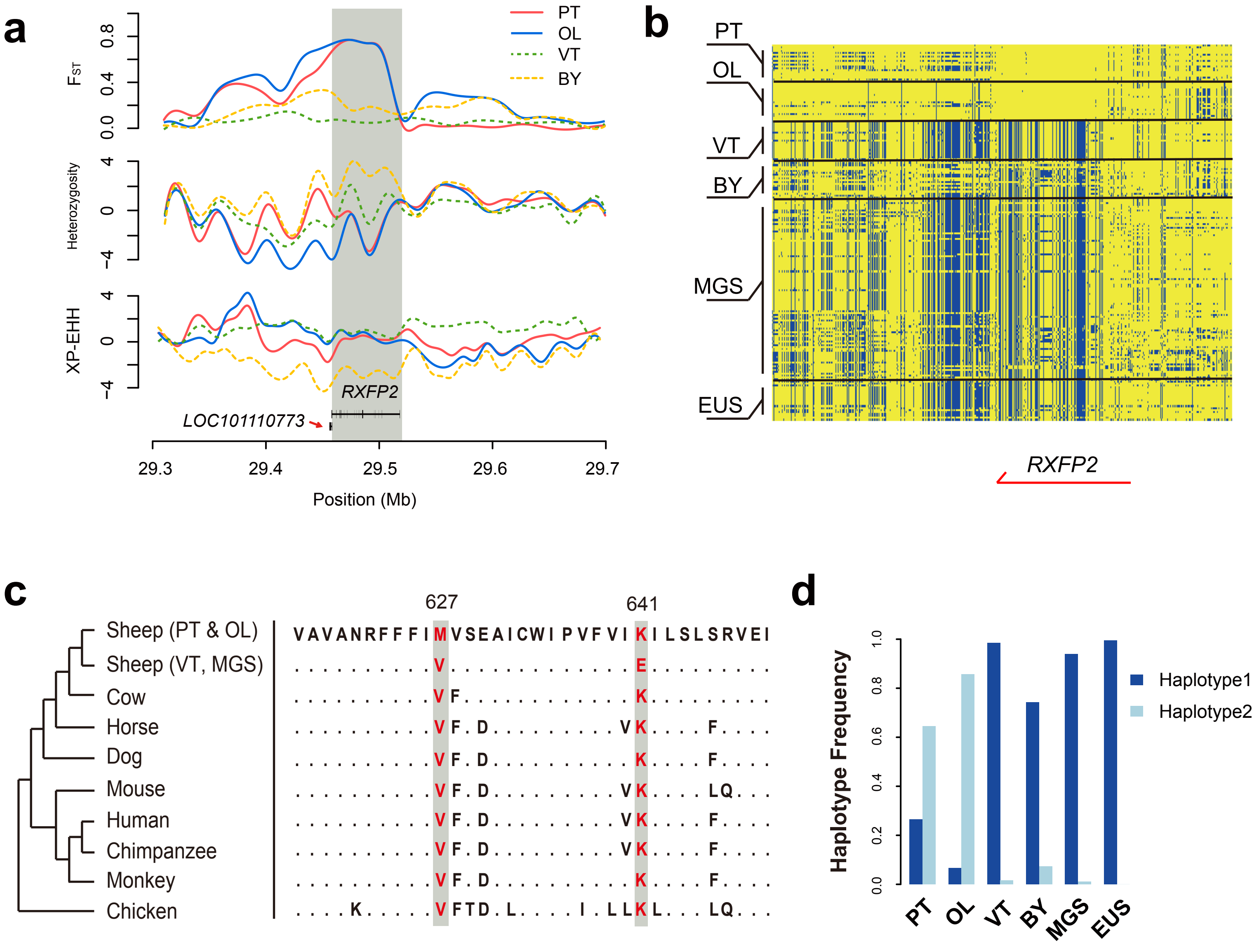

Figure 5

Click here to download Figure Fig.5.revised2.pdf

# Figure 5

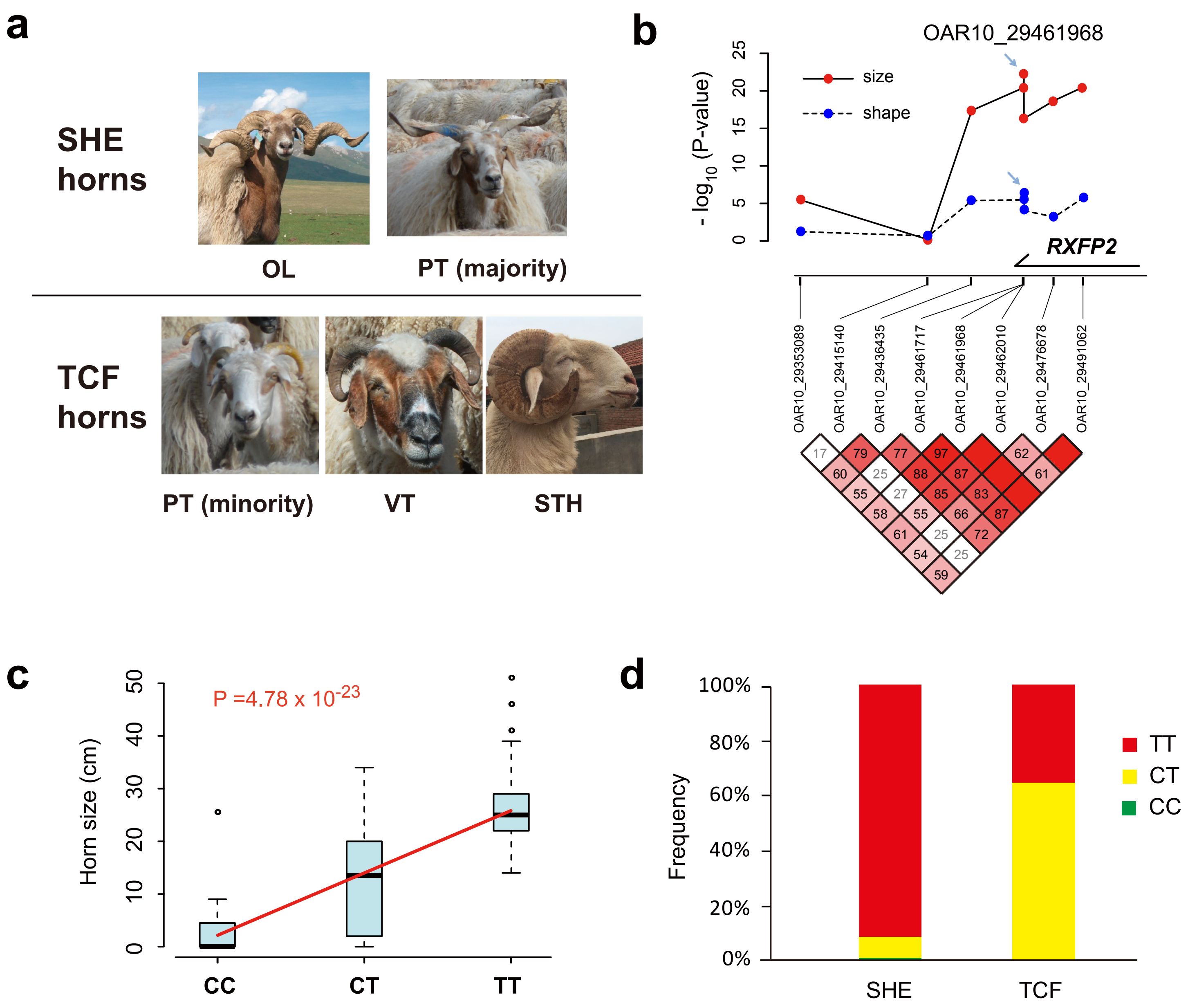

# Figure 6

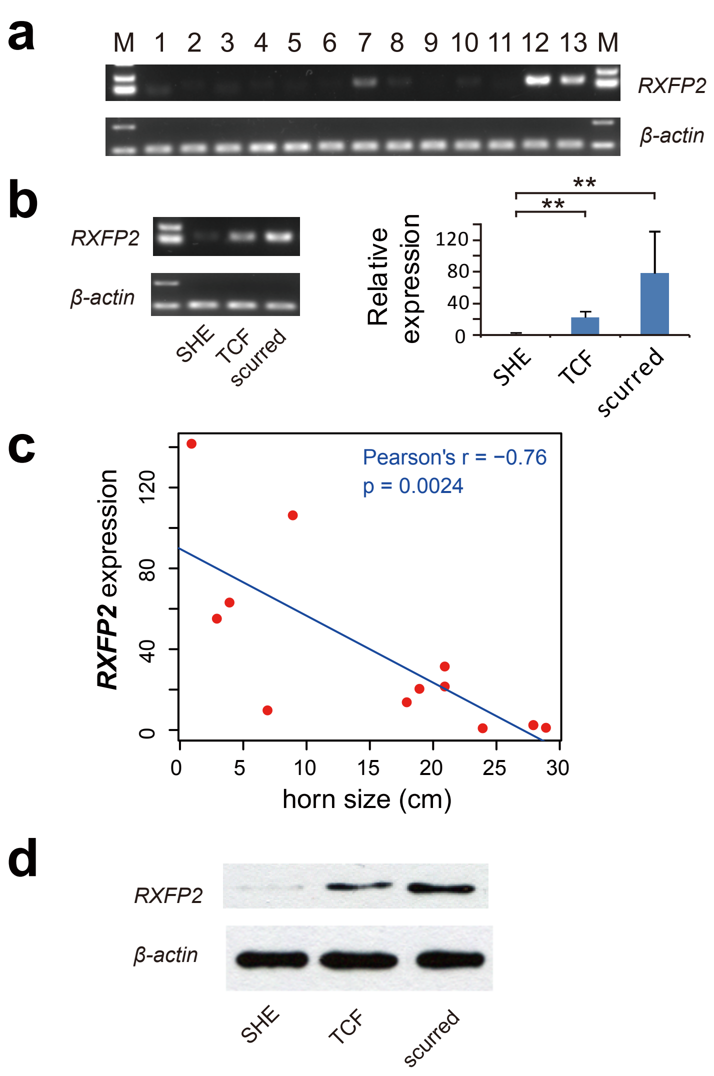
a
M 1 2 3 4 5 6 7 8 9 10 11 12 13 M
RXFP2
β-actin
b
RXFP2
β-actin
SHE
TCF
scurred
Relative expression
120
80
40
0
**
**
SHE
TCF
scurred
c
Pearson's r = −0.76
p = 0.0024
RXFP2 expression
0
40
80
120
0 5 10 15 20 25 30
horn size (cm)
d
RXFP2
β-actin
SHE
TCF
scurred

Supplementary Figures

Click here to access/download
**Supplementary Material**
Supplementary_Materials.revised2.doc

Click here to access/download
**Supplementary Material**
Supplementary Tables.revised2.xls

Cover letter to editor

Click here to download Personal Cover Cover_letter_to_editor.2nd revision.docx

Yixue Li, Ph.D

Key Laboratory of Computational Biology, CAS-MPG Partner Institute for Computational Biology,

Shanghai Institutes for Biological Sciences, Chinese Academy of Sciences,

320 Yue Yang Road, 200031 Shanghai, China

Email: yxli@sibs.ac.cn

Tel: +89-021-54920089

Dear Dr. Hans Zauner,

Thank you for your consideration of our manuscript entitled "**Whole genome sequences of 89 Chinese sheep suggest role of *RXFP2* in the development of unique horn phenotype as response to semi-feralization**" for its potential publication in *GigaScience*.

In the current version of our manuscript, we have made all the minor revisions suggested by the reviewer. We also tried to improve its further readability by using more concise language (e.g. L42, "similar to" -> "like"). References including URLs have been re-formatted as required by the journal style.

In addition, we have fixed some incorrect annotations in **Supplementary Tables S20-23** (e.g. Table S20, row 12 column E, "p.Glu641Gln" -> "p.Glu641Lys"), which were caused by errors during extracting protein-altering information.

All authors have approved this revised manuscript. We are looking forward to hearing from you again.

Best Regards.

Yours Sincerely,

Yixue Li & Mingxing Chu
