# Supplementary material for: Whole-genome sequences of 89 Chinese sheep suggest role of RXFP2 in the development of unique horn phenotype as response to semi-feralization
Source: Gigascience. 2018 Mar 7;7(4):giy019. doi: 10.1093/gigascience/giy019 (PMC5905515; doi:10.1093/gigascience/giy019)
Supplement: Response_to_Reviewer_Comments_Original_Submission.pdf [file giy019_response_to_reviewer_comments_original_submission.pdf]

Author's Response To Reviewer Comments

A general statement from the authors:
We thank all the reviewers for their great work in reviewing our manuscript entitled “Whole-genome sequences of 89 Chinese sheep suggest role of RXFP2 in the development of unique horn phenotype as response to semi-feralization”. Indeed, the advices and comments are provoke-thinking and helpful to improve the quality of our work. We have considered the questions and suggestions raised by the reviewers, and revised the manuscript accordingly. We list some major revisions here:
Additional analyses:
1. We performed selective sweep analysis, gene ontology analysis, RXFP2 haplotype distribution calculation after breaking down four populations PT, OL, VT and BY.
2. We did target sequencing of five random genomic regions in original samples (76 out of 99 sheep) to evaluate the accuracy of our WGS variant calling approach.
3. We compared the candidate gene list of present study with those from two previous approaches of Chinese native sheep.
4. We compared the RXFP2 haplotype in our dataset with that reported in wild bighorn sheep (based on their vcf genotype file, downloaded from internet).
5. We calculated breed-to-breed FST distance and generated a NJ tree.
Figures:
1. Figure 1b is replaced with a FST tree.
2. Original Figure 2 is broken down into two figures. Currently, Figure 2 is comprised of FST and HP manhattan plots from four divergent populations; Figure 3 shows the result of candidate gene analysis.
3. Figure details and legend texts are revised according to the suggestions by reviewers.
Main text:
1. The Introduction and Discussions were revised after we have consulted literatures recommended by the reviewers.
2. The Analyses section was revised based on new results (separately in four populations).
3. We marked up some of the revisions in the main text, those we think are essential for addressing the question asked by the reviewers.
4. Typos have been carefully checked and corrected. The manuscript was edited by a native-English speaker before uploading.

Point-to-point responses
Authors: Here are our point-to-point responses to the reviewers’ questions. Please note that for each reviewer we grouped the comments on typos and language editing as one point.
Reviewer 1
General comments by reviewer:
Reviewer: The authors have performed sequencing of 99 sheep from multiple populations in China (and Australia). They identify many regions with high Fst as well as reduced heterozygosity. Their top region contains the RXFP2 gene, which they use for functional analysis linking it to horn size (as has been reported earlier).

Major comments and responses:
1. Reviewer: Figure 1 nicely summarizes the breeds and their geographic location and their

genomic relationship. However, the authors quickly put the PT and OL population together labeling it TBS1. The VT population is relabeled TBP2. Since the TBS1 population separates according to the two breeds these populations should be treated separately. The paper needs to be redone throughout based on keeping the two (TBS1) populations separate.
Authors: It is a great suggestion to provide more information by treating each breed separately, even if we think PT and OL may share a similar trajectory of adaptation (as they are genetically proximate according to the PCA and admixture plot). In the revised manuscript, we reported selective sweep and candidate gene analysis in PT, OL, VT and BY, by comparing them separately with MGS (line 182-190, also see Figure 2, 3a-c). Overlaps between gene sets in different breeds were showed as venn plots (Figure 3a).

2. Reviewer: Figure 2 shows the Fst for TBS1 vs MGS and TBP2 vs MGS. It would be great to be able to compare the Fst plots of PT, OL, VT and BY separately compared to the MGS. This would allow the comparison of the signals that come up in different combinations of populations. I.e. PT & OL = big SHE horn; PT, OL, VT and BY = high altitude.
Authors: We agree that showing more Manhattan plots potentially gives more combinations of parameters, like horn size and altitudes. To address this, we analyzed the selective sweep and associated candidate genes in PT, OL, VT and BY compared with MGS (line 182-190). The result shows that BY has a smaller gene set (n=758) than PT, OL and VT (n=1125; 999; 1046), which makes sense because BY has relatively small genetic differences from other MGS lineages. Moreover, inclusion of the BY gene set revealed NF1 as a consistent signal across four populations (Figure 3a, c). However, more hypoxia-related genes (e.g. PTEN, PINK1) have undergone lineage-specific evolution (Figure 3c).
It is also worth noting that BY may not necessarily share their altitude adaptation genes with TBS. The polygenic basis of hypoxic response pathway means that different high-altitude lineages may have multiple ways to achieve the similar adaptations (a well-known example is the Tibetan chicken). In this case, although it would be interesting to know whether some “altitude genes” are shared by TBS and BY, considering consistency among high-altitude populations (PT, VT, OL, BY) may not be a golden standard to detect all “altitude genes”, since BY is a distant population and the adaptations are more likely to be lineage-specific. This concern is discussed in line 244-249.

3. Reviewer: While the data would be clearer if each of the four populations were shown, the authors may want to comment on the regions now found on chrom 13 and 15 - maybe they are related to altitude?
Authors: A consistently high FST in PT vs. MGS, OL vs. MGS and VT vs. MGS could be explained by a selective sweep either in TBS (PT + OL + VT), or MGS. A simple way to distinguish these two possibilities is to check whether TBS or MGS shows reduction of heterozygosity, or to calculate which haplotype is more different from the ancestral state (the lineage-specific branch length, LSBL). According to Figure 2, the window FST and HP signals on chromosome 13 and 15 suggest the latter possibility, which means they might contribute to the adaptive evolution of MGS.

4. Reviewer: The authors also need to compare the identified Fst regions with those found in previous sheet selection studies. It would also be interesting to compare the exact RXFP2 haplotypes 'associated' with horn here and in previous studies.

Authors: We followed this helpful suggestion and compared the candidate genes at all sweep regions we found (PT, OL, VT and BY vs. MGS) with two previous studies of Chinese sheep (Supplementary Table S12). Genes underlying altitude adaptations such as NF1 were confirmed by multiple studies. However, our data suggested a large number of genes underwent lineage-specific evolution, which is probably the reason why they are not identified when treating all Tibetan lineages as one group in other approaches.
We also compare the RXFP2 genotypic data in our 99 sheep with that from the wild bighorn sheep (Supplementary Figure S9, S10), whose WGS data are easily accessible from the Dryad (doi:10.5061/dryad.3f2t2). The result suggested that PT and OL were basically carrying a different haplotype from the bighorn sheep, although they were all subject to selective pressures at the same locus RXFP2 for developing strong horns.
We also think it would be informative if we can directly compare our genotypic data with more populations based on SNP chips. We consulted the most extensive collection of world-wide ovine genotypic data [1]. Nevertheless, we found that the SNP markers near RXFP2 are not polymorphic in our dataset, which means that these markers cannot be used to explain the haplotypic differences between SHE and TCF populations in our study.

5. Reviewer: How were SNPs/genotypes called given the low coverage sequencing data? Were all data from a breed analyzed together?
Authors: The variant calling step was performed by an early version of samtools (v1.2), which by default imputed missing genotypes based on other samples that were simultaneously processed. All data from one breed (n=10) were analyzed together, and then data from different breeds were merged into single variant file.
The advantage of the low-coverage design is to have more samples when given a total coverage depth for each breed (average depth per breed = average depth per sample × samples per breed), while the disadvantage is, apparently, the higher missing rate at each genetic variant. However, genotypic imputation (based on individuals from same breed) and window-based metrics compromised the problem, as the missing variants are supposed to be randomly distributed on the genome and among individuals.

6. Reviewer: Using multiple breeds for the analysis it might be possible to break down the selection signal near/over the RXFP2 signal (p 10 first paragraph, p13 1st paragraph - are haplotypes the same in the two semi-feral populations?)
Authors: It is a very helpful suggestion to break down the signal of selective sweep by each population. In the current version of Figure 4 and its related sections in the main text (line 254-275), we separately showed signals of each population (PT, OL, VT and BY), and the result was consistent with the previous one when PT and OL were combined. Basically, PT and OL have one dominant haplotype, and rest of the sheep breeds has another. A slight difference in haplotype frequency was observed between PT and OL as showed in Figure 4d. This is in rough agreement with the fact that SHE-type horns comprise >70% of PT population (in our 182 PT dataset), and is nearly fixed in OL population.

Minor comments and responses:
7. Reviewer: L36 "RXFP2 underlied rapid evolution" replace with "RXFP2 showed signs of rapid evolution"
-L43: "frequent contact with wild surroundings and rare human interventions".

-Keywords: add "sheep"
-L51: "the process where domestic animals"
-L54 "fit natural life while human artificial selection is no longer"
-L57 "trace back to 8 ka" replace with "trace back to 8,000 years ago "
-L58 "mito-genomic evolutionary study"
-L67-69: "as the Tibetan Plateau is rich in grasslands, the local breeds, especially ones living on prairies, have been roaming with nomads and fed on natural ranches"
-L71 "Third, unlike in other"
-L75 "loosened"
-L77-78 "breed from the Tibetan Plateau"
-L79 "sweeps"
-L84: "three"
- L118: "a rooted tree using the genome of the goat"
-L128 "performed principal component"
-L129 "Despite the division"
-L142-3 "statistics across the genome"
-L152 "from a relatively"
-L177 "observed the strongest signal"
-L178 "on chromosome 10"
-L181 "A previous study"
-L197 "in regions of positive selection"
-L199 "processes"
-L213 "intensely"
-L217 "a correlation"
-L231 "the TBS1 populations"
-L234-5 "the harsh environment on the Tibetan"
-L247 "variants in RXFP2"
-L297-8 "over the RXFP2 locus"
-L307 "compared the gene expression in"
-L308 "Despite obvious"
-L322 "study suggest that domestic animals might have re-acquired"
-L331 "to the Tibetan"
-L333 "selective sweeps"
-L340 "in competition to reproduce"
-L373 "evidence"
-L380 "across the RXFP2 gene"
-L483 "in RXFP2"
-L490 "genotyped in 182"
-L533 "levels of the five genes"
-p37: Figure 1 legend: spell out the names/shortenings of all sheep populations
-L746 "of the sheep pictures represent their lineage"
-L751 "clusters from K=2-4"
-L755-6 "regions under selection in TBS1"
-L768 "in red. For 627 TBS1 has the variant allele, whereas for 641 it has the reference allele"
-L779 Fig 4d: write how many individuals were included in analysis
-L789 linear regression is shown in red - it is not red - please correct

Authors: We appreciate these helpful suggestions to improve the quality of text. All these details were revised accordingly, and the manuscript was edited by a native speaker before uploading.

8. Reviewer: - L90 when you describe the two different semi-feral populations, you may want to indicate also which sheep are at high altitude as this might be an important parameter.
Authors: As suggested by the reviewer, we add a sentence at line 95-98 to indicate PT, VT, OL and BY as four high-altitude populations.

9. Reviewer: - L105 You describe that your dataset encompasses 94% of variants found in dbSNP. Can you say also how many novel SNPs you found?
Authors: >37.3% of variants in our dataset were novel compared with dbSNP build 143. This information is supplemented in the current version of manuscript at line 112-113.

10. Reviewer: - L190 MITF is being linked to hearing - it is worth noting that MITF is also frequently mutated in different coat color types so I don't think you know which is the case in this study.
Authors: We agree with the reviewer on this point. In the revised manuscript, we have now emphasized that some of the candidate genes like MITF might have multiple phenotypic outcomes (at line 208-211), and we also highlighted the double function of MITF in Figure 3b.

11. Reviewer: - A larger number of genes are described on p10 and it is not clear how they were selected and how they were assigned potential function.
Authors: We now use Figure 3b to summarize the selection signals of these typical genes, as well as the key words of their functional categories related with semi-feralization (also see Supplementary Table S19).

12. Reviewer: - p16 top paragraph: how were the five genes tested for expression chosen - what was rationale for 900 kb?
Authors: The number 900 was a typo in our original submission. The five genes we tested actually covered a ~1Mb region from chr10:28,984,259 bp to chr10: 30,002,883 bp.
The idea of this step is to include functional genes that are possibly affected by the selective sweep observed near RXFP2. The major concern is that the observed sweep signal might be hitchhiking with a causal variant located in the regulatory DNA elements of the flanking genes. From the LD plot showed in Supplementary Figure S13, it is obvious that the 1Mb region is comprised of multiple LD blocks, which means that the selected window size should be big enough to include all potential hitchhiking variants. Since we did not observed concordant expression level change in flanking genes, it is strong supporting evidence that RXFP2, instead of hitchhiking genes, is causal to the horn phenotypic changes. See revision at line 326-327.

13. Reviewer: -p18 first paragraph discussing expression pattern - would be useful to mention how many tissues you tried for expression before saying the RXFP2 gene is specifically expressed.
Authors: The sentence is now revised as “Thirdly, gene expression analysis in 13 tissues of PT sheep demonstrated …”. (line 364-365)

14. Reviewer: -L363+ How do you know whether sheep have been selected for a new mutation

versus picked up the ancestral state again? Can you find the key candidate mutations and see if they if they overlap between ancestral wild sheep and the re-feralized populations?
Authors: Our observations suggest PT and OL sheep were selected to evolve big and aggressive horns in a similar way like their wild relatives (because of sexual competition), but indeed the data is not sufficient to clarify whether they have derived a new genotype or picked up an old one. We showed two candidate protein mutations 627 and 641 with high FST and strong correlation with horn phenotypes (Figure 4c). Nevertheless, their phylogenetic origins are contradictory (where at 641 PT and OL have the ancestral allele, at 627 they have the mutated allele). We also compared the RXFP2 haplotype of our populations with the wild bighorn sheep (Supplementary Figure S9-S10), but failed to see a correlation between the wild population and SHE horned PT and OL. To further clarify the origin of this phenotype, it would be interesting to test whether the RXFP2 haplotype conferring SHE horns might have come from other sheep population with similar horn shapes (Racka), as well as from other wild populations regarded as the ancestors of Chinese sheep, but this is not currently achievable because there is no available data.

15. Reviewer: -p21 first paragraph - how much false positive and false negative variants do you expect?
Authors: To address this question, we performed targeted sequencing of five random genomic regions (including 33 SNPs) over 72 out of the 99 original samples. The results showed in Supplementary Table S26 were utilized to calculate false positive and false negative rates of the SNP calling step. The false positive rate (FPR) defined as the proportion of wrongly defined mutated alleles is 3.34% (FPR = 34/1017) in tested samples, while the false negative rate (FNR) defined as the proportion of wrongly defined reference alleles is 1.47% (FNR = 53/3611).
Sanger (Validation)
Ref Alt Total
WGS variant calling Ref 3558 34 3630
Alt 53 945 998
Total 3611 1017
Ref, Reference allele; Alt, Alternative allele;
According to the ensuing validation, heterozygotes has a much higher false calling rate than homozygotes: 63 out of 411 heterozygotes and 19 out of 1,903 homozygotes were not correctly genotyped in variant calling step (error rate: heterozygotes = 15.3%; homozygotes = 1.0%). This error type distribution does not exceed our expectation, because calling genotypes of heterozygotes in diploid organisms often requires a high coverage depth. The average read depth for each individual is about 6X in our study (one allele is overlapped with ~3 reads), which is quite satisfactory for detecting population-wide allele frequencies, but with restricted power to annotate allelic heterogeneity.

16. Reviewer: -L449 - Is it possible that the 46 windows with few variants may be selected similarly in both populations?
Authors: We list in the current Supplementary Table S28 the sweep statistics for the 46 removed windows. The rationale for removing these windows is because the sweep signals are less convincing when there are few numbers of variants. Indeed, there are significant FST and HP also in these few variant regions, but it is difficult to test whether that is caused by a few random fixations without sufficient observations of genetic hitchhiking. On a different note, with a

relatively small window size and step size (30kb, 15kb), a selective sweep signal is often broken down into multiple windows (e.g. the signal on chromosome 20), which enables us to detect most of the sweeps even if we exclude 46 few variant regions.

17. Reviewer: -23 last paragraph: it is quite possible some genes, such as MITF might have multiple mutations/multiple sweep signals across the gene for different coat color patterns. I would therefore not use the majority rules but actually report all signals.
Authors: We agree that the majority rule is not applicable if one gene has multiple sweep events. We now have defined candidate genes from each population separately (based on FST and HP), and then calculated their overlaps like showed in Figure 3a.

18. Reviewer: -L765 add the window size used around the gene
Authors: The visualized window is from chromosome 10: 29,400,000-29,550,000 bp. This information is added to the legend text (line 804-805).

19. Reviewer: Figure 1: a. Make lines from map to breed not have extra lines
b/c. replace tBS labels with individual breed labels
Authors: The figure is revised according to the suggestion.

Figure 2: adjust to show all four divergent populations in parallel
Authors: The current version of Figure 2 contains parallel comparison of FST and HP from four divergent populations.
Reviewer 2
General comments by reviewer:
Reviewer: This is a useful study on whole-genome sequences from Chinese sheep, which yields evidence that a mutation in the RXPF2 gene is causative for a horn phenotype as adaptation to semi-feralization. The analysis is according to the state-of-the-art. It is likely that the dataset harbors many more mutations in several of the hundreds of genes implicated in environmental adaptation, so there are clear opportunities for follow-up studies. The present results are interesting and deserve publication after a major revision.

Major comments and responses:
1. Reviewer: We understand that a grandiloquent title attracts attention. However, this title does not mention that the study is focused on Chinese sheep and does not refer to RXPF2, the major target of this study, the subject in three of the five figures and also dominating the Discussion. A possible alternative:
Whole-genome sequences of 89 Chinese sheep breeds suggest a role of RXFP2 in the development of a unique horn phenotype as response to semi-feralization.
Note that such a more informative title transmits the same message as the present one (and does it even better).
Authors: As suggested by the reviewer, we have now revised the title as “Whole-genome sequences of 89 Chinese sheep suggest role of RXFP2 in the development of unique horn phenotype as response to semi-feralization”.

2. Reviewer: Giga amounts of data require time-consuming analyses, which can only deliver a small part of the potential output. However, this should not be at the expense of an essential part

of any scientific report: a comparison with previous literature, mentioning result that are not entirely novel but confirm previous findings. Ref. 14 reports WGS of 80 sheep from 3 climate zones in China. Because in this study Tibetan sheep were treated as one group, RXFP2 as gene subject to selection has been missed. However, Yang et al. [14] also target the high-altitude adaptation of Tibetan sheep, highlighting the role of SOCS2. A complete meta-analysis would be most fruitful, but is outside the scope of the current submission. Nevertheless, the Introduction and Discussion should pay more attention to the previous study [14] and at least touch the following points:
(1) The introduction should refer to the demographic history of the main groups of Chinese breeds [14; Zhao et al. (2017), Genomic reconstruction of the history of Chinese native sheep: insights into peopling role of nomadic nationalities societies and expansions of early pastoralism. Mol. Biol. Evol., in press and accessible via Internet].
Authors: We appreciate this helpful suggestion and agree that the Introduction and Discussion would be more informative after revision on these points. We have consulted the papers suggested by the reviewer, and revised the second paragraph of the Introduction. In its current form, we briefly describe the demographic history and geographic distributions of Chinese sheep based on literatures. This is in order to give a general picture of domestic sheep origin and their spread in China, as well as the sequential order of the split of major ovine groups from their ancestral lineage.

Reviewer: (2) How are the PT, OL and VT breeds related to the Nagqu (ZNQ), Qamdo (ZCD), Shigatse (ZRK), Nyingchi (ZLZ), also from Tibet [14]? Lines 147-150 mention only briefly the proximity of VT and ZLZ. I recommend a Supplementary map giving the locations of the populations studied in [14] and in the present report.
Authors: As suggested by the reviewer, we have provided a map (Supplementary Figure S5) to show the geographic distribution of different Tibetan lineages in our study and in the previous study of native sheep. The introduction of this geographic distribution patterns is described in a separate paragraph at line 152-159.

Reviewer: (3) If phenotypic data are available for the other Tibetan breeds [14]: do they also have twisted SHE horns?
(4) If so, and assuming the WGS data from [14] are accessible: do they also have the same RXPF2 mutation? This would lend strong support to the message of this study!
Authors: We agree that a direct comparison between our genotypic data and that from the suggested reference paper will be valuable. However, the raw reads or genotypic data from the suggested reference paper has not been released to any public resource. We also tried to contact the authors, but failed to get the access.

(5) Do both studies share other genes as being implicated in adaptation to the high-altitude and hypoxia? I saw that at least part of the genes listed in the Supplementary Table 10 are also mentioned in [14] as being selected in Tibetan sheep. It is relevant to indicate these shared genes in Table S10, if only to indicate that these results are not novel.
Authors: In the current Supplementary Table S12, we have listed the candidate genes in our four populations PT, OL, VT and BY, and their overlap with two previous gene list from references [2] and [3].

3. Reviewer: At the beginning of the Discussion, a clear survey of the most essential features of diversity pattern would support the take-home message: a separate position of Tibetan sheep; within these sheep a contrast of domestic and semi-feral breeds, the former even less diverse than the latter; development of a unique semi-feral horn morphology as plausible adaptation to semi-feralization.
Authors: We followed the suggestion and revised the first paragraph of the Discussion accordingly (line 346-353).

4. Reviewer: Fig. 1b: a tree of NeighborNet graph of FST genetic distance between the breeds will be more informative and better support the message of this paper.
Authors: Indeed, the FST tree can better represent the structure across breeds. From the current Figure 1b, a clear relationship between 10 breeds is showed. Nevertheless, we also preserved the previous phylogeny tree in Supplementary Figure S2 because it provided additional information of the relationship between individual samples, and the position of root (goat).

5. Reviewer: The manuscript needs to be read by a native-English speaker, preferable a scientist, in order to weed out the several awkward phrasings. A few are mentioned below.
Line 49: "in order to understand better (etc.)".
Line 52: you probably mean that protection offered by the domestic habitat suppresses the original environmental adaptation.
Line 108: rephrase in order to indicate more clearly that the nucleotide diversity in Tibetan breeds is higher than in other Chinese breeds.
Lines 265-266, rephrase: "The SHE horns are clearly different from the horns of (etc.)".
Line 331: "the Tibetan Plateau". Lines 331-333: awkward and superfluous sentence. Line 333: selective sweeps [plural].
Line 361: "naturalistic" refers to an artistic style; probably you mean a natural wildlife habitat.
Line 543: Goa -> goat
Authors: We have carefully checked and revised these sentences mentioned by the reviewer. The revised manuscript was edited by a native speaker before uploading.

Minor comments and responses:
6. Reviewer: Fig. 1: please define in the legends the abbreviations for the breed categories (EUS, MGS, TBS1, TBS2).
Authors: We revised the legend text of Figure 1. Its current form contains definition of all abbreviations for breeds and lineages.

7. Reviewer: It is a good idea to use colors consistently across figures. However, in Fig. 1a the MGS sheep should be shown at a dark blue background and the TBS sheep at a light blue background instead of vice versa in order to harmonize with Figs. 1d and S3.
Authors: We realize that some color inconsistency in our figures is misleading to the readers. To address the problem, we have adjusted the color in Figure 1 and Figure S3. Also, we paid more attention to color consistency in other figures, such like that between Figure 2 and 3c.

8. Reviewer: Data have been submitted to the SRA. In addition, it would be most useful to submit the novel SNPs to the Ensemble Variation Archive.
Authors: We assume you mean the European Variation Archive (EVA), which collects variation

data from non-human organisms.
The vcf file was submitted to EVA before uploading this revised manuscript. All data has been released to the public. See data access information at line 557-560

9. Reviewer: Line 62: also refer to [14] and Zhao et al. (2017), who on the basis of genome-wide SNPs differentiate three breed clusters.
Authors: We revised the introduction of Chinese sheep lineages based on the suggested literature (line 57-67).

10. Reviewer: Lines 147-149: just mention the close proximity of ZLZ and VT and the comparably low LD. See point 2 about a more complete comparison of these breeds and other Tibetan breeds [14], which should precede this paragraph.
Authors: The sentence is revised as "Moreover, population ZLZ in the other study was proximate to VT and also exhibited a sign of population bottleneck (evidenced by slow LD decay)." (line 166-168)
Also, the geographic distribution of our 3 populations and the 4 populations in [2] is now discussed in the preceding paragraph at line 152-159.

11. Reviewer: Lines 150-153: just mention that the LD indicates a population bottleneck.
Authors: The sentence is revised as "and also exhibited a sign of population bottleneck (evidenced by slow LD decay)" (line 166-168).

12. Reviewer: Lines 154-156: this was already convincingly clear on the basis of Fig. 1.
Authors: We deleted this paragraph.

13. Reviewer: Lines 188-190 repeat the preceding paragraph; this should be integrated.
Authors: We revised this sentence, so it now describes signals in addition to RXFP2 (line 200).

14. Reviewer: In this context, it is should be mentioned that the well-known Hungarian Racka sheep also has SHE horns (haven't they?).
Authors: It is an intriguing similarity between SHE-horned Tibetan sheep and Hungarian Racka sheep, which we hadn't noticed before. An important question behind this is whether the SHE-horn genotype is newly derived in semi-feral TBS, or is an introgression from other sheep populations. We are not sure which is the case, since we don't have the genotypic data from other possible "donors" of RXFP2 haplotypes, including Racka. From our data, what is certain, however, is that this haplotype of RXFP2 confers SHE horns, and is nearly driven to fixation in semi-feral TBS under positive selection. See our discussion at line 375-381.

15. Reviewer: Lines 268, 362: of course, the horns are used during fighting with competitors and predators, but it is a bit curious to state that SHE sheep and the wild ancestors look strong and aggressive; better omit these statements.
Authors: As suggested, we deleted these sentences.

16. Reviewer: Figs. 4b and 4d can easily be combined, while the legends should mention more clearly that (as I understand) they show correlations with horn length and horn shape, respectively.

Authors: These two figures are now combined into Figure 5b, where different line types were used to indicate the measurement outcome (either horn size or shape).

17. Reviewer: Lines 317-323: this paragraph can be omitted since the same points will be made in the Discussion (where it belongs anyway).
Authors: As suggested, we removed this paragraph.

References

1. Kijas JW, Lenstra JA, Hayes B, Boitard S, Neto LRP, San Cristobal M, et al. Genome-Wide Analysis of the World's Sheep Breeds Reveals High Levels of Historic Mixture and Strong Recent Selection. Plos Biology. 2012;10 2.
2. Yang J, Li WR, Lv FH, He SG, Tian SL, Peng WF, et al. Whole-genome sequencing of native sheep provides insights into rapid adaptations to extreme environments. Molecular Biology and Evolution. 2016;33:2576-92. doi:10.1093/molbev/msw129.
3. Wei C, Wang H, Liu G, Zhao F, Kijas JW, Ma Y, et al. Genome-wide analysis reveals adaptation to high altitudes in Tibetan sheep. Scientific reports. 2016;6:26770. doi:10.1038/srep26770.
