# Supplementary material for: Whole-genome sequences of 89 Chinese sheep suggest role of RXFP2 in the development of unique horn phenotype as response to semi-feralization
Source: Gigascience. 2018 Mar 7;7(4):giy019. doi: 10.1093/gigascience/giy019 (PMC5905515; doi:10.1093/gigascience/giy019)
Supplement: Response_to_Reviewer_Comments_Revision_1.pdf [file giy019_response_to_reviewer_comments_revision_1.pdf]

Author's Response To Reviewer Comments

We would like to thank again the editor and reviewers for their critical suggestions on our manuscript during the entire submission process. In this 2nd revision, we have made language corrections including all those suggested by the reviewer. Moreover, we fixed some incorrect annotations in the Supplementary Table S20-23, which were caused by errors during extracting protein-altering information. Details of these corrections are listed below:

Language corrections:
1. Reviewer: L65: denoted as Mongolian sheep because of their distinctive fat-storage phenotypes (fat tails or rumps) (etc).
L68: Omit first 4 words.
L113: were absent from the public collection-> are novel
L159: is not the only determinant of (etc.)
L164: omit "during localization"
L166: Moreover, population ZLA is proximate to VT and also exhihits slow LD decay as sign of a population bottleneck.
L168-170: Omit this sentence. (has already been said)
L209: MITF variants that contribute
L349: critical role of semi-feralization in development of male reproductive success, eating habits (etc.). (present formulation a bit awkward)

Authors: Corrections done.

2. Reviewer: L237: corroborate (present tense for findings that still are true)

Authors: We now use “provide new evidences to the hypoxic adaptation of TBS”, which we agree is more precise.

Update to Supplementary Table S20-23:
Authors: We updated the columns related with protein-altering annotations because we found the previous one is incorrect at some specific rows (for example, in Table S20, row 12 column E, “p.Glu641Gln” should be “p.Glu641Lys”). This is caused by an error in our previous pipeline for extracting these annotation information, which has been fixed now.
