# Supplementary material for: Whole-genome sequences of 89 Chinese sheep suggest role of RXFP2 in the development of unique horn phenotype as response to semi-feralization
Source: Gigascience. 2018 Mar 7;7(4):giy019. doi: 10.1093/gigascience/giy019 (PMC5905515; doi:10.1093/gigascience/giy019)
Supplement: Reviewer_1_Report_(Original_Submission) -- Kerstin Lindblad-Toh — 18 Aug 2017 Reviewed [file giy019_reviewer_1_report_(original_submission).pdf]

Reviewer Report

Title: Whole-genome sequences of 89 Chinese sheep suggest role of RXFP2 in the development of unique horn phenotype as response to semi-feralization

Version: Original Submission **Date:** 18 Aug 2017

Reviewer name: Kerstin Lindblad-Toh

Reviewer Comments to Author:

Review GIGA-17-00165

The authors have performed sequencing of 99 sheep from multiple populations in China (and Australia). They identify many regions with high Fst as well as reduced heterozygosity. Their top region contains the RXFP2 gene, which they use for functional analysis linking it to horn size (as has been reported earlier).

Major comments:
- Figure 1 nicely summarizes the breeds and their geographic location and their genomic relationship. However, the authors quickly put the PT and OL population together labeling it TBS1. The VT population is relabeled TBP2. Since the TBS1 population separates according to the two breeds these populations should be treated separately. The paper needs to be redone throughout based on keeping the two (TBS1) populations separate.

- Figure 2 shows the Fst for TBS1 vs MGS and TBP2 vs MGS. It would be great to be able to compare the Fst plots of PT, OL, VT and BY separately compared to the MGS. This would allow the comparison of the signals that come up in different combinations of populations. I.e. PT & OL = big SHE horn; PT, OL,VT and BY = high altitude.

- While the data would be clearer if each of the four populations were shown, the authors may want to comment on the regions now found on chrom 13 and 15 - maybe they are related to altitude?

- The authors also need to compare the identified Fst regions with those found in previous sheet selection studies. It would also be interesting to compare the exact RXFP2 haplotypes 'associated' with horn here and in previous studies.

- How were SNPs/genotypes called given the low coverage sequencing data? Were all data from a breed analyzed together?

- Using multiple breeds for the analysis it might be possible to break down the selection signal near/over the RXFP2 signal (p 10 first paragraph, p13 1st paragraph - are haplotypes the same in the two semi-feral populations?)

Smaller comments:
- l 36 "RXFP2 underlied rapid evolution" replace with "RXFP2 showed signs of rapid evolution"
- l 43: "frequent contact with wild surroundings and rare human interventions".
- Keywords: add "sheep"
- L 51: "the process where domestic animals"
- L54 "fit natural life while human artificial selection is no longer"
- L57 "trace back to 8 ka" replace with "trace back to 8,000 years ago "
- L58 "mito-genomic evolutionary study"
- L67-69: "as the Tibetan Plateau is rich in grasslands, the local breeds, especially ones living on prairies, have been roaming with nomads and fed on natural ranches"
- L71 "Third, unlike in other"
- L75 "loosened"
- L77-78 "breed from the Tibetan Plateau"
- L79 "sweeps"
- L84: "three"
- L90 when you describe the two different semi-feral populations, you may want to indicate also which sheep are at high altitude as this might be an important parameter.
- L105 You describe that your dataset encompasses 94% of variants found in dbSNP. Can you say also how many novel SNPs you found?
- L118: "a rooted tree using the genome of the goat"
-L128 "performed principal component"
-L129 "Despite the division"
-L142-3 "statistics across the genome"
-L152 "from a relatively"
-L177 "observed the strongest signal"
-L178 "on chromosome 10"
-L181 "A previous study"
- L190 MITF is being linked to hearing - it is worth noting that MITF is also frequently mutated in different coat color types so I don't think you know which is the case in this study.
- A larger number of genes are describe on p10 and it is not clear how they were selected and how they were assigned potential function.
-L197 "in regions of positive selection"
-L199 "processes"
-L213 "intensely"
-L217 "a correlation"
-L231 "the TBS1 populations"
-L234-5 "the harsh environment on the Tibetan"
-L247 "variants in RXFP2"
-L297-8 "over the RXFP2 locus"
- p 16 top paragraph: how were the five genes tested for expression chosen - what was rationale for 900 kb?
-L307 "compared the gene expression in"

-L308 "Despite obvious"
-L322 "study suggest that domestic animals might have re-acquired"
-L331 "to the Tibetan"
-L333 "selective sweeps"
-L340 " in competition to reproduce"
-p 18 first paragraph discussing expression pattern - would be useful to mention how many tissues you tried for expression before saying the RXFP2 gene is specifically expressed.
-L363+ How do you know whether sheep have been selected for a new mutation versus picked up the ancestral state again? Can you find the key candidate mutations and see if they if they overlap between ancestral wild sheep and the re-feralized populations?
-L373 "evidence"
-L380 "across the RXFP2 gene"
-p21 first paragraph - how much false positive and false negative variants do you expect?
-L449 - Is it possible that the 46 windows with few variants may be selected similarly in both populations?
-23 last paragraph: it is quite possible some genes, such as MITF might have multiple mutations/multiple sweep signals across the gene for different coat color patterns. I would therefore not use the majority rules but actually report all signals.
-L483 "in RXFP2"
-L490 "genotyped in 182"
-L533 "levels of the five genes"
-p37: Figure 1 legend: spell out the names/shortenings of all sheep populations
-L746 "of the sheep pictures represent their lineage"
-L751 "clusters from K=2-4"
-L755-6 "regions under selection in TBS1"
-L765 add the window size used around the gene
-L768 "in red. For 627 TBS1 has the variant allele, whereas for 641 it has the reference allele"
-L779 Fig4d : write how many individuals were included in analysis
-L789 linear regression is shown in red - it is not red - please correct

Figure 1: a. Make lines from map to breed not have extra lines
b/c. replace tBS labels with individual breed labels
Figure 2 : adjust to show all four divergent populations in parellel

Methods

Are the methods appropriate to the aims of the study, are they well described, and are necessary controls included? No

Conclusions

Are the conclusions adequately supported by the data shown? No

Reporting Standards

Does the manuscript adhere to the journal's guidelines on [minimum standards of reporting?]() Yes

Statistics

Are you able to assess all statistics in the manuscript, including the appropriateness of statistical tests used? Yes, and I have assessed the statistics in my report.

Quality of Written English

Please indicate the quality of language in the manuscript: Needs some language corrections before being published

Declaration of Competing Interests

Please complete a declaration of competing interests, considering the following questions:

- Have you in the past five years received reimbursements, fees, funding, or salary from an organisation that may in any way gain or lose financially from the publication of this manuscript, either now or in the future?
- Do you hold any stocks or shares in an organisation that may in any way gain or lose financially from the publication of this manuscript, either now or in the future?
- Do you hold or are you currently applying for any patents relating to the content of the manuscript?
- Have you received reimbursements, fees, funding, or salary from an organization that holds or has applied for patents relating to the content of the manuscript?
- Do you have any other financial competing interests?
- Do you have any non-financial competing interests in relation to this paper?

If you can answer no to all of the above, write 'I declare that I have no competing interests' below. If your reply is yes to any, please give details below.

I declare that I have no competing interests

I agree to the open peer review policy of the journal. I understand that my name will be included on my report to the authors and, if the manuscript is accepted for publication, my named report including any attachments I upload will be posted on the website along with the authors' responses. I agree for my report to be made available under an Open Access Creative Commons CC-BY license (http://creativecommons.org/licenses/by/4.0/). I understand that any comments which I do not wish to

be included in my named report can be included as confidential comments to the editors, which will not be published.

I agree to the open peer review policy of the journal

To further support our reviewers, we have joined with Publons, where you can gain additional credit to further highlight your hard work (see: https://publons.com/journal/530/gigascience). On publication of this paper, your review will be automatically added to Publons, you can then choose whether or not to claim your Publons credit. I understand this statement.

Yes
