# Supplementary material for: Whole-genome sequences of 89 Chinese sheep suggest role of RXFP2 in the development of unique horn phenotype as response to semi-feralization
Source: Gigascience. 2018 Mar 7;7(4):giy019. doi: 10.1093/gigascience/giy019 (PMC5905515; doi:10.1093/gigascience/giy019)
Supplement: Reviewer_1_Report_(Original_Submission)_ReviewGIGA-17-00165.pdf [file giy019_reviewer_1_report_(original_submission)_reviewgiga-17-00165.pdf]

Review GIGA-17-00165

The authors have performed sequencing of 99 sheep from multiple populations in China (and Australia). They identify many regions with high Fst as well as reduced heterozygosity. Their top region contains the *RXFP2* gene, which they use for functional analysis linking it to horn size (as has been reported earlier).

Major comments:

- Figure 1 nicely summarizes the breeds and their geographic location and their genomic relationship. However, the authors quickly put the PT and OL population together labeling it TBS1. The VT population is relabeled TBP2. Since the TBS1 population separates according to the two breeds these populations should be treated separately. The paper needs to be redone throughout based on keeping the two (TBS1) populations separate.

- Figure 2 shows the Fst for TBS1 vs MGS and TBP2 vs MGS. It would be great to be able to compare the Fst plots of PT, OL, VT and BY separately compared to the MGS. This would allow the comparison of the signals that come up in different combinations of populations. I.e. PT & OL = big SHE horn; PT, OL,VT and BY = high altitude.

- While the data would be clearer if each of the four populations were shown, the authors may want to comment on the regions now found on chrom 13 and 15 - maybe they are related to altitude?

- The authors also need to compare the identified Fst regions with those found in previous sheet selection studies. It would also be interesting to compare the exact *RXFP2* haplotypes 'associated' with horn here and in previous studies.

- How were SNPs/genotypes called given the low coverage sequencing data? Were all data from a breed analyzed together?

- Using multiple breeds for the analysis it might be possible to break down the selection signal near/over the *RXFP2* signal (p 10 first paragraph, p13 1st paragraph – are haplotypes the same in the two semi-feral populations?)

Smaller comments:

- l 36 "RXFP2 underlied rapid evolution" replace with "*RXFP2* showed signs of rapid evolution"
- l 43: "frequent contact with wild **surroundings** and rare human interventions".
- Keywords: add "sheep"
- L 51: "the process **where** domestic animals"
- L54 "fit natural life **while human** artificial selection **is** no longer"
- L57 "trace back to 8 ka" replace with "trace back to **8,000 years ago** "

- L58 “mito-genomic evolution**ary** study”
- L67-69: “as **the** Tibetan Plateau is rich **in** grassland**s**, the local breeds, especially ones living **on** prairies, **have been** roaming with nomads and fed on natural ranch**es**”
- L71 “Third, unlike **in** other”
- L75 “loosen**ed**”
- L77-78 “breed **from the** Tibet**an** Plateau”
- L79 “sweep**s**”
- L84: “**three**”

- L90 when you describe the two different semi-feral populations, you may want to indicate also which sheep are at high altitude as this might be an important parameter.
- L105 You describe that your dataset encompasses 94% of variants found in dbSNP. Can you say also how many novel SNPs you found?
- L118: “a rooted tree **using** the genome of **the** goat”
-L128 “performed principal component”
-L129 “**Despite** the division”
-L142-3 “statistics **across** the genome”
-L152 “from **a** relatively”
-L177 “observed **the strongest** signal”
-L178 “**on** chromosome 10”
-L181 “**A** previous study”
- L190 MITF is being linked to hearing – it is worth noting that MITF is also frequently mutated in different coat color types so I don’t think you know which is the case in this study.
- A larger number of genes are describe on p10 and it is not clear how they were selected and how they were assigned potential function.
-L197 “**in regions of** positive selection”
-L199 “process**es**”
-L213 “**intensely**”
-L217 “**a** correlation”
-L231 “**the** TBS1 population**s**”
-L234-5 “the **harsh** environment **on the** Tibetan”
-L247 “variants **in** *RXFP2*”
-L297-8 “over **the** *RXFP2* locus”
- p 16 top paragraph: how were the five genes tested for expression chosen – what was rationale for 900 kb?
-L307 “compared **the** gene expressi**on** in”
-L308 “**Despite** obvious”
-L322 “study **suggest** that domestic animals might **have** re-acquired”
-L331 “to **the** Tibetan”
-L333 “selective sweeps”
-L340 “ in competition **to reproduce**”
-p 18 first paragraph discussing expression pattern – would be useful to mention how many tissues you tried for expression before saying the RXFP2 gene is specifically expressed.

-L363+ How do you know whether sheep have been selected for a new mutation versus picked up the ancestral state again? Can you find the key candidate mutations and see if they if they overlap between ancestral wild sheep and the re-feralized populations?
-L373 "evidence"
-L380 "**across the** *RXFP2* gene"
-p21 first paragraph – how much false positive and false negative variants do you expect?
-L449 – Is it possible that the 46 windows with few variants may be selected similarly in both populations?
-23 last paragraph: it is quite possible some genes, such as MITF might have multiple mutations/multiple sweep signals across the gene for different coat color patterns. I would therefore not use the majority rules but actually report all signals.
-L483 "**in** *RXFP2*"
-L490 "genotype**d in** 182"
-L533 "levels of **the five** genes"
-p37: Figure 1 legend: spell out the names/shortenings of all sheep populations
-L746 "**of the sheep pictures represent their lineage**"
-L751 "clusters from **K=**2-4"
-L755-6 "**regions under selection** in TBS1"
-L765 add the window size used around the gene
-L768 "in red. **For 627 TBS1 has the variant allele, whereas for 641 it has the reference allele**"
-L779 Fig4d : write how many individuals were included in analysis
-L789 linear regression is shown in red – it is not red – please correct

Figure 1: a. Make lines from map to breed not have extra lines
b/c. replace tBS labels with individual breed labels
Figure 2 : adjust to show all four divergent populations in parellel
