# Supplementary material for: Whole-genome sequences of 89 Chinese sheep suggest role of RXFP2 in the development of unique horn phenotype as response to semi-feralization
Source: Gigascience. 2018 Mar 7;7(4):giy019. doi: 10.1093/gigascience/giy019 (PMC5905515; doi:10.1093/gigascience/giy019)
Supplement: Reviewer_2_Report_(Original_Submission) -- Johannes Lenstra — 22 Aug 2017 Reviewed [file giy019_reviewer_2_report_(original_submission).pdf]

Reviewer Report

Title: Whole-genome sequences of 89 Chinese sheep suggest role of RXFP2 in the development of unique horn phenotype as response to semi-feralization

Version: Original Submission **Date:** 22 Aug 2017

Reviewer name: Johannes Lenstra

Reviewer Comments to Author:

This is a useful study on whole-genome sequences from Chinese sheep, which yields evidence that a mutation in the RXPF2 gene is causative for a horn phenotype as adaptation to semi-feralization. The analysis is according to the state-of-the-art. It is likely that the dataset harbors many more mutations in several of the hundreds of genes implicated in environmental adaptation, so there are clear opportunities for follow-up studies. The present results are interesting and deserve publication after a major revision.

Major comments

1. We understand that a grandiloquent title attracts attention. However, this title does not mention that the study is focused on Chinese sheep and does not refer to RXPF2, the major target of this study, the subject in three of the five figures and also dominating the Discussion. A possible alternative: Whole-genome sequences of 89 Chinese sheep breeds suggest a role of RXFP2 in the development of a unique horn phenotype as response to semi-feralization.
Note that such a more informative title transmits the same message as the present one (and does it even better).

2. Giga amounts of data require time-consuming analyses, which can only deliver a small part of the potential output. However, this should not be at the expense of an essential part of any scientific report: a comparison with previous literature, mentioning result that are not entirely novel but confirm previous findings. Ref. 14 reports WGS of 80 sheep from 3 climate zones in China. Because in this study Tibetan sheep were treated as one group, RXFP2 as gene subject to selection has been missed. However, Yang et al. [14] also target the high-altitude adaptation of Tibetan sheep, highlighting the role of SOCS2. A complete meta-analysis would be most fruitful, but is outside the scope of the current submission. Nevertheless, the Introduction and Discussion should pay more attention to the previous study [14] and at least touch the following points:

(1) The introduction should refer to the demographic history of the main groups of Chinese breeds [14; Zhao et al. (2017), Genomic reconstruction of the history of Chinese native sheep: insights into peopling role of nomadic nationalities societies and expansions of early pastoralism. Mol. Biol. Evol., in press and accessible via Internet].

(2) How are the PT, OL and VT breeds related to the Nagqu (ZNQ), Qamdo (ZCD), Shigatse (ZRK), Nyingchi (ZLZ), also from Tibet [14]? Lines 147-150 mention only briefly the proximity of VT and ZLZ. I recommend a Supplementary map giving the locations of the populations studied in [14] and in the present report.

(3) If phenotypic data are available for the other Tibetan breeds [14]: do they also have twisted SHE horns?

(4) If so, and assuming the WGS data from [14] are accessible: do they also have the same RXPF2 mutation? This would lend strong support to the message of this study!

(5) Do both studies share other genes as being implicated in adaptation to the high-altitude and hypoxia? I saw that at least part of the genes listed in the Supplementary Table 10 are also mentioned in [14] as being selected in Tibetan sheep. It is relevant to indicate these shared genes in Table S10, if only to indicate that these results are not novel.

3. At the beginning of the Discussion, a clear survey of the most essential features of diversity pattern would support the take-home message: a separate position of Tibetan sheep; within these sheep a contrast of domestic and semi-feral breeds, the former even less diverse than the latter; development of a unique semi-feral horn morphology as plausible adaptation to semi-feralization.

4. Fig. 1b: a tree of NeighborNet graph of FST genetic distance between the breeds will be more informative and better support the message of this paper.

5. The manuscript needs to be read by a native-English speaker, preferable a scientist, in order to weed out the several awkward phrasings. A few are mentioned below.

Minor comments

6. Fig. 1: please define in the legends the abbreviations for the breed categories (EUS, MGS, TBS1, TBS2).

7. It is a good idea to use colors consistently across figures. However, in Fig. 1a the MGS sheep should be shown at a dark blue background and the TBS sheep at a light blue background instead of vice versa in order to harmonize with Figs. 1d and S3.

8. Data have been submitted to the SRA. In addition, it would be most useful to submit the novel SNPs to the Ensemble Variation Archive.

9. Line 49: "in order to understand better (etc.)".

10. Line 52: you probably mean that protection offered by the domestic habitat suppresses the original environmental adaptation.

11. Line 62: also refer to [14] and Zhao et al. (2017), who on the basis of genome-wide SNPs differentiate three breed clusters.

12. Line 108: rephrase in order to indicate more clearly that the nucleotide diversity in Tibetan breeds is higher than in other Chinese breeds.

13. Lines 147-149: just mention the close proximity of ZLZ and VT and the comparably low LD. See point 2 about a more complete comparison of these breeds and other Tibetan breeds [14], which should precede this paragraph.

14. Lines 150-153: just mention that the LD indicates a population bottleneck.

15. Lines 154-156: this was already convincingly clear on the basis of Fig. 1.

16. Lines 188-190 repeat the preceding paragraph; this should be integrated.

17. Lines 265-266, rephrase: "The SHE horns are clearly different from the horns of (etc.)".

18. In this context, it is should be mentioned that the well-known Hungarian Racka sheep also has SHE horns (haven't they?).

19. Lines 268, 362: of course, the horns are used during fighting with competitors and predators, but it is a bit curious to state that SHE sheep and the wild ancestors look strong and aggressive; better omit these statements.

20. Figs. 4b and 4d can easily be combined, while the legends should mention more clearly that (as I understand) they show correlations with horn length and horn shape, respectively.

21. Lines 317-323: this paragraph can be omitted since the same points will be made in the Discussion (where it belongs anyway).

22. Line 331: "the Tibetan Plateau". Lines 331-333: awkward and superfluous sentence. Line 333: selective sweeps [plural].

23. Line 361: "naturalistic" refers to an artistic style; probably you mean a natural wildlife habitat.

24. Line 543: Goa -> goat

Methods

Are the methods appropriate to the aims of the study, are they well described, and are necessary controls included? Yes

Conclusions

Are the conclusions adequately supported by the data shown? Yes

Reporting Standards

Does the manuscript adhere to the journal's guidelines on minimum standards of reporting? Yes

Statistics

Are you able to assess all statistics in the manuscript, including the appropriateness of statistical tests used? No, I do not feel adequately qualified to assess the statistics.

Quality of Written English

Please indicate the quality of language in the manuscript: Needs some language corrections before being published

Declaration of Competing Interests

Please complete a declaration of competing interests, considering the following questions:

- Have you in the past five years received reimbursements, fees, funding, or salary from an organisation that may in any way gain or lose financially from the publication of this manuscript, either now or in the future?
- Do you hold any stocks or shares in an organisation that may in any way gain or lose financially from the publication of this manuscript, either now or in the future?
- Do you hold or are you currently applying for any patents relating to the content of the manuscript?
- Have you received reimbursements, fees, funding, or salary from an organization that holds or has applied for patents relating to the content of the manuscript?
- Do you have any other financial competing interests?
- Do you have any non-financial competing interests in relation to this paper?

If you can answer no to all of the above, write 'I declare that I have no competing interests' below. If your reply is yes to any, please give details below.

I declare that I have no competing interests

I agree to the open peer review policy of the journal. I understand that my name will be included on my report to the authors and, if the manuscript is accepted for publication, my named report including any attachments I upload will be posted on the website along with the authors' responses. I agree for my report to be made available under an Open Access Creative Commons CC-BY license (http://creativecommons.org/licenses/by/4.0/). I understand that any comments which I do not wish to be included in my named report can be included as confidential comments to the editors, which will not be published.

I agree to the open peer review policy of the journal

To further support our reviewers, we have joined with Publons, where you can gain additional credit to further highlight your hard work (see: https://publons.com/journal/530/gigascience). On publication of this paper, your review will be automatically added to Publons, you can then choose whether or not to claim your Publons credit. I understand this statement.

Yes
