# Supplementary material for: Whole-genome sequences of 89 Chinese sheep suggest role of RXFP2 in the development of unique horn phenotype as response to semi-feralization
Source: Gigascience. 2018 Mar 7;7(4):giy019. doi: 10.1093/gigascience/giy019 (PMC5905515; doi:10.1093/gigascience/giy019)
Supplement: Supplemental material [file giy019_supp.zip › Supplementary_Materials.revised3.doc]

**SUPPLEMENTARY MATERIALS**

**Whole genome sequences of 89 Chinese sheep suggest role of *RXFP2* in the development of unique horn phenotype as response to semi-feralization**

Zhangyuan Pan^†,1,2^, Shengdi Li^†,3,4^, Qiuyue Liu^†,1^, Zhen Wang^†,3^, Zhenkui Zhou^1^, Ran Di^1^, Benpeng Miao^3,4^, Wenping Hu^1^, Xiangyu Wang^1^, Xiaoxiang Hu^5^, Ze Xu^6^, Dongkai Wei^6^, Xiaoyun He^1^, Liyun Yuan^3^, Xiaofei Guo^1^, Benmeng Liang^1^, Ruichao Wang^3^, Xiaoyu Li^1^, Xiaohan Cao^1^, Xinlong Dong^1^, Qing Xia^1^, Hongcai Shi^7^, Geng Hao^8^, Jean Yang^9^, Cuicheng Luosang^9^, Yiqiang Zhao^5^, Mei Jin^10^, Yingjie Zhang^11^, Shenjin Lv^2^, Fukuan Li^2^, Guohui Ding^3,12^, Mingxing Chu*^,1^ & Yixue Li*^,3,12^

^1^Institute of Animal Science, Chinese Academy of Agricultural Sciences, Beijing, China.

^2^College of Agriculture and Forestry Science, Linyi University, Linyi, China

^3^Key Lab of Computational Biology, CAS-MPG Partner Institute for Computational Biology, Shanghai Institutes for Biological Sciences, Chinese Academy of Sciences, Shanghai, China.

^4^University of Chinese Academy of Sciences, Beijing, China;

^5^State Key Laboratory for Agrobiotechnology, China Agricultural University, Beijing, China.

^6^BasePair BioTechnology Co., Ltd., Suzhou, China.

^7^Institute of Biotechnology, Xinjiang Academy of Animal Science, Urumqi, China.

^8^Institute of Animal Science, Xinjiang Academy of Animal Science, Urumqi, China.

^9^Research Institute of Animal Science, Tibet Academy of Agricultural and Animal Husbandry Sciences, Lhasa, China.

^10^College of Life Science, Liaoning Normal University, Dalian, China.

^11^College of Animal Science and Technology, Agricultural University of Hebei, Baoding, China.

^12^Shanghai Center for Bioinformation Technology, Shanghai Industrial Technology Institute, Shanghai, China.

^†^These authors contributed equally to this work.

*These authors jointly directed this work.

Correspondence should be addressed to Y.L. (yxli@sibs.ac.cn) or M.C. (mxchu@263.net)

**Supplementary Figures**

**Supplementary Figure S1. Venn diagram of the variant comparison between the present study and dbSNP.**

All validated SNPs and indels from dbSNP build 143 were compared with the 42 million variants characterized in the present study.

**Supplementary Figure S2. Phylogeny tree of 99 sheep and outgroups.**

(**a**) Neighbor-joining tree of 99 sheep and two outgroup genomes (Texel sheep reference genome and goat genome). (**b**) A bootstrapping phylogeny tree constructed based on pruned genomic variants; number on the branch point indicate bootstrapping values.

**Supplementary Figure S3. Population structure analysis assuming 2-10 ancestral lineage.**

**Supplementary Figure S4. Migration events estimated by TreeMix.**

Migration events were estimated by TreeMix using number *m* = 0-5. Table at bottom showed the migration weights for different *m*.

**Supplementary Figure S5. Geographical locations of Tibetan sheep breeds.**

The location of Tibetan breeds were marked. Three lineages from the present study were showed in yellow dots (PT, Prairie Tibetan sheep; OL, Oula sheep; VT, Valley Tibetan sheep), and four lineages from another native sheep study (Yang et al. 2016) were showed in green (ZNQ, ZCD, ZLZ and ZRK).

**Supplementary Figure S6. Distributions of the minor Tajima's *D* and linkage disequilibrium (LD) in 10 sheep breeds.** (**a**) Tajima’s *D* was calculated using a 50-kb sliding window across the genome. (**b**) The mean correlation coefficient (r^2^) between SNP pairs decayed with increasing pair-wise distance.

**Supplementary Figure S7. Human population density in China.** The human population density map is downloaded as picture from CHINA-MIKE website (<http://www.china-mike.com/china-travel-tips/tourist-maps/china-population-maps/>). Human population density around YarluZangbu River where presented PT and OL sheep (blue dashed circle) is higher than the region where presented VT sheep (red dashed circle).

**Supplementary Figure S8. Genotyping using a SNaPshot screening assay of two differentiated SNPs in 1,155 domestic sheep.**

The allele frequencies of two differentiated protein-altering SNPs in *RXFP2* genes are shown: *RXFP2-1*, G>A (E641K); *RXFP2-2*, G>A (V627M). The length of the bar denotes the proportion of individuals carrying the genotype according to color. For each SNP, the frequency was calculated from the 99 WGS dataset (left) and from the 1155 SNaPshot dataset as shown.

**Supplementary Figure S9. Comparison between *RXFP2* haplotypes in Chinese sheep and the wild bighorn sheep.**

Genotype data of the bighorn sheep were downloaded from Dryad: doi:[10.5061/dryad.3f2t2](http://dx.doi.org/10.5061/dryad.3f2t2). Bar plots of allele frequency of 2,575 biallelic SNPs at *RXFP2* locus (assembly oviAri3, chromosome 10: 29,400,000 – 29,600,000 bp) were generated to visualize the haplotypic similarity between breeds, where orange bars denote fraction of the reference allele (identical to the reference genome) and purple bars denote that of the alternative allele. (TET, Tetons; SR, Sun River; WB, Whiskey Basin)

**Supplementary Figure S10. Genetic relationship between different sheep populations at *RXFP2* locus.**

Pair-wise genetic distance between breeds were calculated based on average allele frequency differences (average ΔAF) of 2,575 SNPs at the *RXFP2* locus. Phylogeny tree was generated based on the distance matrix using Neighbor-joining algorithm.

**Supplementary Figure S11. Features of different horn types in PT sheep.**

**Supplementary Figure S12. Association map of eight SNPs within or close to *RXFP2* and horn phenotypes.**

-log_10_(P value) of eight SNPs were showed under different regression models. Different genetic models were considered, including recessive (*AA*: 1, *Aa*: 0, *aa*: 0), additive (*AA*: 2, *Aa*: 1, *aa*: 0) and dominant models (*AA*: 1, *Aa*: 1, *aa*: 0). Missing points under dominant models were resulted from the absence of the homozygotes *aa*.

**Supplementary Figure S13. LD plot of ~1Mb region encompassing *RXFP2*.** Pair-wise LD (*D*’) was calculated using Haploview for each SNP combination within the genomic region from chr10:28,900,000 bp to chr10:30,100,000 bp. Variants were filtered by minor allele frequency (MAF < 0.2 were excluded) to reduce computational complexity. Positions of five tested genes were showed according to NCBI annotations.

**Supplementary Figure S14. Tissue expression profile of *RXFP2* and flanking genes in PT sheep.** (**a**) Soft horn can be developed from horn periosteum, then after cornification, form hard horn. The soft horn and horn periosteum were collected from the root of horn. (**b**) RT-PCR result of 13 tissues: 1, heart; 2, liver; 3, spleen; 4, lung; 5, kidney; 6, muscle; 7, brain; 8, ovary; 9, corpus uteri; 10, adipose; 11, thyroid; 12, soft horn; 13, horn periosteum. (**c**) Quantitative real-time PCR result of *RXFP2* in the same 13 tissue samples.

**Supplementary Figure S15. Tissue expression profile of *RXFP2* in Sonid sheep.**

1, Heart; 2, liver; 3, spleen; 4, lung; 5, kidney; 6, muscle; 7, brain; 8, ovary; 9, corpus uteri;10, adipose; 11, thyroid; 12, soft horn; 13, horn periosteum; 14, cerebellum; 15, hypothalamus; 16, hypophysis; 17, intestine; 18, duodenum.

**Supplementary Figure. S16. The expression level of *RXFP2* and flanking genes in PT soft-horn tissues with different horn types.**

**Supplementary Figure S17. Scatter plot of horn size and expressions of *RXFP2* and flanking genes.**

For each comparison, the Pearson’s correlation coefficient *r* was calculated. *P* value and fitting lines were calculated using linear regressions.

**Supplementary Figure S18.** **Horn features of other sheep breeds.**

Images of Racka sheep (<https://en.wikipedia.org/wiki/Racka>) and 4 different wild sheep were collected from internet: (1) Mouflon (<https://en.wikipedia.org/wiki/Mouflon>); (2) Urial (<https://en.wikipedia.org/wiki/Urial>); (3) Argali (https://en.wikipedia.org/wiki/Argali); and (4) Bighorn sheep (<https://species.wikimedia.org/wiki/Ovis_canadensis>).
